# Capturing factors associated with frailty using routinely collected electronic medical record data in British Columbia, Canada, primary care settings

**DOI:** 10.1017/S1463423625000337

**Published:** 2025-05-08

**Authors:** Manpreet Thandi, Morgan Price, Jennifer Baumbusch, Sharde Brown, Sabrina Wong

**Affiliations:** 1 School of Nursing, University of British Columbia, Vancouver, BC, Canada; 2 Department of Family Practice, University of British Columbia, Vancouver, BC, Canada; 3 Centre for Health Services and Policy Research, University of British Columbia, Vancouver, BC, Canada

**Keywords:** Aging, frailty, frailty index, frailty screening, primary care

## Abstract

**Background:**

Electronic medical record (EMR) systems in primary care present an opportunity to address frailty, a significant health concern for older adults. Researchers in the UK used Read codes to develop a 36-factor electronic frailty index (eFI), which produces frailty scores for patients in primary care settings.

**Aim:**

We aimed to translate the 36-factor eFI to a Canadian context.

**Methods:**

We used manual and automatic mapping to develop a coding set based on standardized terminologies used in Canada to reflect the 36 factors of the eFI. Manual mapping was completed independently by two coders, followed by group consensus among the research team. Automatic mapping was completed using Apelon TermWorks. We then used EMR data from the British Columbia Canadian Primary Care Sentinel Surveillance Network. We searched structured data fields related to diagnoses and reasons for patient visits to develop a list of free text terms associated with any of the 36 factors.

**Results and conclusions:**

A total of 3768 terms were identified; 3021 were codes. A total of 747 free text terms were identified from 527,521 reviewed data entries. Of the 36 frailty factors, 24 were captured mostly by codes; 7 mostly by free text; and 4 approximately equally by codes and free text. Three key findings emerged from this study: (1) It is difficult to capture frailty using only standardized terminologies currently used in Canada and a combination of standardized codes and free text terms better captures the complexity of frailty; (2) EMRs in primary care can be better optimized; (3) Output from this study allows for the development of a frailty screening algorithm that could be implemented in primary care settings to improve individual and system level outcomes related to frailty.

## Background

Increasing frailty in aging populations could overwhelm current healthcare systems. Canada is predicted to have over two million adults, ≥65 years, living with frailty in the next 10 years (The Canadian Frailty Network, 2020). Frailty, a state of increased vulnerability from physical, social, and cognitive factors, is associated with higher hospitalizations and long-term care admission rates, and higher rates of mortality (Clegg *et al.*, 2013; Li *et al.*, 2021; Chi *et al.*, 2021; Thandi *et al.*, 2024). Higher levels of frailty are associated with a greater risk of negative health-related outcomes such as falls, activity limitations, loss of independence, and declines in quality of life (Rockwood *et al.*, 2005; Fried *et al.*, 2001; Fedarko, 2011; Matuskik *et al.*, 2012; Shamliyan *et al.*, 2013; Morley *et al.*, 2013; Clegg *et al.*, 2013; Grenier, 2020; Langton *et al.*, 2020; Thandi *et al.*, 2024). Beyond physical limitations, frailty also affects social, emotional, and cognitive aspects of individuals’ health such as social isolation and loneliness, and challenges with mental health (Clegg *et al.*, 2013; Urquhart *et al.*, 2017; Grenier, 2020; Thandi *et al.*, 2024).

In order for interventions to be effective, early identification of frailty is needed (Travers *et al.*, 2019). Yet, early identification of frailty remains elusive because: loss of function is not easily detectable, it occurs slowly over time and there is no single definitive diagnostic test (e.g. HbA1C for diabetes) for frailty. Additionally, time constraints and overwhelming demands in primary care (Hoogendijk *et al.*, 2014) present a challenge for frailty identification.

Primary care clinicians are well placed to identify frailty early given their longitudinal and continuous relationship with patients. Early identification could delay or reduce frailty severity (Thandi, Brown, and Wong, 2021; Williamson *et al.*, 2020; Wong *et al.*, 2020) since more easily attainable interventions (e.g. completing 30 minutes of walking) could be achieved at a low cost to both the individual and system. With the majority of primary care using electronic medical records (EMR), a standardized, efficient, and consistent case-finding approach using these data could be applied (Aponte-Hao *et al.*, 2018; Williamson *et al.*, 2020; Wong *et al.*, 2020; Aponte-Hao *et al.*, 2021; Leghissa *et al.*, 2023).

Work done in the United Kingdom (UK) resulted in the development of a valid and reliable 36-item electronic frailty index (eFI) (Clegg *et al.*, 2016). The eFI automatically calculates frailty scores from existing EMR data. The 36-item eFI is useful and effective as a starting point in identifying and managing frailty (Devereux *et al.*, 2019; Hollinghurst *et al.*, 2019; Abbasi *et al.*, 2019). The eFI is a UK standard of primary care practice, having evidence of adequate construct and predictive validity in predicting mortality, hospitalizations, and nursing home admissions for increasingly frail patients (Clegg *et al.*, 2016). One of the main advantages of the eFI is that it can be translated into other contexts and countries, but this has not yet been completed in Canada.

We aimed to build an eFI specific to a Canadian context. This study addresses the research question: Can factors associated with frailty be captured using routinely collected EMR data in Canada?

## Methods

We conducted a descriptive study that mapped factors associated with frailty to existing clinical terminologies used in Canadian primary care settings.

In the development of the UK eFI, Clegg *et al.* (2016) followed published guidance in creating a frailty index using the cumulative deficit model (Searle *et al.*, 2008; Theou *et al.*, 2023). A series of searches were completed by UK researchers to identify Read codes for frailty factors. Read codes are components of a coded thesaurus of clinical terms, representing a term or a short phrase describing health-related concepts such as signs and symptoms, diagnoses, laboratory results, and information about social or functional circumstances (Clegg *et al.*, 2016). The purpose of clinical codes is to have a standardized method of terminology that allows for the sharing of clinical information across different platforms (Cardillo, 2015). Imposing common code use in documenting patient care provides the opportunity to increase clinical coordination and health service planning (Cardillo, 2015), including the case-finding of frail patients.

There is no comparable tool to the eFI that has been developed for Canadian primary care. We sought to build off Clegg et al.’s work and validate a tool already proven useful in primary care. To develop a tool similar to the eFI for use in Canada, adaptation was necessary because the UK and Canada use different standardized clinical terminology systems.

Before mapping the eFI, we completed a modified Delphi study to understand whether the UK eFI represents frailty from the perspectives of primary care clinicians and older adults in BC (Thandi *et al.*, 2024). Of the 36 frailty factors, 33 (92%) achieved consensus from the Delphi panel, providing content validation of the tool. Not only was this the first study to review the conceptualization of frailty in the eFI before adaptation or translation to another context, but did so with a diverse panel of primary care clinicians (family physicians, nurse practitioners, nurses, allied health team members) and older adults.

In this study, two mapping techniques were used to develop a list of codes that reflect the 36 frailty factors in the eFI. The two activities occurred in parallel, in that the results of each were merged together, with the second approach complementing the first approach.

1. Manual mapping in which we physically searched standardized terminology codes that reflect the frailty factors, including ICD9, ICD9-CM, LOINC, and ATC codes.
2. Automatic mapping using Apelon TermWorks software. Apelon TermWorks is a spreadsheet add-on for a computer-generated approach to mapping terms to national standards.

We also mapped free text terms found in Canadian primary care EMRs to the 36 factors.

### Data sources: ICD, LOINC, ATC codes, and free text terms

*Codes.* The International Classification of Diseases (ICD) (Government of British Columbia, 2017) includes clinical terminologies for diagnoses and procedures and is the main basis for health recording and statistics on disease (Centres for Disease Control and Prevention, 2024). The ICD 9th Revision (ICD-9) is the most commonly used version in Canadian primary care. Some clinicians are also coding using the International Classification of Disease 9th Revision, Clinical Modification (ICD9-CM), which is a modification of ICD9 allowing for more specific coding of diagnoses.

The Logical Observation Identifiers Names and Codes (LOINC, 2024) includes clinical terminologies used for tests, measurements, and observations.

Medications are represented by Anatomical Therapeutic Chemical Codes (ATC codes) (WHO, 2024). An ATC code is a unique code assigned to a medication according to the organ or system it works on and how it works. This classification system is maintained by the World Health Organization.

*Free Text Terms.* The data source for free text terms was BC-CPCSSN. In BC, the Canadian Sentinel Surveillance Network (CPCSSN) houses over 80 clinicians and data for more than 120,000 patients. CPCSSN extracts EMR data, standardizes these data into a common format, and makes data available for the purposes of quality improvement, disease surveillance, and research (Canadian Primary Care Sentinel Surveillance Network (CPCSSN), 2024). The following inclusion criteria were used for data extraction: data from 2017-07-01 to 2022-06-30 for patients ≥65 years as of 2022-06-30.

### Procedures

#### Manually developing a list of codes from standardized terminologies (ICD9, ICD9-CM, LOINC, ATC)

Two researchers (MT and SB) were trained for the mapping process through research team meetings and working through examples. Mapping meant manually reviewing the clinical terminologies and selecting those reflective of the specific frailty factors. Guidelines for mapping included:

1. Mapping based on clinical definitions of frailty factors.
2. If no clinical definition, look for a different definition and come to a consensus with the other researcher.
3. Developing a definition based on personal experience and clinical judgement.
4. Seeking validation with members of the research team (nurses, family physicians).

MT and SB independently mapped two frailty factors to ICD9 codes and then met for consensus between the two coders. Coders then met with the research team (SW and MP) to review coding decisions and come to a final consensus on codes to achieve face validity of the frailty construct. They followed the same steps for all frailty factors. If there was agreement on a code between MT and SB, the code was included; if there was disagreement, then the research team made the final decision about whether or not to include the code. Codes were included only once, even if they potentially fit with more than one frailty factor to prevent overcounting in the final frailty index.

ICD9-CM codes are also used in addition to ICD9 codes. All ICD9 codes are included within the more specific ICD9-CM terminology system. MT reviewed the established list of ICD-9 codes and added the more specific codes listed in the ICD9-CM that were relevant to each frailty factor.

LOINC is used to code for lab tests within EMRs. Only lab tests that were definitively diagnostic of specific frailty factors were included.

Finally, medications that were explicitly used for the management of a specific frailty factor were included in the final set of codes.

#### Adding to the list of codes from standardized terminologies with automatic mapping

Automatic mapping was an additional step to the manual mapping and was completed by MT using Apelon TermWorks software (Apelon TermWorks, 2024), a tool installed into Microsoft Excel. The purpose of this step was to ensure that codes were not missed through manual mapping. Terms searched in Apelon TermWorks were based on the Read codes from the original eFI and clinical judgement. Results were reviewed for relevancy and compared with manual mapping. Codes that were not relevant or were previously excluded were removed from the final list. Automatic mapping was completed for both ICD9 and ICD9-CM clinical terminologies. LOINC and ATC codes did not require mapping as CPCSSN already maintains a list for both terminologies that are extracted from EMRs.

#### Combining mapping

The final list of codes was a superset (i.e. including all relevant codes from all sources) reflecting each frailty factor. A superset set of codes allows for maximal inclusivity and is necessary given the heterogeneity of coding between clinicians where the same frailty factor can be coded in different ways. Maximal inclusivity enhances trustworthiness that frailty is adequately represented by the eFI. In the calculation of the frailty index score, when any **one** of the terms representing the specific frailty factor is present within the EMR, this indicates that the frailty factor is present.

#### Developing a list of semi-structured free text terms

The second part of this study involved developing a list of semi-structured free-text terms commonly used by primary care clinicians to reflect frailty factors. Functional diagnoses and non-biomedical factors are often lacking from medically based standard clinical terminologies. Thus, a list of additional concepts (defined as semi-structured free text terms, herein free text terms) was also created. These terms are semi-structured because only specific data fields within EMRs were searched.

Through a BC-CPCSSN data access request, we were granted access to EMR data meeting our inclusion criteria. Specifically, access to the ‘Encounter Diagnosis’ and ‘Encounter’ data tables was granted. These data tables reflect reasons for patients’ primary care visits and were manually searched for free text terms that were reflective of the 36 frailty factors of the eFI.

Criteria used to determine which free text terms to include were: (1) the name of the specific diagnosis, (2) a description or definition of the diagnosis (i.e., high blood pressure for hypertension), (3) different spellings (i.e., anemia and anaemia), (4) acronyms (i.e., CHF for heart failure), (5) terms that were equivalent to already included codes or UK Read codes (i.e., disability and disability form from UK Read codes for activity limitation; housing from UK Read codes for social vulnerability), and (6) clinical judgement (i.e., B12 injection as a treatment for anemia; blood pressure medication as a treatment for hypertension).

Records were searched until no new free text terms appeared. Unique free text terms were added to the maximal set of codes reflecting each frailty factor.

### Ethical considerations

This study was approved by the University of British Columbia Behavioural Research Ethics Board (H22-00692). To access the BC-CPCSSN data repository, a data access request was required and granted. Data were accessed through Population Data BC’s secure research environment.

### Analysis

Mapping to clinical codes was an iterative approach. The reliability of mapping between the two coders was addressed through multiple discussions between the research team. Descriptive analyses using Microsoft Excel summarized the numbers of codes and free text terms. Mapping to free text terms involved calculating and reporting the frequencies of free text terms.

## Results

The mapping process took place between January and August 2023. A total of 3768 terms were identified for the 36 factors of the eFI; 3021 (80.2%) were codes and 747 (19.8%) were free text terms. Table 1 summarizes the number of codes (ICD9, ICD9-CM, LOINC, ATC) and free text terms that reflect each of the 36 frailty factors. ICD9 and ICD9-CM codes were grouped together because all ICD9 codes are included within ICD9-CM coding terminologies. The complete list of codes and free text terms is available as a supplemental file.

**Table 1.** Summary of numbers of codes and free text terms reflecting frailty factors

Frailty Factor	Number of ICD9 and ICD9-CM codes	Number of LOINC codes	Total Number of Free Text Terms	Medication Prescriptions	Total Number of Codes and/or Terms
**Activity Limitation**	17	0	16	0	33
**Anaemia and Haematinic Deficiency**	82	5	22	0	109
**Arthritis**	219	4	23	0	246
**Atrial Fibrillation**	5	0	18	0	23
**Cerebrovascular Disease**	91	0	31	0	122
**Chronic Kidney Disease**	62	5	16	0	83
**Diabetes**	96	2	27	1	126
**Dizziness**	40	0	9	0	49
**Dyspnea**	6	0	16	0	22
**Falls**	24	0	21	0	45
**Foot Problems**	63	0	34	0	97
**Fragility Fracture**	845	0	6	0	851
**Hearing Impairment**	40	0	18	0	58
**Heart Failure**	20	1	3	0	24
**Heart Valve Disease**	31	0	13	0	44
**Housebound**	1	0	14	0	15
**Hypertension**	52	0	9	0	61
**Hypotension/Syncope**	11	0	30	0	41
**Ischemic Heart Disease**	80	2	25	0	107
**Memory and/or Cognitive Problems**	52	0	25	0	77
**Mobility and Transfer Problems**	33	0	34	0	67
**Osteoporosis**	7	0	5	0	12
**Parkinsonism and Tremor**	6	0	10	1	17
**Peptic Ulcer**	118	3	18	0	139
**Peripheral Vascular Disease**	80	0	22	0	102
**Polypharmacy**	0	0	1	0	1
**Requirement for Care**	11	0	10	0	21
**Respiratory Disease**	97	0	29	0	126
**Skin Ulcer**	33	0	14	0	47
**Sleep Disturbance**	63	0	32	0	95
**Social Vulnerability**	22	0	66	0	88
**Thyroid Disease**	54	6	14	1	75
**Urinary Incontinence**	19	0	14	0	33
**Urinary System Disease**	110	3	52	0	165
**Visual Impairment**	485	0	33	0	518
**Weight Loss and/or Anorexia**	12	0	17	0	29
**TOTALS**	**2987**	**31**	**747**	**3**	**3768**

### ICD-9 and ICD9-CM Codes

A total of 2987 codes from manual and automatic mapping were included from the ICD9 and ICD9-CM.

Sixteen terms from Apelon Termworks were included that were not originally in the list of codes. Details for specific terms and their sources are provided in the supplemental materials. Table 2 shows the percentage of additional codes (0.5%) that were added using Apelon Termworks.

**Table 2.** Percentage of additional codes added from Apelon TermWorks

Frailty Factor	Number of codes resulting from manual mapping	Number of additional terms added using Termworks	Percentage of Termworks terms for specified factor
Anaemia and Haematinic Deficiency	82	1	1.2%
Foot Problems	63	4	6.3%
Fragility Fracture	845	2	0.2%
Peripheral Vascular Disease	80	8	10.0%
Requirement for Care	11	1	9.1%

### LOINC codes

Thirty-one labs (Table 1), each with unique LOINC codes, were collaboratively decided upon to reflect specific frailty factors.

### Medication prescriptions

A total of three medication prescriptions (Table 1) were included. These medications were determined to be indicative of the respective frailty factor: insulin for diabetes, levothyroxine for thyroid disease, and levodopa-carbidopa for Parkinsonism.

### Free text terms

A total of 527,521 data entries were searched. These searches resulted in a total of 747 unique free text terms. Table 3 shows the top 5 free text terms for each frailty factor. The reported frequencies of terms listed include all possible combinations to avoid double counting frequencies. For example, the reported frequency of the term ‘disability’ already includes the term ‘disability form.’ This approach was used because in the subsequent development of a frailty algorithm, only the term ‘disability’ would need to be part of the algorithm to capture both terms. The two terms were however counted as two separate unique frailty factors in Table 1.

**Table 3.** Top 5 free text terms associated with each frailty factor (36 eFI frailty factors)

Frailty Factor	Top 5 Free Text Terms	Number of Times Term Appears in Search
n, (%)		
Activity Limitation (n = 917) (0.33%)	1. Disability 2. Weakness (excluding arm/no/hand/shoulder weakness and no weakness) 3. Parking permit 4. Trouble walking 5. Deteriorating health	452 (49.29%) 242 (26.39%) 36 (3.93%) 33 (3.60%) 31 (3.38%)
Anaemia and Haematinic Deficiency (n = 7,841) (2.85%)	1. Anemia 2. Iron deficiency 3. B12 deficiency 4. B12 injection 5. Anaemia	4516 (57.59%) 1233 (15.73%) 674 (8.60%) 529 (6.75%) 473 (6.03%)
Arthritis (n = 14,556) (5.29%)	1. Arthritis 2. Pain in joint 3. Gout 4. Arthropathy 5. Joint pain	5759 (39.56%) 4719 (32.42%) 3089 (21.22%) 394 (2.71%) 252 (1.73%)
Atrial Fibrillation (n = 8,934) (3.25%)	1. Atrial fib 2. Cardiac dysrhythmia 3. Flutter (excluding ventricular) 4. Afib 5. A fib	6750 (75.55%) 1231 (13.78%) 478 (5.35%) 126 (1.41%) 112 (1.25%)
Cerebrovascular Disease (n = 4,714) (1.71%)	1. Cerebrovascular disease 2. CVA 3. Stroke 4. Cerebral degeneration 5. Cerebral artery occlusion	2563 (54.37%) 656 (13.92%) 516 (10.95%) 136 (2.89%) 135 (2.86%)
Chronic Kidney Disease (n = 20,897) (7.59%)	1. Renal failure 2. Chronic kidney disease 3. CKD 4. Uremia 5. Proteinuria	7657 (36.64%) 4684 (22.41%) 2754 (13.18%) 332 (1.59%) 210 (1.00%)
Diabetes (n = 38,411) (13.95%)	1. Diabetes 2. DM (excluding adm, abdm, cdm, sdm) 3. Insulin 4. Impaired fasting glucose 5. Diabetic	27,495 (71.58%) 7898 (20.56%) 1020 (2.66%) 967 (2.52%) 560 (1.46%)
Dizziness (n = 5,333) (1.94%)	1. Vertigo 2. Dizziness 3. Dizzy *Remaining terms are captured within the above terms.*	2417 (45.32%) 2302 (43.17%) 614 (11.51%)
Dyspnea (n = 2,768) (1.01%)	1. Dyspnea 2. Shortness of breath 3. SOB 4. Dyspnoea 5. Breathing issue	1305 (47.15%) 660 (23.84%) 515 (18.61%) 69 (2.49%) 60 (2.17%)
Falls (n = 2,368) (0.86%)	1. Fall (excludes ‘Fallopian’) 2. Fell *Remaining terms are captured within the above terms.*	1864 (78.72%) 504 (21.28%)
Foot Problems (n = 2,426) (0.88%)	1. Plantar wart 2. Foot care 3. Foot pain 4. Corns 5. Plantar fasciitis	843 (34.75%) 653 (26.92%) 419 (17.27%) 329 (13.56%) 269 (11.09%)
Fragility Fracture (n = 5,355) (1.95%)	1. Fracture 2. # (exclude # terms related to vaccine, tpi, pain, vax #)* 3. Broken (excludes broken teeth/tooth) 4. Cast *Remaining terms are captured within the above terms.*	3616 (67.53%) 1448 (27.04%) 181 (3.38%) 110 (2.05%)
Hearing Impairment (n = 1,237) (0.45%)	1. Hearing loss 2. Deaf 3. Hearing aid 4. Can’t hear 5. Hard of hearing	857 (69.28%) 221 (17.87%) 39 (3.15%) 27 (2.18%) 19 (1.54%)
Heart Failure (n = 11,530) (4.19%)	1. Heart failure 2. CHF *Remaining terms are captured within the above terms.*	8386 (72.73%) 3144 (27.27%)
Heart Valve Disease (n = 747) (0.27%)	1. Valve disorder 2. Aortic stenosis 3. Tricuspid regurgitation 4. Mitral regurgitation 5. Mitral valve prolapse	297 (39.76%) 281 (37.62%) 49 (6.56%) 39 (5.22%) 21 (2.81%)
Housebound (n = 1,489) (0.54%)	1. Home visit (excludes care home visit, nursing home visit) 2. Home care 3. Home health 4. Home and community care 5. Home support	972 (65.28%) 186 (12.49%) 120 (8.06%) 51 (3.43%) 40 (2.69%)
Hypertension (n = 50,809) (18.46%)	1. Hypertension 2. HTN (except ‘ghtn’) 3. Elevated blood pressure 4. Elevated BP 5. Blood pressure medication	46,577 (91.67%) 3503 (6.89%) 681 (1.34%) 35 (0.07%) 27 (0.05%)
Hypotension/Syncope (n = 2,479) (0.90%)	1. Syncope 2. Hypotension 3. Orthostatic 4. Lightheaded 5. Faint	929 (37.47%) 903 (36.43%) 327 (13.19%) 83 (3.35%) 69 (2.78%)
Ischemic Heart Disease (n = 12,795) (4.65%)	1. Heart disease 2. Chest pain 3. Coronary atherosclerosis 4. Coronary artery disease 5. ASHD	4440 (34.70%) 2280 (17.82%) 1853 (14.48%) 1377 (10.76%) 956 (7.47%)
Memory and/or Cognitive Problems (n=9,091) (3.30%)	1. Dementia 2. Alzheimer 3. Memory 4. Confusion 5. Cognitive impairment	6534 (71.87%) 1071 (11.78%) 590 (6.49%) 328 (3.61%) 312 (3.43%)
Mobility and Transfer Problems (n = 827) (0.30%)	1. Gait 2. Ataxia 3. Scooter 4. Hemiplegia 5. Walker	163 (19.71%) 115 (13.91%) 74 (8.95%) 68 (8.22%) 53 (6.41%)
Osteoporosis (n = 3,817) (1.39%)	1. Osteoporosis 2. Osteopenia 3. Low bone mass 4. Low bone density 5. Osteomalacia	3291 (86.22%) 413 (10.82%) 87 (2.28%) 25 (0.65%) 1 (0.03%)
Parkinsonism and Tremor (n = 2,388) (0.87%)	1. Parkinson 2. Tremor 3. Extrapyramidal 4. Tremour 5. Tremulousness	1380 (57.79%) 785 (32.87%) 168 (7.04%) 54 (2.26%) 1 (0.04%)
Peptic Ulcer (n = 1,240) (0.45%)	1. Blood in stool 2. Peptic ulcer 3. Helicobacter pylori 4. H pylori 5. Gastrointestinal haemorrhage	267 (21.53%) 160 (12.90%) 143 (11.53%) 121 (9.76%) 112 (9.03%)
Peripheral Vascular Disease (n = 3,161) (1.15%)	1. Peripheral vascular disease 2. Embolism (excluding cerebral embolism) 3. Thrombosis (excluding cerebral thrombosis) 4. DVT 5. PVD	743 (23.51%) 733 (23.19%) 581 (18.38%) 295 (9.33%) 220 (6.96%)
Polypharmacy (n = 20) (0.01%)	1. Polypharmacy	20 (100.00%)
Requirement for Care (n = 977) (0.35%)	1. Palliative care 2. Needs help 3. ADLs 4. Groceries 5. Banking	908 (92.94%) 18 (1.84%) 13 (1.33%) 9 (0.92%) 7 (0.72%)
Respiratory Disease (n = 18,610) (6.76%)	1. COPD 2. Asthma 3. Bronchitis 4. Chronic respiratory condition 5. Emphysema	6549 (35.19%) 3675 (19.75%) 3143 (16.89%) 2077 (11.16%) 1219 (6.55%)
Skin Ulcer (n = 2,720) (0.99%)	1. Open wound 2. Skin eruption 3. Wound care 4. Chronic ulcer 5. Skin Ulcer	1069 (39.30%) 1063 (39.08%) 170 (6.25%) 147 (5.40%) 115 (4.23%)
Sleep Disturbance (n = 8,062) (2.93%)	1. Insomnia 2. Sleep apnea 3. Sleep disturbance 4. Sleep disorder 5. Not sleeping	5417 (67.19%) 1694 (21.01%) 486 (6.03%) 109 (1.35%) 68 (0.84%)
Social Vulnerability (n = 1,868) (0.68%)	1. Housing 2. Grief 3. Social work 4. Bereavement 5. SW support	384 (20.56%) 222 (11.88%) 219 (11.72%) 206 (11.03%) 155 (8.30%)
Thyroid Disease (n = 7844) (2.85%)	1. Hypothyroid 2. Thyroid deficiency 3. Thyroid disorder 4. Thyroid nodule 5. Hyperthyroid	7107 (90.60%) 226 (2.88%) 130 (1.66%) 126 (1.61%) 93 (1.19%)
Urinary Incontinence (n = 1,123) (0.41%)	1. Incontinence (excluding faecal/fecal incontinence and incontinence of feces/faeces) 2. Atony of bladder 3. Bladder atony 4. Bladder control *Remaining terms are captured within the above terms.*	1092 (97.24%) 14 (1.25%) 9 (0.80%) 8 (0.71%)
Urinary System Disease (n=14,536) (5.28%)	1. Cystitis 2. UTI (excluding utic, utin, utio, utis) 3. Urinary tract infection 4. Hematuria 5. Dysuria	3707 3132 (21.55%) 2481 (17.07%) 1045 (7.19%) 832 (5.72%)
Visual Impairment (n = 2,733) (1.00%)	1. Cataract 2. Macular degeneration 3. Low vision 4. Blurred vision 5. Visual disturbance	1636 (59.86%) 257 (9.40%) 150 (5.49%) 147 (5.38%) 145 (5.31%)
Weight Loss and/or Anorexia (n=635) (0.23%)	1. Weight loss 2. Wt loss 3. Anorexia 4. Malnutrition 5. No appetite	442 (69.61%) 66 (10.39%) 58 (9.13%) 23 (3.62%) 15 (2.36%)

*The symbol ‘#’ is a way of representing a fracture used by clinicians.

We also needed to exclude certain terms from the search. For example, for atrial fibrillation, we wanted to capture ‘flutter’ but not ‘ventricular flutter,’ thus ‘ventricular’ was excluded from the search. A detailed list of the free text terms for each frailty factor, their frequencies, and indications of combinations and exclusion criteria are available as supplemental material.

### Codes vs. free text

Table 4 shows the percentages of frailty factors captured mostly by codes, factors captured mostly by free text, and codes captured approximately equally. If the difference between number of codes and free text was less than 10%, that frailty factor was considered to be approximately equally represented by codes and free text.

**Table 4.** Frailty factors captured by codes and free text

Frailty Factors Captured Mostly by Codes (% of terms captured by codes)	Frailty Factors Captured Mostly by Free Text (% of terms captured by free text terms)	Frailty Factors Captured Approximately Equally by Codes and Free Text (% difference between number of codes and free text terms)
(n = 35)*		
Anaemia/Haematinic Deficiency (79.82%) Arthritis (90.65%) Cerebrovascular Disease (74.59%) Chronic Kidney Disease (80.72%) Diabetes (78.57%) Dizziness (81.63%) Foot Problems (64.95%) Fragility Fracture (99.29%) Hearing Impairment (68.97%) Heart Failure (87.50%) Heart Valve Disease (70.45%) Hypertension (85.25%) Ischemic Heart Disease (76.64%) Memory/Cognitive Problems (67.53%) Osteoporosis (58.33%) Peptic Ulcer (87.05%) Peripheral Vascular Disease (78.43%) Respiratory Disease (76.98%) Skin Ulcer (70.21%) Sleep Disturbance (66.32%) Thyroid Disease (81.33%) Urinary incontinence (57.58%) Urinary System Disease (68.48%) Visual Impairment (93.63%)	Atrial Fibrillation (78.26%) Dyspnea (72.73%) Housebound (93.33%) Hypotension/Syncope (73.17%) Parkinsonism and Tremor (58.82%) Social Vulnerability (75.00%) Weight Loss/Anorexia (58.62%)	Activity Limitation (3.04%) Falls (6.66%) Mobility/Transfer problems (1.5%) Requirement for Care (4.76%)

*Note: 35 (not 36) frailty factors are listed in this table because polypharmacy was determined by whether a patient is prescribed 5 or more medications in the past 12 months.

Twenty-four of the 36 eFI frailty factors were captured mostly by codes; 7 mostly by free text; and 4 approximately equally by both codes and free text. Polypharmacy was not placed in a category as the presence of this factor was determined by whether a patient is prescribed 5 or more medications.

The final output from this study was a list of 3021 codes (ICD9, ICD9-CM, LOINC, ATC medications) and 747 free text terms that reflect the 36 eFI frailty factors.

## Discussion

This work results in an eFI available for use with Canadian EMRs, using standardized terminologies and ontologies. Three key findings emerged from this study.

### It is difficult to capture the complexity of frailty using only the standardized terminologies/coding systems currently widely used in Canada

In primary care settings, EMRs are mainly used for biomedical billing codes with standardized clinical terminologies. Standardized clinical terminologies are most useful for diseases and chronic conditions such as diabetes and hypertension and less useful in capturing the contextual aspects of individuals’ lives that are more difficult to identify but might contribute to becoming increasingly frail. Our free text search allowed us to mitigate this challenge. For example, the frailty factor ‘housebound’ was reflected by one clinical code, but by 14 free text terms. The factor ‘social vulnerability’ was reflected by 22 clinical codes and 66 free text terms. Additionally, factors such as dyspnea, hypotension/syncope, Parkinsonism/tremor, and weight loss which indicate symptoms that can be related to multiple chronic conditions appeared to be better captured by free text terms.

We also found that although chronic conditions often had clear associated clinical codes, there were several free text terms that also captured the condition. A key reason for this finding was that clinicians were entering clinical codes in designated data fields of the EMR and also entering similar terms in free text fields, while sometimes using abbreviations, different spellings, or acronyms. Including both codes and free text ensures that we don’t miss the opportunity to capture a factor, especially if it was mis-entered in the EMR, or if a clinician is using the free text fields to capture a patient’s history and/or multiple health issues because only one code can be billed.

It was important to capture the frailty factors using both the codes and the free text in order to have a superset of terms that can represent the construct of frailty. Including both codes and free text also ensures that we don’t miss the opportunity to capture a factor, especially if it was mis-entered in the EMR, or if a clinician is using the free text fields to capture a patient’s history and/or multiple health issues because only one code can be billed. Semantic equivalence declares that the data in free text and data in billing codes (i.e. elements from two different vocabularies/data sources) have similar meanings, making it important to include both in developing a comprehensive screening tool. If we focused only on codes, or only on free text, we risk creating gaps in knowledge because certain terms will be captured better by medical billing codes and others will be captured better by free text charting/data entry.

### EMRs in primary care hold a lot of potential and can be better optimized

There are substantial opportunities to use EMRs to enhance clinical decision support and continuity of care. Common tasks supported by EMRs include electronic prescribing of medication, looking up patient notes, electronic reminders for patient care, checking drug interactions, generating patient lists by diagnosis, providing patient summaries, and billing purposes (Chang and Gupta, 2015; Rimmer *et al.*, 2014). Primary care EMRs have great potential beyond these tasks to facilitate effective ways to monitor, treat, and improve patients’ health outcomes; improve practice efficiency, patient safety, and patient care; and improve the continuity of care for chronic conditions (Chang and Gupta, 2015).

One such use of the EMR is to screen for frailty in clinical practice. Clegg et al.’s work (2016) is an excellent example of how routinely collected data by primary care clinicians can be used to assign patients a frailty score. An electronic frailty score can consistently and efficiently screen for frailty, allowing for earlier intervention and management, using both standardized clinical terminologies and free text fields. Additionally, there is potential to explore the possibility of coupling frailty screening with generative AI to automate different tasks and recognize patterns by using human-made computing algorithms (Well AI Voice, 2024).

### Output from this study allows for the development of a frailty screening algorithm

Output from this study allows for the development of a reliable and valid frailty screening algorithm that can automatically calculate frailty scores (currently underway). The development of an automated clinical decision support tool forms the basis for early frailty identification and subsequent treatment options based on clinical preventive guidelines and research. A validated frailty algorithm using primary care EMRs would allow for frailty screening in a time-effective and efficient way. Similar to the UK 36-item eFI, the algorithm will calculate a continuous frailty score by dividing the number of frailty factors present in a patient’s medical record (as indicated by the presence of at least one code or free text term reflecting that frailty factor) by the total number of possible factors, 36. For example, if the algorithm finds that a patient has 8 of the 36 frailty factors documented in their EMR, their frailty score would be 8/36 or 0.22.

#### Impact on future frailty research and clinical practice

We hope that our broad research study will result in an electronic frailty screening tool that can use existing EMR data to calculate frailty scores for patients. This study maps frailty factors to standardized clinical terminologies and free-text terms, and our subsequent study will develop and test an algorithm based on these clinical terminologies and free-text terms. Potential impacts we hope our work will have on frailty screening in clinical practice include (Thandi *et al.*, 2024): (1) Optimizing EMR use to include frailty screening and identification of patients who require further frailty assessment and follow-up. (2) Contributing to quick and efficient frailty screening, mitigating the challenge of time that often inhibits clinicians from screening patients for frailty. (3) Implementing the eFI in BC primary care settings for further validation and ultimately becoming a provincial standard of practice. Table 5 provides a summary of this work and its significance.

## Limitations

The free text entries we examined were not interdisciplinary but described how physicians are using free text terms. Future research could also consider extracting notes from multi-disciplinary primary care team members such as through the extraction of progress notes for the representation of social determinants of health and/or broader contextual factors of individuals’ lives to capture frailty more holistically and to add to the strengths of our results.

Although we reviewed data from 22 different BC primary care practices, we did not assess the quality of EMR data within or across these practices within the scope of this study. Using existing EMR data to produce frailty scores would need to assume that there is adequate data being documented. Thus, we would need to assume that clinicians have assessed for and documented any relevant issues in order for the frailty score to be accurate. Implementing an electronic screening tool within primary care settings may also pose a challenge due to the interoperability of EMR systems.

Additionally, differences in clinical terminologies between the UK and Canada were evident. The read codes used in the UK incorporate activities such as ‘referral to diabetes nurse’ or ‘home visit’ whereas codes such as these are not readily available within the ICD9 or ICD9-CM. For example, the frailty factor ‘housebound’ did not have an equivalent code within the ICD9. Often when physicians input data, there is an activity code that is paired with an ICD code. Thus, if a clinician is doing a home visit (indicative of the frailty factor ‘housebound’), the home visit would need to be entered as an activity code and not an ICD code, thus would be missed in the calculation of the frailty score. Our free text search did capture ‘home visit’ to reflect the frailty factor ‘housebound’ demonstrating the importance of needing to include free text terms in this research. Similarly, referrals are not captured within the ICD9.

Finally, we used EMR data from primary care in British Columbia. We searched a total of 527,521 free text data entries and mapped frailty terms to clinical terminology systems that are used across Canadian provinces. It is likely that using data from other jurisdictions would have similar results. It is possible that free text used by clinicians in other provinces and territories could capture frailty differently, thus this work should be replicated with a pan-Canadian EMR dataset.

## Conclusion

Three key findings emerged from this study: (1) It is difficult to capture frailty using only the standardized terminologies currently widely used in Canada; a combination of standardized codes and free text terms better captures the complexity of frailty. (2) EMRs in primary care hold a lot of potential and can be better optimized. (3) Output from this study allows for the development of a frailty screening algorithm and subsequently, a standardized frailty screening tool that can be implemented into BC primary care settings to improve outcomes related to frailty at both an individual and system level.

A standard approach to frailty identification using existing EMR data provides an exciting and significant opportunity to identify, manage, and/or prevent frailty to reduce negative patient health outcomes and often preventable healthcare expenditures. Implementing the eFI within primary care clinical practice settings has the potential to improve the continuity and coordination of care while alleviating the time burden related to the identification and management of frailty. The output from this study will inform a subsequent study (currently underway) to test an algorithm that can calculate frailty scores for patients.

**Table 5.** Summary and significance of this work

Problem	• There is currently no equivalent to the electronic frailty index to screen for frailty in Canada.
**What is already known**	• It is important to screen for frailty early in patients’ health trajectories in order to manage, treat, or reverse negative health outcomes associated with frailty. • The 36-item electronic frailty index, developed in the UK, has been stated to be an extremely efficient, useful, and effective starting point in identifying and managing frailty.
**What this paper adds**	• This research built off of the UK 36-factor eFI to develop an electronic frailty index specific to a Canadian context. • Output from this study allows for development of a frailty screening algorithm that will use existing electronic medical record data to calculate frailty scores for patients in British Columbia, Canada primary care settings.
**Goals of broader work**	• Optimizing EMR use to include frailty screening and identification of patients who require further frailty assessment and follow up. • Contributing to quick and efficient frailty screening, mitigating the challenge of time that often inhibits clinicians from frailty screening. • Implementing the frailty screening tool in BC primary care settings for pilot testing to ultimately become a provincial standard of practice.

## List of Abbreviations (in alphabetical order)

Anatomical Therapeutic Classification = ATC

British Columbia = BC

Canadian Sentinel Surveillance Network = CPCSSN

Clinical Terms Version 3 = CTV3

Electronic Frailty Index = eFI

Electronic Medical Record = EMR

Systemized Nomenclature of Medicine Clinical Terms = SNOMED-CT

The International Classification of Diseases 9^th^ Revision = ICD9

The International Classification of Diseases 9^th^ Revision – Clinical Modification = ICD9-CM

The Logical Observation Identifier Names and Codes = LOINC

United Kingdom = UK

## Supporting information

Thandi et al. supplementary material 1 Thandi et al. supplementary material

Thandi et al. supplementary material 2 Thandi et al. supplementary material

## Data Availability

All data generated or analyzed during this study are included in this published article (and its supplementary information files). Any further requested information is available from the corresponding author on reasonable request.

## Supplementary material

The supplementary material for this article can be found at https://doi.org/10.1017/S1463423625000337

## Authors’ Information

MT is a PhD candidate at the University of British Columbia.

SW is MT’s dissertation supervisor.

MP and JB are members of MT’s dissertation committee.

SB is a nurse and previous graduate student at the University of British Columbia
