# Supplementary material for: Capturing factors associated with frailty using routinely collected electronic medical record data in British Columbia, Canada, primary care settings
Source: Prim Health Care Res Dev. 2025 May 8;26:e41. doi: 10.1017/S1463423625000337 (PMC12099269; doi:10.1017/S1463423625000337)
Supplement: Thandi et al. supplementary material 1 — Thandi et al. supplementary material [file S1463423625000337sup001.docx]

Using primary care electronic medical record data in British Columbia, Canada to develop an electronic frailty index: Mapping of read codes used to reflect frailty factors in the UK 36-factor eFI to clinical terminologies used in BC, Canada: Final Results

PhD Candidate: Manpreet Thandi

Dissertation Committee: Sabrina Wong (supervisor), Morgan Price, Jennifer Baumbusch

University of British Columbia, School of Nursing

Table of Contents

[Understanding this Document 4](#_Toc170119459)

[eFI Frailty Factors (n=36) 5](#_Toc170119460)

[Activity Limitation 5](#_Toc170119461)

[Anaemia and Haematinic Deficiency 6](#_Toc170119462)

[Arthritis 10](#_Toc170119463)

[Atrial fibrillation 19](#_Toc170119464)

[Cerebrovascular disease 20](#_Toc170119465)

[Chronic kidney disease 24](#_Toc170119466)

[Diabetes 27](#_Toc170119467)

[Dizziness 32](#_Toc170119468)

[Dyspnea 34](#_Toc170119469)

[Falls 35](#_Toc170119470)

[Foot problems 36](#_Toc170119471)

[Fragility fracture 40](#_Toc170119472)

[Hearing impairment 43](#_Toc170119473)

[Heart failure 46](#_Toc170119474)

[Heart valve disease 47](#_Toc170119475)

[Housebound 49](#_Toc170119476)

[Hypertension 49](#_Toc170119477)

[Hypotension/syncope 52](#_Toc170119478)

[Ischemic heart disease 53](#_Toc170119479)

[Memory and/or cognitive problems 57](#_Toc170119480)

[Mobility and transfer problems 60](#_Toc170119481)

[Osteoporosis 62](#_Toc170119482)

[Parkinsonism and Tremor 63](#_Toc170119483)

[Peptic Ulcer 64](#_Toc170119484)

[Peripheral Vascular Disease 69](#_Toc170119485)

[Polypharmacy 72](#_Toc170119486)

[Requirement for care 73](#_Toc170119487)

[Respiratory Disease 74](#_Toc170119488)

[Skin Ulcer 78](#_Toc170119489)

[Sleep disturbance 80](#_Toc170119490)

[Social vulnerability 84](#_Toc170119491)

[Thyroid Disorder 87](#_Toc170119492)

[Urinary incontinence 90](#_Toc170119493)

[Urinary system disease 91](#_Toc170119494)

[Visual impairment 97](#_Toc170119495)

[Weight loss and/or anorexia 102](#_Toc170119496)

[Additional Frailty Factors Suggested by Panelists (n=13) 104](#_Toc170119497)

[Cancer 104](#_Toc170119498)

[Challenges to healthcare access 109](#_Toc170119499)

[Chronic pain/back pain 110](#_Toc170119500)

[Communication challenges 112](#_Toc170119501)

[Fecal incontinence 113](#_Toc170119502)

[Inadequate Diet and Nutrition 113](#_Toc170119503)

[Liver failure/cirrhosis 115](#_Toc170119504)

[Mental health challenges 116](#_Toc170119505)

[Medication noncompliance 126](#_Toc170119506)

[Poverty/financial difficulties 126](#_Toc170119507)

[Race/ethnic disparity 127](#_Toc170119508)

[Sedentary/low activity levels 127](#_Toc170119509)

[Substance use/misuse 129](#_Toc170119510)

# Understanding this Document

- In this document, there is a table for each of the 36 frailty factors (and the 13 additional suggested frailty factors). Each table includes a list of codes that reflect the frailty factor, and the source of the code (either ICD9, ICD9-CM, Termworks, LOINC or CPCSSN free text)
- Termworks is an automatic mapping software that was used as a complementary mapping method to our manual mapping. The specific terms that were searched within Termworks are specified below each frailty factor’s table. For the additional suggested factors, search terms were based on what panelists suggested as explanations of the factors.
- LOINC codes are specific to lab tests. Mapping was not required for lab tests as CPCSSN data managers already have a list of codes for the labs they extract.
- A * indicates that all sub-codes below the specific code are included.
- Please see the below table explaining the highlighting of codes in this document:

| No highlight indicates agreement between researchers and between researchers and automatic mapping |
| --- |
| Green highlight indicates researcher agreement but did not appear in the automatic mapping |
| Pink highlight indicates codes that appeared in automatic mapping but not during researchers’ manual mapping – collaborative decisions were subsequently made to include these codes |

# eFI Frailty Factors (n=36)

## Activity Limitation

| **Codes and Terms** | **Source(s)** |
| --- | --- |
| \| 719.7 DIFFICULTY IN WALKING (Under "arthropathies") \| \| --- \| \|  \| \| V49 PROBLEMS WITH LIMBS AND OTHER PROBLEMS \| \| V49.0 DEFICIENCIES OF LIMBS \| \| V49.1 MECHANICAL PROBLEMS WITH LIMBS \| \| V49.2 MOTOR PROBLEMS WITH LIMBS \| \| V49.3 SENSORY PROBLEMS WITH LIMBS \| \| V49.4 DISFIGUREMENTS OF LIMBS \| \| V49.7 Lower limb amputation status \| \| V49.70 Unspecified level lower limb amputation status \| \| V49.73 Foot amputation status \| \| V49.74 Ankle amputation status \| \| V49.75 Below knee amputation status \| \| V49.76 Above knee amputation status \| \| V49.77 Hip amputation status \| \|  \| \| V52.1 Fitting and adjustment of artificial leg (complete) (partial) \| \| V68.01 Disability examination \| \| 799.3 Debility, unspecified \| \|  \| \| Debilitation \| \| Debility \| \| Deconditioning \| \| Deteriorating \| \| Difficulty walking \| \| Disability \| \| Disability form \| \| Lower extremity weakness \| \| General deconditioning \| \| Hard to walk \| \| Leg weakness \| \| Muscle wasting \| \| Parking permit \| \| Sarcopenia \| \| Trouble walking \| \| Weakness \| \|  \| | \| **ICD9/TERMWORKS** \| \| --- \| \|  \| \| **ICD9** \| \| **ICD9** \| \| **ICD9** \| \| **ICD9** \| \| **ICD9** \| \| **ICD9** \| \| **ICD9-CM** \| \| **ICD9-CM** \| \| **ICD9-CM** \| \| **ICD9-CM** \| \| **ICD9-CM** \| \| **ICD9-CM** \| \| **ICD9-CM** \| \|  \| \| **ICD9-CM** \| \| **ICD9-CM/TERMWORKS** \| \| **ICD9-CM** \| \|  \| \| **CPCSSN Free Text** \| \| **CPCSSN Free Text** \| \| **CPCSSN Free Text** \| \| **CPCSSN Free Text** \| \| **CPCSSN Free Text** \| \| **CPCSSN Free Text** \| \| **CPCSSN Free Text** \| \| **CPCSSN Free Text** \| \| **CPCSSN Free Text** \| \| **CPCSSN Free Text** \| \| **CPCSSN Free Text** \| \| **CPCSSN Free Text** \| \| **CPCSSN Free Text** \| \| **CPCSSN Free Text** \| \| **CPCSSN Free Text** \| \| **CPCSSN Free Text** \| |

Termworks search terms: activity $+ limitation, activity limitation, walking, activity, disability

## Anaemia and Haematinic Deficiency

| **Codes and Terms** | **Source(s)** |
| --- | --- |
| \| \| 266 DEFICIENCY OF 'B'-COMPLEX COMPONENTS \| \| --- \| \| 266.2 OTHER 'B'-COMPLEX DEFICIENCIES \| \| 266.9 UNSPECIFIED VITAMIN 'B' DEFICIENCY \| \|  \| \| 275.0 DISORDERS OF IRON METABOLISM \| \| 275.01 Hereditary hemochromatosis \| \| 275.02 Hemochromatosis due to repeated red blood cell transfusions \| \| 275.03 Other hemochromatosis \| \| 275.09 Other disorders of iron metabolism \| \|  \| \| DISEASES OF BLOOD AND BLOOD FORMING ORGANS \| \| 280 IRON DEFICIENCY ANAEMIAS \| \| 280.0 Iron deficiency anemia secondary to blood loss (chronic) \| \| 280.1 Iron deficiency anemia secondary to inadequate dietary iron intake \| \| 280.8 Other specified iron deficiency anemias \| \| 280.9 Iron deficiency anemia, unspecified \| \|  \| \| 281 OTHER DEFICIENCY ANAEMIAS \| \| 281.0 PERNICIOUS ANAEMIA \| \| 281.1 OTHER VITAMIN-'B12'-DEFICIENCY ANAEMIA \| \| 281.2 FOLATE-DEFICIENCY ANAEMIA \| \| 281.3 OTHER SPECIFIED MEGALOBLASTIC ANAEMIAS, NOT ELSEWHERE CLASSIFIED \| \| 281.4 PROTEIN-DEFICIENCY ANAEMIA \| \| 281.8 ANAEMIA ASSOCIATED WITH OTHER SPECIFIED NUTRITIONAL DEFICIENCY \| \| 281.9 UNSPECIFIED \| \|  \| \| 282 HEREDITARY HAEMOLYTIC ANAEMIAS \| \| 282.0 HEREDITARY SPHEROCYTOSIS \| \| 282.1 HEREDITARY ELLIPTOCYTOSIS \| \| 282.2 ANAEMIA DUE TO DISORDERS OF GLUTATHIONE METABOLISM \| \| 282.3 OTHER HAEMOLYTIC ANAEMIAS DUE TO ENZYME DEFICIENCY \| \| 282.4 THALASSAEMIAS \| \| 282.40 Thalassemia, unspecified \| \| 282.41 Sickle-cell thalassemia without crisis \| \| 282.42 Sickle-cell thalassemia with crisis \| \| 282.43 Alpha thalassemia \| \| 282.44 Beta thalassemia \| \| 282.45 Delta-beta thalassemia \| \| 282.46 Thalassemia minor \| \| 282.47 Hemoglobin E-beta thalassemia \| \| 282.49 Other thalassemia \| \| 282.5 SICKLE-CELL TRAIT \| \| 282.6 SICKLE-CELL ANAEMIA \| \| 282.60 Sickle-cell disease, unspecified \| \| 282.61 Hb-SS disease without crisis \| \| 282.62 Hb-SS disease with crisis \| \| 282.63 Sickle-cell/Hb-C disease without crisis \| \| 282.64 Sickle-cell/Hb-C disease with crisis \| \| 282.68 Other sickle-cell disease without crisis \| \| 282.69 Other sickle-cell disease with crisis \| \| 282.7 OTHER HAEMOGLOBINOPATHIES \| \| 282.8 OTHER \| \| 282.9 UNSPECIFIED \| \|  \| \| 283 ACQUIRED HAEMOLYTIC ANAEMIAS \| \| 283.0 AUTOIMMUNE HAEMOLYTIC ANAEMIAS \| \| 283.1 NON-AUTOIMMUNE HAEMOLYTIC ANAEMIAS \| \| 283.10 Non-autoimmune hemolytic anemia, unspecified \| \| 283.11 Hemolytic-uremic syndrome \| \| 283.19 Other non-autoimmune hemolytic anemias \| \| 283.2 HAEMOGLOBINURIA DUE TO HAEMOLYSIS FROM EXTERNAL CAUSES \| \| 283.9 UNSPECIFIED \| \|  \| \| 284 APLASTIC ANAEMIA \| \| 284.0 CONSTITUTIONAL APLASTIC ANAEMIA \| \| 284.01 Constitutional red blood cell aplasia \| \| 284.09 Other constitutional aplastic anemia \| \| 284.1 Pancytopenia \| \| 284.11 Antineoplastic chemotherapy induced pancytopenia \| \| 284.12 Other drug-induced pancytopenia \| \| 284.19 Other pancytopenia \| \| 284.2 Myelophthisis \| \| 284.8 OTHER \| \| 284.81 Red cell aplasia (acquired)(adult)(with thymoma) \| \| 284.89 Other specified aplastic anemias \| \| 284.9 UNSPECIFIED \| \|  \| \| 285 OTHER AND UNSPECIFIED ANAEMIAS \| \| 285.0 SIDEROBLASTIC ANAEMIA \| \| 285.1 ACUTE POSTHAEMORRHAGIC ANAEMIA \| \| 285.2 Anemia of chronic illness \| \| 285.21 Anemia in chronic kidney disease \| \| 285.22 Anemia in neoplastic disease \| \| 285.29 Anemia of other chronic disease \| \| 285.3 Antineoplastic chemotherapy induced anemia \| \| 285.8 OTHER SPECIFIED ANAEMIAS \| \| 285.9 ANAEMIA, UNSPECIFIED \| \|  \| \| SPECIAL SCREENING FOR DISORDERS OF BLOOD AND BLOOD-FORMING ORGANS \| \| V78.0 IRON DEFICIENCY ANAEMIA \| \| V78.1 OTHER AND UNSPECIFIED DEFICIENCY ANAEMIA \| \| V78.2 SICKLE-CELL DISEASE OR TRAIT \| \|  \| \| **RBC count (10^12/L)** \| \| Female Normal: 3.50–5.00 × 10^12/L \| \| Male Normal: 4.00–5.50 × 10^12^/L \| \|  \| \| **hemoglobin (g/dL)** \| \| MEN 13.6 OR LOWER (g/dL) \| \| WOMEN 11.9 or lower (g/dL) \| \|  \| \| **hematocrit (%)** \| \| MEN <40% \| \| WOMEN <35% \| \|  \| \| **ferritin in serum or plasma (ng/mL)** \| \| LESS THAN 15 UG/L \| \|  \| \| **Vitamin B12 (cobalamin) (moles/volume) in serum or plasma (pmol/L )** \| \| Normal: greater than 150 pmol/L \| \|  \| \| Anaemia \| \| Anemia \| \| B12 deficiency \| \| B12 injection \| \| B12 low \| \| B12 shot \| \| Ferritin low \| \| Hgb low \| \| Iron deficiency \| \| Iron deficiency anemia \| \| Iron deficiency anaemia \| \| Iron low \| \| Low B12 \| \| Low ferritin \| \| Low hgb \| \| Low hemoglobin \| \| Low iron \| \| Vitamin B12 deficiency \| \| Vitamin B12 injection \| \| Vit b12 injection \| \| Vitamin B12 replacement therapy \| \| Vit b12 shot \| \|  \| \| \| --- \| --- \| --- \| --- \| --- \| --- \| --- \| --- \| --- \| --- \| --- \| --- \| --- \| --- \| --- \| --- \| --- \| --- \| --- \| --- \| --- \| --- \| --- \| --- \| --- \| --- \| --- \| --- \| --- \| --- \| --- \| --- \| --- \| --- \| --- \| --- \| --- \| --- \| --- \| --- \| --- \| --- \| --- \| --- \| --- \| --- \| --- \| --- \| --- \| --- \| --- \| --- \| --- \| --- \| --- \| --- \| --- \| --- \| --- \| --- \| --- \| --- \| --- \| --- \| --- \| --- \| --- \| --- \| --- \| --- \| --- \| --- \| --- \| --- \| --- \| --- \| --- \| --- \| --- \| --- \| --- \| --- \| --- \| --- \| --- \| --- \| --- \| --- \| --- \| --- \| --- \| --- \| --- \| --- \| --- \| --- \| --- \| --- \| --- \| --- \| --- \| --- \| --- \| --- \| --- \| --- \| --- \| --- \| --- \| --- \| --- \| --- \| --- \| --- \| --- \| --- \| --- \| --- \| --- \| --- \| --- \| --- \| --- \| --- \| --- \| --- \| --- \| --- \| --- \| --- \| --- \| --- \| --- \| --- \| --- \| | \| **ICD9** \| \| --- \| \| **ICD9** \| \| **ICD9** \| \|  \| \| **ICD9/TERMWORKS** \| \| **ICD9-CM** \| \| **ICD9-CM** \| \| **ICD9-CM** \| \| **ICD9-CM/TERMWORKS** \| \|  \| \|  \| \| **ICD9/TERMWORKS** \| \| **ICD9-CM/TERMWORKS** \| \| **ICD9-CM/TERMWORKS** \| \| **ICD9-CM/TERMWORKS** \| \| **ICD9-CM/TERMWORKS** \| \|  \| \| **ICD9/TERMWORKS** \| \| **ICD9/TERMWORKS** \| \| **ICD9/TERMWORKS** \| \| **ICD9/TERMWORKS** \| \| **ICD9/TERMWORKS** \| \| **ICD9/TERMWORKS** \| \| **ICD9/TERMWORKS** \| \| **ICD9/TERMWORKS** \| \|  \| \| **ICD9/TERMWORKS** \| \| **ICD9** \| \| **ICD9** \| \| **ICD9/TERMWORKS** \| \| **ICD9/TERMWORKS** \| \| **ICD9** \| \| **ICD9-CM/TERMWORKS** \| \| **ICD9-CM/TERMWORKS** \| \| **ICD9-CM/TERMWORKS** \| \| **ICD9-CM/TERMWORKS** \| \| **ICD9-CM/TERMWORKS** \| \| **ICD9-CM/TERMWORKS** \| \| **ICD9-CM/TERMWORKS** \| \| **ICD9-CM/TERMWORKS** \| \| **ICD9-CM/TERMWORKS** \| \| **ICD9/TERMWORKS** \| \| **ICD9/TERMWORKS** \| \| **ICD9-CM/TERMWORKS** \| \| **ICD9-CM** \| \| **ICD9-CM** \| \| **ICD9-CM/TERMWORKS** \| \| **ICD9-CM/TERMWORKS** \| \| **ICD9-CM/TERMWORKS** \| \| **ICD9-CM/TERMWORKS** \| \| **ICD9** \| \| **ICD9/TERMWORKS** \| \| **ICD9/TERMWORKS** \| \|  \| \| **ICD9/TERMWORKS** \| \| **ICD9/TERMWORKS** \| \| **ICD9/TERMWORKS** \| \| **ICD9-CM/TERMWORKS** \| \| **ICD9-CM** \| \| **ICD9-CM/TERMWORKS** \| \| **ICD9** \| \| **ICD9/TERMWORKS** \| \|  \| \| **ICD9/TERMWORKS** \| \| **ICD9/TERMWORKS** \| \| **ICD9-CM** \| \| **ICD9-CM/TERMWORKS** \| \| **ICD9-CM** \| \| **ICD9-CM** \| \| **ICD9-CM** \| \| **ICD9-CM** \| \| **ICD9-CM** \| \| **ICD9/TERMWORKS** \| \| **ICD9-CM** \| \| **ICD9-CM/TERMWORKS** \| \| **ICD9/TERMWORKS** \| \|  \| \| **ICD9/TERMWORKS** \| \| **ICD9/TERMWORKS** \| \| **ICD9/TERMWORKS** \| \| **ICD9-CM/TERMWORKS** \| \| **ICD9-CM/TERMWORKS** \| \| **ICD9-CM/TERMWORKS** \| \| **ICD9-CM/TERMWORKS** \| \| **ICD9-CM/TERMWORKS** \| \| **ICD9/TERMWORKS** \| \| **ICD9/TERMWORKS** \| \|  \| \|  \| \| **ICD9/TERMWORKS** \| \| **ICD9/TERMWORKS** \| \| **TERMWORKS** \| \|  \| \| **LOINC 789-8** \| \|  \| \|  \| \|  \| \| **LOINC 718-7** \| \|  \| \|  \| \|  \| \| **LOINC 20570-8** \| \|  \| \|  \| \|  \| \| **LOINC 2276-4** \| \|  \| \|  \| \| **LOINC 14685-2** \| \|  \| \|  \| \| **CPCSSN Free Text** \| \| **CPCSSN Free Text** \| \| **CPCSSN Free Text** \| \| **CPCSSN Free Text** \| \| **CPCSSN Free Text** \| \| **CPCSSN Free Text** \| \| **CPCSSN Free Text** \| \| **CPCSSN Free Text** \| \| **CPCSSN Free Text** \| \| **CPCSSN Free Text** \| \| **CPCSSN Free Text** \| \| **CPCSSN Free Text** \| \| **CPCSSN Free Text** \| \| **CPCSSN Free Text** \| \| **CPCSSN Free Text** \| \| **CPCSSN Free Text** \| \| **CPCSSN Free Text** \| \| **CPCSSN Free Text** \| \| **CPCSSN Free Text** \| \| **CPCSSN Free Text** \| \| **CPCSSN Free Text** \| \| **CPCSSN Free Text** \| \|  \| |

Termworks search terms: anemia, anaemia, iron, sickle-cell, ferritin, b12, thalassaemia, folate

## Arthritis

| **Codes and Terms** | **Source(s)** |
| --- | --- |
| \| \| 696.0 PSORIATIC ARTHROPATHY \| \| --- \| \|  \| \| 274 GOUT \| \| 274.0 GOUTY ARTHROPATHY \| \| 274.00 Gouty arthropathy, unspecified \| \| 274.01 Acute gouty arthropathy \| \| 274.02 Chronic gouty arthropathy without mention of tophus (tophi) \| \| 274.03 Chronic gouty arthropathy with tophus (tophi) \| \| 274.1 GOUTY NEPHROPATHY \| \| 274.10 Gouty nephropathy, unspecified \| \| 274.11 Uric acid nephrolithiasis \| \| 274.19 Other gouty nephropathy \| \| 274.8 GOUT WITH OTHER MANIFESTATIONS \| \| 274.81 Gouty tophi of ear \| \| 274.82 Gouty tophi of other sites, except ear \| \| 274.89 Gout with other specified manifestations \| \| 274.9 UNSPECIFIED \| \|  \| \|  \| \| DISEASES OF THE MUSKULOSKELETAL SYSTEM AND CONNECTIVE TISSUE \| \| 712 CRYSTAL ARTHROPATHIES \| \| 712.0 GOUTY ARTHRITIS \| \|  \| \| 713 ARTHROPATHY ASSOCIATED WITH OTHER DISORDERS CLASSIFIED ELSEWHERE \| \| 713.0 Arthropathy associated with other endocrine and metabolic disorders \| \| 713.1 Arthropathy associated with gastrointestinal conditions other than infections \| \| 713.2 Arthropathy associated with hematological disorders \| \| 713.3 Arthropathy associated with dermatological disorders \| \| 713.4 Arthropathy associated with respiratory disorders \| \| 713.5 Arthropathy associated with neurological disorders \| \| 713.6 Arthropathy associated with hypersensitivity reaction \| \| 713.7 Other general diseases with articular involvement \| \| 713.8 Arthropathy associated with other conditions classifiable elsewhere \| \|  \| \| 714 RHEUMATOID ARTHRITIS AND OTHER INFLAMMATORY POLYARTHROPATHIES \| \| 714.0 RHEUMATOID ARTHRITIS \| \| 714.1 FELTY'S SYNDROME \| \| 714.2 OTHER RHEUMATOID ARTHRITIS WITH VISCERAL OR SYSTEMIC INVOLVEMENT \| \|  \| \| 715 OSTEOARTHROSIS AND ALLIED DISORDERS \| \| 715.0 GENERALIZED \| \| 715.00 Osteoarthrosis, generalized, site unspecified \| \| 715.04 Osteoarthrosis, generalized, hand \| \| 715.09 Osteoarthrosis, generalized, multiple sites \| \| 715.1 LOCALIZED, PRIMARY \| \| 715.10 Osteoarthrosis, localized, primary, site unspecified \| \| 715.11 Osteoarthrosis, localized, primary, shoulder region \| \| 715.12 Osteoarthrosis, localized, primary, upper arm \| \| 715.13 Osteoarthrosis, localized, primary, forearm \| \| 715.14 Osteoarthrosis, localized, primary, hand \| \| 715.15 Osteoarthrosis, localized, primary, pelvic region and thigh \| \| 715.16 Osteoarthrosis, localized, primary, lower leg \| \| 715.17 Osteoarthrosis, localized, primary, ankle and foot \| \| 715.18 Osteoarthrosis, localized, primary, other specified sites \| \| 715.2 LOCALIZED, SECONDARY \| \| 715.20 Osteoarthrosis, localized, secondary, site unspecified \| \| 715.21 Osteoarthrosis, localized, secondary, shoulder region \| \| 715.22 Osteoarthrosis, localized, secondary, upper arm \| \| 715.23 Osteoarthrosis, localized, secondary, forearm \| \| 715.24 Osteoarthrosis, localized, secondary, hand \| \| 715.25 Osteoarthrosis, localized, secondary, pelvic region and thigh \| \| 715.26 Osteoarthrosis, localized, secondary, lower leg \| \| 715.27 Osteoarthrosis, localized, secondary, ankle and foot \| \| 715.28 Osteoarthrosis, localized, secondary, other specified sites \| \| 715.3 LOCALIZED, NOT SPECIFIED WHETHER PRIMARY OR SECONDARY \| \| 715.30 Osteoarthrosis, localized, not specified whether primary or secondary, site unspecified \| \| 715.31 Osteoarthrosis, localized, not specified whether primary or secondary, shoulder region \| \| 715.32 Osteoarthrosis, localized, not specified whether primary or secondary, upper arm \| \| 715.33 Osteoarthrosis, localized, not specified whether primary or secondary, forearm \| \| 715.34 Osteoarthrosis, localized, not specified whether primary or secondary, hand \| \| 715.35 Osteoarthrosis, localized, not specified whether primary or secondary, pelvic region and thigh \| \| 715.36 Osteoarthrosis, localized, not specified whether primary or secondary, lower leg \| \| 715.37 Osteoarthrosis, localized, not specified whether primary or secondary, ankle and foot \| \| 715.38 Osteoarthrosis, localized, not specified whether primary or secondary, other specified sites \| \| 715.8 AS GENERALIZED INVOLVING OR WITH MENTION OF MORE THAN ONE SITE BUT NOT SPECIFIED \| \| 715.80 Osteoarthrosis involving, or with mention of more than one site, but not specified as generalized, site unspecified \| \| 715.89 Osteoarthrosis involving, or with mention of more than one site, but not specified as generalized, multiple sites \| \| 715.9 UNSPECIFIED WHETHER GENERALIZED OR LOCALIZED \| \| 715.90 Osteoarthrosis, unspecified whether generalized or localized, site unspecified \| \| 715.91 Osteoarthrosis, unspecified whether generalized or localized, shoulder region \| \| 715.92 Osteoarthrosis, unspecified whether generalized or localized, upper arm \| \| 715.93 Osteoarthrosis, unspecified whether generalized or localized, forearm \| \| 715.94 Osteoarthrosis, unspecified whether generalized or localized, hand \| \| 715.95 Osteoarthrosis, unspecified whether generalized or localized, pelvic region and thigh \| \| 715.96 Osteoarthrosis, unspecified whether generalized or localized, lower leg \| \| 715.97 Osteoarthrosis, unspecified whether generalized or localized, ankle and foot \| \| 715.98 Osteoarthrosis, unspecified whether generalized or localized, other specified sites \| \|  \| \| 716 OTHER AND UNSPECIFIED ARTHROPATHIES \| \| 716.2 ALLERGIC ARTHRITIS \| \| 716.20 Allergic arthritis, site unspecified \| \| 716.21 Allergic arthritis, shoulder region \| \| 716.22 Allergic arthritis, upper arm \| \| 716.23 Allergic arthritis, forearm \| \| 716.24 Allergic arthritis, hand \| \| 716.25 Allergic arthritis, pelvic region and thigh \| \| 716.26 Allergic arthritis, lower leg \| \| 716.27 Allergic arthritis, ankle and foot \| \| 716.28 Allergic arthritis, other specified sites \| \| 716.29 Allergic arthritis, multiple sites \| \| 716.5 UNSPECIFIED POLYARTHROPATHY OR POLYARTHRITIS \| \| 716.50 Unspecified polyarthropathy or polyarthritis, site unspecified \| \| 716.51 Unspecified polyarthropathy or polyarthritis, shoulder region \| \| 716.52 Unspecified polyarthropathy or polyarthritis, upper arm \| \| 716.53 Unspecified polyarthropathy or polyarthritis, forearm \| \| 716.54 Unspecified polyarthropathy or polyarthritis, hand \| \| 716.55 Unspecified polyarthropathy or polyarthritis, pelvic region and thigh \| \| 716.56 Unspecified polyarthropathy or polyarthritis, lower leg \| \| 716.57 Unspecified polyarthropathy or polyarthritis, ankle and foot \| \| 716.58 Unspecified polyarthropathy or polyarthritis, other specified sites \| \| 716.59 Unspecified polyarthropathy or polyarthritis, multiple sites \| \| 716.6 UNSPECIFIED MONOARTHRITIS \| \| 716.60 Unspecified monoarthritis, site unspecified \| \| 716.61 Unspecified monoarthritis, shoulder region \| \| 716.62 Unspecified monoarthritis, upper arm \| \| 716.63 Unspecified monoarthritis, forearm \| \| 716.64 Unspecified monoarthritis, hand \| \| 716.65 Unspecified monoarthritis, pelvic region and thigh \| \| 716.66 Unspecified monoarthritis, lower leg \| \| 716.67 Unspecified monoarthritis, ankle and foot \| \| 716.68 Unspecified monoarthritis, other specified sites \| \| 716.8 OTHER SPECIFIED ARTHROPATHY \| \| 716.80 Other specified arthropathy, site unspecified \| \| 716.81 Other specified arthropathy, shoulder region \| \| 716.82 Other specified arthropathy, upper arm \| \| 716.83 Other specified arthropathy, forearm \| \| 716.84 Other specified arthropathy, hand \| \| 716.85 Other specified arthropathy, pelvic region and thigh \| \| 716.86 Other specified arthropathy, lower leg \| \| 716.87 Other specified arthropathy, ankle and foot \| \| 716.88 Other specified arthropathy, other specified sites \| \| 716.89 Other specified arthropathy, multiple sites \| \| 716.9 UNSPECIFIED \| \| 716.90 Arthropathy, unspecified, site unspecified \| \| 716.91 Arthropathy, unspecified, shoulder region \| \| 716.92 Arthropathy, unspecified, upper arm \| \| 716.93 Arthropathy, unspecified, forearm \| \| 716.94 Arthropathy, unspecified, hand \| \| 716.95 Arthropathy, unspecified, pelvic region and thigh \| \| 716.96 Arthropathy, unspecified, lower leg \| \| 716.97 Arthropathy, unspecified, ankle and foot \| \| 716.98 Arthropathy, unspecified, other specified sites \| \| 716.99 Arthropathy, unspecified, multiple sites \| \|  \| \| 718.5 ANKYLOSIS OF JOINT \| \| 718.50 Ankylosis of joint, site unspecified \| \| 718.51 Ankylosis of joint, shoulder region \| \| 718.52 Ankylosis of joint, upper arm \| \| 718.53 Ankylosis of joint, forearm \| \| 718.54 Ankylosis of joint, hand \| \| 718.55 Ankylosis of joint, pelvic region and thigh \| \| 718.56 Ankylosis of joint, lower leg \| \| 718.57 Ankylosis of joint, ankle and foot \| \| 718.58 Ankylosis of joint, other specified sites \| \| 718.59 Ankylosis of joint, multiple sites \| \|  \| \| 719 OTHER AND UNSPECIFIED DISORDER OF JOINT \| \| 719.0 EFFUSION OF JOINT \| \| 719.00 Effusion of joint, site unspecified \| \| 719.01 Effusion of joint, shoulder region \| \| 719.02 Effusion of joint, upper arm \| \| 719.03 Effusion of joint, forearm \| \| 719.04 Effusion of joint, hand \| \| 719.05 Effusion of joint, pelvic region and thigh \| \| 719.06 Effusion of joint, lower leg \| \| 719.07 Effusion of joint, ankle and foot \| \| 719.08 Effusion of joint, other specified sites \| \| 719.09 Effusion of joint, multiple sites \| \| 719.3 PALINDROMIC RHEUMATISM \| \| 719.30 Palindromic rheumatism, site unspecified \| \| 719.31 Palindromic rheumatism, shoulder region \| \| 719.32 Palindromic rheumatism, upper arm \| \| 719.33 Palindromic rheumatism, forearm \| \| 719.34 Palindromic rheumatism, hand \| \| 719.35 Palindromic rheumatism, pelvic region and thigh \| \| 719.36 Palindromic rheumatism, lower leg \| \| 719.37 Palindromic rheumatism, ankle and foot \| \| 719.38 Palindromic rheumatism, other specified sites \| \| 719.39 Palindromic rheumatism, multiple sites \| \| 719.4 PAIN IN JOINT \| \| 719.40 Pain in joint, site unspecified \| \| 719.41 Pain in joint, shoulder region \| \| 719.42 Pain in joint, upper arm \| \| 719.43 Pain in joint, forearm \| \| 719.44 Pain in joint, hand \| \| 719.45 Pain in joint, pelvic region and thigh \| \| 719.46 Pain in joint, lower leg \| \| 719.47 Pain in joint, ankle and foot \| \| 719.48 Pain in joint, other specified sites \| \| 719.49 Pain in joint, multiple sites \| \| 719.5 STIFFNESS OF JOINT, NOT ELSEWHERE CLASSIFIED \| \| 719.50 Stiffness of joint, not elsewhere classified, site unspecified \| \| 719.51 Stiffness of joint, not elsewhere classified, shoulder region \| \| 719.52 Stiffness of joint, not elsewhere classified, upper arm \| \| 719.53 Stiffness of joint, not elsewhere classified, forearm \| \| 719.54 Stiffness of joint, not elsewhere classified, hand \| \| 719.55 Stiffness of joint, not elsewhere classified, pelvic region and thigh \| \| 719.56 Stiffness of joint, not elsewhere classified, lower leg \| \| 719.57 Stiffness of joint, not elsewhere classified, ankle and foot \| \| 719.58 Stiffness of joint, not elsewhere classified, other specified sites \| \| 719.59 Stiffness of joint, not elsewhere classified, multiple sites \| \|  \| \| DORSOPATHIES \| \| 720 ANKYLOSING SPONDYLITIS AND OTHER INFLAMMATORY SPONDYLOPATHIES \| \| 720.0 ANKYLOSING SPONDYLITIS \| \| 720.1 SPINAL ENTHESOPATHY \| \| 720.2 SACROILIITIS, NOT ELSEWHERE CLASSIFIED \| \| 720.8 OTHER INFLAMMATORY SPONDYLOPATHIES \| \| 720.81 Inflammatory spondylopathies in diseases classified elsewhere \| \| 720.89 Other inflammatory spondylopathies \| \| 720.9 UNSPECIFIED INFLAMMATORY SPONDYLOPATHY \| \|  \| \| 721 SPONDYLOSIS AND ALLIED DISORDERS \| \| 721.0 CERVICAL SPONDYLOSIS WITHOUT MYELOPATHY \| \| 721.1 CERVICAL SPONDYLOSIS WITH MYELOPATHY \| \| 721.2 THORACIC SPONDYLOSIS WITHOUT MYELOPATHY \| \| 721.3 LUMBOSACRAL SPONDYLOSIS WITHOUT MYELOPATHY \| \| 721.4 THORACIC OR LUMBAR SPONDYLOSIS WITH MYELOPATHY \| \| 721.41 Spondylosis with myelopathy, thoracic region \| \| 721.42 Spondylosis with myelopathy, lumbar region \| \| 721.5 KISSING SPINE \| \| 721.6 ANKYLOSING VERTEBRAL HYPEROSTOSIS \| \| 721.7 TRAUMATIC SPONDYLOPATHY \| \| 721.8 OTHER \| \| 721.9 SPONDYLOSIS OF UNSPECIFIED SITE \| \| 721.90 Spondylosis of unspecified site, without mention of myelopathy \| \| 721.91 Spondylosis of unspecified site, with myelopathy \| \|  \| \| V13.4 ARTHRITIS (Under "personal history of other diseases") \| \| V77.5 GOUT (Under "special screening for endocrine, nutritional, metabolic, and immunity disorders") \| \| V82.1 RHEUMATOID ARTHRITIS (Under special screening for other conditions" \| \| V54.81 Aftercare following joint replacement \| \|  \| \| V43.64 Hip joint replacement \| \| V43.65 Knee joint replacement \| \|  \| \| **Rheumatoid Factor in Serum or Plasma** \| \| > 24 U/mL (normal: <24 units/mL) \| \| Presence \| \|  \| \| **Anti-CCP antibodies (Cyclic Citrullinated Peptide IgG Ab in Serum or Plasma)** \| \| > 20 U/mL (normal: <20 units/mL) \| \| Presence \| \|  \| \| Arthritis \| \| Arthralgia \| \| Arthritis chronic \| \| Arthritis gout \| \| Arthritis psoriatic \| \| Arthritis rheumatoid \| \| arthritis septic \| \| arthritis inflammatory \| \| Arthropathy \| \| Arthritic \| \| Gout \| \| Gouty \| \| Gouty arthropathy \| \| Hip replacement \| \| Joint pain \| \| Knee replacement \| \| Osteoarthritis \| \| Pain in joint \| \| Polyarthralgia \| \| Polyarthritis \| \| Pseudogout \| \| Rheumatoid arthritis \| \| Spondylopathy \| \|  \| \| \| --- \| --- \| --- \| --- \| --- \| --- \| --- \| --- \| --- \| --- \| --- \| --- \| --- \| --- \| --- \| --- \| --- \| --- \| --- \| --- \| --- \| --- \| --- \| --- \| --- \| --- \| --- \| --- \| --- \| --- \| --- \| --- \| --- \| --- \| --- \| --- \| --- \| --- \| --- \| --- \| --- \| --- \| --- \| --- \| --- \| --- \| --- \| --- \| --- \| --- \| --- \| --- \| --- \| --- \| --- \| --- \| --- \| --- \| --- \| --- \| --- \| --- \| --- \| --- \| --- \| --- \| --- \| --- \| --- \| --- \| --- \| --- \| --- \| --- \| --- \| --- \| --- \| --- \| --- \| --- \| --- \| --- \| --- \| --- \| --- \| --- \| --- \| --- \| --- \| --- \| --- \| --- \| --- \| --- \| --- \| --- \| --- \| --- \| --- \| --- \| --- \| --- \| --- \| --- \| --- \| --- \| --- \| --- \| --- \| --- \| --- \| --- \| --- \| --- \| --- \| --- \| --- \| --- \| --- \| --- \| --- \| --- \| --- \| --- \| --- \| --- \| --- \| --- \| --- \| --- \| --- \| --- \| --- \| --- \| --- \| --- \| --- \| --- \| --- \| --- \| --- \| --- \| --- \| --- \| --- \| --- \| --- \| --- \| --- \| --- \| --- \| --- \| --- \| --- \| --- \| --- \| --- \| --- \| --- \| --- \| --- \| --- \| --- \| --- \| --- \| --- \| --- \| --- \| --- \| --- \| --- \| --- \| --- \| --- \| --- \| --- \| --- \| --- \| --- \| --- \| --- \| --- \| --- \| --- \| --- \| --- \| --- \| --- \| --- \| --- \| --- \| --- \| --- \| --- \| --- \| --- \| --- \| --- \| --- \| --- \| --- \| --- \| --- \| --- \| --- \| --- \| --- \| --- \| --- \| --- \| --- \| --- \| --- \| --- \| --- \| --- \| --- \| --- \| --- \| --- \| --- \| --- \| --- \| --- \| --- \| --- \| --- \| --- \| --- \| --- \| --- \| --- \| --- \| --- \| --- \| --- \| --- \| --- \| --- \| --- \| --- \| --- \| --- \| --- \| --- \| --- \| --- \| --- \| --- \| --- \| --- \| --- \| --- \| --- \| --- \| --- \| --- \| --- \| --- \| --- \| --- \| --- \| --- \| --- \| --- \| --- \| --- \| --- \| --- \| | \| **ICD9/TERMWORKS** \| \| --- \| \|  \| \| **ICD9/TERMWORKS** \| \| **ICD9/TERMWORKS** \| \| **ICD9-CM/TERMWORKS** \| \| **ICD9-CM/TERMWORKS** \| \| **ICD9-CM/TERMWORKS** \| \| **ICD9-CM/TERMWORKS** \| \| **ICD9/TERMWORKS** \| \| **ICD9-CM/TERMWORKS** \| \| **ICD9-CM/TERMWORKS** \| \| **ICD9/TERMWORKS** \| \| **ICD9-CM/TERMWORKS** \| \| **ICD9-CM/TERMWORKS** \| \| **ICD9-CM/TERMWORKS** \| \| **ICD9/TERMWORKS** \| \|  \| \|  \| \|  \| \| **ICD9** \| \| **ICD9/TERMWORKS** \| \|  \| \| **ICD9/TERMWORKS** \| \| **ICD9-CM/TERMWORKS** \| \| **ICD9-CM/TERMWORKS** \| \| **ICD9-CM/TERMWORKS** \| \| **ICD9-CM/TERMWORKS** \| \| **ICD9-CM/TERMWORKS** \| \| **ICD9-CM/TERMWORKS** \| \| **ICD9-CM/TERMWORKS** \| \| **ICD9-CM** \| \| **ICD9-CM/TERMWORKS** \| \|  \| \| **ICD9/TERMWORKS** \| \| **ICD9/TERMWORKS** \| \| **ICD9** \| \| **ICD9/TERMWORKS** \| \|  \| \| **ICD9/TERMWORKS** \| \| **ICD9** \| \| **ICD9-CM** \| \| **ICD9-CM** \| \| **ICD9-CM** \| \| **ICD9** \| \| **ICD9-CM** \| \| **ICD9-CM** \| \| **ICD9-CM** \| \| **ICD9-CM** \| \| **ICD9-CM** \| \| **ICD9-CM** \| \| **ICD9-CM** \| \| **ICD9-CM** \| \| **ICD9-CM** \| \| **ICD9** \| \| **ICD9-CM** \| \| **ICD9-CM** \| \| **ICD9-CM** \| \| **ICD9-CM** \| \| **ICD9-CM** \| \| **ICD9-CM** \| \| **ICD9-CM** \| \| **ICD9-CM** \| \| **ICD9-CM** \| \| **ICD9** \| \| **ICD9-CM** \| \| **ICD9-CM** \| \| **ICD9-CM** \| \| **ICD9-CM** \| \| **ICD9-CM** \| \| **ICD9-CM** \| \| **ICD9-CM** \| \| **ICD9-CM** \| \| **ICD9-CM** \| \| **ICD9** \| \| **ICD9-CM** \| \| **ICD9-CM** \| \| **ICD9** \| \| **ICD9-CM** \| \| **ICD9-CM** \| \| **ICD9-CM** \| \| **ICD9-CM** \| \| **ICD9-CM** \| \| **ICD9-CM** \| \| **ICD9-CM** \| \| **ICD9-CM** \| \| **ICD9-CM** \| \|  \| \|  \| \| **ICD9/TERMWORKS** \| \| **ICD9/TERMWORKS** \| \| **ICD9-CM/TERMWORKS** \| \| **ICD9-CM/TERMWORKS** \| \| **ICD9-CM/TERMWORKS** \| \| **ICD9-CM/TERMWORKS** \| \| **ICD9-CM/TERMWORKS** \| \| **ICD9-CM/TERMWORKS** \| \| **ICD9-CM/TERMWORKS** \| \| **ICD9-CM/TERMWORKS** \| \| **ICD9-CM/TERMWORKS** \| \| **ICD9-CM/TERMWORKS** \| \| **ICD9/TERMWORKS** \| \| **ICD9-CM** \| \| **ICD9-CM** \| \| **ICD9-CM** \| \| **ICD9-CM** \| \| **ICD9-CM** \| \| **ICD9-CM** \| \| **ICD9-CM** \| \| **ICD9-CM** \| \| **ICD9-CM** \| \| **ICD9-CM** \| \| **ICD9/TERMWORKS** \| \| **ICD9-CM** \| \| **ICD9-CM** \| \| **ICD9-CM** \| \| **ICD9-CM** \| \| **ICD9-CM** \| \| **ICD9-CM** \| \| **ICD9-CM** \| \| **ICD9-CM** \| \| **ICD9-CM** \| \| **ICD9/TERMWORKS** \| \| **ICD9-CM/TERMWORKS** \| \| **ICD9-CM/TERMWORKS** \| \| **ICD9-CM/TERMWORKS** \| \| **ICD9-CM/TERMWORKS** \| \| **ICD9-CM/TERMWORKS** \| \| **ICD9-CM/TERMWORKS** \| \| **ICD9-CM/TERMWORKS** \| \| **ICD9-CM/TERMWORKS** \| \| **ICD9-CM/TERMWORKS** \| \| **ICD9-CM/TERMWORKS** \| \| **ICD9/TERMWORKS** \| \| **ICD9-CM/TERMWORKS** \| \| **ICD9-CM/TERMWORKS** \| \| **ICD9-CM/TERMWORKS** \| \| **ICD9-CM/TERMWORKS** \| \| **ICD9-CM/TERMWORKS** \| \| **ICD9-CM/TERMWORKS** \| \| **ICD9-CM/TERMWORKS** \| \| **ICD9-CM/TERMWORKS** \| \| **ICD9-CM/TERMWORKS** \| \| **ICD9-CM/TERMWORKS** \| \|  \| \| **ICD9** \| \| **ICD9-CM** \| \| **ICD9-CM** \| \| **ICD9-CM** \| \| **ICD9-CM** \| \| **ICD9-CM** \| \| **ICD9-CM** \| \| **ICD9-CM** \| \| **ICD9-CM** \| \| **ICD9-CM** \| \|  \| \|  \| \| **ICD9** \| \| **ICD9** \| \| **ICD9-CM** \| \| **ICD9-CM** \| \| **ICD9-CM** \| \| **ICD9-CM** \| \| **ICD9-CM** \| \| **ICD9-CM** \| \| **ICD9-CM** \| \| **ICD9-CM** \| \| **ICD9-CM** \| \| **ICD9-CM** \| \| **ICD9/TERMWORKS** \| \| **ICD9-CM** \| \| **ICD9-CM** \| \| **ICD9-CM** \| \| **ICD9-CM** \| \| **ICD9-CM** \| \| **ICD9-CM** \| \| **ICD9-CM** \| \| **ICD9-CM** \| \| **ICD9-CM** \| \| **ICD9-CM** \| \| **ICD9** \| \| **ICD9-CM** \| \| **ICD9-CM** \| \| **ICD9-CM** \| \| **ICD9-CM** \| \| **ICD9-CM** \| \| **ICD9-CM** \| \| **ICD9-CM** \| \| **ICD9-CM** \| \| **ICD9-CM** \| \| **ICD9-CM** \| \| **ICD9** \| \| **ICD9-CM** \| \| **ICD9-CM** \| \| **ICD9-CM** \| \| **ICD9-CM** \| \| **ICD9-CM** \| \| **ICD9-CM** \| \| **ICD9-CM** \| \| **ICD9-CM** \| \| **ICD9-CM** \| \| **ICD9-CM** \| \|  \| \|  \| \| **ICD9** \| \| **ICD9** \| \| **ICD9** \| \| **ICD9** \| \| **ICD9** \| \| **ICD9-CM** \| \| **ICD9-CM** \| \| **ICD9** \| \|  \| \| **ICD9/TERMWORKS** \| \| **ICD9/TERMWORKS** \| \| **ICD9/TERMWORKS** \| \| **ICD9/TERMWORKS** \| \| **ICD9/TERMWORKS** \| \| **ICD9/TERMWORKS** \| \| **ICD9-CM/TERMWORKS** \| \| **ICD9-CM/TERMWORKS** \| \| **ICD9** \| \| **ICD9** \| \| **ICD9** \| \| **ICD9** \| \| **ICD9/TERMWORKS** \| \| **ICD9-CM/TERMWORKS** \| \| **ICD9-CM/TERMWORKS** \| \|  \| \| **ICD9/TERMWORKS** \| \| **ICD9/TERMWORKS** \| \| **ICD9/TERMWORKS** \| \| **ICD9-CM/TERMWORKS** \| \|  \| \| **ICD9-CM/TERMWORKS** \| \| **ICD9-CM/TERMWORKS** \| \|  \| \|  \| \| **LOINC 11572-5** \| \| **LOINC 33910-1** \| \|  \| \|  \| \| **LOINC 33935-8** \| \| **LOINC 42898-7** \| \|  \| \| **CPCSSN Free Text** \| \| **CPCSSN Free Text** \| \| **CPCSSN Free Text** \| \| **CPCSSN Free Text** \| \| **CPCSSN Free Text** \| \| **CPCSSN Free Text** \| \| **CPCSSN Free Text** \| \| **CPCSSN Free Text** \| \| **CPCSSN Free Text** \| \| **CPCSSN Free Text** \| \| **CPCSSN Free Text** \| \| **CPCSSN Free Text** \| \| **CPCSSN Free Text** \| \| **CPCSSN Free Text** \| \| **CPCSSN Free Text** \| \| **CPCSSN Free Text** \| \| **CPCSSN Free Text** \| \| **CPCSSN Free Text** \| \| **CPCSSN Free Text** \| \| **CPCSSN Free Text** \| \| **CPCSSN Free Text** \| \| **CPCSSN Free Text** \| \| **CPCSSN Free Text** \| \|  \| |

Termworks search terms: arthritis, osteoarthritis, rheumatoid arthritis, joint replacement, gout, gouty, spondylosis, joint Xray abnormal, arthropathy

## Atrial fibrillation

| **Codes and Terms** | **Source(s)** |
| --- | --- |
| \| \| 427 Cardiac dysrhythmias - AF seems to be coded as this in CPCSSN \| \| --- \| \| 427.3 ATRIAL FIBRILLATION AND FLUTTER \| \| 427.31 Atrial fibrillation \| \| 427.32 Atrial flutter \| \|  \| \| 794.31 Nonspecific abnormal electrocardiogram [ECG] [EKG] \| \|  \| \| A fib \| \| Abnormal ECG \| \| Abnormal electrocardiogram \| \| AFib \| \| Arrythmia \| \| Atrial fib \| \| Atrial Fibrillation \| \| Atrial flutter \| \| Cardiac dysrhythmias \| \| ECG abnormal \| \| EKG abnormal \| \| Flutter \| \| Heart flutter \| \| Heart fluttering \| \| Heart rhythm irregular \| \| Irregular heartbeat \| \| PAF \| \| Paroxysmal atrial fibrillation \| \|  \| \| \| --- \| --- \| --- \| --- \| --- \| --- \| --- \| --- \| --- \| --- \| --- \| --- \| --- \| --- \| --- \| --- \| --- \| --- \| --- \| --- \| --- \| --- \| --- \| --- \| --- \| --- \| --- \| | \| **ICD9-CM** \| \| --- \| \| **ICD9/TERMWORKS** \| \| **ICD9-CM/TERMWORKS** \| \| **ICD9-CM/TERMWORKS** \| \|  \| \| **ICD9-CM/TERMWORKS** \| \|  \| \| **CPCSSN Free Text** \| \| **CPCSSN Free Text** \| \| **CPCSSN Free Text** \| \| **CPCSSN Free Text** \| \| **CPCSSN Free Text** \| \| **CPCSSN Free Text** \| \| **CPCSSN Free Text** \| \| **CPCSSN Free Text** \| \| **CPCSSN Free Text** \| \| **CPCSSN Free Text** \| \| **CPCSSN Free Text** \| \| **CPCSSN Free Text** \| \| **CPCSSN Free Text** \| \| **CPCSSN Free Text** \| \| **CPCSSN Free Text** \| \| **CPCSSN Free Text** \| \| **CPCSSN Free Text** \| \| **CPCSSN Free Text** \| |

Termworks search terms: atrial $+ fibrillation, atrial fibrillation, ECG, irregular $+ pulse, flutter

## Cerebrovascular disease

| **Codes and Terms** | **Source(s)** |
| --- | --- |
| \| \| 430 SUBARACHNOID HAEMORRHAGE \| \| --- \| \| 431 INTRACEREBRAL HAEMORRHAGE \| \|  \| \| 432 OTHER AND UNSPECIFIED INTRACRANIAL HAEMORRHAGE \| \| 432.0 NONTRAUMATIC EXTRADURAL HAEMORRHAGE \| \| 432.1 SUBDURAL HAEMORRHAGE \| \| 432.9 UNSPECIFIED INTRACRANIAL HAEMORRHAGE \| \|  \| \| 433 OCCLUSION AND STENOSIS OF PRECEREBRAL ARTERIES \| \| 433.0 BASILAR ARTERY \| \| 433.00 Occlusion and stenosis of basilar artery without mention of cerebral infarction \| \| 433.01 Occlusion and stenosis of basilar artery with cerebral infarction \| \| 433.1 CAROTID ARTERY \| \| 433.10 Occlusion and stenosis of carotid artery without mention of cerebral infarction \| \| 433.11 Occlusion and stenosis of carotid artery with cerebral infarction \| \| 433.2 VERTEBRAL ARTERY \| \| 433.20 Occlusion and stenosis of vertebral artery without mention of cerebral infarction \| \| 433.21 Occlusion and stenosis of vertebral artery with cerebral infarction \| \| 433.3 MULTIPLE AND BILATERAL \| \| 433.30 Occlusion and stenosis of multiple and bilateral precerebral arteries without mention of cerebral infarction \| \| 433.31 Occlusion and stenosis of multiple and bilateral precerebral arteries with cerebral infarction \| \| 433.8 OTHER \| \| 433.80 Occlusion and stenosis of other specified precerebral artery without mention of cerebral infarction \| \| 433.81 Occlusion and stenosis of other specified precerebral artery with cerebral infarction \| \| 433.9 UNSPECIFIED \| \| 433.90 Occlusion and stenosis of unspecified precerebral artery without mention of cerebral infarction \| \| 433.91 Occlusion and stenosis of unspecified precerebral artery with cerebral infarction \| \|  \| \| 434 OCCLUSION OF CEREBRAL ARTERIES \| \| 434.0 CEREBRAL THROMBOSIS \| \| 434.00 Cerebral thrombosis without mention of cerebral infarction \| \| 434.01 Cerebral thrombosis with cerebral infarction \| \| 434.1 CEREBRAL EMBOLISM \| \| 434.10 Cerebral embolism without mention of cerebral infarction \| \| 434.11 Cerebral embolism with cerebral infarction \| \| 434.9 UNSPECIFIED \| \| 434.90 Cerebral artery occlusion, unspecified without mention of cerebral infarction \| \| 434.91 Cerebral artery occlusion, unspecified with cerebral infarction \| \|  \| \| 435 TRANSIENT CEREBRAL ISCHAEMIA \| \| 435.0 Basilar artery syndrome \| \| 435.1 Vertebral artery syndrome \| \| 435.2 Subclavian steal syndrome \| \| 435.3 Vertebrobasilar artery syndrome \| \| 435.8 Other specified transient cerebral ischemias \| \| 435.9 Unspecified transient cerebral ischemia \| \|  \| \| 436 ACUTE BUT ILL-DEFINED CEREBROVASCULAR DISEASE \| \|  \| \| 437 OTHER AND ILL-DEFINED CEREBROVASCULAR DISEASE \| \| 437.0 CEREBRAL ATHEROSCLEROSIS \| \| 437.1 OTHER GENERALIZED ISCHAEMIC CEREBROVASCULAR DISEASE \| \| 437.2 HYPERTENSIVE ENCEPHALOPATHY \| \| 437.3 CEREBRAL ANEURYSM, NONRUPTURED \| \| 437.4 CEREBRAL ARTERITIS \| \| 437.5 MOYAMOYA DISEASE \| \| 437.6 NONPYOGENIC THROMBOSIS OF INTRACRANIAL VENOUS SINUS \| \| 437.7 Transient global amnesia \| \| 437.8 OTHER \| \| 437.9 UNSPECIFIED \| \|  \| \| 438 LATE EFFECTS OF CEREBROVASCULAR DISEASE \| \| 438.0 Late effects of cerebrovascular disease, cognitive deficits \| \| 438.1 Speech and language deficits \| \| 438.10 Late effects of cerebrovascular disease, speech and language deficit, unspecified \| \| 438.11 Late effects of cerebrovascular disease, aphasia \| \| 438.12 Late effects of cerebrovascular disease, dysphasia \| \| 438.13 Late effects of cerebrovascular disease, dysarthria \| \| 438.14 Late effects of cerebrovascular disease, fluency disorder \| \| 438.19 Late effects of cerebrovascular disease, other speech and language deficits \| \| 438.2 Hemiplegia/hemiparesis \| \| 438.20 Late effects of cerebrovascular disease, hemiplegia affecting unspecified side \| \| 438.21 Late effects of cerebrovascular disease, hemiplegia affecting dominant side \| \| 438.22 Late effects of cerebrovascular disease, hemiplegia affecting nondominant side \| \| 438.3 Monoplegia of upper limb \| \| 438.30 Late effects of cerebrovascular disease, monoplegia of upper limb affecting unspecified side \| \| 438.31 Late effects of cerebrovascular disease, monoplegia of upper limb affecting dominant side \| \| 438.32 Late effects of cerebrovascular disease, monoplegia of upper limb affecting nondominant side \| \| 438.4 Monoplegia of lower limb \| \| 438.40 Late effects of cerebrovascular disease, monoplegia of lower limb affecting unspecified side \| \| 438.41 Late effects of cerebrovascular disease, monoplegia of lower limb affecting dominant side \| \| 438.42 Late effects of cerebrovascular disease, monoplegia of lower limb affecting nondominant side \| \| 438.5 Other paralytic syndrome \| \| 438.50 Late effects of cerebrovascular disease, other paralytic syndrome affecting unspecified side \| \| 438.51 Late effects of cerebrovascular disease, other paralytic syndrome affecting dominant side \| \| 438.52 Late effects of cerebrovascular disease, other paralytic syndrome affecting nondominant side \| \| 438.53 Late effects of cerebrovascular disease, other paralytic syndrome, bilateral \| \| 438.6 Late effects of cerebrovascular disease, alterations of sensations \| \| 438.7 Late effects of cerebrovascular disease, disturbances of vision \| \| 438.8 Other late effects of cerebrovascular disease \| \| 438.81 Other late effects of cerebrovascular disease, apraxia \| \| 438.82 Other late effects of cerebrovascular disease, dysphagia \| \| 438.83 Other late effects of cerebrovascular disease, facial weakness \| \| 438.84 Other late effects of cerebrovascular disease, ataxia \| \| 438.85 Other late effects of cerebrovascular disease, vertigo \| \| 438.89 Other late effects of cerebrovascular disease \| \| 438.9 Unspecified late effects of cerebrovascular disease \| \|  \| \| V12.54 Personal history of transient ischemic attack (TIA), and cerebral infarction without residual deficits \| \|  \| \| Brain aneurysm \| \| Brain ischemia \| \| Cerebellar atrophy \| \| Cerebellar CVA \| \| Cerebral aneurysm \| \| Cerebral artery occlusion \| \| Cerebral CVA \| \| Cerebral degeneration \| \| Cerebral infarction \| \| Cerebral thrombosis \| \| Cerebrovascular accident \| \| Cerebrovascular disease \| \| CVA \| \| Intracerebral haemorrhage \| \| Intracerebral hemorrhage \| \| Intracranial aneurysm \| \| Intracranial bleed  Lacunar \| \| Mini stroke \| \| Post stroke \| \| Post CVA \| \| Post TIA \| \| Stroke \| \| Stroke clinic \| \| Stroke recovery \| \| Subarachnoid haemorrhage \| \| Subarachnoid hemorrhage \| \| Subdural hematoma \| \| Subdural haemorrhage \| \| Subdural hemorrhage \| \| Transient ischemic attack \| \|  \| \| \| --- \| --- \| --- \| --- \| --- \| --- \| --- \| --- \| --- \| --- \| --- \| --- \| --- \| --- \| --- \| --- \| --- \| --- \| --- \| --- \| --- \| --- \| --- \| --- \| --- \| --- \| --- \| --- \| --- \| --- \| --- \| --- \| --- \| --- \| --- \| --- \| --- \| --- \| --- \| --- \| --- \| --- \| --- \| --- \| --- \| --- \| --- \| --- \| --- \| --- \| --- \| --- \| --- \| --- \| --- \| --- \| --- \| --- \| --- \| --- \| --- \| --- \| --- \| --- \| --- \| --- \| --- \| --- \| --- \| --- \| --- \| --- \| --- \| --- \| --- \| --- \| --- \| --- \| --- \| --- \| --- \| --- \| --- \| --- \| --- \| --- \| --- \| --- \| --- \| --- \| --- \| --- \| --- \| --- \| --- \| --- \| --- \| --- \| --- \| --- \| --- \| --- \| --- \| --- \| --- \| --- \| --- \| --- \| --- \| --- \| --- \| --- \| --- \| --- \| --- \| --- \| --- \| --- \| --- \| --- \| --- \| --- \| --- \| --- \| --- \| --- \| --- \| --- \| --- \| --- \| --- \| --- \| | \| **ICD9/TERMWORKS** \| \| --- \| \| **ICD9/TERMWORKS** \| \|  \| \| **ICD9/TERMWORKS** \| \| **ICD9** \| \| **ICD9/TERMWORKS** \| \| **ICD9/TERMWORKS** \| \|  \| \| **ICD9** \| \| **ICD9** \| \| **ICD9-CM/TERMWORKS** \| \| **ICD9-CM/TERMWORKS** \| \| **ICD9/TERMWORKS** \| \| **ICD9-CM/TERMWORKS** \| \| **ICD9-CM/TERMWORKS** \| \| **ICD9** \| \| **ICD9-CM/TERMWORKS** \| \| **ICD9-CM/TERMWORKS** \| \| **ICD9** \| \| **ICD9-CM/TERMWORKS** \| \| **ICD9-CM/TERMWORKS** \| \| **ICD9** \| \| **ICD9-CM/TERMWORKS** \| \| **ICD9-CM/TERMWORKS** \| \| **ICD9** \| \| **ICD9-CM/TERMWORKS** \| \| **ICD9-CM/TERMWORKS** \| \|  \| \| **ICD9/TERMWORKS** \| \| **ICD9/TERMWORKS** \| \| **ICD9-CM/TERMWORKS** \| \| **ICD9-CM/TERMWORKS** \| \| **ICD9/TERMWORKS** \| \| **ICD9-CM/TERMWORKS** \| \| **ICD9-CM/TERMWORKS** \| \| **ICD9/TERMWORKS** \| \| **ICD9-CM/TERMWORKS** \| \| **ICD9-CM/TERMWORKS** \| \|  \| \| **ICD9/TERMWORKS** \| \| **ICD9-CM** \| \| **ICD9-CM** \| \| **ICD9-CM** \| \| **ICD9-CM** \| \| **ICD9-CM** \| \| **ICD9-CM/TERMWORKS** \| \|  \| \| **ICD9/TERMWORKS** \| \|  \| \| **ICD9/TERMWORKS** \| \| **ICD9/TERMWORKS** \| \| **ICD9/TERMWORKS** \| \| **ICD9** \| \| **ICD9/TERMWORKS** \| \| **ICD9/TERMWORKS** \| \| **ICD9** \| \| **ICD9** \| \| **ICD9-CM** \| \| **ICD9/TERMWORKS** \| \| **ICD9/TERMWORKS** \| \|  \| \| **ICD9/TERMWORKS** \| \| **ICD9-CM/TERMWORKS** \| \| **ICD9-CM** \| \| **ICD9-CM/TERMWORKS** \| \| **ICD9-CM/TERMWORKS** \| \| **ICD9-CM/TERMWORKS** \| \| **ICD9-CM/TERMWORKS** \| \| **ICD9-CM/TERMWORKS** \| \| **ICD9-CM/TERMWORKS** \| \| **ICD9-CM** \| \| **ICD9-CM/TERMWORKS** \| \| **ICD9-CM/TERMWORKS** \| \| **ICD9-CM/TERMWORKS** \| \| **ICD9-CM** \| \| **ICD9-CM/TERMWORKS** \| \| **ICD9-CM/TERMWORKS** \| \| **ICD9-CM/TERMWORKS** \| \| **ICD9-CM** \| \| **ICD9-CM/TERMWORKS** \| \| **ICD9-CM/TERMWORKS** \| \| **ICD9-CM/TERMWORKS** \| \| **ICD9-CM** \| \| **ICD9-CM/TERMWORKS** \| \| **ICD9-CM/TERMWORKS** \| \| **ICD9-CM/TERMWORKS** \| \| **ICD9-CM/TERMWORKS** \| \| **ICD9-CM/TERMWORKS** \| \| **ICD9-CM/TERMWORKS** \| \| **ICD9-CM/TERMWORKS** \| \| **ICD9-CM/TERMWORKS** \| \| **ICD9-CM/TERMWORKS** \| \| **ICD9-CM/TERMWORKS** \| \| **ICD9-CM/TERMWORKS** \| \| **ICD9-CM/TERMWORKS** \| \| **ICD9-CM/TERMWORKS** \| \| **ICD9-CM/TERMWORKS** \| \|  \| \| **ICD9-CM/TERMWORKS** \| \|  \| \| **CPCSSN Free Text** \| \| **CPCSSN Free Text** \| \| **CPCSSN Free Text** \| \| **CPCSSN Free Text** \| \| **CPCSSN Free Text** \| \| **CPCSSN Free Text** \| \| **CPCSSN Free Text** \| \| **CPCSSN Free Text** \| \| **CPCSSN Free Text** \| \| **CPCSSN Free Text** \| \| **CPCSSN Free Text** \| \| **CPCSSN Free Text** \| \| **CPCSSN Free Text** \| \| **CPCSSN Free Text** \| \| **CPCSSN Free Text** \| \| **CPCSSN Free Text** \| \| **CPCSSN Free Text** \| \| **CPCSSN Free Text/** \| \| **CPCSSN Free Text** \| \| **CPCSSN Free Text** \| \| **CPCSSN Free Text** \| \| **CPCSSN Free Text** \| \| **CPCSSN Free Text** \| \| **CPCSSN Free Text** \| \| **CPCSSN Free Text** \| \| **CPCSSN Free Text** \| \| **CPCSSN Free Text** \| \| **CPCSSN Free Text** \| \| **CPCSSN Free Text** \| \| **CPCSSN Free Text** \| \| **CPCSSN Free Text** \| |

Termworks search terms: cerebrovascular, stroke, amaurosis fugax, cerebral, cerebral thrombosis, Intracerebral hemorrhage, subdural haemorrhage, carotid artery, cerebral ischemia, cerebral artery, subarachnoid hemorrhage, Intracerebral hemorrhage, intracranial hemorrhage, transient ischaemic attack, CVA, TIA

## Chronic kidney disease

| **Codes and Terms** | **Source(s)** |
| --- | --- |
| \| \| 581 NEPHROTIC SYNDROME \| \| --- \| \| 581.0 WITH LESION OF PROLIFERATIVE GLOMERULONEPHRITIS \| \| 581.1 WITH LESION OF MEMBRANOUS GLOMERULONEPHRITIS \| \| 581.2 WITH LESION OF MEMBRANOPROLIFERATIVE GLOMERULONEPHRITIS \| \| 581.3 WITH LESION OF MINIMAL CHANGE GLOMERULONEPHRITIS \| \| 581.8 WITH OTHER SPECIFIED PATHOLOGICAL LESION IN KIDNEY \| \| 581.81 Nephrotic syndrome in diseases classified elsewhere \| \| 581.89 Nephrotic syndrome with other specified pathological lesion in kidney \| \| 581.9 UNSPECIFIED \| \|  \| \| 582 CHRONIC GLOMERULONEPHRITIS \| \| 582.0 WITH LESION OF PROLIFERATIVE GLOMERULONEPHRITIS \| \| 582.1 WITH LESION OF MEMBRANOUS GLOMERULONEPHRITIS \| \| 582.2 WITH LESION OF MEMBRANOPROLIFERATIVE GLOMERULONEPHRITIS \| \| 582.4 WITH LESION OF RAPIDLY PROGRESSIVE GLOMERULONEPHRITIS \| \| 582.8 WITH OTHER SPECIFIED PATHOLOGICAL LESION IN KIDNEY \| \| 582.81 Chronic glomerulonephritis in diseases classified elsewhere \| \| 582.89 Chronic glomerulonephritis with other specified pathological lesion in kidney \| \| 582.9 UNSPECIFIED \| \|  \| \| 583 NEPHRITIS AND NEPHROPATHY, NOT SPECIFIED AS ACUTE OR CHRONIC \| \| 583.0 WITH LESION OF PROLIFERATIVE GLOMERULONEPHRITIS \| \| 583.1 WITH LESION OF MEMBRANOUS GLOMERULONEPHRITIS \| \| 583.2 WITH LESION OF MEMBRANOPROLIFERATIVE GLOMERULONEPHRITIS \| \| 583.4 WITH LESION OF RAPIDLY PROGRESSIVE GLOMERULONEPHRITIS \| \| 583.6 WITH LESION OF RENAL CORTICAL NECROSIS \| \| 583.7 WITH LESION OF RENAL MEDULLARY NECROSIS \| \| 583.8 WITH OTHER SPECIFIED PATHOLOGICAL LESION IN KIDNEY \| \| 583.81 Nephritis and nephropathy, not specified as acute or chronic, in diseases classified elsewhere \| \| 583.89 Nephritis and nephropathy, not specified as acute or chronic, with other specified pathological lesion in kidney \| \| 583.9 WITH UNSPECIFIED PATHOLOGICAL LESION IN KIDNEY \| \|  \| \| 585 CHRONIC RENAL FAILURE (in ICD9-CM 585 CHRONIC KIDNEY DISEASE) \| \| 585.1 Chronic kidney disease, Stage I \| \| 585.2 Chronic kidney disease, Stage II (mild) \| \| 585.3 Chronic kidney disease, Stage III (moderate) \| \| 585.4 Chronic kidney disease, Stage IV (severe) \| \| 585.5 Chronic kidney disease, Stage V \| \| 585.6 End stage renal disease \| \| 585.9 Chronic kidney disease, unspecified \| \| 586 RENAL FAILURE, UNSPECIFIED \| \|  \| \| 588 DISORDERS RESULTING FROM IMPAIRED RENAL FUNCTION \| \| 588.0 RENAL OSTEODYSTROPHY \| \| 588.1 NEPHROGENIC DIABETES INSIPIDUS \| \| 588.8 OTHER \| \| 588.81 Secondary hyperparathyroidism (of renal origin) \| \| 588.89 Other specified disorders resulting from impaired renal function \| \| 588.9 UNSPECIFIED \| \|  \| \| 590.0 CHRONIC PYELONEPHRITIS AND CHRONIC PYONEPHROSIS (Under 590 Infections of Kidney) \| \| 590.00 Chronic pyelonephritis without lesion of renal medullary necrosis \| \| 590.01 Chronic pyelonephritis with lesion of renal medullary necrosis \| \| 590.8 PYELONEPHRITIS OR PYONEPHROSIS, NOT SPECIFIED AS ACUTE OR CHRONIC \| \| 590.80 Pyelonephritis, unspecified \| \| 590.81 Pyelitis or pyelonephritis in diseases classified elsewhere \| \|  \| \| NON-SPECIFIC ABNORMAL FINDINGS \| \| 791 NONSPECIFIC FINDINGS ON EXAMINATION OF URINE \| \| 791.0 PROTEINURIA \| \| 791.1 CHYLURIA \| \| 791.2 HAEMOGLOBINURIA \| \| 791.3 MYOGLOBINURIA \| \| 791.4 BILIURIA \| \| 791.5 GLYCOSURIA \| \| 791.6 ACETONURIA \| \| 791.7 OTHER CELLS AND CASTS IN URINE \| \| 791.9 OTHER \| \|  \| \| V81.5 SCREENING FOR NEPHROPATHY \| \|  \| \| **Proteinuria** \| \| mass/volume in urine: >100 mg/24 hour collection \| \| presence \| \|  \| \| **Albuminuria** \| \| mass/volume in urine: >25 mg/24-hour collection \| \| presence \| \|  \| \| **eGFR** <60 mL/min/1.73 m^2 \| \|  \| \| Chronic kidney disease \| \| CKD \| \| CRF \| \| Chronic renal failure \| \| End stage renal disease \| \| Microalbuminuria \| \| Nephrology \| \| Nephropathy \| \| Nephrotic syndrome \| \| Proteinuria \| \| Renal disease \| \| Renal disease chronic \| \| Renal disease end stage \| \| Renal failure \| \| Renal insufficiency \| \| Uremia \| \|  \| \| \| --- \| --- \| --- \| --- \| --- \| --- \| --- \| --- \| --- \| --- \| --- \| --- \| --- \| --- \| --- \| --- \| --- \| --- \| --- \| --- \| --- \| --- \| --- \| --- \| --- \| --- \| --- \| --- \| --- \| --- \| --- \| --- \| --- \| --- \| --- \| --- \| --- \| --- \| --- \| --- \| --- \| --- \| --- \| --- \| --- \| --- \| --- \| --- \| --- \| --- \| --- \| --- \| --- \| --- \| --- \| --- \| --- \| --- \| --- \| --- \| --- \| --- \| --- \| --- \| --- \| --- \| --- \| --- \| --- \| --- \| --- \| --- \| --- \| --- \| --- \| --- \| --- \| --- \| --- \| --- \| --- \| --- \| --- \| --- \| --- \| --- \| --- \| --- \| --- \| --- \| --- \| --- \| --- \| --- \| --- \| --- \| --- \| --- \| --- \| | \| **ICD9/TERMWORKS** \| \| --- \| \| **ICD9** \| \| **ICD9** \| \| **ICD9** \| \| **ICD9** \| \| **ICD9** \| \| **ICD9-CM** \| \| **ICD9-CM/TERMWORKS** \| \| **ICD9** \| \|  \| \| **ICD9** \| \| **ICD9** \| \| **ICD9** \| \| **ICD9** \| \| **ICD9** \| \| **ICD9/TERMWORKS** \| \| **ICD9-CM** \| \| **ICD9-CM/TERMWORKS** \| \| **ICD9/TERMWORKS** \| \|  \| \| **ICD9/TERMWORKS** \| \| **ICD9/TERMWORKS** \| \| **ICD9/TERMWORKS** \| \| **ICD9/TERMWORKS** \| \| **ICD9/TERMWORKS** \| \| **ICD9/TERMWORKS** \| \| **ICD9/TERMWORKS** \| \| **ICD9/TERMWORKS** \| \| **ICD9-CM/TERMWORKS** \| \| **ICD9-CM/TERMWORKS** \| \| **ICD9/TERMWORKS** \| \|  \| \| **ICD9/TERMWORKS** \| \| **ICD9-CM/TERMWORKS** \| \| **ICD9-CM/TERMWORKS** \| \| **ICD9-CM/TERMWORKS** \| \| **ICD9-CM/TERMWORKS** \| \| **ICD9-CM/TERMWORKS** \| \| **ICD9-CM/TERMWORKS** \| \| **ICD9-CM/TERMWORKS** \| \| **ICD9/TERMWORKS** \| \|  \| \| **ICD9/TERMWORKS** \| \| **ICD9/TERMWORKS** \| \| **ICD9** \| \| **ICD9/TERMWORKS** \| \| **ICD9-CM/TERMWORKS** \| \| **ICD9-CM/TERMWORKS** \| \| **ICD9/TERMWORKS** \| \|  \| \| **ICD9** \| \| **ICD9-CM** \| \| **ICD9-CM** \| \| **ICD9** \| \| **ICD9-CM** \| \| **ICD9-CM** \| \|  \| \|  \| \| **ICD9/TERMWORKS** \| \| **ICD9** \| \| **ICD9** \| \| **ICD9** \| \| **ICD9** \| \| **ICD9** \| \| **ICD9** \| \| **ICD9** \| \| **ICD9/TERMWORKS** \| \| **ICD9/TERMWORKS** \| \|  \| \| **ICD9/TERMWORKS** \| \|  \| \|  \| \| **LOINC 2889-4** \| \| **LOINC 2887-8** \| \|  \| \|  \| \| **LOINC 1755-8** \| \| **LOINC 1754-1** \| \|  \| \| **LOINC 12195-4** \| \|  \| \| **CPCSSN Free Text** \| \| **CPCSSN Free Text** \| \| **CPCSSN Free Text** \| \| **CPCSSN Free Text** \| \| **CPCSSN Free Text** \| \| **CPCSSN Free Text** \| \| **CPCSSN Free Text** \| \| **CPCSSN Free Text** \| \| **CPCSSN Free Text** \| \| **CPCSSN Free Text** \| \| **CPCSSN Free Text** \| \| **CPCSSN Free Text** \| \| **CPCSSN Free Text** \| \| **CPCSSN Free Text** \| \| **CPCSSN Free Text** \| \| **CPCSSN Free Text** \| \|  \| |

Termworks search terms: chronic kidney disease, kidney, renal, urine, nephropathy, proteinuria

## Diabetes

| **Codes and Terms** | **Source(s)** |
| --- | --- |
| \| \| 249 Secondary diabetes mellitus \| \| --- \| \| 249.0 Secondary diabetes mellitus without mention of complication \| \| 249.00 Secondary diabetes mellitus without mention of complication, not stated as uncontrolled, or unspecified \| \| 249.01 Secondary diabetes mellitus without mention of complication, uncontrolled \| \| 249.1 Secondary diabetes mellitus with ketoacidosis \| \| 249.10 Secondary diabetes mellitus with ketoacidosis, not stated as uncontrolled, or unspecified \| \| 249.11 Secondary diabetes mellitus with ketoacidosis, uncontrolled \| \| 249.2 Secondary diabetes mellitus with hyperosmolarity \| \| 249.20 Secondary diabetes mellitus with hyperosmolarity, not stated as uncontrolled, or unspecified \| \| 249.21 Secondary diabetes mellitus with hyperosmolarity, uncontrolled \| \| 249.3 Secondary diabetes mellitus with other coma \| \| 249.30 Secondary diabetes mellitus with other coma, not stated as uncontrolled, or unspecified \| \| 249.31 Secondary diabetes mellitus with other coma, uncontrolled \| \| 249.4 Secondary diabetes mellitus with renal manifestations \| \| 249.40 Secondary diabetes mellitus with renal manifestations, not stated as uncontrolled, or unspecified \| \| 249.41 Secondary diabetes mellitus with renal manifestations, uncontrolled \| \| 249.5 Secondary diabetes mellitus with ophthalmic manifestations \| \| 249.50 Secondary diabetes mellitus with ophthalmic manifestations, not stated as uncontrolled, or unspecified \| \| 249.51 Secondary diabetes mellitus with ophthalmic manifestations, uncontrolled \| \| 249.6 Secondary diabetes mellitus with neurological manifestations \| \| 249.60 Secondary diabetes mellitus with neurological manifestations, not stated as uncontrolled, or unspecified \| \| 249.61 Secondary diabetes mellitus with neurological manifestations, uncontrolled \| \| 249.7 Secondary diabetes mellitus with peripheral circulatory disorders \| \| 249.70 Secondary diabetes mellitus with peripheral circulatory disorders, not stated as uncontrolled, or unspecified \| \| 249.71 Secondary diabetes mellitus with peripheral circulatory disorders, uncontrolled \| \| 249.8 Secondary diabetes mellitus with other specified manifestations \| \| 249.80 Secondary diabetes mellitus with other specified manifestations, not stated as uncontrolled, or unspecified \| \| 249.81 Secondary diabetes mellitus with other specified manifestations, uncontrolled \| \| 249.9 Secondary diabetes mellitus with unspecified complication \| \| 249.90 Secondary diabetes mellitus with unspecified complication, not stated as uncontrolled, or unspecified \| \| 249.91 Secondary diabetes mellitus with unspecified complication, uncontrolled \| \|  \| \| 250 DIABETES MELLITUS \| \| 250.0 DIABETES MELLITUS WITHOUT MENTION OF COMPLICATION \| \| 250.00 Diabetes mellitus without mention of complication, type II or unspecified type, not stated as uncontrolled \| \| 250.01 Diabetes mellitus without mention of complication, type I [juvenile type], not stated as uncontrolled \| \| 250.02 Diabetes mellitus without mention of complication, type II or unspecified type, uncontrolled \| \| 250.03 Diabetes mellitus without mention of complication, type I [juvenile type], uncontrolled \| \| 250.1 DIABETES WITH KETOACIDOSIS \| \| 250.10 Diabetes with ketoacidosis, type II or unspecified type, not stated as uncontrolled \| \| 250.11 Diabetes with ketoacidosis, type I [juvenile type], not stated as uncontrolled \| \| 250.12 Diabetes with ketoacidosis, type II or unspecified type, uncontrolled \| \| 250.13 Diabetes with ketoacidosis, type I [juvenile type], uncontrolled \| \| 250.2 DIABETES WITH COMA (250.3 in ICD9-CM) \| \| 250.30 Diabetes with other coma, type II or unspecified type, not stated as uncontrolled \| \| 250.31 Diabetes with other coma, type I [juvenile type], not stated as uncontrolled \| \| 250.32 Diabetes with other coma, type II or unspecified type, uncontrolled \| \| 250.33 Diabetes with other coma, type I [juvenile type], uncontrolled \| \| 250.2 Diabetes with hyperosmolarity \| \| 250.20 Diabetes with hyperosmolarity, type II or unspecified type, not stated as uncontrolled \| \| 250.21 Diabetes with hyperosmolarity, type I [juvenile type], not stated as uncontrolled \| \| 250.22 Diabetes with hyperosmolarity, type II or unspecified type, uncontrolled \| \| 250.23 Diabetes with hyperosmolarity, type I [juvenile type], uncontrolled \| \| 250.3 DIABETES WITH RENAL MANIFESTATIONS (250.4 in ICD9-CM) \| \| 250.40 Diabetes with renal manifestations, type II or unspecified type, not stated as uncontrolled \| \| 250.41 Diabetes with renal manifestations, type I [juvenile type], not stated as uncontrolled \| \| 250.42 Diabetes with renal manifestations, type II or unspecified type, uncontrolled \| \| 250.43 Diabetes with renal manifestations, type I [juvenile type], uncontrolled \| \| 250.4 DIABETES WITH OPHTHALMIC MANIFESTATIONS (250.5 in ICD9-CM) \| \| 250.50 Diabetes with ophthalmic manifestations, type II or unspecified type, not stated as uncontrolled \| \| 250.51 Diabetes with ophthalmic manifestations, type I [juvenile type], not stated as uncontrolled \| \| 250.52 Diabetes with ophthalmic manifestations, type II or unspecified type, uncontrolled \| \| 250.53 Diabetes with ophthalmic manifestations, type I [juvenile type], uncontrolled \| \| 250.5 DIABETES WITH NEUROLOGICAL MANIFESTATIONS (250.6 in ICD9-CM) \| \| 250.50 DIABETES WITH OCULAR INVOLVMENT, ADULT \| \| 250.60 Diabetes with neurological manifestations, type II or unspecified type, not stated as uncontrolled \| \| 250.61 Diabetes with neurological manifestations, type I [juvenile type], not stated as uncontrolled \| \| 250.62 Diabetes with neurological manifestations, type II or unspecified type, uncontrolled \| \| 250.63 Diabetes with neurological manifestations, type I [juvenile type], uncontrolled \| \| 250.6 DIABETES WITH PERIPHERAL CIRCULATORY DISORDERS (250.7 in ICD9-CM) \| \| 250.70 Diabetes with peripheral circulatory disorders, type II or unspecified type, not stated as uncontrolled \| \| 250.71 Diabetes with peripheral circulatory disorders, type I [juvenile type], not stated as uncontrolled \| \| 250.72 Diabetes with peripheral circulatory disorders, type II or unspecified type, uncontrolled \| \| 250.73 Diabetes with peripheral circulatory disorders, type I [juvenile type], uncontrolled \| \| 250.7 DIABETES WITH OTHER SPECIFIED MANIFESTATIONS (250.8 in ICD9-CM) \| \| 250.80 Diabetes with other specified manifestations, type II or unspecified type, not stated as uncontrolled \| \| 250.81 Diabetes with other specified manifestations, type I [juvenile type], not stated as uncontrolled \| \| 250.82 Diabetes with other specified manifestations, type II or unspecified type, uncontrolled \| \| 250.83 Diabetes with other specified manifestations, type I [juvenile type], uncontrolled \| \| 250.9 DIABETES WITH UNSPECIFIED COMPLICATIONS \| \| 250.90 Diabetes with unspecified complication, type II or unspecified type, not stated as uncontrolled \| \| 250.91 Diabetes with unspecified complication, type I [juvenile type], not stated as uncontrolled \| \| 250.92 Diabetes with unspecified complication, type II or unspecified type, uncontrolled \| \| 250.93 Diabetes with unspecified complication, type I [juvenile type], uncontrolled \| \|  \| \| 251.0 HYPOGLYCAEMIC COMA \| \| 251.1 OTHER HYPERINSULINISM \| \| 251.2 HYPOGLYCAEMIA, UNSPECIFIED \| \| 251.4 ABNORMALITY OF SECRETION OF GLUCAGON \| \|  \| \| DISEASES OF NERVOUS SYSTEM AND SENSE ORGANS \| \| 357.2 POLYNEUROPATHY IN DIABETES \| \|  \| \| V53.91 Fitting and adjustment of insulin pump \| \| V58.67 Long-term (current) use of insulin \| \| V65.46 Encounter for insulin pump training \| \| V77.1 SCREENING FOR DIABETES MELLITUS \| \|  \| \| 790.2 Abnormal glucose \| \| 790.21 Impaired fasting glucose \| \| 790.22 Impaired glucose tolerance test (oral) \| \| 790.29 Other abnormal glucose \| \|  \| \| **Hgb A1C (%)** GREATER OR EQUAL TO 6.5% \| \| Any Hemoglobin A1c (HbA1c) test (LOINC = 17856-6) in the Lab table with a result ≥ 6.5 \| \|  \| \| **Fasting Blood Glucose** GREATER OR EQUAL TO 7.0 MMOL/L (mmol/L (serum/plasma)) \| \|  \| \| **Insulin Prescription** \| \|  \| \| BG management \| \| Blood sugar elevated \| \| DM \| \| CDM GV \| \| Diabetic \| \| Diabetic foot \| \| Diabetic foot ulcer \| \| Diabetic ketoacidosis \| \| Diabetic neuropathy \| \| Diabetic ulcer \| \| Diabetes \| \| Diabetes Insipidus \| \| Diabetes mellitus \| \| DM2 \| \| DMGV \| \| DM Group Visit \| \| DM Review \| \| Hyperglycemia \| \| Hypoglycemia \| \| IFG \| \| Impaired fasting glucose \| \| impaired glucose tolerance \| \| Insulin \| \| Low blood sugar \| \| NIDDM \| \| IDDM \| \| T2 Diabetes \| \|  \| \| \| --- \| --- \| --- \| --- \| --- \| --- \| --- \| --- \| --- \| --- \| --- \| --- \| --- \| --- \| --- \| --- \| --- \| --- \| --- \| --- \| --- \| --- \| --- \| --- \| --- \| --- \| --- \| --- \| --- \| --- \| --- \| --- \| --- \| --- \| --- \| --- \| --- \| --- \| --- \| --- \| --- \| --- \| --- \| --- \| --- \| --- \| --- \| --- \| --- \| --- \| --- \| --- \| --- \| --- \| --- \| --- \| --- \| --- \| --- \| --- \| --- \| --- \| --- \| --- \| --- \| --- \| --- \| --- \| --- \| --- \| --- \| --- \| --- \| --- \| --- \| --- \| --- \| --- \| --- \| --- \| --- \| --- \| --- \| --- \| --- \| --- \| --- \| --- \| --- \| --- \| --- \| --- \| --- \| --- \| --- \| --- \| --- \| --- \| --- \| --- \| --- \| --- \| --- \| --- \| --- \| --- \| --- \| --- \| --- \| --- \| --- \| --- \| --- \| --- \| --- \| --- \| --- \| --- \| --- \| --- \| --- \| --- \| --- \| --- \| --- \| --- \| --- \| --- \| --- \| --- \| --- \| --- \| --- \| --- \| --- \| --- \| --- \| --- \| --- \| | \| **ICD9-CM** \| \| --- \| \| **ICD9-CM** \| \| **ICD9-CM** \| \| **ICD9-CM** \| \| **ICD9-CM** \| \| **ICD9-CM** \| \| **ICD9-CM** \| \| **ICD9-CM** \| \| **ICD9-CM** \| \| **ICD9-CM** \| \| **ICD9-CM** \| \| **ICD9-CM** \| \| **ICD9-CM** \| \| **ICD9-CM** \| \| **ICD9-CM** \| \| **ICD9-CM** \| \| **ICD9-CM** \| \| **ICD9-CM** \| \| **ICD9-CM** \| \| **ICD9-CM** \| \| **ICD9-CM** \| \| **ICD9-CM** \| \| **ICD9-CM** \| \| **ICD9-CM** \| \| **ICD9-CM** \| \| **ICD9-CM** \| \| **ICD9-CM** \| \| **ICD9-CM** \| \| **ICD9-CM** \| \| **ICD9-CM** \| \| **ICD9-CM** \| \|  \| \| **ICD9/TERMWORKS** \| \| **ICD9/TERMWORKS** \| \| **ICD9-CM** \| \| **ICD9-CM** \| \| **ICD9-CM** \| \| **ICD9-CM** \| \| **ICD9/TERMWORKS** \| \| **ICD9-CM** \| \| **ICD9-CM** \| \| **ICD9-CM** \| \| **ICD9-CM** \| \| **ICD9/TERMWORKS** \| \| **ICD9-CM** \| \| **ICD9-CM** \| \| **ICD9-CM** \| \| **ICD9-CM** \| \| **ICD9-CM** \| \| **ICD9-CM** \| \| **ICD9-CM** \| \| **ICD9-CM** \| \| **ICD9-CM** \| \| **ICD9/TERMWORKS** \| \| **ICD9-CM** \| \| **ICD9-CM** \| \| **ICD9-CM** \| \| **ICD9-CM** \| \| **ICD9/TERMWORKS** \| \| **ICD9-CM** \| \| **ICD9-CM** \| \| **ICD9-CM** \| \| **ICD9-CM** \| \| **ICD9/TERMWORKS** \| \| **ICD9/TERMWORKS** \| \| **ICD9-CM** \| \| **ICD9-CM** \| \| **ICD9-CM** \| \| **ICD9-CM** \| \| **ICD9/TERMWORKS** \| \| **ICD9-CM** \| \| **ICD9-CM** \| \| **ICD9-CM** \| \| **ICD9-CM** \| \| **ICD9/TERMWORKS** \| \| **ICD9-CM** \| \| **ICD9-CM** \| \| **ICD9-CM** \| \| **ICD9-CM** \| \| **ICD9/TERMWORKS** \| \| **ICD9-CM** \| \| **ICD9-CM** \| \| **ICD9-CM** \| \| **ICD9-CM** \| \|  \| \| **ICD9** \| \| **ICD9** \| \| **ICD9** \| \| **ICD9** \| \|  \| \|  \| \| **ICD9** \| \|  \| \| **ICD9/TERMWORKS** \| \| **ICD9-CM** \| \| **ICD9-CM** \| \| **ICD9-CM** \| \|  \| \| **ICD9-CM/TERMWORKS** \| \| **ICD9-CM/TERMWORKS** \| \| **ICD9-CM/TERMWORKS** \| \| **ICD9-CM/TERMWORKS** \| \|  \| \| **LOINC 17856-6** \| \|  \| \| **LOINC 14771-0** \| \|  \| \|  \| \|  \| \|  \| \| **CPCSSN Free Text** \| \| **CPCSSN Free Text** \| \| **CPCSSN Free Text** \| \| **CPCSSN Free Text** \| \| **CPCSSN Free Text** \| \| **CPCSSN Free Text** \| \| **CPCSSN Free Text** \| \| **CPCSSN Free Text** \| \| **CPCSSN Free Text** \| \| **CPCSSN Free Text** \| \| **CPCSSN Free Text** \| \| **CPCSSN Free Text** \| \| **CPCSSN Free Text** \| \| **CPCSSN Free Text** \| \| **CPCSSN Free Text** \| \| **CPCSSN Free Text** \| \| **CPCSSN Free Text** \| \| **CPCSSN Free Text** \| \| **CPCSSN Free Text** \| \| **CPCSSN Free Text** \| \| **CPCSSN Free Text** \| \| **CPCSSN Free Text** \| \| **CPCSSN Free Text** \| \| **CPCSSN Free Text** \| \| **CPCSSN Free Text** \| \| **CPCSSN Free Text** \| \| **CPCSSN Free Text** \| \|  \| |

Termworks search terms: diabetes, Insulin, diabetic, glucose

## Dizziness

| **Codes and Terms** | **Source(s)** |
| --- | --- |
| \| \| 386 VERTIGINOUS SYNDROMES AND OTHER DISORDERS OF VESTIBULAR SYSTEM \| \| --- \| \| 386.0 MENIERE'S DISEASE \| \| 386.00 Ménière's disease, unspecified \| \| 386.01 Active Ménière's disease, cochleovestibular \| \| 386.02 Active Ménière's disease, cochlear \| \| 386.03 Active Ménière's disease, vestibular \| \| 386.04 Inactive Ménière's disease \| \| 386.1 OTHER AND UNSPECIFIED PERIPHERAL VERTIGO \| \| 386.10 Peripheral vertigo, unspecified \| \| 386.11 Benign paroxysmal positional vertigo \| \| 386.12 Vestibular neuronitis \| \| 386.19 Other peripheral vertigo \| \| 386.2 VERTIGO OF CENTRAL ORIGIN \| \| 386.3 LABYRINTHITIS \| \| 386.30 Labyrinthitis, unspecified \| \| 386.31 Serous labyrinthitis \| \| 386.32 Circumscribed labyrinthitis \| \| 386.33 Suppurative labyrinthitis \| \| 386.34 Toxic labyrinthitis \| \| 386.35 Viral labyrinthitis \| \| 386.4 LABYRINTHINE FISTULA \| \| 386.40 Labyrinthine fistula, unspecified \| \| 386.41 Round window fistula \| \| 386.42 Oval window fistula \| \| 386.43 Semicircular canal fistula \| \| 386.48 Labyrinthine fistula of combined sites \| \| 386.5 LABYRINTHINE DYSFUNCTION \| \| 386.50 Labyrinthine dysfunction, unspecified \| \| 386.51 Hyperactive labyrinth, unilateral \| \| 386.52 Hyperactive labyrinth, bilateral \| \| 386.53 Hypoactive labyrinth, unilateral \| \| 386.54 Hypoactive labyrinth, bilateral \| \| 386.55 Loss of labyrinthine reactivity, unilateral \| \| 386.56 Loss of labyrinthine reactivity, bilateral \| \| 386.58 Other forms and combinations of labyrinthine dysfunction \| \| 386.8 OTHER DISORDERS OF LABYRINTH \| \| 386.9 UNSPECIFIED VERTIGINOUS SYNDROMES AND LABYRINTHINE DISORDERS \| \|  \| \| 780.4 DIZZINESS AND GIDDINESS \| \| 794.16 Nonspecific abnormal vestibular function studies \| \|  \| \| 01A DIZZINESS, VERTIGO, INSOMNIA \| \|  \| \| Dizziness \| \| Dizzyness \| \| Dizzy \| \| Dizziness chronic \| \| Dizziness intermittent \| \| Dizzy spell \| \| Epidemic vertigo \| \| Peripheral vertigo \| \| Vertigo \| \|  \| \| \| --- \| --- \| --- \| --- \| --- \| --- \| --- \| --- \| --- \| --- \| --- \| --- \| --- \| --- \| --- \| --- \| --- \| --- \| --- \| --- \| --- \| --- \| --- \| --- \| --- \| --- \| --- \| --- \| --- \| --- \| --- \| --- \| --- \| --- \| --- \| --- \| --- \| --- \| --- \| --- \| --- \| --- \| --- \| --- \| --- \| --- \| --- \| --- \| --- \| --- \| --- \| --- \| --- \| --- \| | \| **ICD9/TERMWORKS** \| \| --- \| \| **ICD9/TERMWORKS** \| \| **ICD9/TERMWORKS** \| \| **ICD9-CM/TERMWORKS** \| \| **ICD9-CM/TERMWORKS** \| \| **ICD9-CM/TERMWORKS** \| \| **ICD9-CM/TERMWORKS** \| \| **ICD9/TERMWORKS** \| \| **ICD9-CM/TERMWORKS** \| \| **ICD9-CM/TERMWORKS** \| \| **ICD9-CM/TERMWORKS** \| \| **ICD9-CM/TERMWORKS** \| \| **ICD9/TERMWORKS** \| \| **ICD9** \| \| **ICD9-CM** \| \| **ICD9-CM** \| \| **ICD9-CM** \| \| **ICD9-CM** \| \| **ICD9-CM** \| \| **ICD9-CM** \| \| **ICD9** \| \| **ICD9-CM** \| \| **ICD9-CM** \| \| **ICD9-CM** \| \| **ICD9-CM** \| \| **ICD9-CM** \| \| **ICD9** \| \| **ICD9-CM** \| \| **ICD9-CM** \| \| **ICD9-CM** \| \| **ICD9-CM** \| \| **ICD9-CM** \| \| **ICD9-CM** \| \| **ICD9-CM** \| \| **ICD9-CM** \| \| **ICD9** \| \| **ICD9** \| \|  \| \| **ICD9/TERMWORKS** \| \| **ICD9-CM/TERMWORKS** \| \|  \| \| **ICD9** \| \|  \| \| **CPCSSN Free Text** \| \| **CPCSSN Free Text** \| \| **CPCSSN Free Text** \| \| **CPCSSN Free Text** \| \| **CPCSSN Free Text** \| \| **CPCSSN Free Text** \| \| **CPCSSN Free Text** \| \| **CPCSSN Free Text** \| \| **CPCSSN Free Text** \| |

Termworks search terms: dizziness, faint, vestibular, vertigo, Meniere

## Dyspnea

| **Codes and Terms** | **Source(s)** |
| --- | --- |
| \| \| 786.0 DYSPNOEA AND RESPIRATORY ABNORMALITIES \| \| --- \| \| 786.00 Respiratory abnormality, unspecified \| \| 786.02 Orthopnea \| \| 786.03 Apnea \| \| 786.05 Shortness of breath \| \| 786.09 Other respiratory abnormalities \| \|  \| \| Breathing difficulty \| \| Breathing difficulties \| \| Breathing issues \| \| Breathing problem \| \| Dyspnea on exertion \| \| Dyspnea \| \| Dyspnoea \| \| Dyspneic \| \| Difficult breathing \| \| Difficulty breathing \| \| SOB \| \| Short of breath \| \| Shortness of breath \| \| shortness of breath on exertion \| \| SOBOE \| \| Trouble breathing \| \|  \| \| \| --- \| --- \| --- \| --- \| --- \| --- \| --- \| --- \| --- \| --- \| --- \| --- \| --- \| --- \| --- \| --- \| --- \| --- \| --- \| --- \| --- \| --- \| --- \| --- \| --- \| | \| **ICD9/TERMWORKS** \| \| --- \| \| **ICD9-CM** \| \| **ICD9-CM/TERMWORKS** \| \| **ICD9-CM** \| \| **ICD9-CM/TERMWORKS** \| \| **ICD9-CM/TERMWORKS** \| \|  \| \| **CPCSSN Free Text** \| \| **CPCSSN Free Text** \| \| **CPCSSN Free Text** \| \| **CPCSSN Free Text** \| \| **CPCSSN Free Text** \| \| **CPCSSN Free Text** \| \| **CPCSSN Free Text** \| \| **CPCSSN Free Text** \| \| **CPCSSN Free Text** \| \| **CPCSSN Free Text** \| \| **CPCSSN Free Text** \| \| **CPCSSN Free Text** \| \| **CPCSSN Free Text** \| \| **CPCSSN Free Text** \| \| **CPCSSN Free Text** \| \| **CPCSSN Free Text** \| |

Termworks search terms: dyspnoea, shortness of breath, dyspnea, orthopnea, breathless, breathing

## Falls

| **Codes and Terms** | **Source(s)** |
| --- | --- |
| \| \| ACCIDENTAL FALLS \| \| --- \| \| E880 Accidental fall on or from stairs or steps \| \| E880.0 Accidental fall on or from escalator \| \| E880.1 Accidental fall on or from sidewalk curb \| \| E880.9 Accidental fall on or from other stairs or steps \| \|  \| \| E881 Accidental fall on or from ladders or scaffolding \| \| E881.0 Accidental fall from ladder \| \| E881.1 Accidental fall from scaffolding \| \|  \| \| E884 Other accidental falls from one level to another \| \| E884.2 Accidental fall from chair \| \| E884.3 Accidental fall from wheelchair \| \| E884.4 Accidental fall from bed \| \| E884.5 Accidental fall from other furniture \| \| E884.6 Accidental fall from commode \| \| E884.9 Other accidental fall from one level to another \| \|  \| \| E885 Accidental fall on same level from slipping tripping or stumbling \| \| E885.0 Fall from (nonmotorized) scooter \| \| E885.9 Fall from other slipping, tripping, or stumbling \| \|  \| \| E888 Other and unspecified fall \| \| E888.0 Fall resulting in striking against sharp object \| \| E888.1 Fall resulting in striking against other object \| \| E888.8 Other fall \| \| E888.9 Unspecified fall \| \|  \| \| E929.3 Late effects of accidental fall \| \|  \| \| V15.88 History of fall (Under 15.8 Other specified personal history presenting hazards to health) \| \|  \| \| Fall \| \| Falling \| \| Falls \| \| Fall injury \| \| Fall injuries \| \| Fall multiple \| \| Fall related injury \| \| Fall risk \| \| Fell \| \| Fell down \| \| Frequent falling \| \| Frequent falls \| \| History of fall \| \| Hx fall \| \| Orthostatic fall \| \| Recent fall \| \| Recurrent falls \| \| Risk of falling \| \| Risk of fall \| \| Unwitnessed fall \| \| Witnessed fall \| \|  \| \| \| --- \| --- \| --- \| --- \| --- \| --- \| --- \| --- \| --- \| --- \| --- \| --- \| --- \| --- \| --- \| --- \| --- \| --- \| --- \| --- \| --- \| --- \| --- \| --- \| --- \| --- \| --- \| --- \| --- \| --- \| --- \| --- \| --- \| --- \| --- \| --- \| --- \| --- \| --- \| --- \| --- \| --- \| --- \| --- \| --- \| --- \| --- \| --- \| --- \| --- \| --- \| --- \| --- \| --- \| --- \| | \| **ICD9-CM/TERMWORKS** \| \| --- \| \| **ICD9-CM/TERMWORKS** \| \| **ICD9-CM/TERMWORKS** \| \| **ICD9-CM/TERMWORKS** \| \|  \| \| **ICD9-CM/TERMWORKS** \| \| **ICD9-CM/TERMWORKS** \| \| **ICD9-CM/TERMWORKS** \| \|  \| \| **ICD9-CM/TERMWORKS** \| \| **ICD9-CM/TERMWORKS** \| \| **ICD9-CM/TERMWORKS** \| \| **ICD9-CM/TERMWORKS** \| \| **ICD9-CM/TERMWORKS** \| \| **ICD9-CM/TERMWORKS** \| \| **ICD9-CM/TERMWORKS** \| \|  \| \| **ICD9-CM/TERMWORKS** \| \| **ICD9-CM/TERMWORKS** \| \| **ICD9-CM/TERMWORKS** \| \|  \| \| **ICD9-CM/TERMWORKS** \| \| **ICD9-CM/TERMWORKS** \| \| **ICD9-CM/TERMWORKS** \| \| **ICD9-CM/TERMWORKS** \| \| **ICD9-CM/TERMWORKS** \| \|  \| \| **ICD9-CM/TERMWORKS** \| \|  \| \| **ICD9-CM/TERMWORKS** \| \|  \| \| **CPCSSN Free Text** \| \| **CPCSSN Free Text** \| \| **CPCSSN Free Text** \| \| **CPCSSN Free Text** \| \| **CPCSSN Free Text** \| \| **CPCSSN Free Text** \| \| **CPCSSN Free Text** \| \| **CPCSSN Free Text** \| \| **CPCSSN Free Text** \| \| **CPCSSN Free Text** \| \| **CPCSSN Free Text** \| \| **CPCSSN Free Text** \| \| **CPCSSN Free Text** \| \| **CPCSSN Free Text** \| \| **CPCSSN Free Text** \| \| **CPCSSN Free Text** \| \| **CPCSSN Free Text** \| \| **CPCSSN Free Text** \| \| **CPCSSN Free Text** \| \| **CPCSSN Free Text** \| \| **CPCSSN Free Text** \| |

Termworks search terms: fall, falls

## Foot problems

| **Codes and Terms** | **Source(s)** |
| --- | --- |
| \| \| 727.06 Tenosynovitis of foot and ankle \| \| --- \| \| 727.1 BUNION \| \| 727.67 Nontraumatic rupture of achilles tendon \| \| 727.68 Nontraumatic rupture of other tendons of foot and ankle \| \|  \| \| 730.07 Acute osteomyelitis, ankle and foot \| \| 730.17 Chronic osteomyelitis, ankle and foot \| \| 730.27 Unspecified osteomyelitis, ankle and foot \| \| 730.37 Periostitis, without mention of osteomyelitis, ankle and foot \| \| 730.77 Osteopathy resulting from poliomyelitis, ankle and foot \| \| 730.87 Other infections involving bone in diseases classified elsewhere, ankle and foot \| \| 730.97 Unspecified infection of bone, ankle and foot \| \|  \| \| OSTEOPATHIES, CHONDROPATHIES AND ACQUIRED MUSCULOSKELETAL DEFORMITIES \| \| 734 FLAT FOOT \| \| 735 ACQUIRED DEFORMITIES OF TOE* \| \| 735.0 HALLUX VALGUS (ACQUIRED) \| \| 735.1 HALLUX VARUS (ACQUIRED) \| \| 735.2 HALLUX RIGIDUS \| \| 735.3 HALLUX MALLEUS \| \| 735.4 OTHER HAMMER TOE (ACQUIRED) \| \| 735.5 CLAWTOE (ACQUIRED) \| \| 735.8 OTHER \| \| 735.9 UNSPECIFIED \| \|  \| \| 736.7 OTHER ACQUIRED DEFORMITIES OF ANKLE AND FOOT \| \| 736.70 Unspecified deformity of ankle and foot, acquired \| \| 736.71 Acquired equinovarus deformity \| \| 736.72 Equinus deformity of foot, acquired \| \| 736.73 Cavus deformity of foot, acquired \| \| 736.74 Claw foot, acquired \| \| 736.75 Cavovarus deformity of foot, acquired \| \| 736.76 Other acquired calcaneus deformity \| \| 736.79 Other acquired deformities of ankle and foot \| \|  \| \| INFECTIONS OF SKIN AND SUBCUTANEOUS TISSUE \| \| 680.7 CARBUNCLE AND FURUNCLE OF FOOT \| \|  \| \| OTHER DISEASES OF SKIN AND SUBCUTANEOUS TISSUE \| \| 681 CELLULITIS AND ABSCESS OF FINGER AND TOE \| \| 681.1 CELLULITIS AND ABSCRESS OF TOE \| \| 681.10 Cellulitis and abscess of toe, unspecified \| \| 681.11 Onychia and paronychia of toe \| \| 682.7 CELLULITIS AND ABSCESS OF FOOT, EXCEPT TOES \| \|  \| \| 700 CORNS AND CALLOSITIES \| \| 703 DISEASES OF NAIL \| \| 703.0 INGROWING NAIL \| \| 703.8 OTHER DISEASES OF NAIL \| \| 703.9 Unspecified disease of nail \| \|  \| \| 845 SPRAINS AND STRAINS OF ANKLE AND FOOT \| \| 845.0 ANKLE \| \| 845.00 Sprain of ankle, unspecified site \| \| 845.01 Sprain of deltoid (ligament), ankle \| \| 845.02 Sprain of calcaneofibular (ligament) of ankle \| \| 845.03 Sprain of tibiofibular (ligament), distal of ankle \| \| 845.09 Other sprains and strains of ankle \| \| 845.1 FOOT \| \| 845.10 Sprain of foot, unspecified site \| \| 845.11 Sprain of tarsometatarsal (joint) (ligament) of foot \| \| 845.12 Sprain of metatarsophalangeal (joint) of foot \| \| 845.13 Sprain of interphalangeal (joint), toe \| \| 845.19 Other sprain of foot \| \|  \| \| 892 OPEN WOUND OF FOOT EXCEPT TOE(S) ALONE \| \| 892.0 WITHOUT MENTION OF COMPLICATION \| \| 892.1 COMPLICATED \| \| 892.2 WITH TENDON INVOLVEMENT \| \|  \| \| 893 OPEN WOUND OF TOE(S) \| \| 893.0 WITHOUT MENTION OF COMPLICATION \| \| 893.1 COMPLICATED \| \| 893.2 WITH TENDON INVOLVEMENT \| \|  \| \| ADDITIONAL DIAGNOSTIC CODES \| \| 45A PLANTAR WARTS \| \|  \| \| Bunion \| \| Callous on foot \| \| Corns \| \| Flat feet \| \| Flat foot \| \| Feet concern \| \| Foot abnormality \| \| Foot callous \| \| Foot care \| \| Foor concern \| \| Foot exam \| \| Foot gangrene \| \| Foot infection \| \| Foot issue \| \| Foot lesion \| \| Foot pain \| \| Foot problem \| \| Foot wound \| \| Heel pain \| \| Ingrown toenail \| \| Infected toe \| \| Orthotics \| \| Orthotics form \| \| Plantar fasciitis \| \| Plantar wart \| \| Podiatry \| \| Podiatry referral \| \| Podiatrist \| \| Toe callous \| \| Toe infection \| \| Toenail infection \| \| Toenail fungus \| \| Toenail care \| \| Wound on foot \| \|  \| \| \| --- \| --- \| --- \| --- \| --- \| --- \| --- \| --- \| --- \| --- \| --- \| --- \| --- \| --- \| --- \| --- \| --- \| --- \| --- \| --- \| --- \| --- \| --- \| --- \| --- \| --- \| --- \| --- \| --- \| --- \| --- \| --- \| --- \| --- \| --- \| --- \| --- \| --- \| --- \| --- \| --- \| --- \| --- \| --- \| --- \| --- \| --- \| --- \| --- \| --- \| --- \| --- \| --- \| --- \| --- \| --- \| --- \| --- \| --- \| --- \| --- \| --- \| --- \| --- \| --- \| --- \| --- \| --- \| --- \| --- \| --- \| --- \| --- \| --- \| --- \| --- \| --- \| --- \| --- \| --- \| --- \| --- \| --- \| --- \| --- \| --- \| --- \| --- \| --- \| --- \| --- \| --- \| --- \| --- \| --- \| --- \| --- \| --- \| --- \| --- \| --- \| --- \| --- \| --- \| --- \| --- \| --- \| --- \| --- \| --- \| --- \| --- \| --- \| --- \| | \| **ICD9-CM/TERMWORKS** \| \| --- \| \| **ICD9** \| \| **ICD9-CM** \| \| **ICD9-CM/TERMWORKS** \| \|  \| \| **ICD9-CM/TERMWORKS** \| \| **ICD9-CM/TERMWORKS** \| \| **ICD9-CM/TERMWORKS** \| \| **ICD9-CM/TERMWORKS** \| \| **ICD9-CM/TERMWORKS** \| \| **ICD9-CM/TERMWORKS** \| \| **ICD9-CM/TERMWORKS** \| \|  \| \|  \| \| **ICD9/TERMWORKS** \| \| **ICD9** \| \| **ICD9** \| \| **ICD9** \| \| **ICD9** \| \| **ICD9** \| \| **ICD9** \| \| **ICD9** \| \| **ICD9** \| \| **ICD9** \| \|  \| \| **ICD9/TERMWORKS** \| \| **ICD9-CM/TERMWORKS** \| \| **ICD9-CM** \| \| **ICD9-CM/TERMWORKS** \| \| **ICD9-CM/TERMWORKS** \| \| **ICD9-CM/TERMWORKS** \| \| **ICD9-CM/TERMWORKS** \| \| **ICD9-CM** \| \| **ICD9-CM/TERMWORKS** \| \|  \| \|  \| \| **ICD9/TERMWORKS** \| \|  \| \|  \| \| **ICD9/TERMWORKS** \| \| **ICD9/TERMWORKS** \| \| **ICD9-CM** \| \| **ICD9-CM** \| \| **ICD9/TERMWORKS** \| \|  \| \| **ICD9/TERMWORKS** \| \| **ICD9** \| \| **ICD9** \| \| **ICD9** \| \| **ICD9-CM** \| \|  \| \| **ICD9/TERMWORKS** \| \| **ICD9** \| \| **ICD9-CM** \| \| **ICD9-CM** \| \| **ICD9-CM** \| \| **ICD9-CM** \| \| **ICD9-CM** \| \| **ICD9/TERMWORKS** \| \| **ICD9-CM** \| \| **ICD9-CM** \| \| **ICD9-CM/TERMWORKS** \| \| **ICD9-CM** \| \| **ICD9-CM/TERMWORKS** \| \|  \| \| **TERMWORKS** \| \| **TERMWORKS** \| \| **TERMWORKS** \| \| **TERMWORKS** \| \|  \| \| **ICD9** \| \| **ICD9** \| \| **ICD9** \| \| **ICD9** \| \|  \| \|  \| \| **ICD9** \| \|  \| \| **CPCSSN Free Text** \| \| **CPCSSN Free Text** \| \| **CPCSSN Free Text** \| \| **CPCSSN Free Text** \| \| **CPCSSN Free Text** \| \| **CPCSSN Free Text** \| \| **CPCSSN Free Text** \| \| **CPCSSN Free Text** \| \| **CPCSSN Free Text** \| \| **CPCSSN Free Text** \| \| **CPCSSN Free Text** \| \| **CPCSSN Free Text** \| \| **CPCSSN Free Text** \| \| **CPCSSN Free Text** \| \| **CPCSSN Free Text** \| \| **CPCSSN Free Text** \| \| **CPCSSN Free Text** \| \| **CPCSSN Free Text** \| \| **CPCSSN Free Text** \| \| **CPCSSN Free Text** \| \| **CPCSSN Free Text** \| \| **CPCSSN Free Text** \| \| **CPCSSN Free Text** \| \| **CPCSSN Free Text** \| \| **CPCSSN Free Text** \| \| **CPCSSN Free Text** \| \| **CPCSSN Free Text** \| \| **CPCSSN Free Text** \| \| **CPCSSN Free Text** \| \| **CPCSSN Free Text** \| \| **CPCSSN Free Text** \| \| **CPCSSN Free Text** \| \| **CPCSSN Free Text** \| \| **CPCSSN Free Text** \| |

Termworks search terms: foot, foot $+ problems, chiropody, podiatry, corns, callosities

## Fragility fracture

| **Codes and Terms** | **Source(s)** |
| --- | --- |
| \| \| 733.1 PATHOLOGICAL FRACTURE \| \| --- \| \| 733.10 Pathologic fracture, unspecified site \| \| 733.11 Pathologic fracture of humerus \| \| 733.12 Pathologic fracture of distal radius and ulna \| \| 733.13 Pathologic fracture of vertebrae \| \| 733.14 Pathologic fracture of neck of femur \| \| 733.15 Pathologic fracture of other specified part of femur \| \| 733.16 Pathologic fracture of tibia or fibula \| \| 733.19 Pathologic fracture of other specified site \| \|  \| \| OTHER AND UNSPECIFIED DISORDERS OF BONE AND CARTILAGE \| \| 733.93 Stress fracture of tibia or fibula \| \| 733.94 Stress fracture of the metatarsals \| \| 733.95 Stress fracture of other bone \| \| 733.96 Stress fracture of femoral neck \| \| 733.97 Stress fracture of shaft of femur \| \| 733.98 Stress fracture of pelvis \| \|  \| \| FRACTURE OF SKULL \| \| 800 FRACTURE OF VAULT OF SKULL* \| \| 801 FRACTURE OF BASE OF SKULL* \| \| 802 FRACTURE OF FACE BONES* \| \| 803 OTHER AND UNQUALIFIED SKULL FRACTURES* \| \| 804 MULTIPLE FRACTURES INVOLVING SKULL OR FACE WITH OTHER BONES* \| \|  \| \| FRACTURE OF SPINE AND TRUNK \| \| 805 FRACTURE OF VERTEBRAL COLUMN WITHOUT MENTION OF SPINAL CORD LESION* \| \| 806 FRACTURE OF VERTEBRAL COLUMN WITH SPINAL CORD LESION* \| \| 807 FRACTURE OF RIB(S), STERNUM, LARYNX AND TRACHEA* \| \| 808 FRACTURE OF PELVIS* \| \| 809 ILL-DEFINED FRACTURES OF TRUNK* \| \|  \| \| FRACTURE OF UPPER LIMB* \| \| 810 FRACTURE OF CLAVICLE* \| \| 811 FRACTURE OF SCAPULA* \| \| 812 FRACTURE OF HUMERUS* \| \| 813 FRACTURE OF RADIUS AND ULNA* \| \| 814 FRACTURE OF CARPAL BONE(S)* \| \| 815 FRACTURE OF METACARPAL BONE(S)* \| \| 816 FRACTURE OF ONE OR MORE PHALANGES OF HAND* \| \| 817 MULTIPLE FRACTURES OF HAND BONES* \| \| 818 ILL-DEFINED FRACTURES OF UPPER LIMB* \| \| 819 MULTIPLE FRACTURES INVOLVING BOTH UPPER LIMBS, AND UPPER LIMB* \| \|  \| \| FRACTURE OF LOWER LIMB \| \| 820 FRACTURE OF NECK OF FEMUR* \| \| 821 FRACTURE OF OTHER AND UNSPECIFIED PARTS OF FEMUR* \| \| 822 FRACTURE OF PATELLA* \| \| 823 FRACTURE OF TIBIA AND FIBULA* \| \| 824 FRACTURE OF ANKLE* \| \| 825 FRACTURE OF ONE OR MORE TARSAL AND METATARSAL BONES* \| \| 826 FRACTURE OF ONE OR MORE PHALANGES OF FOOT* \| \| 827 OTHER, MULTIPLE AND ILL-DEFINED FRACTURES OF LOWER LIMB* \| \| 828 MULTIPLE FRACTURES INVOLVING BOTH LOWER LIMBS, LOWER WITH UPPER LIMB, AND LOWER LIMB(S) WITH \| \| RIB(S) AND STERNUM* \| \| 829 FRACTURE OF UNSPECIFIED BONES* \| \|  \| \| ADDITIONAL DIAGNOSTIC CODES \| \| 01X X-RAY \| \|  \| \| V54.0 AFTERCARE INVOLVING REMOVAL OF FRACTURE PLATE OR OTHER INTERNAL FIXATION DEVICE \| \| V54.01 Encounter for removal of internal fixation device \| \| V54.02 Encounter for lengthening/adjustment of growth rod \| \| V54.09 Other aftercare involving internal fixation device \| \| V54.1 Aftercare for healing traumatic fracture \| \| V54.10 Aftercare for healing traumatic fracture of arm, unspecified \| \| V54.11 Aftercare for healing traumatic fracture of upper arm \| \| V54.12 Aftercare for healing traumatic fracture of lower arm \| \| V54.13 Aftercare for healing traumatic fracture of hip \| \| V54.14 Aftercare for healing traumatic fracture of leg, unspecified \| \| V54.15 Aftercare for healing traumatic fracture of upper leg \| \| V54.16 Aftercare for healing traumatic fracture of lower leg \| \| V54.17 Aftercare for healing traumatic fracture of vertebrae \| \| V54.19 Aftercare for healing traumatic fracture of other bone \| \| V54.2 Aftercare for healing pathologic fracture \| \| V54.20 Aftercare for healing pathologic fracture of arm, unspecified \| \| V54.21 Aftercare for healing pathologic fracture of upper arm \| \| V54.22 Aftercare for healing pathologic fracture of lower arm \| \| V54.23 Aftercare for healing pathologic fracture of hip \| \| V54.24 Aftercare for healing pathologic fracture of leg, unspecified \| \| V54.25 Aftercare for healing pathologic fracture of upper leg \| \| V54.26 Aftercare for healing pathologic fracture of lower leg \| \| V54.27 Aftercare for healing pathologic fracture of vertebrae \| \| V54.29 Aftercare for healing pathologic fracture of other bone \| \|  \| \| CONVALESCENCE \| \| V66.4 FOLLOWING TREATMENT OF FRACTURE \| \|  \| \| FOLLOW-UP EXAMINATION \| \| V67.4 FOLLOWING TREATMENT OF FRACTURE \| \|  \| \| V13.51 Personal history of pathologic fracture \| \| V13.52 Personal history of stress fracture \| \| V15.51 Personal history of traumatic fracture \| \|  \| \| Broken \| \| Cast \| \| Fracture \| \| Fractured \| \| Stress fracture \| \| # \| \| \| --- \| --- \| --- \| --- \| --- \| --- \| --- \| --- \| --- \| --- \| --- \| --- \| --- \| --- \| --- \| --- \| --- \| --- \| --- \| --- \| --- \| --- \| --- \| --- \| --- \| --- \| --- \| --- \| --- \| --- \| --- \| --- \| --- \| --- \| --- \| --- \| --- \| --- \| --- \| --- \| --- \| --- \| --- \| --- \| --- \| --- \| --- \| --- \| --- \| --- \| --- \| --- \| --- \| --- \| --- \| --- \| --- \| --- \| --- \| --- \| --- \| --- \| --- \| --- \| --- \| --- \| --- \| --- \| --- \| --- \| --- \| --- \| --- \| --- \| --- \| --- \| --- \| --- \| --- \| --- \| --- \| --- \| --- \| --- \| --- \| --- \| --- \| --- \| --- \| --- \| --- \| --- \| --- \| --- \| --- \| --- \| --- \| --- \| --- \| --- \| --- \| --- \| | \| **ICD9/TERMWORKS** \| \| --- \| \| **ICD9-CM/TERMWORKS** \| \| **ICD9-CM/TERMWORKS** \| \| **ICD9-CM/TERMWORKS** \| \| **ICD9-CM/TERMWORKS** \| \| **ICD9-CM/TERMWORKS** \| \| **ICD9-CM/TERMWORKS** \| \| **ICD9-CM/TERMWORKS** \| \| **ICD9-CM/TERMWORKS** \| \|  \| \| **ICD9-CM** \| \| **ICD9-CM/TERMWORKS** \| \| **ICD9-CM/TERMWORKS** \| \| **ICD9-CM/TERMWORKS** \| \| **ICD9-CM/TERMWORKS** \| \| **ICD9-CM/TERMWORKS** \| \| **ICD9-CM/TERMWORKS** \| \|  \| \|  \| \| **ICD9/TERMWORKS** \| \| **ICD9/TERMWORKS** \| \| **ICD9/TERMWORKS** \| \| **ICD9/TERMWORKS** \| \| **ICD9/TERMWORKS** \| \|  \| \|  \| \| **ICD9/TERMWORKS** \| \| **ICD9/TERMWORKS** \| \| **ICD9/TERMWORKS** \| \| **ICD9/TERMWORKS** \| \| **ICD9/TERMWORKS** \| \|  \| \|  \| \| **ICD9/TERMWORKS** \| \| **ICD9/TERMWORKS** \| \| **ICD9/TERMWORKS** \| \| **ICD9/TERMWORKS** \| \| **ICD9/TERMWORKS** \| \| **ICD9/TERMWORKS** \| \| **ICD9/TERMWORKS** \| \| **ICD9/TERMWORKS** \| \| **ICD9/TERMWORKS** \| \| **ICD9/TERMWORKS** \| \|  \| \|  \| \| **ICD9/TERMWORKS** \| \| **ICD9/TERMWORKS** \| \| **ICD9/TERMWORKS** \| \| **ICD9/TERMWORKS** \| \| **ICD9/TERMWORKS** \| \| **ICD9/TERMWORKS** \| \| **ICD9/TERMWORKS** \| \| **ICD9/TERMWORKS** \| \| **ICD9/TERMWORKS** \| \|  \| \| **ICD9/TERMWORKS** \| \|  \| \|  \| \| **ICD9** \| \|  \| \| **ICD9** \| \| **ICD9-CM** \| \| **ICD9-CM** \| \| **ICD9-CM** \| \| **ICD9-CM/TERMWORKS** \| \| **ICD9-CM/TERMWORKS** \| \| **ICD9-CM/TERMWORKS** \| \| **ICD9-CM/TERMWORKS** \| \| **ICD9-CM/TERMWORKS** \| \| **ICD9-CM/TERMWORKS** \| \| **ICD9-CM/TERMWORKS** \| \| **ICD9-CM/TERMWORKS** \| \| **ICD9-CM/TERMWORKS** \| \| **ICD9-CM/TERMWORKS** \| \| **ICD9-CM/TERMWORKS** \| \| **ICD9-CM/TERMWORKS** \| \| **ICD9-CM/TERMWORKS** \| \| **ICD9-CM/TERMWORKS** \| \| **ICD9-CM/TERMWORKS** \| \| **ICD9-CM/TERMWORKS** \| \| **ICD9-CM/TERMWORKS** \| \| **ICD9-CM/TERMWORKS** \| \| **ICD9-CM/TERMWORKS** \| \| **ICD9-CM/TERMWORKS** \| \|  \| \|  \| \| **TERMWORKS** \| \|  \| \|  \| \| **TERMWORKS** \| \|  \| \| **ICD9-CM/TERMWORKS** \| \| **ICD9-CM/TERMWORKS** \| \| **ICD9-CM/TERMWORKS** \| \|  \| \| **CPCSSN Free Text** \| \| **CPCSSN Free Text** \| \| **CPCSSN Free Text** \| \| **CPCSSN Free Text** \| \| **CPCSSN Free Text** \| \| **CPCSSN Free Text** \| |

Termworks search terms: fracture, fragility $+ fracture

## Hearing impairment

| **Codes and Terms** | **Source(s)** |
| --- | --- |
| \| \| OTHER DISORDERS OF EAR \| \| --- \| \| 388.01 Presbyacusis (Under 388.0 Degenerative and vascular disorders of ear) \| \| 388.12 Noise-induced hearing loss (Under 388.1 Noise effects on inner ear) \| \| 388.2 SUDDEN HEARING LOSS, UNSPECIFIED \| \| 388.4 OTHER ABNORMAL AUDITORY PERCEPTION \| \| 388.41 Diplacusis \| \| 388.5 DISORDERS OF ACOUSTIC NERVE \| \|  \| \| 389 DEAFNESS ('HEARING LOSS" IN ICD9-CM) \| \| 389.0 CONDUCTIVE DEAFNESS \| \| 389.00 Conductive hearing loss, unspecified \| \| 389.01 Conductive hearing loss, external ear \| \| 389.02 Conductive hearing loss, tympanic membrane \| \| 389.03 Conductive hearing loss, middle ear \| \| 389.04 Conductive hearing loss, inner ear \| \| 389.05 Conductive hearing loss, unilateral \| \| 389.06 Conductive hearing loss, bilateral \| \| 389.08 Conductive hearing loss of combined types \| \| 389.1 SENSORINEURAL DEAFNESS \| \| 389.10 Sensorineural hearing loss, unspecified \| \| 389.11 Sensory hearing loss, bilateral \| \| 389.12 Neural hearing loss, bilateral \| \| 389.13 Neural hearing loss, unilateral \| \| 389.14 Central hearing loss \| \| 389.15 Sensorineural hearing loss, unilateral \| \| 389.16 Sensorineural hearing loss, asymmetrical \| \| 389.17 Sensory hearing loss, unilateral \| \| 389.18 Sensorineural hearing loss, bilateral \| \| 389.2 MIXED CONDUCTIVE AND SENSORINEURAL DEAFNESS \| \| 389.20 Mixed hearing loss, unspecified \| \| 389.21 Mixed hearing loss, unilateral \| \| 389.22 Mixed hearing loss, bilateral \| \| 389.7 DEAF MUTISM, NOT ELSEWHERE CLASSIFIABLE \| \| 389.8 OTHER SPECIFIED FORMS OF DEAFNESS/HEARING LOSS \| \| 389.9 UNSPECIFIED DEAFNESS/HEARING LOSS \| \|  \| \| PERSONS WITH CONDITIONS INFLUENCING THEIR HEALTH STATUS \| \| V41.2 PROBLEMS WITH HEARING \| \|  \| \| PERSONS ENCOUNTERING HEALTH SERVICES FOR SPECIFIC PROCEDURES AND AFTER \| \| V53.2 HEARING AID \| \|  \| \| V72.1 EXAMINATION OF EARS AND HEARING \| \| V72.11 Encounter for hearing examination following failed hearing screening \| \| V72.12 Encounter for hearing conservation and treatment \| \| V72.19 Other examination of ears and hearing \| \|  \| \| 794.15 Nonspecific abnormal auditory function studies \| \|  \| \| Audiometry \| \| Can’t hear \| \| Cochlear implant \| \| Deaf \| \| Deafness \| \| Decreased hearing \| \| Hard of hearing \| \| Hearing aid \| \| Hearing decreased \| \| Hearing deficit \| \| Hearing impaired \| \| Hearing impairment \| \| Hearing issue \| \| Hearing loss \| \| Hearing problem \| \| Presbycusis \| \| Reduced hearing \| \| Trouble hearing \| \| \| --- \| --- \| --- \| --- \| --- \| --- \| --- \| --- \| --- \| --- \| --- \| --- \| --- \| --- \| --- \| --- \| --- \| --- \| --- \| --- \| --- \| --- \| --- \| --- \| --- \| --- \| --- \| --- \| --- \| --- \| --- \| --- \| --- \| --- \| --- \| --- \| --- \| --- \| --- \| --- \| --- \| --- \| --- \| --- \| --- \| --- \| --- \| --- \| --- \| --- \| --- \| --- \| --- \| --- \| --- \| --- \| --- \| --- \| --- \| --- \| --- \| --- \| --- \| --- \| --- \| --- \| --- \| --- \| \|  \| | \|  \| \| --- \| \| **ICD9-CM/TERMWORKS** \| \| **ICD9-CM/TERMWORKS** \| \| **ICD9/TERMWORKS** \| \| **ICD9/TERMWORKS** \| \| **ICD9-CM** \| \| **ICD9** \| \|  \| \| **ICD9/TERMWORKS** \| \| **ICD9/TERMWORKS** \| \| **ICD9-CM/TERMWORKS** \| \| **ICD9-CM/TERMWORKS** \| \| **ICD9-CM/TERMWORKS** \| \| **ICD9-CM/TERMWORKS** \| \| **ICD9-CM/TERMWORKS** \| \| **ICD9-CM/TERMWORKS** \| \| **ICD9-CM/TERMWORKS** \| \| **ICD9-CM/TERMWORKS** \| \| **ICD9/TERMWORKS** \| \| **ICD9-CM/TERMWORKS** \| \| **ICD9-CM/TERMWORKS** \| \| **ICD9-CM/TERMWORKS** \| \| **ICD9-CM/TERMWORKS** \| \| **ICD9-CM/TERMWORKS** \| \| **ICD9-CM/TERMWORKS** \| \| **ICD9-CM/TERMWORKS** \| \| **ICD9-CM/TERMWORKS** \| \| **ICD9-CM/TERMWORKS** \| \| **ICD9/TERMWORKS** \| \| **ICD9-CM/TERMWORKS** \| \| **ICD9-CM/TERMWORKS** \| \| **ICD9-CM/TERMWORKS** \| \| **ICD9/TERMWORKS** \| \| **ICD9/TERMWORKS** \| \| **ICD9/TERMWORKS** \| \|  \| \|  \| \| **ICD9/TERMWORKS** \| \|  \| \|  \| \| **ICD9/TERMWORKS** \| \|  \| \| **ICD9/TERMWORKS** \| \| **ICD9-CM/TERMWORKS** \| \| **ICD9-CM/TERMWORKS** \| \| **ICD9-CM/TERMWORKS** \| \|  \| \| **ICD9-CM/TERMWORKS** \| \|  \| \| **CPCSSN Free Text** \| \| **CPCSSN Free Text** \| \| **CPCSSN Free Text** \| \| **CPCSSN Free Text** \| \| **CPCSSN Free Text** \| \| **CPCSSN Free Text** \| \| **CPCSSN Free Text** \| \| **CPCSSN Free Text** \| \| **CPCSSN Free Text** \| \| **CPCSSN Free Text** \| \| **CPCSSN Free Text** \| \| **CPCSSN Free Text** \| \| **CPCSSN Free Text** \| \| **CPCSSN Free Text** \| \| **CPCSSN Free Text** \| \| **CPCSSN Free Text** \| \| **CPCSSN Free Text** \| \| **CPCSSN Free Text** \| |

Termworks search terms: hearing $+ impairment, hearing, deaf, deafness, hearing $+ aid, auditory, Presbyacusis, ototoxicity, auditory, audiometry

## Heart failure

| **Codes and Terms** | **Source(s)** |
| --- | --- |
| \| \| 398.91 Rheumatic heart failure (congestive) \| \| --- \| \|  \| \| 428 HEART FAILURE \| \| 428.0 CONGESTIVE HEART FAILURE \| \| 428.1 LEFT HEART FAILURE \| \| 428.2 Systolic heart failure \| \| 428.20 Systolic heart failure, unspecified \| \| 428.21 Acute systolic heart failure \| \| 428.22 Chronic systolic heart failure \| \| 428.23 Acute on chronic systolic heart failure \| \| 428.3 Diastolic heart failure \| \| 428.30 Diastolic heart failure, unspecified \| \| 428.31 Acute diastolic heart failure \| \| 428.32 Chronic diastolic heart failure \| \| 428.33 Acute on chronic diastolic heart failure \| \| 428.4 Combined systolic and diastolic heart failure \| \| 428.40 Combined systolic and diastolic heart failure, unspecified \| \| 428.41 Acute combined systolic and diastolic heart failure \| \| 428.42 Chronic combined systolic and diastolic heart failure \| \| 428.43 Acute on chronic combined systolic and diastolic heart failure \| \| 428.9 UNSPECIFIED \| \|  \| \| **BNP (B Natriuretic Peptide) ([Mass/volume] in Serum or Plasma)** \| \| HIGHER THAN 400 pg/mL (NIH) \| \|  \| \| CHF \| \| Congestive heart failure \| \| Heart failure \| \|  \| \| \| --- \| --- \| --- \| --- \| --- \| --- \| --- \| --- \| --- \| --- \| --- \| --- \| --- \| --- \| --- \| --- \| --- \| --- \| --- \| --- \| --- \| --- \| --- \| --- \| --- \| --- \| --- \| --- \| --- \| --- \| | \| **ICD9-CM/TERMWORKS** \| \| --- \| \|  \| \| **ICD9/TERMWORKS** \| \| **ICD9/TERMWORKS** \| \| **ICD9/TERMWORKS** \| \| **ICD9-CM/TERMWORKS** \| \| **ICD9-CM/TERMWORKS** \| \| **ICD9-CM/TERMWORKS** \| \| **ICD9-CM/TERMWORKS** \| \| **ICD9-CM/TERMWORKS** \| \| **ICD9-CM/TERMWORKS** \| \| **ICD9-CM/TERMWORKS** \| \| **ICD9-CM/TERMWORKS** \| \| **ICD9-CM/TERMWORKS** \| \| **ICD9-CM/TERMWORKS** \| \| **ICD9-CM/TERMWORKS** \| \| **ICD9-CM/TERMWORKS** \| \| **ICD9-CM/TERMWORKS** \| \| **ICD9-CM/TERMWORKS** \| \| **ICD9-CM/TERMWORKS** \| \| **ICD9/TERMWORKS** \| \|  \| \| **LOINC 30934-4** \| \|  \| \|  \| \| **CPCSSN Free Text** \| \| **CPCSSN Free Text** \| \| **CPCSSN Free Text** \| |

Termworks search terms: heart failure, Paroxysmal nocturnal dyspnea, left ventricular failure

## Heart valve disease

| **Codes and Terms** | **Source(s)** |
| --- | --- |
| \| \| CHRONIC RHEUMATIC HEART DISEASE \| \| --- \| \| 394 DISEASES OF MITRAL VALVE \| \| 394.0 MITRAL STENOSIS \| \| 394.1 RHEUMATIC MITRAL INSUFFICIENCY \| \| 394.2 MITRAL STENOSIS WITH INSUFFICIENCY \| \| 394.9 OTHER AND UNSPECIFIED \| \|  \| \| 395 DISEASES OF AORTIC VALVE \| \| 395.0 RHEUMATIC AORTIC STENOSIS \| \| 395.1 RHEUMATIC AORTIC INSUFFICIENCY \| \| 395.2 RHEUMATIC AORTIC STENOSIS WITH INSUFFICIENCY \| \| 395.9 OTHER AND UNSPECIFIED \| \|  \| \| 396 DISEASES OF MITRAL AND AORTIC VALVES \| \| 396.0 Mitral valve stenosis and aortic valve stenosis \| \| 396.1 Mitral valve stenosis and aortic valve insufficiency \| \| 396.2 Mitral valve insufficiency and aortic valve stenosis \| \| 396.3 Mitral valve insufficiency and aortic valve insufficiency \| \| 396.8 Multiple involvement of mitral and aortic valves \| \| 396.9 Mitral and aortic valve diseases, unspecified \| \|  \| \| 397 DISEASES OF OTHER ENDOCARDIAL STRUCTURES \| \| 397.0 DISEASES OF TRICUSPID VALVE \| \| 397.1 RHEUMATIC DISEASES OF PULMONARY VALVE \| \| 397.9 RHEUMATIC DISEASES OF ENDOCARDIUM, VALVE UNSPECIFIED \| \|  \| \| 424 OTHER DISEASES OF ENDOCARDIUM \| \| 424.0 MITRAL VALVE DISORDERS \| \| 424.1 AORTIC VALVE DISORDERS \| \| 424.2 TRICUSPID VALVE DISORDERS, SPECIFIED AS NONRHEUMATIC \| \| 424.3 PULMONARY VALVE DISORDERS \| \| 424.9 ENDOCARDITIS, VALVE UNSPECIFIED \| \| 424.90 Endocarditis, valve unspecified, unspecified cause \| \| 424.91 Endocarditis in diseases classified elsewhere \| \| 424.99 Other endocarditis, valve unspecified \| \|  \| \| V43.3 HEART VALVE REPLACED BY OTHER MEANS \| \|  \| \| Aortic regurgitation \| \| Aortic stenosis \| \| Mitral valve prolapse \| \| Mitral valve disorder \| \| Mitral regurgitation \| \| Prosthetic aortic valve \| \| Prosthetic mitral valve \| \| Stenosis of aortic valve \| \| Tricuspid regurgitation \| \| Valvular heart disease \| \| Valve disease \| \| Valve disorder \| \| Valve stenosis \| \|  \| \| \| --- \| --- \| --- \| --- \| --- \| --- \| --- \| --- \| --- \| --- \| --- \| --- \| --- \| --- \| --- \| --- \| --- \| --- \| --- \| --- \| --- \| --- \| --- \| --- \| --- \| --- \| --- \| --- \| --- \| --- \| --- \| --- \| --- \| --- \| --- \| --- \| --- \| --- \| --- \| --- \| --- \| --- \| --- \| --- \| --- \| --- \| --- \| --- \| --- \| --- \| --- \| --- \| --- \| | \| **ICD9/TERMWORKS** \| \| --- \| \| **ICD9/TERMWORKS** \| \| **ICD9/TERMWORKS** \| \| **ICD9/TERMWORKS** \| \| **ICD9/TERMWORKS** \| \|  \| \| **ICD9/TERMWORKS** \| \| **ICD9/TERMWORKS** \| \| **ICD9/TERMWORKS** \| \| **ICD9/TERMWORKS** \| \| **ICD9/TERMWORKS** \| \|  \| \| **ICD9/TERMWORKS** \| \| **ICD9-CM/TERMWORKS** \| \| **ICD9-CM/TERMWORKS** \| \| **ICD9-CM/TERMWORKS** \| \| **ICD9-CM/TERMWORKS** \| \| **ICD9-CM/TERMWORKS** \| \| **ICD9-CM/TERMWORKS** \| \|  \| \| **ICD9** \| \| **ICD9/TERMWORKS** \| \| **ICD9/TERMWORKS** \| \| **ICD9/TERMWORKS** \| \|  \| \| **ICD9** \| \| **ICD9/TERMWORKS** \| \| **ICD9/TERMWORKS** \| \| **ICD9/TERMWORKS** \| \| **ICD9/TERMWORKS** \| \| **ICD9/TERMWORKS** \| \| **ICD9-CM/TERMWORKS** \| \| **ICD9-CM** \| \| **ICD9-CM/TERMWORKS** \| \|  \| \| **ICD9/TERMWORKS** \| \|  \| \| **CPCSSN Free Text** \| \| **CPCSSN Free Text** \| \| **CPCSSN Free Text** \| \| **CPCSSN Free Text** \| \| **CPCSSN Free Text** \| \| **CPCSSN Free Text** \| \| **CPCSSN Free Text** \| \| **CPCSSN Free Text** \| \| **CPCSSN Free Text** \| \| **CPCSSN Free Text** \| \| **CPCSSN Free Text** \| \| **CPCSSN Free Text** \| \| **CPCSSN Free Text** \| |

Termworks search terms: valve, stenosis, mitral, aortic

## Housebound

| **Codes and Terms** | **Source(s)** |
| --- | --- |
| \|  \| \| --- \| \| \| V49.84 Bed confinement status \| \| --- \| \|  \| \| Bed bound \| \| Bedridden \| \| Bed ridden \| \| Home and community care \| \| Home and community care referral \| \| Home care \| \| Home care issue \| \| Home care need \| \| Home care referral \| \| Home health \| \| Home support \| \| Home visit \| \| House call \| \| House visit \| \|  \| \| | \| **ICD9-CM** \| \| --- \| \|  \| \| **CPCSSN Free Text** \| \| **CPCSSN Free Text** \| \| **CPCSSN Free Text** \| \| **CPCSSN Free Text** \| \| **CPCSSN Free Text** \| \| **CPCSSN Free Text** \| \| **CPCSSN Free Text** \| \| **CPCSSN Free Text** \| \| **CPCSSN Free Text** \| \| **CPCSSN Free Text** \| \| **CPCSSN Free Text** \| \| **CPCSSN Free Text** \| \| **CPCSSN Free Text** \| \| **CPCSSN Free Text** \| |

Termworks search terms: housebound, home care, home visit, home, domiciliary

## Hypertension

| **Codes and Terms** | **Source(s)** |
| --- | --- |
| \| \| 401 ESSENTIAL HYPERTENSION \| \| --- \| \| 401.0 SPECIFIED AS MALIGNANT (401.0 Malignant essential hypertension) \| \| 401.1 SPECIFIED AS BENIGN (401.1 Benign essential hypertension) \| \| 401.9 NOT SPECIFIED AS MALIGNANT OR BENIGN (401.9 Unspecified essential hypertension) \| \|  \| \| 402 HYPERTENSIVE HEART DISEASE \| \| 402.0 SPECIFIED AS MALIGNANT (402.0 Malignant hypertensive heart disease) \| \| 402.00 Malignant hypertensive heart disease without heart failure \| \| 402.01 Malignant hypertensive heart disease with heart failure \| \| 402.1 SPECIFIED AS BENIGN (402.1 Benign hypertensive heart disease) \| \| 402.10 Benign hypertensive heart disease without heart failure \| \| 402.11 Benign hypertensive heart disease with heart failure \| \| 402.9 NOT SPECIFIED AS MALIGNANT OR BENIGN (402.9 Unspecified hypertensive heart disease) \| \| 402.90 Unspecified hypertensive heart disease without heart failure \| \| 402.91 Unspecified hypertensive heart disease with heart failure \| \|  \| \| 403 HYPERTENSIVE RENAL DISEASE (403 Hypertensive chronic kidney disease) \| \| 403.0 SPECIFIED AS MALIGNANT (403.0 Malignant hypertensive renal disease) \| \| 403.00  With chronic kidney disease stage I through stage IV, or unspecified \| \| 403.01  With chronic kidney disease stage V or end stage renal disease \| \| 403.1 SPECIFIED AS BENIGN (403.1 Benign hypertensive renal disease) \| \| 403.10 With chronic kidney disease stage I through stage IV, or unspecified \| \| 403.11 With chronic kidney disease stage V or end stage renal disease \| \| 403.9 NOT SPECIFIED AS MALIGNANT OR BENIGN (403.9 Unspecified hypertensive renal disease) \| \| 403.90 With chronic kidney disease stage I through stage IV, or unspecified \| \| 403.91 With chronic kidney disease stage V or end stage renal disease \| \|  \| \| 404 HYPERTENSIVE HEART AND RENAL DISEASE (404 Hypertensive heart and chronic kidney disease) \| \| 404.0 SPECIFIED AS MALIGNANT (404.0 Malignant hypertensive heart and renal disease) \| \| 404.00 Without heart failure and with chronic kidney disease stage I through stage IV, or unspecified \| \| 404.01 With heart failure and with chronic kidney disease stage I through stage IV, or unspecified \| \| 404.02 Without heart failure and with chronic kidney disease stage V or end stage renal disease \| \| 404.03 With heart failure and with chronic kidney disease stage V or end stage renal disease \| \| 404.1 SPECIFIED AS BENIGN (404.1 Benign hypertensive heart and renal disease) \| \| 404.10 Without heart failure and with chronic kidney disease stage I through stage IV, or unspecified \| \| 404.11 With heart failure and with chronic kidney disease stage I through stage IV, or unspecified \| \| 404.12 Without heart failure and with chronic kidney disease stage V or end stage renal disease \| \| 404.13 With heart failure and chronic kidney disease stage V or end stage renal disease \| \| 404.9 NOT SPECIFIED AS MALIGNANT OR BENIGN (404.9 Unspecified hypertensive heart and renal disease) \| \| 404.90 Without heart failure and with chronic kidney disease stage I through stage IV, or unspecified \| \| 404.91 With heart failure and with chronic kidney disease stage I through stage IV, or unspecified \| \| 404.92 Without heart failure and with chronic kidney disease stage V or end stage renal disease \| \| 404.93 With heart failure and chronic kidney disease stage V or end stage renal disease \| \|  \| \| 405 SECONDARY HYPERTENSION \| \| 405.0 SPECIFIED AS MALIGNANT (405.0 Malignant secondary hypertension) \| \| 405.01 Malignant renovascular hypertension \| \| 405.09 Other malignant secondary hypertension \| \| 405.1 SPECIFIED AS BENIGN (405.1 Benign secondary hypertension) \| \| 405.11 Benign renovascular hypertension \| \| 405.19 Other benign secondary hypertension \| \| 405.9 NOT SPECIFIED AS MALIGNANT OR BENIGN (405.9 Unspecified secondary hypertension) \| \| 405.91 Unspecified renovascular hypertension \| \| 405.99 Other unspecified secondary hypertension \| \|  \| \| 796.2 ELEVATED BLOOD PRESSURE READING WITHOUT DIAGNOSIS OF HYPERTENSION \| \|  \| \| SPECIAL SCREENING FOR CARDIOVASCULAR, RESPIRATORY AND GENITOURINARY DISEASES \| \| V81.1 HYPERTENSION \| \|  \| \| Blood pressure med \| \| Blood pressure medication \| \| BP rise \| \| Elevated BP \| \| Elevated blood pressure \| \| HTN \| \| HTN Group \| \| HTN GV \| \| Hypertension \| \|  \| \| \| --- \| --- \| --- \| --- \| --- \| --- \| --- \| --- \| --- \| --- \| --- \| --- \| --- \| --- \| --- \| --- \| --- \| --- \| --- \| --- \| --- \| --- \| --- \| --- \| --- \| --- \| --- \| --- \| --- \| --- \| --- \| --- \| --- \| --- \| --- \| --- \| --- \| --- \| --- \| --- \| --- \| --- \| --- \| --- \| --- \| --- \| --- \| --- \| --- \| --- \| --- \| --- \| --- \| --- \| --- \| --- \| --- \| --- \| --- \| --- \| --- \| --- \| --- \| --- \| --- \| --- \| --- \| --- \| --- \| --- \| --- \| | \| **ICD9/TERMWORKS** \| \| --- \| \| **ICD9/TERMWORKS** \| \| **ICD9/TERMWORKS** \| \| **ICD9/TERMWORKS** \| \|  \| \| **ICD9/TERMWORKS** \| \| **ICD9/TERMWORKS** \| \| **ICD9-CM/TERMWORKS** \| \| **ICD9-CM/TERMWORKS** \| \| **ICD9/TERMWORKS** \| \| **ICD9-CM/TERMWORKS** \| \| **ICD9-CM/TERMWORKS** \| \| **ICD9/TERMWORKS** \| \| **ICD9-CM/TERMWORKS** \| \| **ICD9-CM/TERMWORKS** \| \|  \| \| **ICD9/TERMWORKS** \| \| **ICD9/TERMWORKS** \| \| **ICD9-CM/TERMWORKS** \| \| **ICD9-CM/TERMWORKS** \| \| **ICD9/TERMWORKS** \| \| **ICD9-CM/TERMWORKS** \| \| **ICD9-CM/TERMWORKS** \| \| **ICD9/TERMWORKS** \| \| **ICD9-CM/TERMWORKS** \| \| **ICD9-CM/TERMWORKS** \| \|  \| \| **ICD9/TERMWORKS** \| \| **ICD9/TERMWORKS** \| \| **ICD9-CM/TERMWORKS** \| \| **ICD9-CM/TERMWORKS** \| \| **ICD9-CM/TERMWORKS** \| \| **ICD9-CM/TERMWORKS** \| \| **ICD9/TERMWORKS** \| \| **ICD9-CM/TERMWORKS** \| \| **ICD9-CM/TERMWORKS** \| \| **ICD9-CM/TERMWORKS** \| \| **ICD9-CM/TERMWORKS** \| \| **ICD9/TERMWORKS** \| \| **ICD9-CM/TERMWORKS** \| \| **ICD9-CM/TERMWORKS** \| \| **ICD9-CM/TERMWORKS** \| \| **ICD9-CM/TERMWORKS** \| \|  \| \| **ICD9/TERMWORKS** \| \| **ICD9/TERMWORKS** \| \| **ICD9-CM/TERMWORKS** \| \| **ICD9-CM/TERMWORKS** \| \| **ICD9/TERMWORKS** \| \| **ICD9-CM/TERMWORKS** \| \| **ICD9-CM/TERMWORKS** \| \| **ICD9/TERMWORKS** \| \| **ICD9-CM/TERMWORKS** \| \| **ICD9-CM/TERMWORKS** \| \|  \| \| **ICD9/TERMWORKS** \| \|  \| \|  \| \| **ICD9/TERMWORKS** \| \|  \| \| **CPCSSN Free Text** \| \| **CPCSSN Free Text** \| \| **CPCSSN Free Text** \| \| **CPCSSN Free Text** \| \| **CPCSSN Free Text** \| \| **CPCSSN Free Text** \| \| **CPCSSN Free Text** \| \| **CPCSSN Free Text** \| \| **CPCSSN Free Text** \| \|  \| |

Termworks search terms: hypertension, high blood pressure, blood pressure, hypertensive

## Hypotension/syncope

| **Codes and Terms** | **Source(s)** |
| --- | --- |
| \| \| 458 HYPOTENSION \| \| --- \| \| 458.0 ORTHOSTATIC HYPOTENSION \| \| 458.1 CHRONIC HYPOTENSION \| \| 458.2 Iatrogenic hypotension \| \| 458.21 Hypotension of hemodialysis \| \| 458.29 Other iatrogenic hypotension \| \| 458.8 Other specified hypotension \| \| 458.9 UNSPECIFIED \| \|  \| \| NON-SPECIFIC ABNORMAL FINDINGS \| \| 780.2 SYNCOPE AND COLLAPSE \| \| 796.3 NONSPECIFIC LOW BLOOD PRESSURE READING \| \|  \| \| Blood pressure low \| \| BP low \| \| Cardiac syncope \| \| Faint \| \| Fainting \| \| hypotension \| \| hypotension chronic \| \| hypotension orthostatic \| \| hypotension persistent \| \| hypotensive \| \| Hypotensive episode \| \| Lightheaded \| \| Lightheadedness \| \| Low bp \| \| Orthopnea \| \| Orthosis \| \| Orthostasis \| \| Orthostatic \| \| Orthostatic hypotension \| \| Passing out \| \| Postural hypotension \| \| Postural lightheadedness \| \| Presyncope \| \| Pseudo-syncope \| \| Syncopal episode \| \| Syncope \| \| Syncope micturition \| \| Syncope recurrent \| \| Syncope syndrome \| \| Vasovagal episode \| \|  \| \| \| --- \| --- \| --- \| --- \| --- \| --- \| --- \| --- \| --- \| --- \| --- \| --- \| --- \| --- \| --- \| --- \| --- \| --- \| --- \| --- \| --- \| --- \| --- \| --- \| --- \| --- \| --- \| --- \| --- \| --- \| --- \| --- \| --- \| --- \| --- \| --- \| --- \| --- \| --- \| --- \| --- \| --- \| --- \| --- \| --- \| | \| **ICD9/TERMWORKS** \| \| --- \| \| **ICD9/TERMWORKS** \| \| **ICD9/TERMWORKS** \| \| **ICD9-CM/TERMWORKS** \| \| **ICD9-CM/TERMWORKS** \| \| **ICD9-CM/TERMWORKS** \| \| **ICD9-CM/TERMWORKS** \| \| **ICD9/TERMWORKS** \| \|  \| \| **ICD9/TERMWORKS** \| \| **ICD9/TERMWORKS** \| \| **ICD9/TERMWORKS** \| \|  \| \| **CPCSSN Free Text** \| \| **CPCSSN Free Text** \| \| **CPCSSN Free Text** \| \| **CPCSSN Free Text** \| \| **CPCSSN Free Text** \| \| **CPCSSN Free Text** \| \| **CPCSSN Free Text** \| \| **CPCSSN Free Text** \| \| **CPCSSN Free Text** \| \| **CPCSSN Free Text** \| \| **CPCSSN Free Text** \| \| **CPCSSN Free Text** \| \| **CPCSSN Free Text** \| \| **CPCSSN Free Text** \| \| **CPCSSN Free Text** \| \| **CPCSSN Free Text** \| \| **CPCSSN Free Text** \| \| **CPCSSN Free Text** \| \| **CPCSSN Free Text** \| \| **CPCSSN Free Text** \| \| **CPCSSN Free Text** \| \| **CPCSSN Free Text** \| \| **CPCSSN Free Text** \| \| **CPCSSN Free Text** \| \| **CPCSSN Free Text** \| \| **CPCSSN Free Text** \| \| **CPCSSN Free Text** \| \| **CPCSSN Free Text** \| \| **CPCSSN Free Text** \| \| **CPCSSN Free Text** \| \|  \| |

Termworks search terms: hypotension, low blood Pressure, syncope, faint, lightheaded, vasovagal, dizziness

## Ischemic heart disease

| **Codes and Terms** | **Source(s)** |
| --- | --- |
| \| \| ISCHAEMIC HEART DISEASE \| \| --- \| \| 410 ACUTE MYOCARDIAL INFARCTION \| \| 410.0 ACUTE MYOCARDIAL INFARCTION \| \| 410.00 Acute myocardial infarction of anterolateral wall, episode of care unspecified \| \| 410.01 Acute myocardial infarction of anterolateral wall, initial episode of care \| \| 410.02 Acute myocardial infarction of anterolateral wall, subsequent episode of care \| \| 410.1 Acute myocardial infarction of other anterior wall \| \| 410.10 Acute myocardial infarction of other anterior wall, episode of care unspecified \| \| 410.11 Acute myocardial infarction of other anterior wall, initial episode of care \| \| 410.12 Acute myocardial infarction of other anterior wall, subsequent episode of care \| \| 410.2 Acute myocardial infarction of inferolateral wall \| \| 410.20 Acute myocardial infarction of inferolateral wall, episode of care unspecified \| \| 410.21 Acute myocardial infarction of inferolateral wall, initial episode of care \| \| 410.22 Acute myocardial infarction of inferolateral wall, subsequent episode of care \| \| 410.3 Acute myocardial infarction of inferoposterior wall \| \| 410.30 Acute myocardial infarction of inferoposterior wall, episode of care unspecified \| \| 410.31 Acute myocardial infarction of inferoposterior wall, initial episode of care \| \| 410.32 Acute myocardial infarction of inferoposterior wall, subsequent episode of care \| \| 410.4 Acute myocardial infarction of other inferior wall \| \| 410.40 Acute myocardial infarction of other inferior wall, episode of care unspecified \| \| 410.41 Acute myocardial infarction of other inferior wall, initial episode of care \| \| 410.42 Acute myocardial infarction of other inferior wall, subsequent episode of care \| \| 410.5 Acute myocardial infarction of other lateral wall \| \| 410.50 Acute myocardial infarction of other lateral wall, episode of care unspecified \| \| 410.51 Acute myocardial infarction of other lateral wall, initial episode of care \| \| 410.52 Acute myocardial infarction of other lateral wall, subsequent episode of care \| \| 410.6 True posterior wall infarction \| \| 410.60 True posterior wall infarction, episode of care unspecified \| \| 410.61 True posterior wall infarction, initial episode of care \| \| 410.62 True posterior wall infarction, subsequent episode of care \| \| 410.7 Subendocardial infarction \| \| 410.70 Subendocardial infarction, episode of care unspecified \| \| 410.71 Subendocardial infarction, initial episode of care \| \| 410.72 Subendocardial infarction, subsequent episode of care \| \| 410.8 Acute myocardial infarction of other specified sites \| \| 410.80 Acute myocardial infarction of other specified sites, episode of care unspecified \| \| 410.81 Acute myocardial infarction of other specified sites, initial episode of care \| \| 410.82 Acute myocardial infarction of other specified sites, subsequent episode of care \| \| 410.9 Acute myocardial infarction of unspecified site \| \| 410.90 Acute myocardial infarction of unspecified site, episode of care unspecified \| \| 410.91 Acute myocardial infarction of unspecified site, initial episode of care \| \| 410.92 Acute myocardial infarction of unspecified site, subsequent episode of care \| \|  \| \| 411 OTHER ACUTE AND SUBACUTE FORMS OF ISCHAEMIC HEART DISEASE \| \| 411.0 Postmyocardial infarction syndrome \| \| 411.1 Intermediate coronary syndrome \| \| 411.8 Other acute and subacute forms of ischemic heart disease \| \| 411.81 Acute coronary occlusion without myocardial infarction \| \| 411.89 Other acute and subacute forms of ischemic heart disease, other \| \|  \| \| 412 OLD MYOCARDIAL INFARCTION \| \|  \| \| 413 ANGINA PECTORIS \| \| 413.0 Angina decubitus \| \| 413.1 Prinzmetal angina \| \| 413.9 Other and unspecified angina pectoris \| \|  \| \| 414 OTHER FORMS OF CHRONIC ISCHAEMIC HEART DISEASE \| \| 414.0 CORONARY ATHEROSCLEROSIS \| \| 414.00 Coronary atherosclerosis of unspecified type of vessel, native or graft \| \| 414.01 Coronary atherosclerosis of native coronary artery \| \| 414.02 Coronary atherosclerosis of autologous vein bypass graft \| \| 414.03 Coronary atherosclerosis of nonautologous biological bypass graft \| \| 414.04 Coronary atherosclerosis of artery bypass graft \| \| 414.05 Coronary atherosclerosis of unspecified bypass graft \| \| 414.06 Coronary atherosclerosis of native coronary artery of transplanted heart \| \| 414.07 Coronary atherosclerosis of bypass graft (artery) (vein) of transplanted heart \| \| 414.1 ANEURYSM OF HEART \| \| 414.10 Aneurysm of heart (wall) \| \| 414.11 Aneurysm of coronary vessels \| \| 414.12 Dissection of coronary artery \| \| 414.19 Other aneurysm of heart \| \| 414.2 Chronic total occlusion of coronary artery \| \| 414.3 Coronary atherosclerosis due to lipid rich plaque \| \| 414.4 Coronary atherosclerosis due to calcified coronary lesion \| \| 414.8 OTHER \| \| 414.9 UNSPECIFIED \| \|  \| \| 429.2 CARDIOVASCULAR DISEASE, UNSPECIFIED \| \|  \| \| 786.5 CHEST PAIN \| \| 786.50 Chest pain, unspecified \| \| 786.51 Precordial pain \| \| 786.52 Painful respiration \| \| 786.59 Other chest pain \| \|  \| \| V71.7 OBSERVATION FOR SUSPECTED CARDIOVASCULAR DISEASE \| \|  \| \| SPECIAL SCREENING FOR CARDIOVASCULAR, RESPIRATORY AND GENITOURINARY DISEASES \| \| V81.0 ISCHAEMIC HEART DISEASE \| \|  \| \| **Troponin (ng/mL)** \| \| **Troponin T.cardiac** [Mass/volume] in Serum or Plasma: Greater than 0.01 ng/mL \| \| **Troponin I.cardiac** [Mass/volume] in Serum or Plasma: Greater than 0.04 ng/mL \| \|  \| \| Angina \| \| ASHD \| \| Atherosclerotic heart disease \| \| Atypical chest pain \| \| Cardiovascular disease \| \| Chest discomfort \| \| Chest heaviness \| \| Chest pain \| \| Chest pressure \| \| Chest tight \| \| Coronary artery disease \| \| Coronary atherosclerosis \| \| CVD \| \| Heart disease \| \| Heart attack \| \| Ischemic heart disease \| \| Ischaemic heart disease \| \| IHD \| \| Mini heart attack \| \| Myocardial Infarction \| \| NSTEMI \| \| STEMI \| \| Post MI \| \| Recent MI \| \| Tight chest \| \|  \| \| \| --- \| --- \| --- \| --- \| --- \| --- \| --- \| --- \| --- \| --- \| --- \| --- \| --- \| --- \| --- \| --- \| --- \| --- \| --- \| --- \| --- \| --- \| --- \| --- \| --- \| --- \| --- \| --- \| --- \| --- \| --- \| --- \| --- \| --- \| --- \| --- \| --- \| --- \| --- \| --- \| --- \| --- \| --- \| --- \| --- \| --- \| --- \| --- \| --- \| --- \| --- \| --- \| --- \| --- \| --- \| --- \| --- \| --- \| --- \| --- \| --- \| --- \| --- \| --- \| --- \| --- \| --- \| --- \| --- \| --- \| --- \| --- \| --- \| --- \| --- \| --- \| --- \| --- \| --- \| --- \| --- \| --- \| --- \| --- \| --- \| --- \| --- \| --- \| --- \| --- \| --- \| --- \| --- \| --- \| --- \| --- \| --- \| --- \| --- \| --- \| --- \| --- \| --- \| --- \| --- \| --- \| --- \| --- \| --- \| --- \| --- \| --- \| --- \| --- \| --- \| --- \| --- \| --- \| --- \| --- \| --- \| --- \| | \| **ICD9/TERMWORKS** \| \| --- \| \| **ICD9** \| \| **ICD9-CM/TERMWORKS** \| \| **ICD9-CM/TERMWORKS** \| \| **ICD9-CM/TERMWORKS** \| \| **ICD9-CM/TERMWORKS** \| \| **ICD9-CM/TERMWORKS** \| \| **ICD9-CM/TERMWORKS** \| \| **ICD9-CM/TERMWORKS** \| \| **ICD9-CM/TERMWORKS** \| \| **ICD9-CM/TERMWORKS** \| \| **ICD9-CM/TERMWORKS** \| \| **ICD9-CM/TERMWORKS** \| \| **ICD9-CM/TERMWORKS** \| \| **ICD9-CM/TERMWORKS** \| \| **ICD9-CM/TERMWORKS** \| \| **ICD9-CM/TERMWORKS** \| \| **ICD9-CM/TERMWORKS** \| \| **ICD9-CM/TERMWORKS** \| \| **ICD9-CM/TERMWORKS** \| \| **ICD9-CM/TERMWORKS** \| \| **ICD9-CM/TERMWORKS** \| \| **ICD9-CM/TERMWORKS** \| \| **ICD9-CM/TERMWORKS** \| \| **ICD9-CM/TERMWORKS** \| \| **ICD9-CM/TERMWORKS** \| \| **ICD9-CM/TERMWORKS** \| \| **ICD9-CM/TERMWORKS** \| \| **ICD9-CM/TERMWORKS** \| \| **ICD9-CM/TERMWORKS** \| \| **ICD9-CM/TERMWORKS** \| \| **ICD9-CM/TERMWORKS** \| \| **ICD9-CM/TERMWORKS** \| \| **ICD9-CM/TERMWORKS** \| \| **ICD9-CM/TERMWORKS** \| \| **ICD9-CM/TERMWORKS** \| \| **ICD9-CM/TERMWORKS** \| \| **ICD9-CM/TERMWORKS** \| \| **ICD9-CM/TERMWORKS** \| \| **ICD9-CM/TERMWORKS** \| \| **ICD9-CM/TERMWORKS** \| \|  \| \| **ICD9/TERMWORKS** \| \| **ICD9-CM** \| \| **ICD9-CM/TERMWORKS** \| \| **ICD9-CM/TERMWORKS** \| \| **ICD9-CM/TERMWORKS** \| \| **ICD9-CM/TERMWORKS** \| \|  \| \| **ICD9/TERMWORKS** \| \|  \| \| **ICD9/TERMWORKS** \| \| **ICD9-CM/TERMWORKS** \| \| **ICD9-CM/TERMWORKS** \| \| **ICD9-CM/TERMWORKS** \| \|  \| \| **ICD9/TERMWORKS** \| \| **ICD9/TERMWORKS** \| \| **ICD9-CM/TERMWORKS** \| \| **ICD9-CM/TERMWORKS** \| \| **ICD9-CM/TERMWORKS** \| \| **ICD9-CM/TERMWORKS** \| \| **ICD9-CM/TERMWORKS** \| \| **ICD9-CM/TERMWORKS** \| \| **ICD9-CM/TERMWORKS** \| \| **ICD9-CM/TERMWORKS** \| \| **ICD9/TERMWORKS** \| \| **ICD9-CM/TERMWORKS** \| \| **ICD9-CM/TERMWORKS** \| \| **ICD9-CM/TERMWORKS** \| \| **ICD9-CM/TERMWORKS** \| \| **ICD9-CM/TERMWORKS** \| \| **ICD9-CM/TERMWORKS** \| \| **ICD9-CM/TERMWORKS** \| \| **ICD9/TERMWORKS** \| \| **ICD9/TERMWORKS** \| \|  \| \| **ICD9/TERMWORKS** \| \|  \| \| **ICD9/TERMWORKS** \| \| **ICD9-CM/TERMWORKS** \| \| **ICD9-CM** \| \| **ICD9-CM** \| \| **ICD9-CM/TERMWORKS** \| \|  \| \| **ICD9/TERMWORKS** \| \|  \| \|  \| \| **ICD9/TERMWORKS** \| \|  \| \|  \| \| **LOINC 6598-7** \| \| **LOINC 10839-9** \| \|  \| \| **CPCSSN Free Text** \| \| **CPCSSN Free Text** \| \| **CPCSSN Free Text** \| \| **CPCSSN Free Text** \| \| **CPCSSN Free Text** \| \| **CPCSSN Free Text** \| \| **CPCSSN Free Text** \| \| **CPCSSN Free Text** \| \| **CPCSSN Free Text** \| \| **CPCSSN Free Text** \| \| **CPCSSN Free Text** \| \| **CPCSSN Free Text** \| \| **CPCSSN Free Text** \| \| **CPCSSN Free Text** \| \| **CPCSSN Free Text** \| \| **CPCSSN Free Text** \| \| **CPCSSN Free Text** \| \| **CPCSSN Free Text** \| \| **CPCSSN Free Text** \| \| **CPCSSN Free Text** \| \| **CPCSSN Free Text** \| \| **CPCSSN Free Text** \| \| **CPCSSN Free Text** \| \| **CPCSSN Free Text** \| \| **CPCSSN Free Text** \| |

Termworks search terms: cardiovascular, myocardial, myocardial infarct, angina, coronary, ischemic, ischameic, ischaemia, chest pain, heart aneurysm, ischemic heart disease

## Memory and/or cognitive problems

| **Codes and Terms** | **Source(s)** |
| --- | --- |
| \| \| 290 SENILE AND PRESENILE ORGANIC PSYCHOTIC CONDITIONS (ICD9-CM: DEMENTIAS) \| \| --- \| \| 290.0 SENILE DEMENTIA, SIMPLE TYPE \| \| 290.1 PRESENILE DEMENTIA \| \| 290.10 Presenile dementia, uncomplicated \| \| 290.11 Presenile dementia with delirium \| \| 290.12 Presenile dementia with delusional features \| \| 290.13 Presenile dementia with depressive features \| \| 290.2 SENILE DEMENTIA, DEPRESSED OR PARANOID TYPE \| \| 290.20 Senile dementia with delusional features \| \| 290.21 Senile dementia with depressive features \| \| 290.3 SENILE DEMENTIA WITH ACUTE CONFUSIONAL STATE/DELIRIUM \| \| 290.4 ARTERIOSCLEROTIC DEMENTIA (ICD9-CM: VASCULAR DEMENTIA" \| \| 290.40 Vascular dementia, uncomplicated \| \| 290.41 Vascular dementia, with delirium \| \| 290.42 Vascular dementia, with delusions \| \| 290.43 Vascular dementia, with depressed mood \| \| 290.8 OTHER \| \| 290.9 UNSPECIFIED \| \|  \| \| 291.1 KORSAKOV’S PSYCHOSIS, ALCOHOLIC (ICD9-CM: ALCOHOL-INDUCED PERSISTING AMNESTIC DISORDER) \| \| 291.2 OTHER ALCOHOLIC DEMENTIA (ICD9-CM: ALCOHOL-INDUCED PERSISTING DEMENTIA) \| \|  \| \| 293 TRANSIENT ORGANIC PSYCHOTIC CONDITIONS \| \| 293.0 ACUTE CONFUSIONAL STATE \| \| 293.1 SUBACUTE CONFUSIONAL STATE \| \| 293.8 OTHER \| \| 293.9 UNSPECIFIED \| \|  \| \| 294 OTHER ORGANIC PSYCHOTIC CONDITIONS (CHRONIC) (ICD9-CM: PERISTENT MENTAL DISORDERS DUE TO CONDITIONS CLASSIFIED ELSEWHERE) \| \| 294.0 KORSAKOV’S PSYCHOSIS OR SYNDROME (NONALCOHOLIC) (ICD9-CM: AMNESTIC DISORDER IN CONDITIONS CLASSIFIED ELSEWHERE) \| \| 294.1 DEMENTIA IN CONDITIONS CLASSIFIED ELSEWHERE \| \| 294.10 Dementia in conditions classified elsewhere without behavioral disturbance \| \| 294.11 Dementia in conditions classified elsewhere with behavioral disturbance \| \| 294.2 Dementia, unspecified \| \| 294.20 Dementia, unspecified, without behavioral disturbance \| \| 294.21 Dementia, unspecified, with behavioral disturbance \| \| 294.8 OTHER \| \| 294.9 UNSPECIFIED \| \|  \| \| 298.2 REACTIVE CONFUSION (Under "other nonorganic psychoses") \| \|  \| \| HEREDITARY AND DEGENERATIVE DISEASES OF CENTRAL NERVOUS SYSTEM \| \| 331 OTHER CEREBRAL DEGENERATION \| \| 331.0 ALZHEIMER'S DISEASE \| \| 331.1 FRONTOTEMPORAL DEMENTIA \| \| 331.11 Pick's disease \| \| 331.19 Other frontotemporal dementia \| \| 331.2 SENILE DEGENERATION OF BRAIN \| \| 331.7 Cerebral degeneration in diseases classified elsewhere \| \| 331.8 Other cerebral degeneration \| \| 331.81 Reye's syndrome \| \| 331.82 Dementia with lewy bodies \| \| 331.83 Mild cognitive impairment, so stated \| \| 331.8 Other cerebral degeneration \| \| 331.9 Cerebral degeneration, unspecified \| \|  \| \| SYMPTOMS, SIGNS AND ILL-DEFINED CONDITIONS \| \| 797 SENILITY WITHOUT MENTION OF PSYCHOSIS \| \| 780.93 Memory loss \| \| 780.97 Altered mental status \| \|  \| \| Alzheimer’s \| \| Alzheimer’s Disease \| \| Cognition \| \| Cognitive concern \| \| Cognitive deficit \| \| Cognitive decline \| \| Cognitive impairment \| \| Cognitive issue \| \| Cognitive loss \| \| Concern about memory \| \| Confusion \| \| Dementia \| \| Early memory loss \| \| Memory \| \| Memory change \| \| Memory concern \| \| Memory decline \| \| Memory impairment \| \| Memory issues \| \| Memory lapse \| \| Memory loss \| \| Memory loss issue \| \| Memory problem \| \| Mild cognitive impairment \| \| Neurocognitive impairment \| \|  \| \| \| --- \| --- \| --- \| --- \| --- \| --- \| --- \| --- \| --- \| --- \| --- \| --- \| --- \| --- \| --- \| --- \| --- \| --- \| --- \| --- \| --- \| --- \| --- \| --- \| --- \| --- \| --- \| --- \| --- \| --- \| --- \| --- \| --- \| --- \| --- \| --- \| --- \| --- \| --- \| --- \| --- \| --- \| --- \| --- \| --- \| --- \| --- \| --- \| --- \| --- \| --- \| --- \| --- \| --- \| --- \| --- \| --- \| --- \| --- \| --- \| --- \| --- \| --- \| --- \| --- \| --- \| --- \| --- \| --- \| --- \| --- \| --- \| --- \| --- \| --- \| --- \| --- \| --- \| --- \| --- \| --- \| --- \| --- \| --- \| --- \| --- \| --- \| --- \| | \| **ICD9/TERMWORKS** \| \| --- \| \| **ICD9/TERMWORKS** \| \| **ICD9/TERMWORKS** \| \| **ICD9-CM/TERMWORKS** \| \| **ICD9-CM/TERMWORKS** \| \| **ICD9-CM/TERMWORKS** \| \| **ICD9-CM/TERMWORKS** \| \| **ICD9/TERMWORKS** \| \| **ICD9-CM/TERMWORKS** \| \| **ICD9-CM/TERMWORKS** \| \| **ICD9/TERMWORKS** \| \| **ICD9/TERMWORKS** \| \| **ICD9-CM/TERMWORKS** \| \| **ICD9-CM/TERMWORKS** \| \| **ICD9-CM/TERMWORKS** \| \| **ICD9-CM/TERMWORKS** \| \| **ICD9/TERMWORKS** \| \| **ICD9/TERMWORKS** \| \|  \| \| **ICD9/TERMWORKS** \| \| **ICD9/TERMWORKS** \| \|  \| \| **ICD9** \| \| **ICD9** \| \| **ICD9** \| \| **ICD9** \| \| **ICD9** \| \|  \| \| **ICD9** \| \| **ICD9/TERMWORKS** \| \| **ICD9/TERMWORKS** \| \| **ICD9-CM/TERMWORKS** \| \| **ICD9-CM/TERMWORKS** \| \| **ICD9-CM/TERMWORKS** \| \| **ICD9-CM/TERMWORKS** \| \| **ICD9-CM/TERMWORKS** \| \| **ICD9** \| \| **ICD9** \| \|  \| \| **ICD9/TERMWORKS** \| \|  \| \|  \| \| **ICD9/TERMWORKS** \| \| **ICD9/TERMWORKS** \| \| **ICD9/TERMWORKS** \| \| **ICD9-CM** \| \| **ICD9-CM/TERMWORKS** \| \| **ICD9/TERMWORKS** \| \| **ICD9-CM/TERMWORKS** \| \| **ICD9-CM/TERMWORKS** \| \| **ICD9-CM** \| \| **ICD9-CM/TERMWORKS** \| \| **ICD9-CM/TERMWORKS** \| \| **ICD9-CM/TERMWORKS** \| \| **ICD9-CM/TERMWORKS** \| \|  \| \|  \| \| **ICD9/TERMWORKS** \| \| **ICD9-CM/TERMWORKS** \| \| **ICD9-CM** \| \|  \| \| **CPCSSN Free Text** \| \| **CPCSSN Free Text** \| \| **CPCSSN Free Text** \| \| **CPCSSN Free Text** \| \| **CPCSSN Free Text** \| \| **CPCSSN Free Text** \| \| **CPCSSN Free Text** \| \| **CPCSSN Free Text** \| \| **CPCSSN Free Text** \| \| **CPCSSN Free Text** \| \| **CPCSSN Free Text** \| \| **CPCSSN Free Text** \| \| **CPCSSN Free Text** \| \| **CPCSSN Free Text** \| \| **CPCSSN Free Text** \| \| **CPCSSN Free Text** \| \| **CPCSSN Free Text** \| \| **CPCSSN Free Text** \| \| **CPCSSN Free Text** \| \| **CPCSSN Free Text** \| \| **CPCSSN Free Text** \| \| **CPCSSN Free Text** \| \| **CPCSSN Free Text** \| \| **CPCSSN Free Text** \| \| **CPCSSN Free Text** \| |

Termworks search terms: memory, cognitive, confusion, dementia, psychotic, Alzheimer, amnesia, psychosis, cerebral degeneration, confused, lewy body, Binswanger's disease

## Mobility and transfer problems

| **Codes and Terms** | **Source(s)** |
| --- | --- |
| \| \| 344 OTHER PARALYTIC SYNDROMES \| \| --- \| \| 344.0 QUADRIPLEGIA \| \| 344.00 Quadriplegia, unspecified \| \| 344.01 Quadriplegia, C1-C4, complete \| \| 344.02 Quadriplegia, C1-C4, incomplete \| \| 344.03 Quadriplegia, C5-C7, complete \| \| 344.04 Quadriplegia, C5-C7, incomplete \| \| 344.09 Other quadriplegia \| \| 344.1 PARAPLEGIA \| \| 344.2 DIPLEGIA OF UPPER LIMBS \| \| 344.3 MONOPLEGIA OF LOWER LIMB \| \| 344.30 Monoplegia of lower limb affecting unspecified side \| \| 344.31 Monoplegia of lower limb affecting dominant side \| \| 344.32 Monoplegia of lower limb affecting nondominant side \| \| 344.4 MONOPLEGIA OF UPPER LIMB \| \| 344.40 Monoplegia of upper limb affecting unspecified side \| \| 344.41 Monoplegia of upper limb affecting dominant side \| \| 344.42 Monoplegia of upper limb affecting nondominant sde \| \| 344.5 UNSPECIFIED MONOPLEGIA \| \| 344.6 CAUDA EQUINA SYNDROME \| \| 344.60 Cauda equina syndrome without mention of neurogenic bladder \| \| 344.61 Cauda equina syndrome with neurogenic bladder \| \| 344.8 OTHER \| \| 344.81 Locked-in state \| \| 344.89 Other specified paralytic syndrome \| \| 344.9 UNSPECIFIED \| \|  \| \| 780.72 Functional quadriplegia \| \| 781.4 Transient paralysis of limb \| \| 781.2 ABNORMALITY OF GAIT (Under "symptoms involving nervous and muskuloskeletal systems") \| \| 781.3 LACK OF COORDINATION (Under "symptoms involving nervous and muskuloskeletal systems") \| \|  \| \| V46.3 Wheelchair dependence \| \| V52.1 ARTIFICIAL LEG (COMPLETE) (PARTIAL) \| \| V53.8 WHEELCHAIR (ICD9-CM V53.8 Fitting and adjustment of wheelchair) \| \|  \| \| Abnormality of gait \| \| Ataxia \| \| Balance difficulty \| \| Balance issue \| \| Balance problem \| \| Decreased mobility \| \| Difficulty moving \| \| Dysmobility \| \| Gait \| \| Gait abnormal \| \| Gait change \| \| Gait unstable \| \| Gait unsteady \| \| Handidart \| \| Handydart \| \| Hemiplegia \| \| Immobility \| \| Impaired mobility \| \| Mobility concern \| \| Mobility issue \| \| Mobility problem \| \| Paralysis \| \| Paralysis partial \| \| Paraplegia \| \| Poor mobility \| \| Poor balance \| \| Problem with balance \| \| Quadriplegia \| \| Scooter \| \| Unsteady \| \| Unsteady gait \| \| Unsteadiness \| \| Walker \| \| Wheelchair \| \|  \| \| \| --- \| --- \| --- \| --- \| --- \| --- \| --- \| --- \| --- \| --- \| --- \| --- \| --- \| --- \| --- \| --- \| --- \| --- \| --- \| --- \| --- \| --- \| --- \| --- \| --- \| --- \| --- \| --- \| --- \| --- \| --- \| --- \| --- \| --- \| --- \| --- \| --- \| --- \| --- \| --- \| --- \| --- \| --- \| --- \| --- \| --- \| --- \| --- \| --- \| --- \| --- \| --- \| --- \| --- \| --- \| --- \| --- \| --- \| --- \| --- \| --- \| --- \| --- \| --- \| --- \| --- \| --- \| --- \| --- \| --- \| --- \| --- \| | \| **ICD9** \| \| --- \| \| **ICD9** \| \| **ICD9-CM** \| \| **ICD9-CM** \| \| **ICD9-CM** \| \| **ICD9-CM** \| \| **ICD9-CM** \| \| **ICD9-CM** \| \| **ICD9** \| \| **ICD9** \| \| **ICD9** \| \| **ICD9-CM** \| \| **ICD9-CM** \| \| **ICD9-CM** \| \| **ICD9** \| \| **ICD9-CM** \| \| **ICD9-CM** \| \| **ICD9-CM** \| \| **ICD9** \| \| **ICD9** \| \| **ICD9-CM** \| \| **ICD9-CM** \| \| **ICD9** \| \| **ICD9-CM** \| \| **ICD9-CM** \| \| **ICD9** \| \|  \| \| **ICD9-CM** \| \| **ICD9-CM** \| \| **ICD9/TERMWORKS** \| \| **ICD9** \| \|  \| \| **ICD9-CM/TERMWORKS** \| \| **ICD9** \| \| **ICD9/TERMWORKS** \| \|  \| \| **CPCSSN Free Text** \| \| **CPCSSN Free Text** \| \| **CPCSSN Free Text** \| \| **CPCSSN Free Text** \| \| **CPCSSN Free Text** \| \| **CPCSSN Free Text** \| \| **CPCSSN Free Text** \| \| **CPCSSN Free Text** \| \| **CPCSSN Free Text** \| \| **CPCSSN Free Text** \| \| **CPCSSN Free Text** \| \| **CPCSSN Free Text** \| \| **CPCSSN Free Text** \| \| **CPCSSN Free Text** \| \| **CPCSSN Free Text** \| \| **CPCSSN Free Text** \| \| **CPCSSN Free Text** \| \| **CPCSSN Free Text** \| \| **CPCSSN Free Text** \| \| **CPCSSN Free Text** \| \| **CPCSSN Free Text** \| \| **CPCSSN Free Text** \| \| **CPCSSN Free Text** \| \| **CPCSSN Free Text** \| \| **CPCSSN Free Text** \| \| **CPCSSN Free Text** \| \| **CPCSSN Free Text** \| \| **CPCSSN Free Text** \| \| **CPCSSN Free Text** \| \| **CPCSSN Free Text** \| \| **CPCSSN Free Text** \| \| **CPCSSN Free Text** \| \| **CPCSSN Free Text** \| \| **CPCSSN Free Text** \| |

Termworks search terms: mobility, transfer, exercise, walking aid, wheelchair, mobility aid, locomotary, reduced mobility, gait

## Osteoporosis

| **Codes and Terms** | **Source(s)** |
| --- | --- |
| \| \| 733.0 OSTEOPOROSIS \| \| --- \| \| 733.00 Osteoporosis, unspecified \| \| 733.01 Senile osteoporosis \| \| 733.02 Idiopathic osteoporosis \| \| 733.03 Disuse osteoporosis \| \| 733.09 Other osteoporosis \| \|  \| \| V82.81 Special screening for osteoporosis \| \|  \| \| Low bone density \| \| Low bone mass \| \| Osteopenia \| \| Osteoporosis \| \| Osteomalacia \| \|  \| \| \| --- \| --- \| --- \| --- \| --- \| --- \| --- \| --- \| --- \| --- \| --- \| --- \| --- \| --- \| --- \| --- \| | \| **ICD9/TERMWORKS** \| \| --- \| \| **ICD9-CM/TERMWORKS** \| \| **ICD9-CM/TERMWORKS** \| \| **ICD9-CM/TERMWORKS** \| \| **ICD9-CM/TERMWORKS** \| \| **ICD9-CM/TERMWORKS** \| \|  \| \| **ICD9-CM/TERMWORKS** \| \|  \| \| **CPCSSN Free Text** \| \| **CPCSSN Free Text** \| \| **CPCSSN Free Text** \| \| **CPCSSN Free Text** \| \| **CPCSSN Free Text** \| \|  \| |

Termworks search terms: osteoporosis, bone density, osteopenia

## Parkinsonism and Tremor

| **Codes and Terms** | **Source(s)** |
| --- | --- |
| \| \| 332 PARKINSON'S DISEASE \| \| --- \| \| 332.0 PARALYSIS AGITANS \| \| 332.1 SECONDARY PARKINSONISM \| \|  \| \| 333 OTHER EXTRAPYRAMIDAL DISEASE AND ABNORMAL MOVEMENT DISORDERS \| \| 333.1 ESSENTIAL AND OTHER SPECIFIED FORMS OF TREMOR \| \|  \| \| 781.0 ABNORMAL INVOLUNTARY MOVEMENTS \| \|  \| \| **Levodopa-Carbidopa Prescription** \| \|  \| \| Benign essential tremor \| \| Extrapyramidal \| \| Hand tremor \| \| Idiopathic tremor \| \| Idiopathic tremour \| \| Parkinson \| \| Parkinsonism \| \| Tremor \| \| Tremour \| \| Tremulousness \| \|  \| \| \| --- \| --- \| --- \| --- \| --- \| --- \| --- \| --- \| --- \| --- \| --- \| --- \| --- \| --- \| --- \| --- \| --- \| --- \| --- \| --- \| --- \| --- \| --- \| | \| **ICD9/TERMWORKS** \| \| --- \| \| **ICD9/TERMWORKS** \| \| **ICD9** \| \|  \| \| **ICD9/TERMWORKS** \| \| **ICD9/TERMWORKS** \| \|  \| \| **ICD9** \| \|  \| \|  \| \|  \| \| **CPCSSN Free Text** \| \| **CPCSSN Free Text** \| \| **CPCSSN Free Text** \| \| **CPCSSN Free Text** \| \| **CPCSSN Free Text** \| \| **CPCSSN Free Text** \| \| **CPCSSN Free Text** \| \| **CPCSSN Free Text** \| \| **CPCSSN Free Text** \| \| **CPCSSN Free Text** \| |

Termworks search terms: Parkinson, Parkinsonian, tremor, extrapyramidal, paralysis agitans, parkinsonism

## Peptic Ulcer

| **Codes and Terms** | **Source(s)** |
| --- | --- |
| \| \| 531 GASTRIC ULCER \| \| --- \| \| 531.0 ACUTE WITH HAEMORRHAGE \| \| 531.00 Acute gastric ulcer with hemorrhage, without mention of obstruction \| \| 531.01 Acute gastric ulcer with hemorrhage, with obstruction \| \| 531.1 ACUTE WITH PERFORATION \| \| 531.10 Acute gastric ulcer with perforation, without mention of obstruction \| \| 531.11 Acute gastric ulcer with perforation, with obstruction \| \| 531.2 ACUTE WITH HAEMORRHAGE AND PERFORATION \| \| 531.20 Acute gastric ulcer with hemorrhage and perforation, without mention of obstruction \| \| 531.21 Acute gastric ulcer with hemorrhage and perforation, with obstruction \| \| 531.3 ACUTE WITHOUT MENTION OF HAEMORRHAGE OR PERFORATION \| \| 531.30 Acute gastric ulcer without mention of hemorrhage or perforation, without mention of obstruction \| \| 531.31 Acute gastric ulcer without mention of hemorrhage or perforation, with obstruction \| \| 531.4 CHRONIC OR UNSPECIFIED WITH HAEMORRHAGE \| \| 531.40 Chronic or unspecified gastric ulcer with hemorrhage, without mention of obstruction \| \| 531.41 Chronic or unspecified gastric ulcer with hemorrhage, with obstruction \| \| 531.5 CHRONIC OR UNSPECIFIED WITH PERFORATION \| \| 531.50 Chronic or unspecified gastric ulcer with perforation, without mention of obstruction \| \| 531.51 Chronic or unspecified gastric ulcer with perforation, with obstruction \| \| 531.6 CHRONIC OR UNSPECIFIED WITH HAEMORRHAGE AND PERFORATION \| \| 531.60 Chronic or unspecified gastric ulcer with hemorrhage and perforation, without mention of obstruction \| \| 531.61 Chronic or unspecified gastric ulcer with hemorrhage and perforation, with obstruction \| \| 531.7 CHRONIC WITHOUT MENTION OF HAEMORRHAGE OR PERFORATION \| \| 531.70 Chronic gastric ulcer without mention of hemorrhage or perforation, without mention of obstruction \| \| 531.71 Chronic gastric ulcer without mention of hemorrhage or perforation, with obstruction \| \| 531.9 UNSPECIFIED AS ACUTE OR CHRONIC, WITHOUT MENTION OF HAEMORRHAGE OR PERFORATION \| \| 531.90 Gastric ulcer, unspecified as acute or chronic, without mention of hemorrhage or perforation, without mention of obstruction \| \| 531.91 Gastric ulcer, unspecified as acute or chronic, without mention of hemorrhage or perforation, with obstruction \| \|  \| \| 532 DUODENAL ULCER \| \| 532.0 ACUTE WITH HAEMORRHAGE \| \| 532.00 Acute duodenal ulcer with hemorrhage, without mention of obstruction \| \| 532.01 Acute duodenal ulcer with hemorrhage, with obstruction \| \| 532.1 ACUTE WITH PERFORATION \| \| 532.10 Acute duodenal ulcer with perforation, without mention of obstruction \| \| 532.11 Acute duodenal ulcer with perforation, with obstruction \| \| 532.2 ACUTE WITH HAEMORRHAGE AND PERFORATION \| \| 532.20 Acute duodenal ulcer with hemorrhage and perforation, without mention of obstruction \| \| 532.21 Acute duodenal ulcer with hemorrhage and perforation, with obstruction \| \| 532.3 ACUTE WITHOUT MENTION OF HAEMORRHAGE OR PERFORATION \| \| 532.30 Acute duodenal ulcer without mention of hemorrhage or perforation, without mention of obstruction \| \| 532.31 Acute duodenal ulcer without mention of hemorrhage or perforation, with obstruction \| \| 532.4 CHRONIC OR UNSPECIFIED WITH HAEMORRHAGE \| \| 532.40 Chronic or unspecified duodenal ulcer with hemorrhage, without mention of obstruction \| \| 532.41 Chronic or unspecified duodenal ulcer with hemorrhage, with obstruction \| \| 532.5 CHRONIC OR UNSPECIFIED WITH PERFORATION \| \| 532.50 Chronic or unspecified duodenal ulcer with perforation, without mention of obstruction \| \| 532.51 Chronic or unspecified duodenal ulcer with perforation, with obstruction \| \| 532.6 CHRONIC OR UNSPECIFIED WITH HAEMORRHAGE AND PERFORATION \| \| 532.60 Chronic or unspecified duodenal ulcer with hemorrhage and perforation, without mention of obstruction \| \| 532.61 Chronic or unspecified duodenal ulcer with hemorrhage and perforation, with obstruction \| \| 532.7 CHRONIC WITHOUT MENTION OF HAEMORRHAGE OR PERFORATION \| \| 532.70 Chronic duodenal ulcer without mention of hemorrhage or perforation, without mention of obstruction \| \| 532.71 Chronic duodenal ulcer without mention of hemorrhage or perforation, with obstruction \| \| 532.9 UNSPECIFIED AS ACUTE OR CHRONIC, WITHOUT MENTION OF HAEMORRHAGE OR PERFORATION \| \| 532.90 Duodenal ulcer, unspecified as acute or chronic, without hemorrhage or perforation, without mention of obstruction \| \| 532.91 Duodenal ulcer, unspecified as acute or chronic, without mention of hemorrhage or perforation, with obstruction \| \|  \| \| 533 PEPTIC ULCER, SITE UNSPECIFIED \| \| 533.0 ACUTE WITH HAEMORRHAGE \| \| 533.00 Acute peptic ulcer of unspecified site with hemorrhage, without mention of obstruction \| \| 533.01 Acute peptic ulcer of unspecified site with hemorrhage, with obstruction \| \| 533.1 ACUTE WITH PERFORATION \| \| 533.10 Acute peptic ulcer of unspecified site with perforation, without mention of obstruction \| \| 533.11 Acute peptic ulcer of unspecified site with perforation, with obstruction \| \| 533.2 ACUTE WITH HAEMORRHAGE AND PERFORATION \| \| 533.20 Acute peptic ulcer of unspecified site with hemorrhage and perforation, without mention of obstruction \| \| 533.21 Acute peptic ulcer of unspecified site with hemorrhage and perforation, with obstruction \| \| 533.3 ACUTE WITHOUT MENTION OF HAEMORRHAGE OR PERFORATION \| \| 533.30 Acute peptic ulcer of unspecified site without mention of hemorrhage and perforation, without mention of obstruction \| \| 533.31 Acute peptic ulcer of unspecified site without mention of hemorrhage and perforation, with obstruction \| \| 533.4 CHRONIC OR UNSPECIFIED WITH HAEMORRHAGE \| \| 533.40 Chronic or unspecified peptic ulcer of unspecified site with hemorrhage, without mention of obstruction \| \| 533.41 Chronic or unspecified peptic ulcer of unspecified site with hemorrhage, with obstruction \| \| 533.5 CHRONIC OR UNSPECIFIED WITH PERFORATION \| \| 533.50 Chronic or unspecified peptic ulcer of unspecified site with perforation, without mention of obstruction \| \| 533.51 Chronic or unspecified peptic ulcer of unspecified site with perforation, with obstruction \| \| 533.6 CHRONIC OR UNSPECIFIED WITH HAEMORRHAGE AND PERFORATION \| \| 533.60 Chronic or unspecified peptic ulcer of unspecified site with hemorrhage and perforation, without mention of obstruction \| \| 533.61 Chronic or unspecified peptic ulcer of unspecified site with hemorrhage and perforation, with obstruction \| \| 533.7 CHRONIC WITHOUT MENTION OF HAEMORRHAGE OR PERFORATION \| \| 533.70 Chronic peptic ulcer of unspecified site without mention of hemorrhage or perforation, without mention of obstruction \| \| 533.71 Chronic peptic ulcer of unspecified site without mention of hemorrhage or perforation, with obstruction \| \| 533.9 UNSPECIFIED AS ACUTE OR CHRONIC, WITHOUT MENTION OF HAEMORRHAGE OR PERFORATION \| \| 533.90 Peptic ulcer of unspecified site, unspecified as acute or chronic, without mention of hemorrhage or perforation, without mention of obstruction \| \| 533.91 Peptic ulcer of unspecified site, unspecified as acute or chronic, without mention of hemorrhage or perforation, with obstruction \| \|  \| \| 534 GASTROJEJUNAL ULCER \| \| 534.0 ACUTE WITH HAEMORRHAGE \| \| 534.00 Acute gastrojejunal ulcer with hemorrhage, without mention of obstruction \| \| 534.01 Acute gastrojejunal ulcer, with hemorrhage, with obstruction \| \| 534.1 ACUTE WITH PERFORATION \| \| 534.10 Acute gastrojejunal ulcer with perforation, without mention of obstruction \| \| 534.11 Acute gastrojejunal ulcer with perforation, with obstruction \| \| 534.2 ACUTE WITH HAEMORRHAGE AND PERFORATION \| \| 534.20 Acute gastrojejunal ulcer with hemorrhage and perforation, without mention of obstruction \| \| 534.21 Acute gastrojejunal ulcer with hemorrhage and perforation, with obstruction \| \| 534.3 ACUTE WITHOUT MENTION OF HAEMORRHAGE OR PERFORATION \| \| 534.30 Acute gastrojejunal ulcer without mention of hemorrhage or perforation, without mention of obstruction \| \| 534.31 Acute gastrojejunal ulcer without mention of hemorrhage or perforation, with obstruction \| \| 534.4 CHRONIC OR UNSPECIFIED WITH HAEMORRHAGE \| \| 534.40 Chronic or unspecified gastrojejunal ulcer with hemorrhage, without mention of obstruction \| \| 534.41 Chronic or unspecified gastrojejunal ulcer, with hemorrhage, with obstruction \| \| 534.5 CHRONIC OR UNSPECIFIED WITH PERFORATION \| \| 534.50 Chronic or unspecified gastrojejunal ulcer with perforation, without mention of obstruction \| \| 534.51 Chronic or unspecified gastrojejunal ulcer with perforation, with obstruction \| \| 534.6 CHRONIC OR UNSPECIFIED WITH HAEMORRHAGE AND PERFORATION \| \| 534.60 Chronic or unspecified gastrojejunal ulcer with hemorrhage and perforation, without mention of obstruction \| \| 534.61 Chronic or unspecified gastrojejunal ulcer with hemorrhage and perforation, with obstruction \| \| 534.7 CHRONIC WITHOUT MENTION OF HAEMORRHAGE OR PERFORATION \| \| 534.70 Chronic gastrojejunal ulcer without mention of hemorrhage or perforation, without mention of obstruction \| \| 534.71 Chronic gastrojejunal ulcer without mention of hemorrhage or perforation, with obstruction \| \| 534.9 UNSPECIFIED AS ACUTE OR CHRONIC, WITHOUT MENTION OF HAEMORRHAGE OR PERFORATION \| \| 534.90 Gastrojejunal ulcer, unspecified as acute or chronic, without mention of hemorrhage or perforation, without mention of obstruction \| \| 534.91 Gastrojejunal ulcer, unspecified as acute or chronic, without mention of hemorrhage or perforation, with obstruction \| \|  \| \| 578 GASTROINTESTINAL HAEMORRHAGE \| \| 578.0 Hematemesis \| \| 578.1 Blood in stool \| \| 578.9 Hemorrhage of gastrointestinal tract, unspecified \| \|  \| \| V12.71 Personal history of peptic ulcer disease \| \| 041.86 Helicobacter pylori [H. pylori] \| \|  \| \| **H.Pylori** \| \| H. Pylori (Helicobacter pylori IgG Ab [Presence] in Serum or Plasma by Immunoassay) \| \| Helicobacter pylori Ag [Presence] in Stool by Immunoassay \| \| Helicobacter pylori Ag [Presence] in Stool \| \|  \| \| Blood in stool \| \| Bloody stool \| \| Colon ulcer \| \| Duodenal ulcer \| \| Esophageal ulcer \| \| Gastric ulcer \| \| Gastrointestinal bleed \| \| Gastrointestinal haemorrhage \| \| Gastrointestinal hemorrhage \| \| GI bleed \| \| H pylori \| \| H. pylori \| \| Helicobacter pylori \| \| Occult blood \| \| Peptic ulcer \| \| Peptic ulcer disease \| \| PUD \| \| Stool heme positive \| \|  \| \| \| --- \| --- \| --- \| --- \| --- \| --- \| --- \| --- \| --- \| --- \| --- \| --- \| --- \| --- \| --- \| --- \| --- \| --- \| --- \| --- \| --- \| --- \| --- \| --- \| --- \| --- \| --- \| --- \| --- \| --- \| --- \| --- \| --- \| --- \| --- \| --- \| --- \| --- \| --- \| --- \| --- \| --- \| --- \| --- \| --- \| --- \| --- \| --- \| --- \| --- \| --- \| --- \| --- \| --- \| --- \| --- \| --- \| --- \| --- \| --- \| --- \| --- \| --- \| --- \| --- \| --- \| --- \| --- \| --- \| --- \| --- \| --- \| --- \| --- \| --- \| --- \| --- \| --- \| --- \| --- \| --- \| --- \| --- \| --- \| --- \| --- \| --- \| --- \| --- \| --- \| --- \| --- \| --- \| --- \| --- \| --- \| --- \| --- \| --- \| --- \| --- \| --- \| --- \| --- \| --- \| --- \| --- \| --- \| --- \| --- \| --- \| --- \| --- \| --- \| --- \| --- \| --- \| --- \| --- \| --- \| --- \| --- \| --- \| --- \| --- \| --- \| --- \| --- \| --- \| --- \| --- \| --- \| --- \| --- \| --- \| --- \| --- \| --- \| --- \| --- \| --- \| --- \| --- \| --- \| --- \| --- \| --- \| --- \| --- \| | \| **ICD9/TERMWORKS** \| \| --- \| \| **ICD9** \| \| **ICD9-CM/TERMWORKS** \| \| **ICD9-CM/TERMWORKS** \| \| **ICD9** \| \| **ICD9-CM/TERMWORKS** \| \| **ICD9-CM/TERMWORKS** \| \| **ICD9** \| \| **ICD9-CM/TERMWORKS** \| \| **ICD9-CM/TERMWORKS** \| \| **ICD9** \| \| **ICD9-CM/TERMWORKS** \| \| **ICD9-CM/TERMWORKS** \| \| **ICD9** \| \| **ICD9-CM/TERMWORKS** \| \| **ICD9-CM/TERMWORKS** \| \| **ICD9** \| \| **ICD9-CM/TERMWORKS** \| \| **ICD9-CM/TERMWORKS** \| \| **ICD9** \| \| **ICD9-CM/TERMWORKS** \| \| **ICD9-CM/TERMWORKS** \| \| **ICD9** \| \| **ICD9-CM/TERMWORKS** \| \| **ICD9-CM/TERMWORKS** \| \| **ICD9** \| \| **ICD9-CM/TERMWORKS** \| \| **ICD9-CM/TERMWORKS** \| \|  \| \| **ICD9/TERMWORKS** \| \| **ICD9** \| \| **ICD9-CM/TERMWORKS** \| \| **ICD9-CM/TERMWORKS** \| \| **ICD9** \| \| **ICD9-CM/TERMWORKS** \| \| **ICD9-CM/TERMWORKS** \| \| **ICD9** \| \| **ICD9-CM/TERMWORKS** \| \| **ICD9-CM/TERMWORKS** \| \| **ICD9** \| \| **ICD9-CM/TERMWORKS** \| \| **ICD9-CM/TERMWORKS** \| \| **ICD9** \| \| **ICD9-CM/TERMWORKS** \| \| **ICD9-CM/TERMWORKS** \| \| **ICD9** \| \| **ICD9-CM/TERMWORKS** \| \| **ICD9-CM/TERMWORKS** \| \| **ICD9** \| \| **ICD9-CM/TERMWORKS** \| \| **ICD9-CM/TERMWORKS** \| \| **ICD9** \| \| **ICD9-CM/TERMWORKS** \| \| **ICD9-CM/TERMWORKS** \| \| **ICD9** \| \| **ICD9-CM/TERMWORKS** \| \| **ICD9-CM/TERMWORKS** \| \|  \| \| **ICD9/TERMWORKS** \| \| **ICD9** \| \| **ICD9-CM/TERMWORKS** \| \| **ICD9-CM/TERMWORKS** \| \| **ICD9** \| \| **ICD9-CM/TERMWORKS** \| \| **ICD9-CM/TERMWORKS** \| \| **ICD9** \| \| **ICD9-CM/TERMWORKS** \| \| **ICD9-CM/TERMWORKS** \| \| **ICD9** \| \| **ICD9-CM/TERMWORKS** \| \| **ICD9-CM/TERMWORKS** \| \| **ICD9** \| \| **ICD9-CM/TERMWORKS** \| \| **ICD9-CM/TERMWORKS** \| \| **ICD9** \| \| **ICD9-CM/TERMWORKS** \| \| **ICD9-CM/TERMWORKS** \| \| **ICD9** \| \| **ICD9-CM/TERMWORKS** \| \| **ICD9-CM/TERMWORKS** \| \| **ICD9** \| \| **ICD9-CM/TERMWORKS** \| \| **ICD9-CM/TERMWORKS** \| \| **ICD9** \| \| **ICD9-CM/TERMWORKS** \| \| **ICD9-CM/TERMWORKS** \| \|  \| \| **ICD9/TERMWORKS** \| \| **ICD9** \| \| **ICD9-CM/TERMWORKS** \| \| **ICD9-CM/TERMWORKS** \| \| **ICD9** \| \| **ICD9-CM/TERMWORKS** \| \| **ICD9-CM/TERMWORKS** \| \| **ICD9** \| \| **ICD9-CM/TERMWORKS** \| \| **ICD9-CM/TERMWORKS** \| \| **ICD9** \| \| **ICD9-CM/TERMWORKS** \| \| **ICD9-CM/TERMWORKS** \| \| **ICD9** \| \| **ICD9-CM/TERMWORKS** \| \| **ICD9-CM/TERMWORKS** \| \| **ICD9** \| \| **ICD9-CM/TERMWORKS** \| \| **ICD9-CM/TERMWORKS** \| \| **ICD9** \| \| **ICD9-CM/TERMWORKS** \| \| **ICD9-CM/TERMWORKS** \| \| **ICD9** \| \| **ICD9-CM/TERMWORKS** \| \| **ICD9-CM/TERMWORKS** \| \| **ICD9** \| \| **ICD9-CM/TERMWORKS** \| \| **ICD9-CM/TERMWORKS** \| \|  \| \| **ICD9** \| \| **ICD9-CM** \| \| **ICD9-CM** \| \| **ICD9-CM** \| \|  \| \| **ICD9-CM/TERMWORKS** \| \| **ICD9-CM** \| \|  \| \|  \| \| **LOINC 17859-0** \| \| **LOINC 17780-8** \| \| **LOINC 31843-6** \| \|  \| \| **CPCSSN Free Text** \| \| **CPCSSN Free Text** \| \| **CPCSSN Free Text** \| \| **CPCSSN Free Text** \| \| **CPCSSN Free Text** \| \| **CPCSSN Free Text** \| \| **CPCSSN Free Text** \| \| **CPCSSN Free Text** \| \| **CPCSSN Free Text** \| \| **CPCSSN Free Text** \| \| **CPCSSN Free Text** \| \| **CPCSSN Free Text** \| \| **CPCSSN Free Text** \| \| **CPCSSN Free Text** \| \| **CPCSSN Free Text** \| \| **CPCSSN Free Text** \| \| **CPCSSN Free Text** \| \| **CPCSSN Free Text** \| |

Termworks search terms: ulcer

## Peripheral Vascular Disease

| **Codes and Terms** | **Source(s)** |
| --- | --- |
| \| \| 440 ATHEROSCLEROSIS \| \| --- \| \| 440.0 OF AORTA \| \| 440.1 OF RENAL ARTERY \| \| 440.2 OF ARTERIES OF THE EXTREMITIES \| \| 440.20 Atherosclerosis of native arteries of the extremities, unspecified \| \| 440.21 Atherosclerosis of native arteries of the extremities with intermittent claudication \| \| 440.22 Atherosclerosis of native arteries of the extremities with rest pain \| \| 440.23 Atherosclerosis of native arteries of the extremities with ulceration \| \| 440.24 Atherosclerosis of native arteries of the extremities with gangrene \| \| 440.29 Other atherosclerosis of native arteries of the extremities \| \| 440.3 Atherosclerosis of bypass graft of the extremities \| \| 440.30 Atherosclerosis of unspecified bypass graft of the extremities \| \| 440.31 Atherosclerosis of autologous vein bypass graft of the extremities \| \| 440.32 Atherosclerosis of nonautologous biological bypass graft of the extremities \| \| 440.4 Chronic total occlusion of artery of the extremities \| \| 440.8 OF OTHER SPECIFIED ARTERIES \| \| 440.9 GENERALIZED AND UNSPECIFIED \| \|  \| \| 443 OTHER PERIPHERAL VASCULAR DISEASE \| \| 443.0 RAYNAUD'S SYNDROME \| \| 443.1 THROMBOANGIITIS OBLITERANS (BUERGER'S DISEASE) \| \| 443.2 Other arterial dissection \| \| 443.21 Dissection of carotid artery \| \| 443.22 Dissection of iliac artery \| \| 443.23 Dissection of renal artery \| \| 443.24 Dissection of vertebral artery \| \| 443.29 Dissection of other artery \| \| 443.8 OTHER \| \| 443.81 Peripheral angiopathy in diseases classified elsewhere \| \| 443.82 Erythromelalgia \| \| 443.89 Other specified peripheral vascular diseases \| \| 443.9 UNSPECIFIED \| \|  \| \| 444 ARTERIAL EMBOLISM AND THROMBOSIS \| \| 444.2 OF ARTERIES OF THE EXTREMITIES \| \| 444.21 Arterial embolism and thrombosis of upper extremity \| \| 444.22 Arterial embolism and thrombosis of lower extremity \| \|  \| \| 445 Atheroembolism \| \| 445.0 Atheroembolism of extremities \| \| 445.01 Atheroembolism of upper extremity \| \| 445.02 Atheroembolism of lower extremity \| \| 445.8 Atheroembolism of other sites \| \| 445.81 Atheroembolism of kidney \| \| 445.89 Atheroembolism of other site \| \|  \| \| 451 PHLEBITIS AND THROMBOPHLEBITIS \| \| 451.0 OF SUPERFICIAL VESSELS OF LOWER EXTREMITIES \| \| 451.1 OF DEEP VESSELS OF LOWER EXTREMITIES \| \| 451.2 OF LOWER EXTREMITIES, UNSPECIFIED \| \| 451.8 OF OTHER SITES \| \| 451.9 OF UNSPECIFIED SITE \| \|  \| \| 453 OTHER VENOUS EMBOLISM AND THROMBOSIS \| \| 453.4 Acute venous embolism and thrombosis of deep vessels of lower extremity \| \| 453.40 Acute venous embolism and thrombosis of unspecified deep vessels of lower extremity \| \| 453.41 Acute venous embolism and thrombosis of deep vessels of proximal lower extremity \| \| 453.42 Acute venous embolism and thrombosis of deep vessels of distal lower extremity \| \| 453.5 Chronic venous embolism and thrombosis of deep vessels of lower extremity \| \| 453.50 Chronic venous embolism and thrombosis of unspecified deep vessels of lower extremity \| \| 453.51 Chronic venous embolism and thrombosis of deep vessels of proximal lower extremity \| \| 453.52 Chronic venous embolism and thrombosis of deep vessels of distal lower extremity \| \| 453.6 Venous embolism and thrombosis of superficial vessels of lower extremity \| \| 453.7 Chronic venous embolism and thrombosis of other specified vessels \| \| 453.71 Chronic venous embolism and thrombosis of superficial veins of upper extremity \| \| 453.72 Chronic venous embolism and thrombosis of deep veins of upper extremity \| \| 453.73 Chronic venous embolism and thrombosis of upper extremity, unspecified \| \| 453.74 Chronic venous embolism and thrombosis of axillary veins \| \| 453.75 Chronic venous embolism and thrombosis of subclavian veins \| \| 453.76 Chronic venous embolism and thrombosis of internal jugular veins \| \| 453.77 Chronic venous embolism and thrombosis of other thoracic veins \| \| 453.79 Chronic venous embolism and thrombosis of other specified veins \| \| 453.8 Acute venous embolism and thrombosis of other specified veins \| \| 453.81 Acute venous embolism and thrombosis of superficial veins of upper extremity \| \| 453.82 Acute venous embolism and thrombosis of deep veins of upper extremity \| \| 453.83 Acute venous embolism and thrombosis of upper extremity, unspecified \| \| 453.84 Acute venous embolism and thrombosis of axillary veins \| \| 453.85 Acute venous embolism and thrombosis of subclavian veins \| \| 453.86 Acute venous embolism and thrombosis of internal jugular veins \| \| 453.87 Acute venous embolism and thrombosis of other thoracic veins \| \| 453.89 Acute venous embolism and thrombosis of other specified veins \| \| 453.9 Other venous embolism and thrombosis of unspecified site \| \|  \| \| 997.2 PERIPHERAL VASCULAR COMPLICATIONS \| \|  \| \| V12.51 Personal history of venous thrombosis and embolism \| \| V12.52 Personal history of thrombophlebitis \| \|  \| \| Arteriosclerosis \| \| Arterial disease \| \| Arterial insufficiency \| \| Atherosclerosis \| \| Blood clot \| \| Deep vein thrombosis \| \| DVT \| \| Embolism \| \| Lower extremity arterial insufficiency \| \| Lower extremity thrombophlebitis \| \| Lower extremity venous insufficiency \| \| Lower extremity venous stasis \| \| Lower leg venous stasis \| \| Peripheral vascular disease \| \| PVD \| \| Thromboembolic disease \| \| Thrombosis \| \| Vascular deficiency \| \| Vascular disorder \| \| Vascular insufficiency \| \| Venous stasis \| \| Venous thrombosis \| \|  \| \| \| --- \| --- \| --- \| --- \| --- \| --- \| --- \| --- \| --- \| --- \| --- \| --- \| --- \| --- \| --- \| --- \| --- \| --- \| --- \| --- \| --- \| --- \| --- \| --- \| --- \| --- \| --- \| --- \| --- \| --- \| --- \| --- \| --- \| --- \| --- \| --- \| --- \| --- \| --- \| --- \| --- \| --- \| --- \| --- \| --- \| --- \| --- \| --- \| --- \| --- \| --- \| --- \| --- \| --- \| --- \| --- \| --- \| --- \| --- \| --- \| --- \| --- \| --- \| --- \| --- \| --- \| --- \| --- \| --- \| --- \| --- \| --- \| --- \| --- \| --- \| --- \| --- \| --- \| --- \| --- \| --- \| --- \| --- \| --- \| --- \| --- \| --- \| --- \| --- \| --- \| --- \| --- \| --- \| --- \| --- \| --- \| --- \| --- \| --- \| --- \| --- \| --- \| --- \| --- \| --- \| --- \| --- \| --- \| --- \| --- \| --- \| --- \| | \| **ICD9/TERMWORKS** \| \| --- \| \| **ICD9/TERMWORKS** \| \| **ICD9/TERMWORKS** \| \| **ICD9/TERMWORKS** \| \| **ICD9-CM/TERMWORKS** \| \| **ICD9-CM/TERMWORKS** \| \| **ICD9-CM/TERMWORKS** \| \| **ICD9-CM/TERMWORKS** \| \| **ICD9-CM/TERMWORKS** \| \| **ICD9-CM/TERMWORKS** \| \| **ICD9-CM/TERMWORKS** \| \| **ICD9-CM/TERMWORKS** \| \| **ICD9-CM/TERMWORKS** \| \| **ICD9-CM/TERMWORKS** \| \| **ICD9-CM/TERMWORKS** \| \| **ICD9/TERMWORKS** \| \| **ICD9/TERMWORKS** \| \|  \| \| **ICD9/TERMWORKS** \| \| **ICD9** \| \| **ICD9** \| \| **ICD9-CM** \| \| **ICD9-CM** \| \| **ICD9-CM** \| \| **ICD9-CM** \| \| **ICD9-CM** \| \| **ICD9-CM** \| \| **ICD9/TERMWORKS** \| \| **ICD9-CM/TERMWORKS** \| \| **ICD9-CM** \| \| **ICD9-CM/TERMWORKS** \| \| **ICD9/TERMWORKS** \| \|  \| \| **TERMWORKS** \| \| **TERMWORKS** \| \| **ICD9-CM/TERMWORKS** \| \| **ICD9-CM/TERMWORKS** \| \|  \| \| **ICD9-CM** \| \| **ICD9-CM/TERMWORKS** \| \| **ICD9-CM** \| \| **ICD9-CM** \| \| **ICD9-CM** \| \| **ICD9-CM** \| \| **ICD9-CM** \| \|  \| \| **ICD9/TERMWORKS** \| \| **ICD9/TERMWORKS** \| \| **ICD9/TERMWORKS** \| \| **ICD9/TERMWORKS** \| \| **ICD9/TERMWORKS** \| \| **ICD9/TERMWORKS** \| \|  \| \| **ICD9/TERMWORKS** \| \| **ICD9-CM/TERMWORKS** \| \| **ICD9-CM/TERMWORKS** \| \| **ICD9-CM/TERMWORKS** \| \| **ICD9-CM/TERMWORKS** \| \| **ICD9-CM/TERMWORKS** \| \| **ICD9-CM/TERMWORKS** \| \| **ICD9-CM/TERMWORKS** \| \| **ICD9-CM/TERMWORKS** \| \| **ICD9-CM/TERMWORKS** \| \| **ICD9-CM/TERMWORKS** \| \| **ICD9-CM/TERMWORKS** \| \| **ICD9-CM/TERMWORKS** \| \| **ICD9-CM/TERMWORKS** \| \| **ICD9-CM/TERMWORKS** \| \| **ICD9-CM/TERMWORKS** \| \| **ICD9-CM/TERMWORKS** \| \| **ICD9-CM/TERMWORKS** \| \| **ICD9-CM/TERMWORKS** \| \| **ICD9-CM/TERMWORKS** \| \| **ICD9-CM/TERMWORKS** \| \| **ICD9-CM/TERMWORKS** \| \| **ICD9-CM/TERMWORKS** \| \| **ICD9-CM/TERMWORKS** \| \| **ICD9-CM/TERMWORKS** \| \| **ICD9-CM/TERMWORKS** \| \| **ICD9-CM/TERMWORKS** \| \| **ICD9-CM/TERMWORKS** \| \| **ICD9-CM/TERMWORKS** \| \|  \| \| **ICD9/TERMWORKS** \| \|  \| \| **ICD9-CM/TERMWORKS** \| \| **ICD9-CM** \| \|  \| \| **CPCSSN Free Text** \| \| **CPCSSN Free Text** \| \| **CPCSSN Free Text** \| \| **CPCSSN Free Text** \| \| **CPCSSN Free Text** \| \| **CPCSSN Free Text** \| \| **CPCSSN Free Text** \| \| **CPCSSN Free Text** \| \| **CPCSSN Free Text** \| \| **CPCSSN Free Text** \| \| **CPCSSN Free Text** \| \| **CPCSSN Free Text** \| \| **CPCSSN Free Text** \| \| **CPCSSN Free Text** \| \| **CPCSSN Free Text** \| \| **CPCSSN Free Text** \| \| **CPCSSN Free Text** \| \| **CPCSSN Free Text** \| \| **CPCSSN Free Text** \| \| **CPCSSN Free Text** \| \| **CPCSSN Free Text** \| \| **CPCSSN Free Text** \| |

Termworks search terms: peripheral vascular, pulse, dorsalis pedis, gangrene, atherosclerosis, thrombosis, extremities

## Polypharmacy

| **Codes and Terms** | **Source(s)** |
| --- | --- |
| \| *Defined if patient is taking 5 or more medications*  Polypharmacy \| \| --- \| | **CPCSSN Free Text** |

Termworks search terms: polypharmacy, medications

## Requirement for care

| **Codes and Terms** | **Source(s)** |
| --- | --- |
| \| \| PERSONS ENCOUNTERING HEALTH SERVICES IN OTHER CIRCUMSTANCES \| \| --- \| \| V60.4 NO OTHER HOUSEHOLD MEMBER ABLE TO RENDER CARE \| \| V60.5 HOLIDAY RELIEF CARE \| \| V60.6 PERSON LIVING IN RESIDENTIAL INSTITUTION \| \|  \| \| V63 UNAVAILABILITY OF OTHER MEDICAL FACILITIES FOR CARE \| \| V63.1 MEDICAL SERVICES IN HOME NOT AVAILABLE \| \| V63.2 PERSON AWAITING ADMISSION TO ADEQUATE FACILITY ELSEWHERE \| \| V63.8 OTHER SPECIFIED REASONS FOR UNAVAILABILITY OF MEDICAL FACILITIES \| \| V63.9 UNSPECIFIED REASON FOR UNAVAILABILITY OF MEDICAL FACILITIES \| \|  \| \| V66 CONVALESCENCE AND PALLIATIVE CARE \| \| V66.7 Encounter for palliative care \| \| V66.9 Unspecified convalescence \| \|  \| \| ADLs \| \| Banking \| \| Errands \| \| Grocery \| \| Groceries \| \| IADLs \| \| Lifeline \| \| No help \| \| Needs help \| \| Palliative care \| \| \| --- \| --- \| --- \| --- \| --- \| --- \| --- \| --- \| --- \| --- \| --- \| --- \| --- \| --- \| --- \| --- \| --- \| --- \| --- \| --- \| --- \| --- \| --- \| --- \| --- \| --- \| | \| **ICD9/TERMWORKS** \| \| --- \| \| **ICD9/TERMWORKS** \| \| **ICD9/TERMWORKS** \| \|  \| \| **ICD9/TERMWORKS** \| \| **ICD9/TERMWORKS** \| \| **ICD9/TERMWORKS** \| \| **ICD9/TERMWORKS** \| \| **ICD9/TERMWORKS** \| \|  \| \| **TERMWORKS** \| \| **ICD9-CM** \| \| **ICD9-CM** \| \|  \| \| **CPCSSN Free Text** \| \| **CPCSSN Free Text** \| \| **CPCSSN Free Text** \| \| **CPCSSN Free Text** \| \| **CPCSSN Free Text** \| \| **CPCSSN Free Text** \| \| **CPCSSN Free Text** \| \| **CPCSSN Free Text** \| \| **CPCSSN Free Text** \| \| **CPCSSN Free Text** \| \|  \| |

Termworks search terms: care requirement, nursing home, care home, help, residential, facility, carer

## Respiratory Disease

| **Codes and Terms** | **Source(s)** |
| --- | --- |
| \| \| DISEASES OF PULMONARY CIRCULATION \| \| --- \| \| 415.1 PULMONARY EMBOLISM and infarction \| \| 415.11 Iatrogenic pulmonary embolism and infarction \| \| 415.12 Septic pulmonary embolism \| \| 415.13 Saddle embolus of pulmonary artery \| \| 415.19 Other pulmonary embolism and infarction \| \|  \| \| 416 CHRONIC PULMONARY HEART DISEASE \| \| 416.0 PRIMARY PULMONARY HYPERTENSION \| \| 416.1 Kyphoscoliotic heart disease \| \| 416.2 Chronic pulmonary embolism \| \| 416.8 Other chronic pulmonary heart diseases \| \| 416.9 Chronic pulmonary heart disease, unspecified \| \|  \| \| CHRONIC OBSTRUCTIVE PULMONARY DISEASE AND ALLIED CONDITIONS \| \| 490 BRONCHITIS, NOT SPECIFIED AS ACUTE OR CHRONIC \| \| 491 CHRONIC BRONCHITIS \| \| 491.0 SIMPLE CHRONIC BRONCHITIS \| \| 491.1 MUCOPURULENT CHRONIC BRONCHITIS \| \| 491.2 OBSTRUCTIVE CHRONIC BRONCHITIS \| \| 491.20 Obstructive chronic bronchitis without exacerbation \| \| 491.21 Obstructive chronic bronchitis with (acute) exacerbation \| \| 491.22 Obstructive chronic bronchitis with acute bronchitis \| \| 491.8 OTHER CHRONIC BRONCHITIS \| \| 491.9 UNSPECIFIED \| \|  \| \| 492 EMPHYSEMA \| \| 492.0 Emphysematous bleb \| \| 492.8 Other emphysema \| \|  \| \| 493 ASTHMA \| \| 493.0 EXTRINSIC ASTHMA \| \| 493.00 Extrinsic asthma, unspecified \| \| 493.01 Extrinsic asthma with status asthmaticus \| \| 493.02 Extrinsic asthma with (acute) exacerbation \| \| 493.1 INTRINSIC ASTHMA \| \| 493.10 Intrinsic asthma, unspecified \| \| 493.11 Intrinsic asthma with status asthmaticus \| \| 493.12 Intrinsic asthma with (acute) exacerbation \| \| 493.2 Chronic obstructive asthma \| \| 493.20 Chronic obstructive asthma, unspecified \| \| 493.21 Chronic obstructive asthma with status asthmaticus \| \| 493.22 Chronic obstructive asthma with (acute) exacerbation \| \| 493.8 Other forms of asthma \| \| 493.81 Exercise induced bronchospasm \| \| 493.82 Cough variant asthma \| \| 493.9 ASTHMA, UNSPECIFIED \| \| 493.90 Asthma,unspecified type, unspecified \| \| 493.91 Asthma, unspecified type, with status asthmaticus \| \| 493.92 Asthma, unspecified type, with (acute) exacerbation \| \|  \| \| 494 BRONCHIECTASIS \| \| 494.0 Bronchiectasis without acute exacerbation \| \| 494.1 Bronchiectasis with acute exacerbation \| \|  \| \| 495 EXTRINSIC ALLERGIC ALVEOLITIS \| \| 495.0 FARMERS' LUNG \| \| 495.1 BAGASSOSIS \| \| 495.2 BIRD FANCIERS' LUNG \| \| 495.3 SUBEROSIS \| \| 495.4 MALTWORKERS' LUNG \| \| 495.5 MUSHROOM-WORKERS' LUNG \| \| 495.6 MAPLE-BARK-STRIPPERS' LUNG \| \| 495.7 "VENTILATION" PNEUMONITIS \| \| 495.8 OTHER ALLERGIC PNEUMONITIS \| \| 495.9 UNSPECIFIED ALLERGIC ALVEOLITIS \| \|  \| \| 496 CHRONIC AIRWAYS OBSTRUCTION, NOT ELSEWHERE CLASSIFIED \| \|  \| \| PNEUMOCONISOSES AND OTHER LUNG DISEASES DUE TO EXTERNAL AGENTS \| \| 500 COALWORKERS' PNEUMOCONIOSIS \| \| 501 ASBESTOSIS \| \| 502 PNEUMOCONIOSIS DUE TO OTHER SILICA OR SILICATES \| \| 503 PNEUMOCONIOSIS DUE TO OTHER INORGANIC DUST \| \| 504 PNEUMOPATHY DUE TO INHALATION OF OTHER DUST \| \| 505 PNEUMOCONIOSIS, UNSPECIFIED \| \|  \| \| 506.4 CHRONIC RESPIRATORY CONDITIONS DUE TO FUMES AND VAPOURS \| \|  \| \| 508.1 CHRONIC AND OTHER PULMONARY MANIFESTATIONS DUE TO RADIATION \| \|  \| \| OTHER DISEASES OF RESPIRATORY SYSTEM \| \| 514 PULMONARY CONGESTION AND HYPOSTASIS \| \| 515 POSTINFLAMMATORY PULMONARY FIBROSIS \| \| 516 OTHER ALVEOLAR AND PARIETOALVEOLAR PNEUMOPATHY \| \|  \| \| Other Diseases of Lung \| \| 518.1 INTERSTITIAL EMPHYSEMA \| \| 518.2 COMPENSATORY EMPHYSEMA \| \| 518.83 Chronic respiratory failure \| \|  \| \| 519 OTHER DISEASES OF RESPIRATORY SYSTEM \| \| 519.0 TRACHEOSTOMY MALFUNCTION/COMPLICATIONS (ICD9-CM) \| \| 519.00 Tracheostomy complication, unspecified \| \| 519.01 Infection of tracheostomy \| \| 519.02 Mechanical complication of tracheostomy \| \| 519.09 Other tracheostomy complications \| \| 519.1 OTHER DISEASES OF TRACHEA AND BRONCHUS, NOT ELSEWHERE CLASSIFIED \| \| 519.2 MEDIASTINITIS \| \| 519.3 OTHER DISEASES OF MEDIASTINUM, NOT ELSEWHERE CLASSIFIED \| \| 519.4 DISORDERS OF DIAPHRAGM \| \| 519.8 OTHER DISEASES OF RESPIRATORY SYSTEM, NOT ELSEWHERE CLASSIFIED \| \| 519.9 UNSPECIFIED \| \|  \| \| V12.6 DISEASES OF RESPIRATORY SYSTEM (under "personal history of certain other diseases") \| \| V12.60 Personal history of unspecified disease of respiratory system \| \| V12.61 Personal history of pneumonia (recurrent) \| \| V12.69 Personal history of other diseases of respiratory system \| \|  \| \| V12.55 Personal history of pulmonary embolism \| \| V15.82 Personal history of tobacco use \| \| V15.84 Personal history of contact with and (suspected) exposure to asbestos \| \| V44.0 Tracheostomy status \| \| V46.2 Other dependence on machines, supplemental oxygen \| \| V55.0 Attention to tracheostomy \| \|  \| \| SPECIAL SCREENING FOR CARDIOVASCULAR, RESPIRATORY AND GENITOURINARY DISEASES \| \| V81.3 CHRONIC BRONCHITIS AND EMPHYSEMA \| \|  \| \| Asthma \| \| Bronchiectasis \| \| Bronchitis \| \| Chronic airway obstruction \| \| Chronic cough \| \| Chronic lung disease \| \| Chronic respiratory condition \| \| Consistent cough \| \| COPD \| \| Cough chronic \| \| CPAP \| \| Emphysema \| \| Home 02 \| \| Home oxygen \| \| Inhaler \| \| Interstitial lung disease \| \| Low oxygen \| \| Lung disease \| \| Nebulizer \| \| O2 saturation low \| \| Oxygen saturation low \| \| Ongoing cough \| \| Persistent cough \| \| Pulmonary edema \| \| Pulmonary embolism \| \| Pulmonary heart disease \| \| Respiratory difficulty \| \| Respiratory distress \| \| Spirometry \| \|  \| \| \| --- \| --- \| --- \| --- \| --- \| --- \| --- \| --- \| --- \| --- \| --- \| --- \| --- \| --- \| --- \| --- \| --- \| --- \| --- \| --- \| --- \| --- \| --- \| --- \| --- \| --- \| --- \| --- \| --- \| --- \| --- \| --- \| --- \| --- \| --- \| --- \| --- \| --- \| --- \| --- \| --- \| --- \| --- \| --- \| --- \| --- \| --- \| --- \| --- \| --- \| --- \| --- \| --- \| --- \| --- \| --- \| --- \| --- \| --- \| --- \| --- \| --- \| --- \| --- \| --- \| --- \| --- \| --- \| --- \| --- \| --- \| --- \| --- \| --- \| --- \| --- \| --- \| --- \| --- \| --- \| --- \| --- \| --- \| --- \| --- \| --- \| --- \| --- \| --- \| --- \| --- \| --- \| --- \| --- \| --- \| --- \| --- \| --- \| --- \| --- \| --- \| --- \| --- \| --- \| --- \| --- \| --- \| --- \| --- \| --- \| --- \| --- \| --- \| --- \| --- \| --- \| --- \| --- \| --- \| --- \| --- \| --- \| --- \| --- \| --- \| --- \| --- \| --- \| --- \| --- \| --- \| --- \| --- \| --- \| --- \| --- \| --- \| --- \| --- \| --- \| --- \| --- \| --- \| --- \| --- \| --- \| --- \| --- \| --- \| --- \| | \| **ICD9/TERMWORKS** \| \| --- \| \| **ICD9-CM/TERMWORKS** \| \| **ICD9-CM/TERMWORKS** \| \| **ICD9-CM/TERMWORKS** \| \| **ICD9-CM/TERMWORKS** \| \|  \| \| **ICD9/TERMWORKS** \| \| **ICD9/TERMWORKS** \| \| **ICD9-CM** \| \| **ICD9-CM/TERMWORKS** \| \| **ICD9-CM/TERMWORKS** \| \| **ICD9-CM/TERMWORKS** \| \|  \| \|  \| \| **ICD9/TERMWORKS** \| \| **ICD9/TERMWORKS** \| \| **ICD9/TERMWORKS** \| \| **ICD9/TERMWORKS** \| \| **ICD9/TERMWORKS** \| \| **ICD9-CM/TERMWORKS** \| \| **ICD9-CM/TERMWORKS** \| \| **ICD9-CM/TERMWORKS** \| \| **ICD9/TERMWORKS** \| \| **ICD9/TERMWORKS** \| \|  \| \| **ICD9/TERMWORKS** \| \| **ICD9-CM** \| \| **ICD9-CM/TERMWORKS** \| \|  \| \| **ICD9/TERMWORKS** \| \| **ICD9/TERMWORKS** \| \| **ICD9-CM/TERMWORKS** \| \| **ICD9-CM/TERMWORKS** \| \| **ICD9-CM/TERMWORKS** \| \| **ICD9/TERMWORKS** \| \| **ICD9-CM/TERMWORKS** \| \| **ICD9-CM/TERMWORKS** \| \| **ICD9-CM/TERMWORKS** \| \| **ICD9-CM/TERMWORKS** \| \| **ICD9-CM/TERMWORKS** \| \| **ICD9-CM/TERMWORKS** \| \| **ICD9-CM/TERMWORKS** \| \| **ICD9-CM/TERMWORKS** \| \| **ICD9-CM** \| \| **ICD9-CM/TERMWORKS** \| \| **ICD9/TERMWORKS** \| \| **ICD9-CM/TERMWORKS** \| \| **ICD9-CM/TERMWORKS** \| \| **ICD9-CM/TERMWORKS** \| \|  \| \| **ICD9** \| \| **ICD9-CM** \| \| **ICD9-CM** \| \|  \| \| **ICD9** \| \| **ICD9/TERMWORKS** \| \| **ICD9** \| \| **ICD9/TERMWORKS** \| \| **ICD9** \| \| **ICD9/TERMWORKS** \| \| **ICD9/TERMWORKS** \| \| **ICD9/TERMWORKS** \| \| **ICD9** \| \| **ICD9** \| \| **ICD9** \| \|  \| \| **ICD9/TERMWORKS** \| \|  \| \| **ICD9** \| \| **ICD9** \| \| **ICD9** \| \| **ICD9** \| \| **ICD9** \| \| **ICD9** \| \| **ICD9** \| \|  \| \| **ICD9/TERMWORKS** \| \|  \| \| **ICD9/TERMWORKS** \| \|  \| \|  \| \| **ICD9/TERMWORKS** \| \| **ICD9/TERMWORKS** \| \| **ICD9** \| \|  \| \|  \| \| **ICD9/TERMWORKS** \| \| **ICD9/TERMWORKS** \| \| **ICD9-CM/TERMWORKS** \| \|  \| \| **ICD9/TERMWORKS** \| \| **ICD9** \| \| **ICD9-CM** \| \| **ICD9-CM** \| \| **ICD9-CM** \| \| **ICD9-CM** \| \| **ICD9** \| \| **ICD9** \| \| **ICD9** \| \| **ICD9** \| \| **ICD9/TERMWORKS** \| \| **ICD9/TERMWORKS** \| \|  \| \| **ICD9/TERMWORKS** \| \| **ICD9-CM/TERMWORKS** \| \| **ICD9-CM** \| \| **ICD9-CM/TERMWORKS** \| \|  \| \| **ICD9-CM/TERMWORKS** \| \| **ICD9-CM** \| \| **ICD9-CM** \| \| **ICD9-CM** \| \| **ICD9-CM/TERMWORKS** \| \| **ICD9-CM** \| \|  \| \|  \| \| **ICD9/TERMWORKS** \| \|  \| \| **CPCSSN Free Text** \| \| **CPCSSN Free Text** \| \| **CPCSSN Free Text** \| \| **CPCSSN Free Text** \| \| **CPCSSN Free Text** \| \| **CPCSSN Free Text** \| \| **CPCSSN Free Text** \| \| **CPCSSN Free Text** \| \| **CPCSSN Free Text** \| \| **CPCSSN Free Text** \| \| **CPCSSN Free Text** \| \| **CPCSSN Free Text** \| \| **CPCSSN Free Text** \| \| **CPCSSN Free Text** \| \| **CPCSSN Free Text** \| \| **CPCSSN Free Text** \| \| **CPCSSN Free Text** \| \| **CPCSSN Free Text** \| \| **CPCSSN Free Text** \| \| **CPCSSN Free Text** \| \| **CPCSSN Free Text** \| \| **CPCSSN Free Text** \| \| **CPCSSN Free Text** \| \| **CPCSSN Free Text** \| \| **CPCSSN Free Text** \| \| **CPCSSN Free Text** \| \| **CPCSSN Free Text** \| \| **CPCSSN Free Text** \| \| **CPCSSN Free Text** \| |

Termworks search terms: respiratory, chronic obstructive pulmonary disease, COPD, asthma, cough, breathing, respiratory disease, airway, pulmonary, oxygen, nebulizer, nebuliser, spacer, inhaler, bronchitis, wheezing, emphysema, spirometry, airway obstruction

## Skin Ulcer

| **Codes and Terms** | **Source(s)** |
| --- | --- |
| \| \| DISEASES OF VEINS AND LYMPHATICS, AND OTHER DISEASEAS OF CIRCULATORY SYSTEM \| \| --- \| \| 454 VARICOSE VEINS OF LOWER EXTREMITIES \| \| 454.0 WITH ULCER \| \| 454.2 WITH ULCER AND INFLAMMATION \| \|  \| \| 707 CHRONIC ULCER OF SKIN \| \| 707.0 DECUBITUS ULCER (ICD9-CM: "PRESSURE ULCER") \| \| 707.00 Pressure ulcer, unspecified site \| \| 707.01 Pressure ulcer, elbow \| \| 707.02 Pressure ulcer, upper back \| \| 707.03 Pressure ulcer, lower back \| \| 707.04 Pressure ulcer, hip \| \| 707.05 Pressure ulcer, buttock \| \| 707.06 Pressure ulcer, ankle \| \| 707.07 Pressure ulcer, heel \| \| 707.09 Pressure ulcer, other site \| \| 707.1 ULCER OF LOWER LIMBS, EXCEPT DECUBITUS \| \| 707.10 Ulcer of lower limb, unspecified \| \| 707.11 Ulcer of thigh \| \| 707.12 Ulcer of calf \| \| 707.13 Ulcer of ankle \| \| 707.14 Ulcer of heel and midfoot \| \| 707.15 Ulcer of other part of foot \| \| 707.19 Ulcer of other part of lower limb \| \| 707.2 Pressure ulcer stages \| \| 707.20 Pressure ulcer, unspecified stage \| \| 707.21 Pressure ulcer, stage I \| \| 707.22 Pressure ulcer, stage II \| \| 707.23 Pressure ulcer, stage III \| \| 707.24 Pressure ulcer, stage IV \| \| 707.25 Pressure ulcer, unstageable \| \| 707.8 CHRONIC ULCER OF OTHER SPECIFIED SITES \| \| 707.9 CHRONIC ULCER OF UNSPECIFIED SITE \| \|  \| \| V58.3 Attention to dressings and sutures \| \| V58.30 Encounter for change or removal of nonsurgical wound dressing \| \|  \| \| Bedsore \| \| Chronic skin ulcer \| \| Chronic ulcer \| \| Decubitus ulcer \| \| Open wound \| \| Poor wound healing \| \| Pressure sore \| \| Pressure ulcer \| \| Pressure wound \| \| Skin eruption \| \| Skin ulcer \| \| Ulcer decubitus \| \| Wound care \| \| Wound chronic \| \|  \| \| \| --- \| --- \| --- \| --- \| --- \| --- \| --- \| --- \| --- \| --- \| --- \| --- \| --- \| --- \| --- \| --- \| --- \| --- \| --- \| --- \| --- \| --- \| --- \| --- \| --- \| --- \| --- \| --- \| --- \| --- \| --- \| --- \| --- \| --- \| --- \| --- \| --- \| --- \| --- \| --- \| --- \| --- \| --- \| --- \| --- \| --- \| --- \| --- \| --- \| --- \| --- \| --- \| --- \| | \|  \| \| --- \| \| **ICD9/TERMWORKS** \| \| **ICD9/TERMWORKS** \| \| **ICD9/TERMWORKS** \| \|  \| \| **ICD9/TERMWORKS** \| \| **ICD9/TERMWORKS** \| \| **ICD9-CM/TERMWORKS** \| \| **ICD9-CM/TERMWORKS** \| \| **ICD9-CM/TERMWORKS** \| \| **ICD9-CM/TERMWORKS** \| \| **ICD9-CM/TERMWORKS** \| \| **ICD9-CM/TERMWORKS** \| \| **ICD9-CM/TERMWORKS** \| \| **ICD9-CM/TERMWORKS** \| \| **ICD9-CM/TERMWORKS** \| \| **ICD9/TERMWORKS** \| \| **ICD9-CM/TERMWORKS** \| \| **ICD9-CM/TERMWORKS** \| \| **ICD9-CM/TERMWORKS** \| \| **ICD9-CM/TERMWORKS** \| \| **ICD9-CM/TERMWORKS** \| \| **ICD9-CM/TERMWORKS** \| \| **ICD9-CM/TERMWORKS** \| \| **ICD9-CM/TERMWORKS** \| \| **ICD9-CM/TERMWORKS** \| \| **ICD9-CM/TERMWORKS** \| \| **ICD9-CM/TERMWORKS** \| \| **ICD9-CM/TERMWORKS** \| \| **ICD9-CM/TERMWORKS** \| \| **ICD9-CM/TERMWORKS** \| \| **ICD9/TERMWORKS** \| \| **ICD9/TERMWORKS** \| \|  \| \| **ICD9-CM** \| \| **ICD9-CM** \| \|  \| \| **CPCSSN Free Text** \| \| **CPCSSN Free Text** \| \| **CPCSSN Free Text** \| \| **CPCSSN Free Text** \| \| **CPCSSN Free Text** \| \| **CPCSSN Free Text** \| \| **CPCSSN Free Text** \| \| **CPCSSN Free Text** \| \| **CPCSSN Free Text** \| \| **CPCSSN Free Text** \| \| **CPCSSN Free Text** \| \| **CPCSSN Free Text** \| \| **CPCSSN Free Text** \| \| **CPCSSN Free Text** \| |

Termworks search terms: ulcer, pressure sore, varicose vein

## Sleep disturbance

| **Codes and Terms** | **Source(s)** |
| --- | --- |
| \| \| 307.4 SPECIFIC DISORDERS OF SLEEP \| \| --- \| \| 307.40 Nonorganic sleep disorder, unspecified \| \| 307.41 Transient disorder of initiating or maintaining sleep \| \| 307.42 Persistent disorder of initiating or maintaining sleep \| \| 307.43 Transient disorder of initiating or maintaining wakefulness \| \| 307.44 Persistent disorder of initiating or maintaining wakefulness \| \| 307.45 Circadian rhythm sleep disorder of nonorganic origin \| \| 307.46 Sleep arousal disorder \| \| 307.47 Other dysfunctions of sleep stages or arousal from sleep \| \| 307.48 Repetitive intrusions of sleep \| \| 307.49 Other specific disorders of sleep of nonorganic origin \| \|  \| \| 327 Organic sleep disorders \| \| 327.0 Organic disorders of initiating and maintaining sleep [organic insomnia] \| \| 327.00 Organic insomnia, unspecified \| \| 327.01 Insomnia due to medical condition classified elsewhere \| \| 327.02 Insomnia due to mental disorder \| \| 327.09 Other organic insomnia \| \| 327.1 Organic disorder of excessive somnolence [organic hypersomnia] \| \| 327.10 Organic hypersomnia, unspecified \| \| 327.11 Idiopathic hypersomnia with long sleep time \| \| 327.12 Idiopathic hypersomnia without long sleep time \| \| 327.13 Recurrent hypersomnia \| \| 327.14 Hypersomnia due to medical condition classified elsewhere \| \| 327.15 Hypersomnia due to mental disorder \| \| 327.19 Other organic hypersomnia \| \| 327.2 Organic sleep apnea \| \| 327.20 Organic sleep apnea, unspecified \| \| 327.21 Primary central sleep apnea \| \| 327.22 High altitude periodic breathing \| \| 327.23 Obstructive sleep apnea (adult)(pediatric) \| \| 327.24 Idiopathic sleep related non-obstructive alveolar hypoventilation \| \| 327.25 Congenital central alveolar hypoventilation syndrome \| \| 327.26 Sleep related hypoventilation/hypoxemia in conditions classifiable elsewhere \| \| 327.27 Central sleep apnea in conditions classified elsewhere \| \| 327.29 Other organic sleep apnea \| \| 327.3 Circadian rhythm sleep disorder \| \| 327.30 Circadian rhythm sleep disorder, unspecified \| \| 327.31 Circadian rhythm sleep disorder, delayed sleep phase type \| \| 327.32 Circadian rhythm sleep disorder, advanced sleep phase type \| \| 327.33 Circadian rhythm sleep disorder, irregular sleep-wake type \| \| 327.34 Circadian rhythm sleep disorder, free-running type \| \| 327.37 Circadian rhythm sleep disorder in conditions classified elsewhere \| \| 327.39 Other circadian rhythm sleep disorder \| \| 327.4 Organic parasomnia \| \| 327.40 Organic parasomnia, unspecified \| \| 327.41 Confusional arousals \| \| 327.42 REM sleep behavior disorder \| \| 327.43 Recurrent isolated sleep paralysis \| \| 327.44 Parasomnia in conditions classified elsewhere \| \| 327.49 Other organic parasomnia \| \|  \| \| SYMPTOMS, SIGNS AND ILL-DEFINED CONDITIONS \| \| 780.5 SLEEP DISTURBANCES \| \| 780.50 Sleep disturbance, unspecified \| \| 780.51 Insomnia with sleep apnea, unspecified \| \| 780.52 Insomnia, unspecified \| \| 780.53 Hypersomnia with sleep apnea, unspecified \| \| 780.54 Hypersomnia, unspecified \| \| 780.55 Disruption of 24 hour sleep wake cycle, unspecified \| \| 780.56 Dysfunctions associated with sleep stages or arousal from sleep \| \| 780.57 Unspecified sleep apnea \| \| 780.58 Sleep related movement disorder, unspecified \| \| 780.59 Other sleep disturbances \| \|  \| \| V69.4 Lack of adequate sleep \| \| 01A Dizziness, vertigo, insomnia \| \| Can’t sleep \| \| Disturbed sleep \| \| Insomnia \| \| Lack of sleep \| \| Night terror \| \| Night time wandering \| \| Not sleeping \| \| Obstructive sleep apnea \| \| Persistent insomnia \| \| Poor sleep \| \| Sleep aid \| \| Sleep apnea \| \| Sleep clinic \| \| Sleep deprivation \| \| Sleep diary \| \| Sleep difficulty \| \| Sleep disorder \| \| Sleep disruption \| \| Sleep disturbance \| \| Sleep hygiene \| \| Sleep issue \| \| Sleep medication \| \| Sleep problem \| \| Sleep walking \| \| Sleeping disorder \| \| Sleeping issue \| \| Sleeping medication \| \| Sleeping pill \| \| Sleepless \| \| Sleeplessness \| \| Trouble sleeping \| \| Unable to sleep \| \| \| --- \| --- \| --- \| --- \| --- \| --- \| --- \| --- \| --- \| --- \| --- \| --- \| --- \| --- \| --- \| --- \| --- \| --- \| --- \| --- \| --- \| --- \| --- \| --- \| --- \| --- \| --- \| --- \| --- \| --- \| --- \| --- \| --- \| --- \| --- \| --- \| --- \| --- \| --- \| --- \| --- \| --- \| --- \| --- \| --- \| --- \| --- \| --- \| --- \| --- \| --- \| --- \| --- \| --- \| --- \| --- \| --- \| --- \| --- \| --- \| --- \| --- \| --- \| --- \| --- \| --- \| --- \| --- \| --- \| --- \| --- \| --- \| --- \| --- \| --- \| --- \| --- \| --- \| --- \| --- \| --- \| --- \| --- \| --- \| --- \| --- \| --- \| --- \| --- \| --- \| --- \| --- \| --- \| --- \| --- \| --- \| --- \| --- \| --- \| --- \| | \| **ICD9/TERMWORKS** \| \| --- \| \| **ICD9-CM/TERMWORKS** \| \| **ICD9-CM/TERMWORKS** \| \| **ICD9-CM/TERMWORKS** \| \| **ICD9-CM** \| \| **ICD9-CM** \| \| **ICD9-CM/TERMWORKS** \| \| **ICD9-CM/TERMWORKS** \| \| **ICD9-CM/TERMWORKS** \| \| **ICD9-CM/TERMWORKS** \| \| **ICD9-CM/TERMWORKS** \| \|  \| \| **ICD9-CM/TERMWORKS** \| \| **ICD9-CM/TERMWORKS** \| \| **ICD9-CM/TERMWORKS** \| \| **ICD9-CM/TERMWORKS** \| \| **ICD9-CM/TERMWORKS** \| \| **ICD9-CM/TERMWORKS** \| \| **ICD9-CM** \| \| **ICD9-CM** \| \| **ICD9-CM/TERMWORKS** \| \| **ICD9-CM/TERMWORKS** \| \| **ICD9-CM** \| \| **ICD9-CM** \| \| **ICD9-CM** \| \| **ICD9-CM** \| \| **ICD9-CM/TERMWORKS** \| \| **ICD9-CM/TERMWORKS** \| \| **ICD9-CM/TERMWORKS** \| \| **ICD9-CM** \| \| **ICD9-CM/TERMWORKS** \| \| **ICD9-CM/TERMWORKS** \| \| **ICD9-CM** \| \| **ICD9-CM/TERMWORKS** \| \| **ICD9-CM/TERMWORKS** \| \| **ICD9-CM/TERMWORKS** \| \| **ICD9-CM/TERMWORKS** \| \| **ICD9-CM/TERMWORKS** \| \| **ICD9-CM/TERMWORKS** \| \| **ICD9-CM/TERMWORKS** \| \| **ICD9-CM/TERMWORKS** \| \| **ICD9-CM/TERMWORKS** \| \| **ICD9-CM/TERMWORKS** \| \| **ICD9-CM/TERMWORKS** \| \| **ICD9-CM** \| \| **ICD9-CM** \| \| **ICD9-CM** \| \| **ICD9-CM/TERMWORKS** \| \| **ICD9-CM/TERMWORKS** \| \| **ICD9-CM** \| \| **ICD9-CM** \| \|  \| \|  \| \| **ICD9/TERMWORKS** \| \| **ICD9-CM/TERMWORKS** \| \| **ICD9-CM/TERMWORKS** \| \| **ICD9-CM/TERMWORKS** \| \| **ICD9-CM/TERMWORKS** \| \| **ICD9-CM** \| \| **ICD9-CM/TERMWORKS** \| \| **ICD9-CM/TERMWORKS** \| \| **ICD9-CM/TERMWORKS** \| \| **ICD9-CM/TERMWORKS** \| \| **ICD9-CM/TERMWORKS** \| \|  \| \| **ICD9-CM/TERMWORKS** \| \| **ICD9** \| \|  \| \| **CPCSSN Free Text** \| \| **CPCSSN Free Text** \| \| **CPCSSN Free Text** \| \| **CPCSSN Free Text** \| \| **CPCSSN Free Text** \| \| **CPCSSN Free Text** \| \| **CPCSSN Free Text** \| \| **CPCSSN Free Text** \| \| **CPCSSN Free Text** \| \| **CPCSSN Free Text** \| \| **CPCSSN Free Text** \| \| **CPCSSN Free Text** \| \| **CPCSSN Free Text** \| \| **CPCSSN Free Text** \| \| **CPCSSN Free Text** \| \| **CPCSSN Free Text** \| \| **CPCSSN Free Text** \| \| **CPCSSN Free Text** \| \| **CPCSSN Free Text** \| \| **CPCSSN Free Text** \| \| **CPCSSN Free Text** \| \| **CPCSSN Free Text** \| \| **CPCSSN Free Text** \| \| **CPCSSN Free Text** \| \| **CPCSSN Free Text** \| \| **CPCSSN Free Text** \| \| **CPCSSN Free Text** \| \| **CPCSSN Free Text** \| \| **CPCSSN Free Text** \| \| **CPCSSN Free Text** \| \| **CPCSSN Free Text** \| \| **CPCSSN Free Text** \| \| **CPCSSN Free Text** \| |

Termworks search terms: sleep, insomnia

## Social vulnerability

| **Codes and Terms** | **Source(s)** |
| --- | --- |
| \| \| V60 HOUSING, HOUSEHOLD AND ECONOMIC CIRCUMSTANCES \| \| --- \| \| V60.0 LACK OF HOUSING \| \| V60.1 INADEQUATE HOUSING \| \| V60.2 INADEQUATE MATERIAL RESOURCES \| \| V60.3 PERSON LIVING ALONE \| \| V60.8 OTHER \| \| V60.9 UNSPECIFIED \| \|  \| \| V61.03 Family disruption due to divorce or legal separation \| \| V61.07 Family disruption due to death of family member \| \| V61.08 Family disruption due to other extended absence of family member \| \| V61.1 MARITAL PROBLEMS \| \| V61.10 Counseling for marital and partner problems, unspecified \| \| V61.11 Counseling for victim of spousal and partner abuse \| \|  \| \| V62.8 OTHER PSYCHOLOGICAL OR PHYSICAL STRAIN, NOT ELSEWHERE CLASSIFIED \| \| V62.81 Interpersonal problems, not elsewhere classified \| \| V62.82 Bereavement, uncomplicated \| \| V62.89 Other psychological or physical stress, not elsewhere classified \| \| V71.81 Observation and evaluation for suspected abuse and neglect \| \|  \| \| V63.0 RESIDENCE REMOTE FROM HOSPITAL OR OTHER HEALTH CARE FACILITY \| \| V63.1 MEDICAL SERVICES IN HOME NOT AVAILABLE \| \|  \| \| 783.7 Adult failure to thrive \| \|  \| \| 995.85 Other adult abuse and neglect \| \|  \| \| Advocate for housing \| \| Bereavement \| \| Bereavement counselling \| \| Care for spouse \| \| Caregiver burnout \| \| Caregiver burden \| \| Caregiver stress \| \| Death in family \| \| Death of husband \| \| Death of spouse \| \| Death of wife \| \| Economic problem \| \| Elder abuse \| \| Elderly neglect \| \| Emotional support \| \| Emotional issue \| \| Family concern \| \| Family conflict \| \| Family crisis \| \| Family death \| \| Family distress \| \| Family issue \| \| Family problem \| \| Family stressor \| \| Family situation stressful \| \| Financial problem \| \| Grief \| \| Grief reaction \| \| Homeless \| \| Housing \| \| Housing note \| \| Housing issue \| \| Housing inquiries \| \| Husband’s death \| \| Intimate partner violence \| \| Life stressor \| \| Loneliness \| \| Low mood \| \| Marital conflict \| \| Marital separation \| \| Marital problem \| \| No MSP \| \| Poverty \| \| Psychosocial problem \| \| Psychological stress \| \| Psychosocial Stressor \| \| Psychosocial trauma \| \| Psychological trauma \| \| Relationship problem \| \| Safety at home \| \| Self neglect \| \| Situational crisis \| \| Social assistance \| \| Social circumstance \| \| Social isolation \| \| Social issue \| \| Social prescribing \| \| Social problem \| \| Social situation \| \| Social work \| \| Supplemental housing form \| \| SW support \| \| Transient housing \| \| Unable to cope \| \| Unstable housing \| \| Wife’s death \| \|  \| \| \| --- \| --- \| --- \| --- \| --- \| --- \| --- \| --- \| --- \| --- \| --- \| --- \| --- \| --- \| --- \| --- \| --- \| --- \| --- \| --- \| --- \| --- \| --- \| --- \| --- \| --- \| --- \| --- \| --- \| --- \| --- \| --- \| --- \| --- \| --- \| --- \| --- \| --- \| --- \| --- \| --- \| --- \| --- \| --- \| --- \| --- \| --- \| --- \| --- \| --- \| --- \| --- \| --- \| --- \| --- \| --- \| --- \| --- \| --- \| --- \| --- \| --- \| --- \| --- \| --- \| --- \| --- \| --- \| --- \| --- \| --- \| --- \| --- \| --- \| --- \| --- \| --- \| --- \| --- \| --- \| --- \| --- \| --- \| --- \| --- \| --- \| --- \| --- \| --- \| --- \| --- \| --- \| --- \| --- \| --- \| --- \| | \| **ICD9/TERMWORKS** \| \| --- \| \| **ICD9/TERMWORKS** \| \| **ICD9/TERMWORKS** \| \| **ICD9/TERMWORKS** \| \| **ICD9/TERMWORKS** \| \| **ICD9/TERMWORKS** \| \| **ICD9/TERMWORKS** \| \|  \| \| **ICD9-CM/TERMWORKS** \| \| **ICD9-CM/TERMWORKS** \| \| **ICD9-CM/TERMWORKS** \| \| **ICD9** \| \| **ICD9-CM** \| \| **ICD9-CM** \| \|  \| \| **ICD9** \| \| **ICD9-CM** \| \| **ICD9-CM** \| \| **ICD9-CM** \| \| **ICD9-CM** \| \|  \| \| **ICD9** \| \| **ICD9** \| \|  \| \| **ICD9-CM** \| \|  \| \| **ICD9-CM** \| \|  \| \| **CPCSSN Free Text** \| \| **CPCSSN Free Text** \| \| **CPCSSN Free Text** \| \| **CPCSSN Free Text** \| \| **CPCSSN Free Text** \| \| **CPCSSN Free Text** \| \| **CPCSSN Free Text** \| \| **CPCSSN Free Text** \| \| **CPCSSN Free Text** \| \| **CPCSSN Free Text** \| \| **CPCSSN Free Text** \| \| **CPCSSN Free Text** \| \| **CPCSSN Free Text** \| \| **CPCSSN Free Text** \| \| **CPCSSN Free Text** \| \| **CPCSSN Free Text** \| \| **CPCSSN Free Text** \| \| **CPCSSN Free Text** \| \| **CPCSSN Free Text** \| \| **CPCSSN Free Text** \| \| **CPCSSN Free Text** \| \| **CPCSSN Free Text** \| \| **CPCSSN Free Text** \| \| **CPCSSN Free Text** \| \| **CPCSSN Free Text** \| \| **CPCSSN Free Text** \| \| **CPCSSN Free Text** \| \| **CPCSSN Free Text** \| \| **CPCSSN Free Text** \| \| **CPCSSN Free Text** \| \| **CPCSSN Free Text** \| \| **CPCSSN Free Text** \| \| **CPCSSN Free Text** \| \| **CPCSSN Free Text** \| \| **CPCSSN Free Text** \| \| **CPCSSN Free Text** \| \| **CPCSSN Free Text** \| \| **CPCSSN Free Text** \| \| **CPCSSN Free Text** \| \| **CPCSSN Free Text** \| \| **CPCSSN Free Text** \| \| **CPCSSN Free Text** \| \| **CPCSSN Free Text** \| \| **CPCSSN Free Text** \| \| **CPCSSN Free Text** \| \| **CPCSSN Free Text** \| \| **CPCSSN Free Text** \| \| **CPCSSN Free Text** \| \| **CPCSSN Free Text** \| \| **CPCSSN Free Text** \| \| **CPCSSN Free Text** \| \| **CPCSSN Free Text** \| \| **CPCSSN Free Text** \| \| **CPCSSN Free Text** \| \| **CPCSSN Free Text** \| \| **CPCSSN Free Text** \| \| **CPCSSN Free Text** \| \| **CPCSSN Free Text** \| \| **CPCSSN Free Text** \| \| **CPCSSN Free Text** \| \| **CPCSSN Free Text** \| \| **CPCSSN Free Text** \| \| **CPCSSN Free Text** \| \| **CPCSSN Free Text** \| \| **CPCSSN Free Text** \| \| **CPCSSN Free Text** \| |

Termworks search terms: social vulnerability, widow, widowed, vulnerable, social, family, lonely, economic, housing, alone, death

## Thyroid Disorder

| **Codes and Terms** | **Source(s)** |
| --- | --- |
| \| \| 240 SIMPLE AND UNSPECIFIED GOITRE* \| \| --- \| \| 240.0 GOITRE, SPECIFIED AS SIMPLE \| \| 240.9 GOITRE, UNSPECIFIED \| \|  \| \| 241 NONTOXIC NODULAR GOITRE* \| \| 241.0 NONTOXIC UNINODULAR GOITRE \| \| 241.1 NONTOXIC MULTINODULAR GOITRE \| \| 241.9 UNSPECIFIED \| \|  \| \| 242 THYROTOXICOSIS WITH OR WITHOUT GOITRE* \| \| 242.0 TOXIC DIFFUSE GOITRE \| \| 242.00 Toxic diffuse goiter without mention of thyrotoxic crisis or storm \| \| 242.01 Toxic diffuse goiter with mention of thyrotoxic crisis or storm \| \| 242.1 TOXIC UNINODULAR GOITRE \| \| 242.10 Toxic uninodular goiter without mention of thyrotoxic crisis or storm \| \| 242.11 Toxic uninodular goiter with mention of thyrotoxic crisis or storm \| \| 242.2 TOXIC MULTINODULAR GOITRE \| \| 242.20 Toxic multinodular goiter without mention of thyrotoxic crisis or storm \| \| 242.21 Toxic multinodular goiter with mention of thyrotoxic crisis or storm \| \| 242.3 TOXIC NODULAR GOITRE, UNSPECIFIED \| \| 242.30 Toxic nodular goiter, unspecified type, without mention of thyrotoxic crisis or storm \| \| 242.31 Toxic nodular goiter, unspecified type, with mention of thyrotoxic crisis or storm \| \| 242.4 THYROTOXICOSIS FROM ECTOPIC THYROID NODULE \| \| 242.40 Thyrotoxicosis from ectopic thyroid nodule without mention of thyrotoxic crisis or storm \| \| 242.41 Thyrotoxicosis from ectopic thyroid nodule with mention of thyrotoxic crisis or storm \| \| 242.8 THYROTOXICOSIS OF OTHER SPECIFIED ORIGIN \| \| 242.80 Thyrotoxicosis of other specified origin without mention of thyrotoxic crisis or storm \| \| 242.81 Thyrotoxicosis of other specified origin with mention of thyrotoxic crisis or storm \| \| 242.9 THYROTOXICOSIS WITHOUT MENTION OF GOITRE OR OTHER CAUSE \| \| 242.90 Thyrotoxicosis without mention of goiter or other cause, and without mention of thyrotoxic crisis or storm \| \| 242.91 Thyrotoxicosis without mention of goiter or other cause, with mention of thyrotoxic crisis or storm \| \|  \| \| 243 CONGENITAL HYPOTHYROIDISM \| \|  \| \| 244 ACQUIRED HYPOTHYROIDISM* \| \| 244.0 POSTSURGICAL HYPOTHYROIDISM \| \| 244.1 OTHER POSTABLATIVE HYPOTHYROIDISM \| \| 244.2 IODINE HYPOTHYROIDISM \| \| 244.3 OTHER IATROGENIC HYPOTHYROIDISM \| \| 244.8 OTHER \| \| 244.9 UNSPECIFIED HYPOTHYROIDISM \| \|  \| \| 245 THYROIDITIS* \| \| 245.0 ACUTE THYROIDITIS \| \| 245.1 SUBACUTE THYROIDITIS \| \| 245.2 CHRONIC LYMPHOCYTIC THYROIDITIS \| \| 245.3 CHRONIC FIBROUS THYROIDITIS \| \| 245.4 IATROGENIC THYROIDITIS \| \| 245.8 OTHER AND UNSPECIFIED CHRONIC THYROIDITIS \| \| 245.9 UNSPECIFIED \| \|  \| \| 246 OTHER DISORDERS OF THE THYROID* \| \| 246.0 DISORDERS OF THYROCALCITONIN SECRETION \| \| 246.1 DYSHORMONOGENIC GOITRE \| \| 246.2 CYST OF THYROID \| \| 246.3 HAEMORRHAGE AND INFARCTION OF THYROID \| \| 246.8 OTHER \| \| 246.9 UNSPECIFIED \| \|  \| \| NONSPECIFIC ABNORMAL RESULTS OF FUNCTION STUDIES \| \| 794.5 THYROID \| \|  \| \| SPECIAL SCREENING FOR ENDOCRINE, NUTRITIONAL, METABOLIC AND IMMUNITY \| \| V77.0 THYROID DISORDERS \| \|  \| \| **T3 FREE (pmol/L)** \| \| normal: 3.5-6.5 pmol/L \| \|  \| \| **T4 FREE (pmol/L)** \| \| normal: 10.3-23.2 pmol/L \| \|  \| \| **TSH (Thyroid Stimulating Hormone) (mU/L)** \| \| normal: 0.5-4 mIU/L \| \|  \| \| **Thyroperoxidase Ab (IU/mL)** \| \| Thyroperoxidase Ab [Units/volume] in Serum or Plasma: normal <35 IU/mL \| \| Thyroperoxidase Ab [Units/volume] in Serum or Plasma by Immunoassay: normal <35 IU/mL \| \| Thyroperoxidase Ab [Presence] in Serum or Plasma \| \|  \| \| **Levothyroxine Prescription** \| \|  \| \| Goiter \| \| Hyperthyroid \| \| Hyperthyroidism \| \| Hyperactive thyroid \| \| Hypothyroid \| \| hypothyroidism \| \| Low thyroid \| \| Thyroid deficiency \| \| Thyroid disorder \| \| Thyroid function test abnormal \| \| Thyroid goiter \| \| Thyroid issue \| \| Thyroid nodule \| \| Thyroiditis \| \|  \| \| \| --- \| --- \| --- \| --- \| --- \| --- \| --- \| --- \| --- \| --- \| --- \| --- \| --- \| --- \| --- \| --- \| --- \| --- \| --- \| --- \| --- \| --- \| --- \| --- \| --- \| --- \| --- \| --- \| --- \| --- \| --- \| --- \| --- \| --- \| --- \| --- \| --- \| --- \| --- \| --- \| --- \| --- \| --- \| --- \| --- \| --- \| --- \| --- \| --- \| --- \| --- \| --- \| --- \| --- \| --- \| --- \| --- \| --- \| --- \| --- \| --- \| --- \| --- \| --- \| --- \| --- \| --- \| --- \| --- \| --- \| --- \| --- \| --- \| --- \| --- \| --- \| --- \| --- \| --- \| --- \| --- \| --- \| --- \| --- \| --- \| --- \| --- \| --- \| --- \| --- \| --- \| --- \| --- \| --- \| --- \| --- \| --- \| | \| **ICD9** \| \| --- \| \| **ICD9** \| \| **ICD9** \| \|  \| \| **ICD9** \| \| **ICD9** \| \| **ICD9** \| \| **ICD9** \| \|  \| \| **ICD9/TERMWORKS** \| \| **ICD9** \| \| **ICD9-CM** \| \| **ICD9-CM** \| \| **ICD9** \| \| **ICD9-CM** \| \| **ICD9-CM** \| \| **ICD9** \| \| **ICD9-CM** \| \| **ICD9-CM** \| \| **ICD9** \| \| **ICD9-CM** \| \| **ICD9-CM** \| \| **ICD9/TERMWORKS** \| \| **ICD9-CM/TERMWORKS** \| \| **ICD9-CM/TERMWORKS** \| \| **ICD9/TERMWORKS** \| \| **ICD9-CM/TERMWORKS** \| \| **ICD9-CM/TERMWORKS** \| \| **ICD9/TERMWORKS** \| \| **ICD9-CM/TERMWORKS** \| \| **ICD9-CM/TERMWORKS** \| \|  \| \| **ICD9/TERMWORKS** \| \|  \| \| **ICD9/TERMWORKS** \| \| **ICD9/TERMWORKS** \| \| **ICD9/TERMWORKS** \| \| **ICD9/TERMWORKS** \| \| **ICD9/TERMWORKS** \| \| **ICD9/TERMWORKS** \| \| **ICD9/TERMWORKS** \| \|  \| \| **ICD9** \| \| **ICD9** \| \| **ICD9** \| \| **ICD9** \| \| **ICD9** \| \| **ICD9** \| \| **ICD9** \| \| **ICD9** \| \|  \| \| **ICD9/TERMWORKS** \| \| **ICD9** \| \| **ICD9** \| \| **ICD9/TERMWORKS** \| \| **ICD9/TERMWORKS** \| \| **ICD9/TERMWORKS** \| \| **ICD9/TERMWORKS** \| \|  \| \|  \| \| **ICD9/TERMWORKS** \| \|  \| \|  \| \| **ICD9/TERMWORKS** \| \|  \| \| **LOINC 14928-6** \| \|  \| \|  \| \| **LOINC 14920-3** \| \|  \| \|  \| \| **LOINC 3016-3** \| \|  \| \|  \| \|  \| \| **LOINC 8099-4** \| \| **LOINC 56477-3** \| \| **LOINC 32042-4** \| \|  \| \|  \| \|  \| \| **CPCSSN Free Text** \| \| **CPCSSN Free Text** \| \| **CPCSSN Free Text** \| \| **CPCSSN Free Text** \| \| **CPCSSN Free Text** \| \| **CPCSSN Free Text** \| \| **CPCSSN Free Text** \| \| **CPCSSN Free Text** \| \| **CPCSSN Free Text** \| \| **CPCSSN Free Text** \| \| **CPCSSN Free Text** \| \| **CPCSSN Free Text** \| \| **CPCSSN Free Text** \| \| **CPCSSN Free Text** \| \|  \| |

Termworks search terms: thyroid, hyperthyroidism, hypothyroidism, thyrotoxicosis

## Urinary incontinence

| **Codes and Terms** | **Source(s)** |
| --- | --- |
| \| \| 344.61 Cauda equina syndrome with neurogenic bladder \| \| --- \| \|  \| \| 596.4 ATONY OF BLADDER \| \| 596.5 Other functional disorders of bladder \| \| 596.53 Paralysis of bladder \| \| 596.54 Neurogenic bladder NOS \| \|  \| \| 625.6 STRESS INCONTINENCE, FEMALE \| \|  \| \| SYMPTOMS, SIGNS AND ILL-DEFINED CONDITIONS \| \| 788.3 INCONTINENCE OF URINE (ICD9-CM: "URINARY INCONTINENCE") \| \| 788.30 Urinary incontinence, unspecified \| \| 788.31 Urge incontinence \| \| 788.32 Stress incontinence, male \| \| 788.33 Mixed incontinence (male) (female) \| \| 788.34 Incontinence without sensory awareness \| \| 788.35 Post-void dribbling \| \| 788.36 Nocturnal enuresis \| \| 788.37 Continuous leakage \| \| 788.38 Overflow incontinence \| \| 788.39 Other urinary incontinence \| \|  \| \| 788.9 Other symptoms involving urinary system \| \| 788.91 Functional urinary incontinence \| \|  \| \| Atony of bladder \| \| Bladder atony \| \| Bladder control \| \| Incontinence \| \| Incontinence of urine \| \| Mixed incontinence \| \| Stress incontinence \| \| Urge incontinence \| \| Urinary incontinence \| \| Urinary incontinence chronic \| \| Urinary incontinence stress \| \| Urinary incontinence overflow \| \| Urinary incontinence urge \| \| Urine incontinence \| \|  \| \| \| --- \| --- \| --- \| --- \| --- \| --- \| --- \| --- \| --- \| --- \| --- \| --- \| --- \| --- \| --- \| --- \| --- \| --- \| --- \| --- \| --- \| --- \| --- \| --- \| --- \| --- \| --- \| --- \| --- \| --- \| --- \| --- \| --- \| --- \| --- \| --- \| --- \| --- \| --- \| --- \| --- \| | \| **ICD9-CM** \| \| --- \| \|  \| \| **ICD9/TERMWORKS** \| \| **ICD9-CM/TERMWORKS** \| \| **ICD9-CM/TERMWORKS** \| \| **ICD9-CM/TERMWORKS** \| \|  \| \| **ICD9/TERMWORKS** \| \|  \| \|  \| \| **ICD9/TERMWORKS** \| \| **ICD9-CM/TERMWORKS** \| \| **ICD9-CM/TERMWORKS** \| \| **ICD9-CM/TERMWORKS** \| \| **ICD9-CM/TERMWORKS** \| \| **ICD9-CM/TERMWORKS** \| \| **ICD9-CM** \| \| **ICD9-CM** \| \| **ICD9-CM/TERMWORKS** \| \| **ICD9-CM/TERMWORKS** \| \| **ICD9-CM/TERMWORKS** \| \|  \| \| **ICD9-CM** \| \| **ICD9-CM/TERMWORKS** \| \|  \| \| **CPCSSN Free Text** \| \| **CPCSSN Free Text** \| \| **CPCSSN Free Text** \| \| **CPCSSN Free Text** \| \| **CPCSSN Free Text** \| \| **CPCSSN Free Text** \| \| **CPCSSN Free Text** \| \| **CPCSSN Free Text** \| \| **CPCSSN Free Text** \| \| **CPCSSN Free Text** \| \| **CPCSSN Free Text** \| \| **CPCSSN Free Text** \| \| **CPCSSN Free Text** \| \| **CPCSSN Free Text** \| |

Termworks search terms: incontinence, bladder, urine

## Urinary system disease

| **Codes and Terms** | **Source(s)** |
| --- | --- |
| \| \| DISEASES OF THE GENITOURINARY SYSTEM \| \| --- \| \| 594 CALCULUS OF LOWER URINARY TRACT \| \| 594.0 CALCULUS IN DIVERTICULUM OF BLADDER \| \| 594.1 OTHER CALCULUS IN BLADDER \| \| 594.2 CALCULUS IN URETHRA \| \| 594.8 OTHER LOWER URINARY TRACT CALCULUS \| \| 594.9 UNSPECIFIED \| \|  \| \| 595 CYSTITIS \| \| 595.0 ACUTE CYSTITIS \| \| 595.1 CHRONIC INTERSTITIAL CYSTITIS \| \| 595.2 OTHER CHRONIC CYSTITIS \| \| 595.3 TRIGONITIS \| \| 595.4 CYSTITIS IN DISEASES CLASSIFIED ELSEWHERE \| \| 595.8 OTHER \| \| 595.81 Cystitis cystica \| \| 595.82 Irradiation cystitis \| \| 595.89 Other specified types of cystitis \| \| 595.9 UNSPECIFIED \| \|  \| \| 596 OTHER DISORDERS OF BLADDER \| \| 596.0 BLADDER-NECK OBSTRUCTION \| \| 596.1 INTESTINOVESICAL FISTULA \| \| 596.2 VESICAL FISTULA, NOT ELSEWHERE CLASSIFIED \| \| 596.3 DIVERTICULUM OF BLADDER \| \| 596.4 ATONY OF BLADDER \| \| 596.5 OTHER FUNCTIONAL DISORDERS OF BLADDER \| \| 596.51 Hypertonicity of bladder \| \| 596.52 Low bladder compliance \| \| 596.53 Paralysis of bladder \| \| 596.54 Neurogenic bladder NOS \| \| 596.55 Detrusor sphincter dyssynergi \| \| 596.59 Other functional disorder of bladder \| \| 596.6 RUPTURE OF BLADDER, NONTRAUMATIC \| \| 596.7 HAEMORRHAGE INTO BLADDER WALL \| \| 596.8 OTHER \| \| 596.81 Infection of cystostomy \| \| 596.82 Mechanical complication of cystostomy \| \| 596.83 Other complication of cystostomy \| \| 596.89 Other specified disorders of bladder \| \| 596.9 UNSPECIFIED \| \|  \| \| 599.0 URINARY TRACT INFECTION, SITE NOT SPECIFIED \| \| 599.6 URINARY OBSTRUCTION, UNSPECIFIED \| \| 599.60 Urinary obstruction, unspecified \| \| 599.69 Urinary obstruction, not elsewhere classified \| \| 599.7 HAEMATURIA \| \| 599.70 Hematuria, unspecified \| \| 599.71 Gross hematuria \| \| 599.72 Microscopic hematuria \| \|  \| \| DISEASES OF THE MALE GENITAL ORGANS \| \| 600 HYPERPLASIA OF PROSTRATE \| \| 600.0 Hypertrophy (benign) of prostate \| \| 600.00 Without urinary obstruction and other lower urinary tract symptom (LUTS) \| \| 600.01 With urinary obstruction and other lower urinary tract symptoms (LUTS) \| \| 600.1 Nodular prostate \| \| 600.10 Nodular prostate without urinary obstruction \| \| 600.11 Nodular prostate with urinary obstruction \| \| 600.2 Benign localized hyperplasia of prostate \| \| 600.20 Without urinary obstruction and other lower urinary tract symptoms (LUTS) \| \| 600.21 With urinary obstruction and other lower urinary tract symptoms (LUTS) \| \| 600.3 Cyst of prostate \| \| 600.9 Hyperplasia of prostate unspecified \| \| 600.90 Without urinary obstruction and other lower urinary symptoms (LUTS) \| \| 600.91 With urinary obstruction and other lower urinary symptoms (LUTS) \| \|  \| \| 601 INFLAMMATORY DISEASES OF PROSTRATE \| \| 601.1 CHRONIC PROSTATITIS \| \| 601.2 ABSCESS OF PROSTATE \| \| 601.3 PROSTATOCYSTITIS \| \| 601.4 PROSTATITIS IN DISEASES CLASSIFIED ELSEWHERE \| \| 601.8 OTHER \| \| 601.9 UNSPECIFIED \| \|  \| \| 602 OTHER DISORDERS OF PROSTATE \| \| 602.0 CALCULUS OF PROSTATE \| \| 602.1 CONGESTION OR HAEMORRHAGE OF PROSTATE \| \| 602.2 ATROPHY OF PROSTATE \| \| 602.3 Dysplasia of prostate \| \| 602.8 OTHER \| \| 602.9 UNSPECIFIED \| \|  \| \| 788 SYMPTOMS INVOLVING URINARY SYSTEM \| \| 788.0 RENAL COLIC \| \| 788.1 DYSURIA \| \| 788.2 RETENTION OF URINE \| \| 788.20 Retention of urine, unspecified \| \| 788.21 Incomplete bladder emptying \| \| 788.29 Other specified retention of urine \| \| 788.4 FREQUENCY OF URINATION AND POLYURIA \| \| 788.41 Urinary frequency \| \| 788.42 Polyuria \| \| 788.43 Nocturia \| \| 788.5 OLIGURIA AND ANURIA \| \| 788.6 OTHER ABNORMALITY OF URINATION \| \| 788.61 Splitting of urinary stream \| \| 788.62 Slowing of urinary stream \| \| 788.63 Urgency of urination \| \| 788.64 Urinary hesitancy \| \| 788.65 Straining on urination \| \| 788.69 Other abnormality of urination \| \| 788.7 URETHRAL DISCHARGE \| \| 788.8 EXTRAVASATION OF URINE \| \| 788.9 OTHER \| \| 788.91 Functional urinary incontinence \| \| 788.99 Other symptoms involving urinary system \| \|  \| \| V13.0 Personal History of DISORDERS OF the URINARY SYSTEM \| \| V13.00 Personal history of unspecified urinary disorder \| \| V13.01 Personal history of urinary calculi \| \| V13.02 Personal history, urinary (tract) infection \| \| V13.03 Personal history, nephrotic syndrome \| \| V13.09 Personal history of other specified urinary system disorders \| \|  \| \| V47.4 OTHER URINARY PROBLEMS \| \| V53.6 URINARY DEVICES \| \| V58.82 Fitting and adjustment of nonvascular catheter, NEC \| \|  \| \| SPECIAL SCREENING FOR CARDIOVASCULAR, RESPIRATORY AND GENITOURINARY DISEASES \| \| V81.6 OTHER AND UNSPECIFIED GENITOURINARY CONDITIONS \| \|  \| \| 790.93 Elevated prostate specific antigen [PSA] \| \| 996.31 Mechanical complication due to urethral (indwelling) catheter \| \|  \| \| **Nitrite [Presence] in Urine by Test strip** \| \| **WBCs in Urine** \| \| **Haematuria (Hemoglobin [Presence] in Urine by Test strip)** \| \|  \| \| Benign prostate hyperplasia \| \| Benign prostatic hyperplasia \| \| Benign prostatic hypertrophy \| \| Bladder disorder \| \| Bladder hypersensitivity \| \| Bladder infection \| \| Bladder issue \| \| Bladder spasm \| \| Blood in urine \| \| BPH \| \| Burning on voiding \| \| Chronic UTI \| \| Cystitis \| \| Cystitis recurrent \| \| Difficulty urinating \| \| Dysuria \| \| Enlarged prostate \| \| Elevated PSA \| \| Elevated prostate specific antigen \| \| Frequent urination \| \| Haematuria \| \| Hematuria \| \| Lower urinary tract symptoms \| \| LUTS \| \| Nocturia \| \| Overactive bladder \| \| Polyuria \| \| Prostate abnormal \| \| Prostate hyperplasia benign \| \| Prostatism \| \| Prostatitis \| \| PSA elevated \| \| Retention of urine \| \| Recurrent UTI \| \| Urinalysis abnormal \| \| Urine culture positive \| \| Urine dipstick abnormal \| \| Urinary problem \| \| Urinary difficulty \| \| Urinary frequency \| \| Urinary hesitancy \| \| Urinary issue \| \| Urinary retention \| \| Urinary symptoms \| \| Urinary tract issue \| \| Urinary tract infection \| \| Urinary tract infection recurrent \| \| Urinary urgency \| \| Urine retention \| \| Urinary retention \| \| UTI \| \| UTI symptom \| \|  \| \| \| --- \| --- \| --- \| --- \| --- \| --- \| --- \| --- \| --- \| --- \| --- \| --- \| --- \| --- \| --- \| --- \| --- \| --- \| --- \| --- \| --- \| --- \| --- \| --- \| --- \| --- \| --- \| --- \| --- \| --- \| --- \| --- \| --- \| --- \| --- \| --- \| --- \| --- \| --- \| --- \| --- \| --- \| --- \| --- \| --- \| --- \| --- \| --- \| --- \| --- \| --- \| --- \| --- \| --- \| --- \| --- \| --- \| --- \| --- \| --- \| --- \| --- \| --- \| --- \| --- \| --- \| --- \| --- \| --- \| --- \| --- \| --- \| --- \| --- \| --- \| --- \| --- \| --- \| --- \| --- \| --- \| --- \| --- \| --- \| --- \| --- \| --- \| --- \| --- \| --- \| --- \| --- \| --- \| --- \| --- \| --- \| --- \| --- \| --- \| --- \| --- \| --- \| --- \| --- \| --- \| --- \| --- \| --- \| --- \| --- \| --- \| --- \| --- \| --- \| --- \| --- \| --- \| --- \| --- \| --- \| --- \| --- \| --- \| --- \| --- \| --- \| --- \| --- \| --- \| --- \| --- \| --- \| --- \| --- \| --- \| --- \| --- \| --- \| --- \| --- \| --- \| --- \| --- \| --- \| --- \| --- \| --- \| --- \| --- \| --- \| --- \| --- \| --- \| --- \| --- \| --- \| --- \| --- \| --- \| --- \| --- \| --- \| --- \| --- \| --- \| --- \| --- \| --- \| --- \| --- \| --- \| --- \| --- \| --- \| --- \| --- \| --- \| --- \| --- \| --- \| --- \| --- \| --- \| | \| **ICD9/TERMWORKS** \| \| --- \| \| **ICD9/TERMWORKS** \| \| **ICD9/TERMWORKS** \| \| **ICD9/TERMWORKS** \| \| **ICD9/TERMWORKS** \| \| **ICD9/TERMWORKS** \| \|  \| \| **ICD9/TERMWORKS** \| \| **ICD9/TERMWORKS** \| \| **ICD9/TERMWORKS** \| \| **ICD9/TERMWORKS** \| \|  \| \| **ICD9/TERMWORKS** \| \| **ICD9/TERMWORKS** \| \| **ICD9-CM/TERMWORKS** \| \| **ICD9-CM/TERMWORKS** \| \| **ICD9-CM/TERMWORKS** \| \| **ICD9/TERMWORKS** \| \|  \| \| **ICD9/TERMWORKS** \| \| **ICD9/TERMWORKS** \| \| **ICD9** \| \| **ICD9** \| \| **ICD9/TERMWORKS** \| \| **ICD9/TERMWORKS** \| \| **ICD9/TERMWORKS** \| \| **ICD9-CM/TERMWORKS** \| \| **ICD9-CM/TERMWORKS** \| \| **ICD9-CM/TERMWORKS** \| \| **ICD9-CM/TERMWORKS** \| \| **ICD9-CM/TERMWORKS** \| \| **ICD9-CM/TERMWORKS** \| \| **ICD9/TERMWORKS** \| \| **ICD9/TERMWORKS** \| \| **ICD9/TERMWORKS** \| \| **ICD9-CM** \| \| **ICD9-CM** \| \| **ICD9-CM** \| \| **ICD9-CM/TERMWORKS** \| \| **ICD9/TERMWORKS** \| \|  \| \| **ICD9/TERMWORKS** \| \| **ICD9/TERMWORKS** \| \| **ICD9-CM/TERMWORKS** \| \| **ICD9-CM/TERMWORKS** \| \| **ICD9/TERMWORKS** \| \| **ICD9-CM/TERMWORKS** \| \| **ICD9-CM/TERMWORKS** \| \| **ICD9-CM/TERMWORKS** \| \|  \| \|  \| \| **ICD9/TERMWORKS** \| \| **ICD9-CM/TERMWORKS** \| \| **ICD9-CM/TERMWORKS** \| \| **ICD9-CM/TERMWORKS** \| \| **ICD9-CM/TERMWORKS** \| \| **ICD9-CM/TERMWORKS** \| \| **ICD9-CM/TERMWORKS** \| \| **ICD9-CM/TERMWORKS** \| \| **ICD9-CM/TERMWORKS** \| \| **ICD9-CM/TERMWORKS** \| \| **ICD9-CM/TERMWORKS** \| \| **ICD9-CM/TERMWORKS** \| \| **ICD9-CM/TERMWORKS** \| \| **ICD9-CM/TERMWORKS** \| \|  \| \| **ICD9/TERMWORKS** \| \| **ICD9** \| \| **ICD9/TERMWORKS** \| \| **ICD9** \| \| **ICD9** \| \| **ICD9/TERMWORKS** \| \| **ICD9** \| \|  \| \| **ICD9/TERMWORKS** \| \| **ICD9/TERMWORKS** \| \| **ICD9/TERMWORKS** \| \| **ICD9/TERMWORKS** \| \| **ICD9/TERMWORKS** \| \| **ICD9/TERMWORKS** \| \| **ICD9/TERMWORKS** \| \| **ICD9-CM** \| \| **ICD9/TERMWORKS** \| \| **ICD9** \| \| **ICD9/TERMWORKS** \| \| **ICD9/TERMWORKS** \| \| **ICD9-CM/TERMWORKS** \| \| **ICD9-CM/TERMWORKS** \| \| **ICD9-CM/TERMWORKS** \| \| **ICD9/TERMWORKS** \| \| **ICD9-CM/TERMWORKS** \| \| **ICD9-CM/TERMWORKS** \| \| **ICD9-CM/TERMWORKS** \| \| **ICD9** \| \| **ICD9** \| \| **ICD9-CM/TERMWORKS** \| \| **ICD9-CM/TERMWORKS** \| \| **ICD9-CM** \| \| **ICD9-CM/TERMWORKS** \| \| **ICD9-CM** \| \| **ICD9-CM** \| \| **ICD9/TERMWORKS** \| \| **ICD9/TERMWORKS** \| \| **ICD9/TERMWORKS** \| \| **ICD9-CM/TERMWORKS** \| \| **ICD9-CM/TERMWORKS** \| \|  \| \| **ICD9/TERMWORKS** \| \| **ICD9-CM/TERMWORKS** \| \| **ICD9-CM/TERMWORKS** \| \| **ICD9-CM/TERMWORKS** \| \| **ICD9-CM** \| \| **ICD9-CM/TERMWORKS** \| \|  \| \| **ICD9/TERMWORKS** \| \| **ICD9/TERMWORKS** \| \| **ICD9-CM/TERMWORKS** \| \|  \| \|  \| \| **ICD9/TERMWORKS** \| \|  \| \| **ICD9-CM/TERMWORKS** \| \| **ICD9-CM/TERMWORKS** \| \|  \| \| **LOINC 5802-4** \| \| **LOINC 5799-2** \| \| **LOINC 5794-3** \| \|  \| \| **CPCSSN Free Text** \| \| **CPCSSN Free Text** \| \| **CPCSSN Free Text** \| \| **CPCSSN Free Text** \| \| **CPCSSN Free Text** \| \| **CPCSSN Free Text** \| \| **CPCSSN Free Text** \| \| **CPCSSN Free Text** \| \| **CPCSSN Free Text** \| \| **CPCSSN Free Text** \| \| **CPCSSN Free Text** \| \| **CPCSSN Free Text** \| \| **CPCSSN Free Text** \| \| **CPCSSN Free Text** \| \| **CPCSSN Free Text** \| \| **CPCSSN Free Text** \| \| **CPCSSN Free Text** \| \| **CPCSSN Free Text** \| \| **CPCSSN Free Text** \| \| **CPCSSN Free Text** \| \| **CPCSSN Free Text** \| \| **CPCSSN Free Text** \| \| **CPCSSN Free Text** \| \| **CPCSSN Free Text** \| \| **CPCSSN Free Text** \| \| **CPCSSN Free Text** \| \| **CPCSSN Free Text** \| \| **CPCSSN Free Text** \| \| **CPCSSN Free Text** \| \| **CPCSSN Free Text** \| \| **CPCSSN Free Text** \| \| **CPCSSN Free Text** \| \| **CPCSSN Free Text** \| \| **CPCSSN Free Text** \| \| **CPCSSN Free Text** \| \| **CPCSSN Free Text** \| \| **CPCSSN Free Text** \| \| **CPCSSN Free Text** \| \| **CPCSSN Free Text** \| \| **CPCSSN Free Text** \| \| **CPCSSN Free Text** \| \| **CPCSSN Free Text** \| \| **CPCSSN Free Text** \| \| **CPCSSN Free Text** \| \| **CPCSSN Free Text** \| \| **CPCSSN Free Text** \| \| **CPCSSN Free Text** \| \| **CPCSSN Free Text** \| \| **CPCSSN Free Text** \| \| **CPCSSN Free Text** \| \| **CPCSSN Free Text** \| \| **CPCSSN Free Text** \| |

Termworks search terms: micturition, urinary, urine, prostate, prostatism, prostatic, nocturia, dysuria, hematuria, polyuria, Genitourinary, urethral, cystitis, detrusor, urinary tract infection, urine, catheter, urologist, bladder

## Visual impairment

| **Codes and Terms** | **Source(s)** |
| --- | --- |
| \| \| 360.21 PROGRESSIVE HIGH MYOPIA \| \| --- \| \| 360.41 BLIND HYPOTENSIVE EYE \| \| 360.42 BLIND HYPERTENSIVE EYE \| \|  \| \| 361 RETINAL DETACHMENTS AND DEFECTS* \| \| 361.0 RETINAL DETACHMENT WITH RETINAL DEFECT* \| \| 361.1 RETINOSCHISIS AND RETINAL CYSTS* \| \| 361.2 SEROUS RETINAL DETACHMENT* \| \| 361.3 RETINAL DEFECTS WITHOUT DETACHMENT* \| \| 361.8 OTHER FORMS OF RETINAL DETACHMENT* \| \| 361.9 UNSPECIFIED* \| \|  \| \| 362 OTHER RETINAL DISORDERS* \| \| 362.0 DIABETIC RETINOPATHY* \| \| 362.1 OTHER BACKGROUND RETINOPATHY AND RETINAL VASCULAR CHANGES* \| \| 362.2 OTHER PROLIFERATIVE RETINOPATHY* \| \| 362.3 RETINAL VASCULAR OCCLUSION* \| \| 362.4 SEPARATION OF RETINAL LAYERS* \| \| 362.5 DEGENERATION OF MACULA AND POSTERIOR POLE* \| \| 362.6 PERIPHERAL RETINAL DEGENERATIONS* \| \| 362.7 HEREDITARY RETINAL DYSTROPHIES* \| \| 362.8 OTHER RETINAL DISORDERS* \| \| 362.9 UNSPECIFIED* \| \|  \| \| 366 CATARACT* \| \| 366.1 SENILE CATARACT* \| \| 366.2 TRAUMATIC CATARACT* \| \| 366.3 CATARACT SECONDARY TO OCULAR DISORDERS* \| \| 366.4 CATARACT ASSOCIATED WITH OTHER DISORDERS* \| \| 366.5 AFTER-CATARACT* \| \| 366.8 OTHER CATARACT* \| \| 366.9 Unspecified cataract* \| \|  \| \| 367 DISORDERS OF REFRACTION AND ACCOMMODATION* \| \| 367.0 HYPERMETROPIA* \| \| 367.1 MYOPIA* \| \| 367.2 ASTIGMATISM* \| \| 367.3 ANISOMETROPIA AND ANISEIKONIA* \| \| 367.5 DISORDERS OF ACCOMMODATION* \| \| 367.8 OTHER* \| \| 367.9 UNSPECIFIED* \| \|  \| \| 368 VISUAL DISTURBANCES* \| \| 368.0 AMBLYOPIA EX ANOPSIA* \| \| 368.1 SUBJECTIVE VISUAL DISTURBANCES* \| \| 368.2 DIPLOPIA* \| \| 368.3 OTHER DISORDERS OF BINOCULAR VISION* \| \| 368.4 VISUAL FIELD DEFECTS* \| \| 368.5 COLOUR VISION DEFICIENCIES* \| \| 368.6 NIGHT BLINDNESS* \| \| 368.8 OTHER VISUAL DISTURBANCES* \| \| 368.9 UNSPECIFIED* \| \|  \| \| 369 BLINDNESS AND LOW VISION* \| \| 369.0 BLINDNESS, BOTH EYES (ICD9-CM: Profound vision impairment both eyes)* \| \| 369.1 BLINDNESS, ONE EYE, LOW VISION OTHER EYE (ICD9-CM: Modereate or severe visiion impairment better eye; profound vision impairment of lesser eye)* \| \| 369.2 LOW VISION, BOTH EYES (ICD9-CM: Moderate or severe vision impairment both eyes)* \| \| 369.3 UNQUALIFIED VISUAL LOSS, BOTH EYES* \| \| 369.4 BLINDNESS, LEGAL* \| \| 369.6 BLINDNESS, ONE EYE (ICD9-CM: Profound vision impairment one eye)* \| \| 369.7 LOW VISION, ONE EYE (ICD9-CM: Moderate or severe vision impairment one eye)* \| \| 369.8 UNQUALIFIED VISUAL LOSS, ONE EYE* \| \| 369.9 UNSPECIFIED VISUAL LOSS* \| \|  \| \| 370 KERATITIS* \| \| 370.0 CORNEAL ULCER* \| \| 370.1 DENDRITIC KERATITIS* \| \| 370.2 OTHER SUPERFICIAL KERATITIS WITHOUT CONJUNCTIVITIS* \| \| 370.3 CERTAIN TYPES KERATOCONJUNCTIVITIS* \| \| 370.4 OTHER AND UNSPECIFIED KERATOCONJUNCTIVITIS* \| \| 370.5 INTERSTITIAL AND DEEP KERATITIS* \| \| 370.6 CORNEAL NEOVASCULARIZATION* \| \| 370.8 OTHER FORMS OF KERATITIS* \| \| 370.9 UNSPECIFIED* \| \|  \| \| 371 CORNEAL OPACITY AND OTHER DISORDERS OF CORNEA* \| \| 371.0 CORNEAL SCARS AND OPACITIES* \| \| 371.1 CORNEAL PIGMENTATIONS AND DEPOSITS* \| \| 371.2 CORNEAL OEDEMA* \| \| 371.3 CHANGES OF CORNEAL MEMBRANES* \| \| 371.4 CORNEAL DEGENERATIONS* \| \| 371.5 HEREDITARY CORNEAL DYSTROPHIES* \| \| 371.6 KERATOCONUS* \| \| 371.7 OTHER CORNEAL DEFORMITIES* \| \| 371.8 OTHER CORNEAL DISORDERS* \| \| 371.9 UNSPECIFIED* \| \|  \| \| 377 DISORDERS OF OPTIC NERVE AND VISUAL PATHWAYS* \| \| 377.0 PAPILLOEDEMA* \| \| 377.1 OPTIC ATROPHY* \| \| 377.2 OTHER DISORDERS OF OPTIC DISK* \| \| 377.3 OPTIC NEURITIS* \| \| 377.4 OTHER DISORDERS OF OPTIC NERVE* \| \| 377.5 DISORDERS OF OPTIC CHIASM* \| \| 377.6 DISORDERS OF OTHER VISUAL PATHWAYS* \| \| 377.7 DISORDERS OF VISUAL CORTEX* \| \| 377.9 UNSPECIFIED* \| \|  \| \| 378 STRABISMUS AND OTHER DISORDERS OF BINOCULAR EYE MOVEMENTS* \| \| 378.0 CONVERGENT CONCOMITANT STRABISMUS* \| \| 378.1 DIVERGENT CONCOMITANT STRABISMUS (ICD9CM: Extropia)* \| \| 378.2 INTERMITTENT HETEROTROPIA* \| \| 378.3 OTHER AND UNSPECIFIED HETEROTROPIA* \| \| 378.4 HETEROPHORIA* \| \| 378.5 PARALYTIC STRABISMUS* \| \| 378.6 MECHANICAL STRABISMUS* \| \| 378.7 OTHER STRABISMUS* \| \| 378.8 OTHER DISORDERS OF BINOCULAR EYE MOVEMENTS* \| \| 378.9 UNSPECIFIED* \| \|  \| \| PERSONS WITH CONDITIONS INFLUENCING THEIR HEALTH STATUS \| \| V41.0 PROBLEMS WITH SIGHT \| \|  \| \| PERSONS ENCOUNTERING HEALTH SERVICES FOR SPECIFIC PROCEDURES AND AFTER \| \| V52.2 ARTIFICIAL EYE \| \| V53.1 SPECTACLES AND CONTACT LENSES \| \|  \| \| PERSONS WITHOUT REPORTED DIAGNOSIS ENCOUNTERED DURING EXAMINATION AND INVESTIGATION OF INDIVIDALS  AND POPULATIONS \| \| V72.0 EXAMINATION OF EYES AND VISION \| \|  \| \| Amaurosis fugax \| \| Blindness \| \| Blurred vision \| \| Blurry eyes \| \| Blurry vision \| \| Cataract \| \| Deteriorating vision \| \| Double vision \| \| Early cataract \| \| Eye blurred \| \| Eye blurry \| \| Eye vision change \| \| Eyesight blurry \| \| Hemianopsia \| \| Hyperopia \| \| Low vision \| \| Macular degeneration \| \| Poor eyesight \| \| Retinopathy \| \| Sudden vision loss \| \| Trouble with vision \| \| Vision blurred \| \| Vision change \| \| Vision deteriorating \| \| Vision difficulties \| \| Vision issues \| \| Vision loss \| \| Vision problem \| \| Visual abnormalities \| \| Visual acuity decreased \| \| Visual disturbance \| \| Visual field defect \| \| Visual impairment \| \| \| --- \| --- \| --- \| --- \| --- \| --- \| --- \| --- \| --- \| --- \| --- \| --- \| --- \| --- \| --- \| --- \| --- \| --- \| --- \| --- \| --- \| --- \| --- \| --- \| --- \| --- \| --- \| --- \| --- \| --- \| --- \| --- \| --- \| --- \| --- \| --- \| --- \| --- \| --- \| --- \| --- \| --- \| --- \| --- \| --- \| --- \| --- \| --- \| --- \| --- \| --- \| --- \| --- \| --- \| --- \| --- \| --- \| --- \| --- \| --- \| --- \| --- \| --- \| --- \| --- \| --- \| --- \| --- \| --- \| --- \| --- \| --- \| --- \| --- \| --- \| --- \| --- \| --- \| --- \| --- \| --- \| --- \| --- \| --- \| --- \| --- \| --- \| --- \| --- \| --- \| --- \| --- \| --- \| --- \| --- \| --- \| --- \| --- \| --- \| --- \| --- \| --- \| --- \| --- \| --- \| --- \| --- \| --- \| --- \| --- \| --- \| --- \| --- \| --- \| --- \| --- \| --- \| --- \| --- \| --- \| --- \| --- \| --- \| --- \| --- \| --- \| --- \| --- \| --- \| --- \| --- \| --- \| --- \| --- \| --- \| --- \| --- \| --- \| --- \| --- \| --- \| --- \| --- \| --- \| --- \| --- \| --- \| --- \| --- \| --- \| --- \| --- \| --- \| --- \| \|  \| | \| **ICD9** \| \| --- \| \| **ICD9/TERMWORKS** \| \| **ICD9/TERMWORKS** \| \|  \| \| **ICD9/TERMWORKS** \| \| **ICD9/TERMWORKS** \| \| **ICD9/TERMWORKS** \| \| **ICD9/TERMWORKS** \| \| **ICD9/TERMWORKS** \| \| **ICD9/TERMWORKS** \| \| **ICD9/TERMWORKS** \| \|  \| \| **ICD9/TERMWORKS** \| \| **ICD9/TERMWORKS** \| \| **ICD9/TERMWORKS** \| \| **ICD9/TERMWORKS** \| \| **ICD9/TERMWORKS** \| \| **ICD9/TERMWORKS** \| \| **ICD9/TERMWORKS** \| \| **ICD9/TERMWORKS** \| \| **ICD9/TERMWORKS** \| \| **ICD9/TERMWORKS** \| \| **ICD9/TERMWORKS** \| \|  \| \| **ICD9/TERMWORKS** \| \| **ICD9/TERMWORKS** \| \| **ICD9/TERMWORKS** \| \| **ICD9/TERMWORKS** \| \| **ICD9/TERMWORKS** \| \| **ICD9/TERMWORKS** \| \| **ICD9/TERMWORKS** \| \| **ICD9-CM/TERMWORKS** \| \|  \| \| **ICD9** \| \| **ICD9** \| \| **ICD9** \| \| **ICD9** \| \| **ICD9** \| \| **ICD9** \| \| **ICD9** \| \| **ICD9** \| \|  \| \| **ICD9/TERMWORKS** \| \| **ICD9** \| \| **ICD9/TERMWORKS** \| \| **ICD9** \| \| **ICD9/TERMWORKS** \| \| **ICD9/TERMWORKS** \| \| **ICD9/TERMWORKS** \| \| **ICD9/TERMWORKS** \| \| **ICD9/TERMWORKS** \| \| **ICD9/TERMWORKS** \| \|  \| \| **ICD9/TERMWORKS** \| \| **ICD9/TERMWORKS** \| \| **ICD9/TERMWORKS** \| \| **ICD9/TERMWORKS** \| \| **ICD9/TERMWORKS** \| \| **ICD9/TERMWORKS** \| \| **ICD9/TERMWORKS** \| \| **ICD9/TERMWORKS** \| \| **ICD9/TERMWORKS** \| \| **ICD9/TERMWORKS** \| \|  \| \| **ICD9** \| \| **ICD9** \| \| **ICD9** \| \| **ICD9** \| \| **ICD9** \| \| **ICD9** \| \| **ICD9** \| \| **ICD9** \| \| **ICD9** \| \| **ICD9** \| \|  \| \| **ICD9** \| \| **ICD9** \| \| **ICD9** \| \| **ICD9** \| \| **ICD9** \| \| **ICD9** \| \| **ICD9** \| \| **ICD9** \| \| **ICD9** \| \| **ICD9** \| \| **ICD9** \| \|  \| \| **ICD9/TERMWORKS** \| \| **ICD9** \| \| **ICD9/TERMWORKS** \| \| **ICD9/TERMWORKS** \| \| **ICD9/TERMWORKS** \| \| **ICD9/TERMWORKS** \| \| **ICD9/TERMWORKS** \| \| **ICD9/TERMWORKS** \| \| **ICD9/TERMWORKS** \| \| **ICD9/TERMWORKS** \| \|  \| \| **ICD9** \| \| **ICD9** \| \| **ICD9** \| \| **ICD9** \| \| **ICD9** \| \| **ICD9** \| \| **ICD9** \| \| **ICD9** \| \| **ICD9** \| \| **ICD9** \| \| **ICD9** \| \|  \| \|  \| \| **ICD9/TERMWORKS** \| \|  \| \|  \| \| **ICD9** \| \| **ICD9/TERMWORKS** \| \|  \| \|  \| \|  \| \| **ICD9/TERMWORKS** \| \|  \| \| **CPCSSN Free Text** \| \| **CPCSSN Free Text** \| \| **CPCSSN Free Text** \| \| **CPCSSN Free Text** \| \| **CPCSSN Free Text** \| \| **CPCSSN Free Text** \| \| **CPCSSN Free Text** \| \| **CPCSSN Free Text** \| \| **CPCSSN Free Text** \| \| **CPCSSN Free Text** \| \| **CPCSSN Free Text** \| \| **CPCSSN Free Text** \| \| **CPCSSN Free Text** \| \| **CPCSSN Free Text** \| \| **CPCSSN Free Text** \| \| **CPCSSN Free Text** \| \| **CPCSSN Free Text** \| \| **CPCSSN Free Text** \| \| **CPCSSN Free Text** \| \| **CPCSSN Free Text** \| \| **CPCSSN Free Text** \| \| **CPCSSN Free Text** \| \| **CPCSSN Free Text** \| \| **CPCSSN Free Text** \| \| **CPCSSN Free Text** \| \| **CPCSSN Free Text** \| \| **CPCSSN Free Text** \| \| **CPCSSN Free Text** \| \| **CPCSSN Free Text** \| \| **CPCSSN Free Text** \| \| **CPCSSN Free Text** \| \| **CPCSSN Free Text** \| \| **CPCSSN Free Text** \| |

Termworks search terms: vision, visual impairment, cataract, glasses, macula, blind, blindness, retinopathy, macular degeneration, macular, drusen, retinal, visual, optic, Vitelliform dystrophy, sight

## Weight loss and/or anorexia

| **Codes and Terms** | **Source(s)** |
| --- | --- |
| \| \| NEUROTIC DISORDERS, PERSONALITY DISORDERS AND OTHER NONPSYCHOTIC MENTAL DISORDERS \| \| --- \| \| 307.1 ANOREXIA NERVOSA \| \|  \| \| SYMPTOMS, SIGNS AND ILL-DEFINED CONDITIONS \| \| 783.0 ANOREXIA \| \| 783.2 ABNORMAL LOSS OF WEIGHT (and underweight) \| \| 783.21 Loss of weight \| \| 783.22 Underweight \| \| 783.3 FEEDING DIFFICULTIES AND MISMANAGEMENT \| \|  \| \| 799.4 CACHEXIA \| \|  \| \| V65.3 DIETARY SURVEILLANCE AND COUNSELLING \| \| V69.1 Inappropriate diet and eating habits \| \|  \| \| SPECIAL SCREENING FOR ENDOCRINE, NUTRITIONAL, METABOLIC AND IMMUNITY \| \| V77.2 MALNUTRITION \| \|  \| \| V85.0 Body Mass Index less than 19, adult \| \|  \| \| ADDITIONAL DIAGNOSTIC CODES \| \| 07A FEEDING PROBLEM \| \|  \| \| Acute weight loss \| \| Anorexia \| \| Anorexia nervosa \| \| Appetite decreased \| \| Lost weight \| \| Malnourished \| \| Malnutrition \| \| Malnutrition chronic \| \| No appetite \| \| Poor appetite \| \| Poor nutrition \| \| Underweight \| \| Unexplained weight loss \| \| Unwanted weight loss \| \| Weight loss \| \| Weight loss abnormal \| \| Wt loss \| \|  \| \| \| --- \| --- \| --- \| --- \| --- \| --- \| --- \| --- \| --- \| --- \| --- \| --- \| --- \| --- \| --- \| --- \| --- \| --- \| --- \| --- \| --- \| --- \| --- \| --- \| --- \| --- \| --- \| --- \| --- \| --- \| --- \| --- \| --- \| --- \| --- \| --- \| --- \| --- \| --- \| --- \| --- \| --- \| | \| **ICD9/TERMWORKS** \| \| --- \| \|  \| \|  \| \| **ICD9/TERMWORKS** \| \| **ICD9/TERMWORKS** \| \| **ICD9-CM/TERMWORKS** \| \| **ICD9-CM** \| \| **ICD9** \| \|  \| \| **ICD9** \| \|  \| \| **ICD9** \| \| **ICD9-CM** \| \|  \| \|  \| \| **ICD9** \| \|  \| \| **ICD9-CM** \| \|  \| \|  \| \| **ICD9** \| \|  \| \| **CPCSSN Free Text** \| \| **CPCSSN Free Text** \| \| **CPCSSN Free Text** \| \| **CPCSSN Free Text** \| \| **CPCSSN Free Text** \| \| **CPCSSN Free Text** \| \| **CPCSSN Free Text** \| \| **CPCSSN Free Text** \| \| **CPCSSN Free Text** \| \| **CPCSSN Free Text** \| \| **CPCSSN Free Text** \| \| **CPCSSN Free Text** \| \| **CPCSSN Free Text** \| \| **CPCSSN Free Text** \| \| **CPCSSN Free Text** \| \| **CPCSSN Free Text** \| \| **CPCSSN Free Text** \| |

Termworks search terms: weight loss, appetite, weight, anorexia

# Additional Frailty Factors Suggested by Panelists (n=13)

## Cancer

| **Codes** | **Source(s)** |
| --- | --- |
| \| \| MALIGNANT NEOPLASM OF LIP, ORAL CAVITY AND PHARYNX \| \| --- \| \| 140 MALIGNANT NEOPLASM OF LIP* \| \| 141 MALIGNANT NEOPLASM OF TONGUE* \| \| 142 MALIGNANT NEOPLASM OF MAJOR SALIVARY GLANDS* \| \| 143 MALIGNANT NEOPLASM OF GUM* \| \| 144 MALIGNANT NEOPLASM OF FLOOR OF MOUTH* \| \| 145 MALIGNANT NEOPLASM OF OTHER AND UNSPECIFIED PARTS OF MOUTH* \| \| 146 MALIGNANT NEOPLASM OF OROPHARYNX* \| \| 147 MALIGNANT NEOPLASM OF NASOPHARYNX* \| \| 148 MALIGNANT NEOPLASM OF HYPOPHARYNX* \| \| 149 MALIGNANT NEOPLASM OF OTHER AND ILL-DEFINED SITES WITHIN THE LIP, ORAL CAVITY AND PHARYNX* \| \|  \| \| MALIGNANT NEOPLASM OF DIGESTIVE ORGANS AND PERITONEUM \| \| 150 MALIGNANT NEOPLASM OF OESOPHAGUS* \| \| 151 MALIGNANT NEOPLASM OF STOMACH* \| \| 152 MALIGNANT NEOPLASM OF SMALL INTESTINE, INCLUDING DUODENUM* \| \| 153 MALIGNANT NEOPLASM OF COLON* \| \| 154 MALIGNANT NEOPLASM OF RECTUM, RECTOSIGMOID JUNCTION AND ANUS* \| \| 155 MALIGNANT NEOPLASM OF LIVER AND INTRAHEPATIC BILE DUCTS* \| \| 156 MALIGNANT NEOPLASM OF GALLBLADDER AND EXTRAHEPATIC BILE DUCTS* \| \| 157 MALIGNANT NEOPLASM OF PANCREAS* \| \| 158 MALIGNANT NEOPLASM OF RETROPERITONEUM AND PERITONEUM* \| \| 159 MALIGNANT NEOPLASM OF OTHER AND ILL-DEFINED SITES WITHIN THE DIGESTIVE ORGANS AND PERITONEUM* \| \|  \| \| MALIGNANT NEOPLASM OF RESPIRATORY AND INTRATHORACIC ORGANS \| \| 160 MALIGNANT NEOPLASM OF NASAL CAVITIES, MIDDLE EAR AND ACCESSORY SINUSES* \| \| 161 MALIGNANT NEOPLASM OF LARYNX* \| \| 162 MALIGNANT NEOPLASM OF TRACHEA, BRONCHUS AND LUNG* \| \| 163 MALIGNANT NEOPLASM OF PLEURA* \| \| 164 MALIGNANT NEOPLASM OF THYMUS, HEART AND MEDIASTINUM* \| \| 165 MALIGNANT NEOPLASM OF OTHER AND ILL-DEFINED SITES WITHIN THE RESPIRATORY SYSTEM AND INTRATHORACIC ORGANS* \| \|  \| \| MALIGNANT NEOPLASM OF BONE, CONNECTIVE TISSUE, SKIN AND BREAST \| \| 170 MALIGNANT NEOPLASM OF BONE AND ARTICULAR CARTILAGE* \| \| 171 MALIGNANT NEOPLASM OF CONNECTIVE AND OTHER SOFT TISSUE* \| \| 172 MALIGNANT MELANOMA OF SKIN* \| \| 173 OTHER MALIGNANT NEOPLASM OF SKIN* \| \| 174 MALIGNANT NEOPLASM OF FEMALE BREAST* \| \| 175 MALIGNANT NEOPLASM OF MALE BREAST* \| \| 176 Kaposi's sarcoma* \| \|  \| \| MALIGNANT NEOPLASM OF GENITOURINARY ORGANS \| \| 179 MALIGNANT NEOPLASM OF UTERUS, PART UNSPECIFIED* \| \| 180 MALIGNANT NEOPLASM OF CERVIX UTERI* \| \| 181 MALIGNANT NEOPLASM OF PLACENTA* \| \| 182 MALIGNANT NEOPLASM OF BODY OF UTERUS* \| \| 183 MALIGNANT NEOPLASM OF OVARY AND OTHER UTERINE ADNEXA* \| \| 184. MALIGNANT NEOPLASM OF OTHER AND UNSPECIFIED FEMALE GENITAL ORGANS* \| \| 185 MALIGNANT NEOPLASM OF PROSTATE* \| \| 186 MALIGNANT NEOPLASM OF TESTIS* \| \| 187 MALIGNANT NEOPLASM OF PENIS AND OTHER MALE GENITAL ORGANS* \| \| 188 MALIGNANT NEOPLASM OF BLADDER* \| \| 189 MALIGNANT NEOPLASM OF KIDNEY AND OTHER AND UNSPECIFIED URINARY ORGANS* \| \|  \| \| MALIGNANT NEOPLASM OF OTHER AND UNSPECIFIED SITES \| \| 190 MALIGNANT NEOPLASM OF EYE* \| \| 191 MALIGNANT NEOPLASM OF BRAIN* \| \| 192 MALIGNANT NEOPLASM OF OTHER AND UNSPECIFIED PARTS OF NERVOUS SYSTEM* \| \| 193 MALIGNANT NEOPLASM OF THYROID GLAND* \| \| 194 MALIGNANT NEOPLASM OF OTHER ENDOCRINE GLANDS AND RELATED STRUCTURES* \| \| 195 MALIGNANT NEOPLASM OF OTHER AND ILL-DEFINED SITES* \| \| 196 SECONDARY AND UNSPECIFIED MALIGNANT NEOPLASM OF LYMPH NODES* \| \| 197 SECONDARY MALIGNANT NEOPLASM OF RESPIRATORY AND DIGESTIVE SYSTEMS* \| \| 198 SECONDARY MALIGNANT NEOPLASM OF OTHER SPECIFIED SITES* \| \| 199 MALIGNANT NEOPLASM WITHOUT SPECIFICATION OF SITE* \| \|  \| \| MALIGNANT NEOPLASM OF LYMPHATIC AND HAEMATOPOIETIC TISSUE \| \| 200 LYMPHOSARCOMA AND RETICULOSARCOMA* \| \| 201 HODGKIN'S DISEASE* \| \| 202 OTHER MALIGNANT NEOPLASM OF LYMPHOID AND HISTIOCYTIC TISSUE* \| \| 203 MULTIPLE MYELOMA AND IMMUNOPROLIFERATIVE NEOPLASMS* \| \| 204 LYMPHOID LEUKAEMIA* \| \| 205 MYELOID LEUKAEMIA* \| \| 206 MONOCYTIC LEUKAEMIA* \| \| 207 OTHER SPECIFIED LEUKAEMIA* \| \| 208 LEUKAEMIA OF UNSPECIFIED CELL TYPE* \| \| 209 Neuroendocrine tumors* \| \|  \| \| CARCINOMA IN SITU \| \| 230 CARCINOMA IN SITU OF DIGESTIVE ORGANS* \| \| 231 CARCINOMA IN SITU OF RESPIRATORY SYSTEM* \| \| 232 CARCINOMA IN SITU OF SKIN* \| \| 233 CARCINOMA IN SITU OF BREAST AND GENITOURINARY SYSTEM* \| \| 234 CARCINOMA IN SITU OF OTHER AND UNSPECIFIED SITES* \| \|  \| \| NEOPLASMS OF UNCERTAIN BEHAVIOUR \| \| 235 NEOPLASM OF UNCERTAIN BEHAVIOUR OF DIGESTIVE AND RESPIRATORY SYSTEMS* \| \| 236 NEOPLASM OF UNCERTAIN BEHAVIOUR OF GENITOURINARY ORGANS* \| \| 237 NEOPLASM OF UNCERTAIN BEHAVIOUR OF ENDOCRINE GLANDS AND NERVOUS SYSTEM* \| \| 238 NEOPLASM OF UNCERTAIN BEHAVIOUR OF OTHER AND UNSPECIFIED SITES AND TISSUES* \| \|  \| \| NEOPLASMS OF UNSPECIFIED NATURE \| \| 239 NEOPLASM OF UNSPECIFIED NATURE* \| \| 239.0 DIGESTIVE SYSTEM* \| \| 239.1 RESPIRATORY SYSTEM* \| \| 239.2 BONE, SOFT TISSUE AND SKIN* \| \| 239.3 BREAST* \| \| 239.4 BLADDER* \| \| 239.5 OTHER GENITOURINARY ORGANS* \| \| 239.6 BRAIN* \| \| 239.7 ENDOCRINE GLANDS, AND OTHER PARTS OF NERVOUS SYSTEM* \| \| 239.8 OTHER SPECIFIED SITES* \| \| 239.9 SITE UNSPECIFIED* \| \|  \| \| 793.80 Abnormal mammogram, unspecified \| \| 795.8 Abnormal tumor markers \| \| 795.81 Elevated carcinoembryonic antigen [CEA] \| \| 795.82 Elevated cancer antigen 125 [CA 125] \| \| 795.89 Other abnormal tumor markers \| \|  \| \| PERSONS WITH POTENTIAL HEALTH HAZARDS RELATED TO PERSONAL AND FAMILY HISTORY \| \| V10 PERSONAL HISTORY OF MALIGNANT NEOPLASM* \| \| V10.0 GASTROINTESTINAL TRACT* \| \| V10.1 TRACHEA, BRONCHUS AND LUNG* \| \| V10.2 OTHER RESPIRATORY AND INTRATHORACIC ORGANS* \| \| V10.3 BREAST* \| \| V10.4 GENITAL ORGANS* \| \| V10.5 URINARY ORGANS* \| \| V10.6 LEUKAEMIA* \| \| V10.7 OTHER LYMPHATIC AND HAEMATOPOIETIC NEOPLASMS* \| \| V10.8 OTHER* \| \| V10.9 UNSPECIFIED* \| \|  \| \| V71.1 OBSERVATION FOR SUSPECTED MALIGNANT NEOPLASM \| \|  \| \| V76 SPECIAL SCREENING FOR MALIGNANT NEOPLASMS \| \| V76.0 RESPIRATORY ORGANS \| \| V76.1 BREAST \| \| V76.10 Breast screening, unspecified \| \| V76.11 Screening mammogram for high-risk patient \| \| V76.12 Other screening mammogram \| \| V76.19 Other screening breast examination \| \| V76.2 CERVIX \| \| V76.3 BLADDER \| \| V76.4 OTHER SITES \| \| V76.41 Screening for malignant neoplasms of rectum \| \| V76.42 Screening for malignant neoplasms of oral cavity \| \| V76.43 Screening for malignant neoplasms of skin \| \| V76.44 Screening for malignant neoplasms of prostate \| \| V76.45 Screening for malignant neoplasms of testis \| \| V76.46 Special screening for malignant neoplasms of ovary \| \| V76.47 Special screening for malignant neoplasms of vagina \| \| V76.49 Special screening for malignant neoplasms of other sites \| \| V76.5 Special screening for malignant neoplasms of intestine \| \| V76.50 Special screening for malignant neoplasms for intestine, unspecified \| \| V76.51 Special screening for malignant neoplasms of colon \| \| V76.52 Special screening for malignant neoplasms of small intestine \| \| V76.8 OTHER NEOPLASM \| \| V76.81 Special screening for malignant neoplasms of nervous system \| \| V76.89 Special screening for other malignant neoplasms \| \| V76.9 UNSPECIFIED \| \|  \| \| V84.0 Genetic susceptibility to malignant neoplasm \| \| V84.01 Genetic susceptibility to malignant neoplasm of breast \| \| V84.02 Genetic susceptibility to malignant neoplasm of ovary \| \| V84.03 Genetic susceptibility to malignant neoplasm of prostate \| \| V84.04 Genetic susceptibility to malignant neoplasm of endometrium \| \| V84.09 Genetic susceptibility to other malignant neoplasm \| \|  \| \| **Carcinoembryonic Ag (CEA) (ug/L)** \| \| >2.5 ng/mL \| \|  \| \| Cancer \| \| Cancer spreading \| \| Carcinoma \| \| Leiomyoma \| \| Leukemia \| \| Lipoma \| \| Lymphoma \| \| Malignancy \| \| Melanoma \| \| Meningioma \| \| Metastases \| \| Metastatic \| \| Metastatic cancer \| \| Thymoma \| \| Tumor \| \| Tumour \| \| \| --- \| --- \| --- \| --- \| --- \| --- \| --- \| --- \| --- \| --- \| --- \| --- \| --- \| --- \| --- \| --- \| --- \| --- \| --- \| --- \| --- \| --- \| --- \| --- \| --- \| --- \| --- \| --- \| --- \| --- \| --- \| --- \| --- \| --- \| --- \| --- \| --- \| --- \| --- \| --- \| --- \| --- \| --- \| --- \| --- \| --- \| --- \| --- \| --- \| --- \| --- \| --- \| --- \| --- \| --- \| --- \| --- \| --- \| --- \| --- \| --- \| --- \| --- \| --- \| --- \| --- \| --- \| --- \| --- \| --- \| --- \| --- \| --- \| --- \| --- \| --- \| --- \| --- \| --- \| --- \| --- \| --- \| --- \| --- \| --- \| --- \| --- \| --- \| --- \| --- \| --- \| --- \| --- \| --- \| --- \| --- \| --- \| --- \| --- \| --- \| --- \| --- \| --- \| --- \| --- \| --- \| --- \| --- \| --- \| --- \| --- \| --- \| --- \| --- \| --- \| --- \| --- \| --- \| --- \| --- \| --- \| --- \| --- \| --- \| --- \| --- \| --- \| --- \| --- \| --- \| --- \| --- \| --- \| --- \| --- \| --- \| --- \| --- \| --- \| --- \| --- \| --- \| --- \| --- \| --- \| --- \| --- \| --- \| --- \| --- \| --- \| --- \| --- \| --- \| --- \| --- \| --- \| --- \| --- \| --- \| --- \| --- \| --- \| --- \| --- \| --- \| --- \| --- \| --- \| --- \| --- \| --- \| --- \| --- \| --- \| --- \| --- \| --- \| --- \| \|  \| | \| **ICD9/TERMWORKS** \| \| --- \| \| **ICD9/TERMWORKS** \| \| **ICD9/TERMWORKS** \| \| **ICD9/TERMWORKS** \| \| **ICD9/TERMWORKS** \| \| **ICD9/TERMWORKS** \| \| **ICD9/TERMWORKS** \| \| **ICD9/TERMWORKS** \| \| **ICD9/TERMWORKS** \| \| **ICD9/TERMWORKS** \| \|  \| \|  \| \| **ICD9/TERMWORKS** \| \| **ICD9/TERMWORKS** \| \| **ICD9/TERMWORKS** \| \| **ICD9/TERMWORKS** \| \| **ICD9/TERMWORKS** \| \| **ICD9/TERMWORKS** \| \| **ICD9/TERMWORKS** \| \| **ICD9/TERMWORKS** \| \| **ICD9/TERMWORKS** \| \| **ICD9/TERMWORKS** \| \|  \| \|  \| \| **ICD9/TERMWORKS** \| \| **ICD9/TERMWORKS** \| \| **ICD9/TERMWORKS** \| \| **ICD9/TERMWORKS** \| \| **ICD9/TERMWORKS** \| \| **ICD9/TERMWORKS** \| \|  \| \|  \| \| **ICD9/TERMWORKS** \| \| **ICD9/TERMWORKS** \| \| **ICD9/TERMWORKS** \| \| **ICD9/TERMWORKS** \| \| **ICD9/TERMWORKS** \| \| **ICD9/TERMWORKS** \| \| **ICD9-CM** \| \|  \| \|  \| \| **ICD9/TERMWORKS** \| \| **ICD9/TERMWORKS** \| \| **ICD9/TERMWORKS** \| \| **ICD9/TERMWORKS** \| \| **ICD9/TERMWORKS** \| \| **ICD9/TERMWORKS** \| \| **ICD9/TERMWORKS** \| \| **ICD9/TERMWORKS** \| \| **ICD9/TERMWORKS** \| \| **ICD9/TERMWORKS** \| \| **ICD9/TERMWORKS** \| \|  \| \|  \| \| **ICD9/TERMWORKS** \| \| **ICD9/TERMWORKS** \| \| **ICD9/TERMWORKS** \| \| **ICD9/TERMWORKS** \| \| **ICD9/TERMWORKS** \| \| **ICD9/TERMWORKS** \| \| **ICD9/TERMWORKS** \| \| **ICD9/TERMWORKS** \| \| **ICD9/TERMWORKS** \| \| **ICD9/TERMWORKS** \| \|  \| \|  \| \| **ICD9/TERMWORKS** \| \| **ICD9** \| \| **ICD9/TERMWORKS** \| \| **ICD9** \| \| **ICD9** \| \| **ICD9** \| \| **ICD9** \| \| **ICD9** \| \| **ICD9** \| \| **ICD9-CM/TERMWORKS** \| \|  \| \|  \| \| **ICD9** \| \| **ICD9** \| \| **ICD9** \| \| **ICD9** \| \| **ICD9** \| \|  \| \|  \| \| **ICD9** \| \| **ICD9** \| \| **ICD9** \| \| **ICD9** \| \|  \| \|  \| \| **ICD9** \| \| **ICD9** \| \| **ICD9** \| \| **ICD9** \| \| **ICD9** \| \| **ICD9** \| \| **ICD9** \| \| **ICD9** \| \| **ICD9** \| \| **ICD9** \| \| **ICD9** \| \|  \| \| **ICD9-CM** \| \| **ICD9-CM** \| \| **ICD9-CM** \| \| **ICD9-CM** \| \| **ICD9-CM** \| \|  \| \|  \| \| **ICD9/TERMWORKS** \| \| **ICD9** \| \| **ICD9** \| \| **ICD9** \| \| **ICD9/TERMWORKS** \| \| **ICD9** \| \| **ICD9** \| \| **ICD9** \| \| **ICD9** \| \| **ICD9/TERMWORKS** \| \| **ICD9/TERMWORKS** \| \|  \| \| **ICD9/TERMWORKS** \| \|  \| \| **ICD9/TERMWORKS** \| \| **ICD9/TERMWORKS** \| \| **ICD9** \| \| **ICD9-CM** \| \| **ICD9-CM** \| \| **ICD9-CM** \| \| **ICD9-CM** \| \| **ICD9/TERMWORKS** \| \| **ICD9/TERMWORKS** \| \| **ICD9** \| \| **ICD9-CM** \| \| **ICD9-CM** \| \| **ICD9-CM** \| \| **ICD9-CM** \| \| **ICD9-CM** \| \| **ICD9-CM** \| \| **ICD9-CM** \| \| **ICD9-CM** \| \| **ICD9-CM** \| \| **ICD9-CM** \| \| **ICD9-CM** \| \| **ICD9-CM** \| \| **ICD9** \| \| **ICD9-CM** \| \| **ICD9-CM** \| \| **ICD9/TERMWORKS** \| \|  \| \| **ICD9-CM** \| \| **ICD9-CM** \| \| **ICD9-CM** \| \| **ICD9-CM** \| \| **ICD9-CM** \| \| **ICD9-CM** \| \|  \| \| **LOINC 2039-6** \| \|  \| \|  \| \| **CPCSSN Free Text** \| \| **CPCSSN Free Text** \| \| **CPCSSN Free Text** \| \| **CPCSSN Free Text** \| \| **CPCSSN Free Text** \| \| **CPCSSN Free Text** \| \| **CPCSSN Free Text** \| \| **CPCSSN Free Text** \| \| **CPCSSN Free Text** \| \| **CPCSSN Free Text** \| \| **CPCSSN Free Text** \| \| **CPCSSN Free Text** \| \| **CPCSSN Free Text** \| \| **CPCSSN Free Text** \| \| **CPCSSN Free Text** \| \| **CPCSSN Free Text** \| \|  \| |

Termworks search terms: cancer, malignant, tumor

## Challenges to healthcare access

Panelists’ Descriptions:

| -        Inconsistency in finding and accessing medical services |
| --- |
| -        Lack of transportation |
| -        Anyone without a health care advocate (meaning someone to accompany to appointments, pick up prescriptions, wellness checks etc) |
| -        Physical isolation (living far out, no access to transport) |
| -        No primary care provider/lack of family doctor |
| -        Access to regular health care provider |
| -        Lack of a family doctor |

| **Codes** | **Source(s)** |
| --- | --- |
| \| \| V63.0 RESIDENCE REMOTE FROM HOSPITAL OR OTHER HEALTH CARE FACILITY \| \| --- \| \| V63.1 MEDICAL SERVICES IN HOME NOT AVAILABLE \| \| \| --- \| --- \| --- \| \|  \| | \| **ICD9** \| \| --- \| \| **TERMWORKS** \| |

Termworks search terms: Access, resources, transportation, services, advocate, isolation, physician

## Chronic pain/back pain

| **Codes** | **Source(s)** |
| --- | --- |
| \| \| 307.8 PAIN DISORDERS RELATED TO PSYCHOLOGICAL FACTORS (PSYCHALGIA) \| \| --- \| \| 307.80 Psychogenic pain, site unspecified \| \| 307.89 Other pain disorders related to psychological factors \| \|  \| \| 338 Pain, not elsewhere classified \| \| 338.0 Central pain syndrome \| \| 338.2 Chronic pain \| \| 338.21 Chronic pain due to trauma \| \| 338.22 Chronic post-thoracotomy pain \| \| 338.28 Other chronic postoperative pain \| \| 338.29 Other chronic pain \| \| 338.3 Neoplasm related pain (acute) (chronic) \| \| 338.4 Chronic pain syndrome \| \|  \| \| 719.4 PAIN IN JOINT (Under 719 Other and Unspecified Disorder of Joint) \| \| 719.40 Pain in joint, site unspecified \| \| 719.41 Pain in joint, shoulder region \| \| 719.42 Pain in joint, upper arm \| \| 719.43 Pain in joint, forearm \| \| 719.44 Pain in joint, hand \| \| 719.45 Pain in joint, pelvic region and thigh \| \| 719.46 Pain in joint, lower leg \| \| 719.47 Pain in joint, ankle and foot \| \| 719.48 Pain in joint, other specified sites \| \| 719.49 Pain in joint, multiple sites \| \|  \| \| 724.1 PAIN IN THORACIC SPINE (Under 724 Other and Unspecified Disorders of Back) \| \| 724.2 LUMBAGO \| \| 724.3 SCIATICA \| \| 724.5 BACKACHE, UNSPECIFIED \| \|  \| \| 729.5 PAIN IN LIMB (Under 729 Other Disorders of Soft Tissues) \| \|  \| \| 780.96 Generalized pain \| \|  \| \| Back pain \| \| Back problem \| \| Chronic back pain \| \| Chronic pain \| \| Chronic pain syndrome \| \| Complex pain \| \| Fibromyalgia \| \| Muscle pain \| \| Myalgia \| \| Nerve pain \| \| Neuralgia \| \| Neuropathic pain \| \| Pain chronic \| \| Pain chronic management \| \| Pain management \| \| PMR \| \| Polymyalgia rheumatica \| \| Polyneuropathy \| \| Sciatica \| \| Trigger point injection \| \| \| --- \| --- \| --- \| --- \| --- \| --- \| --- \| --- \| --- \| --- \| --- \| --- \| --- \| --- \| --- \| --- \| --- \| --- \| --- \| --- \| --- \| --- \| --- \| --- \| --- \| --- \| --- \| --- \| --- \| --- \| --- \| --- \| --- \| --- \| --- \| --- \| --- \| --- \| --- \| --- \| --- \| --- \| --- \| --- \| --- \| --- \| --- \| --- \| --- \| --- \| --- \| --- \| --- \| --- \| --- \| --- \| \|  \| | \| **TERMWORKS** \| \| --- \| \| **ICD9-CM/TERMWORKS** \| \| **ICD9-CM/TERMWORKS** \| \|  \| \| **ICD9-CM/TERMWORKS** \| \| **ICD9-CM/TERMWORKS** \| \| **ICD9-CM/TERMWORKS** \| \| **ICD9-CM/TERMWORKS** \| \| **ICD9-CM/TERMWORKS** \| \| **ICD9-CM/TERMWORKS** \| \| **ICD9-CM/TERMWORKS** \| \| **ICD9-CM/TERMWORKS** \| \| **ICD9-CM/TERMWORKS** \| \|  \| \| **ICD9/TERMWORKS** \| \| **ICD9-CM/TERMWORKS** \| \| **ICD9-CM/TERMWORKS** \| \| **ICD9-CM/TERMWORKS** \| \| **ICD9-CM/TERMWORKS** \| \| **ICD9-CM/TERMWORKS** \| \| **ICD9-CM/TERMWORKS** \| \| **ICD9-CM/TERMWORKS** \| \| **ICD9-CM/TERMWORKS** \| \| **ICD9-CM/TERMWORKS** \| \| **ICD9-CM/TERMWORKS** \| \|  \| \| **ICD9/TERMWORKS** \| \| **ICD9** \| \| **ICD9** \| \| **ICD9** \| \|  \| \| **ICD9/TERMWORKS** \| \|  \| \| **ICD9-CM/TERMWORKS** \| \|  \| \| **CPCSSN Free Text** \| \| **CPCSSN Free Text** \| \| **CPCSSN Free Text** \| \| **CPCSSN Free Text** \| \| **CPCSSN Free Text** \| \| **CPCSSN Free Text** \| \| **CPCSSN Free Text** \| \| **CPCSSN Free Text** \| \| **CPCSSN Free Text** \| \| **CPCSSN Free Text** \| \| **CPCSSN Free Text** \| \| **CPCSSN Free Text** \| \| **CPCSSN Free Text** \| \| **CPCSSN Free Text** \| \| **CPCSSN Free Text** \| \| **CPCSSN Free Text** \| \| **CPCSSN Free Text** \| \| **CPCSSN Free Text** \| \| **CPCSSN Free Text** \| \| **CPCSSN Free Text** \| |

Termworks search terms: pain

## Communication challenges

Panelists’ Descriptions:

| -        inability to communicate verbally-not able to advocate for themselves, needs, desires for ongoing health care and goals of care |
| --- |
| -        Handwriting deterioration |
| -        Language/cultural barriers |
| -        Low literacy |

| **Codes** | **Source(s)** |
| --- | --- |
| \| \| 784.3 APHASIA \| \| --- \| \|  \| \| 784.5 OTHER SPEECH DISTURBANCE \| \| 784.51 Dysarthria \| \| 784.52 Fluency disorder in conditions classified elsewhere \| \| 784.59 Other speech disturbance \| \| 799.52 Cognitive communication deficit \| \|  \| \| V40.1 PROBLEMS WITH COMMUNICATION (INCLUDING SPEECH) \| \| V41.4 PROBLEMS WITH VOICE PRODUCTION \| \|  \| \| Aphasia \| \| Speech disturbance \| \| \| --- \| --- \| --- \| --- \| --- \| --- \| --- \| --- \| --- \| --- \| --- \| --- \| --- \| --- \| \|  \| | \| **ICD9** \| \| --- \| \|  \| \| **ICD9** \| \| **ICD9-CM** \| \| **ICD9-CM** \| \| **ICD9-CM** \| \| **ICD9-CM/TERMWORKS** \| \|  \| \| **ICD9/TERMWORKS** \| \| **ICD9** \| \|  \| \| **CPCSSN Free Text** \| \| **CPCSSN Free Text** \| \|  \| |

Termworks search terms: communicate, language, literacy, nonverbal

## Fecal incontinence

| **Codes** | **Source(s)** |
| --- | --- |
| \| \| 787.6 INCONTINENCE OF FAECES \| \| --- \| \| 787.60 Full incontinence of feces \| \| 787.61 Incomplete defecation \| \| 787.62 Fecal smearing \| \| 787.63 Fecal urgency \| \|  \| \| 788.33 Mixed incontinence (male) (female) \| \|  \| \| Diarrhea – incontinence \| \| Fecal incontinence  Incontinence of feces  Incontinence of faeces \| \| \| --- \| --- \| --- \| --- \| --- \| --- \| --- \| --- \| --- \| --- \| --- \| \|  \| | \| **ICD9/TERMWORKS** \| \| --- \| \| **ICD9-CM/TERMWORKS** \| \| **ICD9-CM** \| \| **ICD9-CM/TERMWORKS** \| \| **ICD9-CM/TERMWORKS** \| \|  \| \| **ICD9-CM/TERMWORKS** \| \|  \| \| **CPCSSN Free Text** \| \| **CPCSSN Free Text** \| \| **CPCSSN Free Text** \| \| **CPCSSN Free Text** \| |

Termworks search terms: incontinence, feces, fecal

## Inadequate Diet and Nutrition

Panelists’ Descriptions:

| -        Noticeable lack of interest in meal preparation and meal planning |
| --- |
| -        Reduction in amount of fruit and vegetables in diet |
| -        Sense of taste and smell diminish |
| -        Food insecurity |

| **Codes** | **Source(s)** |
| --- | --- |
| \| \| NUTRITIONAL DEFICIENCES \| \| --- \| \| 263 OTHER AND UNSPECIFIED PROTEIN-CALORIE MALNUTRITION \| \| 263.0 MALNUTRITION OF MODERATE DEGREE \| \| 263.1 MALNUTRITION OF MILD DEGREE \| \|  \| \| 307.5 OTHER AND UNSPECIFIED DISORDERS OF EATING \| \| 307.50 Eating disorder, unspecified \| \| 307.51 Bulimia nervosa \| \| 307.52 Pica \| \| 307.53 Rumination disorder \| \| 307.54 Psychogenic vomiting \| \| 307.59 Other disorders of eating \| \|  \| \| 780.94 Early satiety \| \| 781.1 DISTURBANCES OF SENSATION OF SMELL AND TASTE \| \|  \| \| 787.2 DYSPHAGIA \| \| 787.20 Dysphagia, unspecified \| \| 787.21 Dysphagia, oral phase \| \| 787.22 Dysphagia, oropharyngeal phase \| \| 787.23 Dysphagia, pharyngeal phase \| \| 787.24 Dysphagia, pharyngoesophageal phase \| \| 787.29 Other dysphagia \| \|  \| \|  \| \| V12.1 NUTRITIONAL DEFICIENCY \| \| V41.5 PROBLEMS WITH SMELL AND TASTE \| \| V41.6 PROBLEMS WITH SWALLOWING AND MASTICATION \| \| V65.3 DIETARY SURVEILLANCE AND COUNSELLING \| \| V69.1 Inappropriate diet and eating habits \| \| V77.2 MALNUTRITION \| \|  \| \| Decreased appetite \| \| Dietician \| \| Diet poor \| \| Difficulty swallowing \| \| Diminished appetite \| \| Lost sense of smell \| \| Lost sense of taste \| \| No appetite \| \| Seen for nutrition \| \| Trouble eating \| \| \| --- \| --- \| --- \| --- \| --- \| --- \| --- \| --- \| --- \| --- \| --- \| --- \| --- \| --- \| --- \| --- \| --- \| --- \| --- \| --- \| --- \| --- \| --- \| --- \| --- \| --- \| --- \| --- \| --- \| --- \| --- \| --- \| --- \| --- \| --- \| --- \| --- \| --- \| --- \| --- \| --- \| --- \| --- \| \|  \| | \| **ICD9** \| \| --- \| \| **ICD9/TERMWORKS** \| \| **ICD9/TERMWORKS** \| \|  \| \| **ICD9/TERMWORKS** \| \| **ICD9-CM** \| \| **ICD9-CM** \| \| **ICD9-CM** \| \| **ICD9-CM** \| \| **ICD9-CM** \| \| **ICD9-CM** \| \|  \| \| **ICD9-CM** \| \| **ICD9/TERMWORKS** \| \|  \| \| **ICD9** \| \| **ICD9-CM** \| \| **ICD9-CM** \| \| **ICD9-CM** \| \| **ICD9-CM** \| \| **ICD9-CM** \| \| **ICD9-CM** \| \|  \| \|  \| \| **ICD9** \| \| **ICD9/TERMWORKS** \| \|  \| \| **ICD9** \| \| **ICD9-CM** \| \| **ICD9/TERMWORKS** \| \|  \| \| **CPCSSN Free Text** \| \| **CPCSSN Free Text** \| \| **CPCSSN Free Text** \| \| **CPCSSN Free Text** \| \| **CPCSSN Free Text** \| \| **CPCSSN Free Text** \| \| **CPCSSN Free Text** \| \| **CPCSSN Free Text** \| \| **CPCSSN Free Text** \| \| **CPCSSN Free Text** \| |

Termworks search terms: diet, taste, smell, food insecurity, malnutrition

## Liver failure/cirrhosis

| **Codes** | **Source(s)** |
| --- | --- |
| \| \| 570 ACUTE AND SUBACUTE NECROSIS OF LIVER \| \| --- \| \|  \| \| 571 CHRONIC LIVER DISEASE AND CIRRHOSIS \| \| 571.2 ALCOHOLIC CIRRHOSIS OF LIVER \| \| 571.3 ALCOHOLIC LIVER DAMAGE, UNSPECIFIED \| \| 571.5 CIRRHOSIS OF LIVER WITHOUT MENTION OF ALCOHOL \| \| 571.6 BILIARY CIRRHOSIS \| \|  \| \| NONSPECIFIC ABNORMAL RESULTS OF FUNCTION STUDIES \| \| 794.8 LIVER \| \|  \| \| Alcohol cirrhosis liver \| \| Chronic liver disease \| \| Chronic liver failure \| \| Cirrhosis \| \| Cirrhosis of liver \| \| Hepatic Failure \| \| Liver disease end stage \| \| Liver failure \| \| \| --- \| --- \| --- \| --- \| --- \| --- \| --- \| --- \| --- \| --- \| --- \| --- \| --- \| --- \| --- \| --- \| --- \| --- \| --- \| --- \| \|  \| | \| **ICD9/TERMWORKS** \| \| --- \| \|  \| \| **ICD9/TERMWORKS** \| \| **ICD9/TERMWORKS** \| \| **ICD9/TERMWORKS** \| \| **ICD9/TERMWORKS** \| \| **ICD9/TERMWORKS** \| \|  \| \|  \| \| **ICD9/TERMWORKS** \| \|  \| \| **CPCSSN Free Text** \| \| **CPCSSN Free Text** \| \| **CPCSSN Free Text** \| \| **CPCSSN Free Text** \| \| **CPCSSN Free Text** \| \| **CPCSSN Free Text** \| \| **CPCSSN Free Text** \| \| **CPCSSN Free Text** \| \|  \| |

Termworks search terms: cirrhosis, liver, hepatic

## Mental health challenges

Panelists’ Descriptions:

| -        Profound mental illness that impacts day to day functioning |
| --- |
| -        Trauma/history of trauma |
| -        Profound mental Illness (i.e. schizophrenia) that impacts day to day functioning |
| -        Depression |
| -        Anxiety |
| -        Mood disorders |
| -        Mental illness/mental health disorders |
| -        Psychosis |

| **Codes** | **Source(s)** |
| --- | --- |
| \| \| 290 SENILE AND PRESENILE ORGANIC PSYCHOTIC CONDITIONS \| \| --- \| \| 290.0 SENILE DEMENTIA, SIMPLE TYPE \| \| 290.1 PRESENILE DEMENTIA \| \| 290.10 Presenile dementia, uncomplicated \| \| 290.11 Presenile dementia with delirium \| \| 290.12 Presenile dementia with delusional features \| \| 290.13 Presenile dementia with depressive features \| \| 290.2 SENILE DEMENTIA, DEPRESSED OR PARANOID TYPE \| \| 290.20 Senile dementia with delusional features \| \| 290.21 Senile dementia with depressive features \| \| 290.3 SENILE DEMENTIA WITH ACUTE CONFUSIONAL STATE \| \| 290.4 ARTERIOSCLEROTIC DEMENTIA / VASCULAR DEMENTIA (ICD9-CM) \| \| 290.40 Vascular dementia, uncomplicated \| \| 290.41 Vascular dementia, with delirium \| \| 290.42 Vascular dementia, with delusions \| \| 290.43 Vascular dementia, with depressed mood \| \| 290.8 OTHER \| \| 290.9 UNSPECIFIED \| \|  \| \| 291 ALCOHOLIC PSYCHOSES (Alcoholic-induced mental disorders ICD9-CM) \| \| 291.0 DELIRIUM TREMENS (Alcohol withdrawal delirium) \| \| 291.1 KORSAKOV’S PSYCHOSIS, ALCOHOLIC (Alcohol-induced persisting amnestic disorder) \| \| 291.2 OTHER ALCOHOLIC DEMENTIA (Alcohol-induced persisting dementia) \| \| 291.3 OTHER ALCOHOLIC HALLUCINOSIS (Alcohol-induced psychotic disorder with hallucinations) \| \| 291.4 PATHOLOGICAL DRUNKENNESS (Idiosyncratic alcohol intoxication) \| \| 291.5 ALCOHOLIC JEALOUSY (Alcohol-induced psychotic disorder with delusions) \| \| 291.8 OTHER \| \| 291.81 Alcohol withdrawal \| \| 291.82 Alcohol induced sleep disorders \| \| 291.89 Other alcohol-induced mental disorders \| \| 291.9 UNSPECIFIED \| \|  \| \| 292 DRUG PSYCHOSES (Drug-induced mental disorders) \| \| 292.0 DRUG WITHDRAWAL SYNDROME \| \| 292.1 PARANOID AND/OR HALLUCINATORY STATES INDUCED BY DRUGS \| \| 292.11 Drug-induced psychotic disorder with delusions \| \| 292.12 Drug-induced psychotic disorder with hallucinations \| \| 292.2 PATHOLOGICAL DRUG INTOXICATION \| \| 292.8 OTHER \| \| 292.81 Drug-induced delirium \| \| 292.82 Drug-induced persisting dementia \| \| 292.83 Drug-induced persisting amnestic disorder \| \| 292.84 Drug-induced mood disorder \| \| 292.85 Drug induced sleep disorders \| \| 292.89 Other specified drug-induced mental disorders \| \| 292.9 UNSPECIFIED \| \|  \| \| 293 TRANSIENT ORGANIC PSYCHOTIC CONDITIONS (Transient mental disorders due to conditions classified elsewhere) \| \| 293.0 ACUTE CONFUSIONAL STATE (293.0 Delirium due to conditions classified elsewhere) \| \| 293.1 SUBACUTE CONFUSIONAL STATE (293.1 Subacute delirium) \| \| 293.8 OTHER \| \| 293.81 Psychotic disorder with delusions in conditions classified elsewhere \| \| 293.82 Psychotic disorder with hallucinations in conditions classified elsewhere \| \| 293.83 Mood disorder in conditions classified elsewhere \| \| 293.84 Anxiety disorder in conditions classified elsewhere \| \| 293.89 Other specified transient mental disorders due to conditions classified elsewhere, other \| \| 293.9 UNSPECIFIED \| \|  \| \| 294 OTHER ORGANIC PSYCHOTIC CONDITIONS (CHRONIC) (294 Persistent mental disorders due to conditions classified elsewhere) \| \| 294.0 KORSAKOV’S PSYCHOSIS OR SYNDROME (NONALCOHOLIC) (294.0 Amnestic disorder in conditions classified elsewhere) \| \| 294.1 DEMENTIA IN CONDITIONS CLASSIFIED ELSEWHERE \| \| 294.10 Dementia in conditions classified elsewhere without behavioral disturbance \| \| 294.11 Dementia in conditions classified elsewhere with behavioral disturbance \| \| 294.2 Dementia, unspecified \| \| 294.20 Dementia, unspecified, without behavioral disturbance \| \| 294.21 Dementia, unspecified, with behavioral disturbance \| \| 294.8 OTHER \| \| 294.9 UNSPECIFIED \| \|  \| \| 295 SCHIZOPHRENIC PSYCHOSES / disorders (ICD9-CM)* \| \| 295.0 SIMPLE TYPE* \| \| 295.1 HEBEPHRENIC TYPE ( 295.1 Disorganized type schizophrenia)* \| \| 295.2 CATATONIC TYPE* \| \| 295.3 PARANOID TYPE* \| \| 295.4 ACUTE SCHIZOPHRENIC EPISODE (295.4 Schizophreniform disorder)* \| \| 295.5 LATENT SCHIZOPHRENIA* \| \| 295.6 RESIDUAL SCHIZOPHRENIA (295.6 Schizophrenic disorder, residual type)* \| \| 295.7 SCHIZOAFFECTIVE TYPE* \| \| 295.8 OTHER* \| \| 295.9 UNSPECIFIED* \| \|  \| \| 296 AFFECTIVE PSYCHOSES (296 Episodic mood disorders)* \| \| 296.0 MANIC-DEPRESSIVE PSYCHOSIS, MANIC TYPE (296.0 Bipolar i disorder, single manic episode)* \| \| 296.1 MANIC-DEPRESSIVE PSYCHOSIS, DEPRESSED TYPE (296.1 Manic disorder recurrent episode)* \| \| 296.2 MANIC-DEPRESSIVE PSYCHOSIS, CIRCULAR TYPE BUT CURRENTLY MANIC (296.2 Major depressive disorder single episode)* \| \| 296.3 MANIC-DEPRESSIVE PSYCHOSIS, CIRCULAR TYPE BUT CURRENTLY DEPRESSED (296.3 Major depressive disorder recurrent episode)* \| \| 296.4 MANIC-DEPRESSIVE PSYCHOSIS, CIRCULAR TYPE, MIXED (296.4 Bipolar i disorder, most recent episode (or current) manic)* \| \| 296.5 MANIC-DEPRESSIVE PSYCHOSIS, CIRCULAR TYPE, CURRENT CONDITION NOT SPECIFIED (296.5 Bipolar i disorder, most recent episode (or current) depressed)* \| \| 296.6 MANIC-DEPRESSIVE PSYCHOSIS, OTHER AND UNSPECIFIED (296.6 Bipolar i disorder, most recent episode (or current) mixed)* \| \| 296.7 Bipolar I disorder, most recent episode (or current) unspecified* \| \| 296.8 OTHER (296.8 Other and unspecified bipolar disorders)* \| \| 296.9 UNSPECIFIED (296.9 Other and unspecified episodic mood disorder)* \| \|  \| \| 297 PARANOID STATES (297 Delusional disorders) \| \| 297.0 PARANOID STATE, SIMPLE (297.0 Paranoid state, simple) \| \| 297.1 PARANOIA (297.1 Delusional disorder) \| \| 297.2 PARAPHRENIA (297.2 Paraphrenia) \| \| 297.3 INDUCED PSYCHOSIS (297.3 Shared psychotic disorder) \| \| 297.8 OTHER (297.8 Other specified paranoid states) \| \| 297.9 UNSPECIFIED (297.9 Unspecified paranoid state) \| \|  \| \| 298 OTHER NONORGANIC PSYCHOSES \| \| 298.0 DEPRESSIVE TYPE \| \| 298.1 EXCITATIVE TYPE \| \| 298.2 REACTIVE CONFUSION \| \| 298.3 ACUTE PARANOID REACTION \| \| 298.4 PSYCHOGENIC PARANOID PSYCHOSIS \| \| 298.8 OTHER AND UNSPECIFIED REACTIVE PSYCHOSIS \| \| 298.9 UNSPECIFIED PSYCHOSIS \| \|  \| \| 300 NEUROTIC DISORDERS (300 Anxiety, dissociative and somatoform disorders) \| \| 300.0 ANXIETY STATES \| \| 300.00 Anxiety state, unspecified \| \| 300.01 Panic disorder without agoraphobia \| \| 300.02 Generalized anxiety disorder \| \| 300.09 Other anxiety states \| \| 300.1 HYSTERIA (300.1 Dissociative, conversion and factitious disorders) \| \| 300.10 Hysteria, unspecified \| \| 300.11 BLINDNESS, HYSTERICAL ( (300.11 Conversion disorder) \| \| 300.12 Dissociative amnesia \| \| 300.13 Dissociative fugue \| \| 300.14 Dissociative identity disorder \| \| 300.15 Dissociative disorder or reaction, unspecified \| \| 300.16 Factitious disorder with predominantly psychological signs and symptoms \| \| 300.19 Other and unspecified factitious illness \| \| 300.2 PHOBIC STATE (300.2 Phobic disorders) \| \| 300.20 Phobia, unspecified \| \| 300.21 Agoraphobia with panic disorder \| \| 300.22 Agoraphobia without mention of panic attacks \| \| 300.23 Social phobia \| \| 300.29 Other isolated or specific phobias \| \| 300.3 OBSESSIVE-COMPULSIVE DISORDERS \| \| 300.4 NEUROTIC DEPRESSION (300.4 Dysthymic disorder) \| \| 300.5 NEURASTHENIA \| \| 300.6 DEPERSONALIZATION SYNDROME \| \| 300.7 HYPOCHONDRIASIS \| \| 300.8 OTHER NEUROTIC DISORDERS (300.8 Somatoform disorders) \| \| 300.81 Somatization disorder \| \| 300.82 Undifferentiated somatoform disorder \| \| 300.89 Other somatoform disorders \| \| 300.9 UNSPECIFIED \| \|  \| \| 301 PERSONALITY DISORDERS \| \| 301.0 PARANOID PERSONALITY DISORDER \| \| 301.1 AFFECTIVE PERSONALITY DISORDER \| \| 301.10 Affective personality disorder, unspecified \| \| 301.11 Chronic hypomanic personality disorder \| \| 301.12 Chronic depressive personality disorder \| \| 301.13 Cyclothymic disorder \| \| 301.2 SCHIZOID PERSONALITY DISORDER \| \| 301.20 Schizoid personality disorder, unspecified \| \| 301.21 Introverted personality \| \| 301.22 Schizotypal personality disorder \| \| 301.3 EXPLOSIVE PERSONALITY DISORDER \| \| 301.4 ANANKASTIC PERSONALITY DISORDER (301.4 Obsessive-compulsive personality disorder) \| \| 301.5 HYSTERICAL PERSONALITY DISORDER (301.5 Histrionic personality disorder) \| \| 301.50 Histrionic personality disorder, unspecified \| \| 301.51 Chronic factitious illness with physical symptoms \| \| 301.59 Other histrionic personality disorder \| \| 301.6 ASTHENIC PERSONALITY DISORDER (301.6 Dependent personality disorder) \| \| 301.7 MANIFESTATION PERSONALITY DISORDER WITH PREDOMINANTLY SOCIOPATHIC OR ASOCIAL (301.7 Antisocial personality disorder) \| \| 301.8 OTHER PERSONALITY DISORDERS \| \| 301.81 Narcissistic personality disorder \| \| 301.82 Avoidant personality disorder \| \| 301.83 Borderline personality disorder \| \| 301.84 Passive-aggressive personality \| \| 301.89 Other personality disorders \| \| 301.9 UNSPECIFIED \| \|  \| \| 309 ADJUSTMENT REACTION \| \| 309.1 PROLONGED DEPRESSIVE REACTION \| \| 309.2 WITH PREDOMINANT DISTURBANCE OF OTHER EMOTIONS \| \| 309.24 Adjustment disorder with anxiety \| \| 309.28 Adjustment disorder with mixed anxiety and depressed mood \| \| 309.3 WITH PREDOMINANT DISTURBANCE OF CONDUCT \| \| 309.4 WITH MIXED DISTURBANCE OF EMOTIONS AND CONDUCT \| \| 309.8 OTHER \| \| 309.81 Posttraumatic stress disorder \| \| 309.82 Adjustment reaction with physical symptoms \| \| 309.83 Adjustment reaction with withdrawal \| \| 309.89 Other specified adjustment reactions \| \| 309.9 UNSPECIFIED \| \|  \| \| 311 DEPRESSIVE DISORDER, NOT ELSEWHERE CLASSIFIED \| \|  \| \| 312 DISTURBANCE OF CONDUCT NOT ELSEWHERE CLASSIFIED* \| \| 312.0 UNSOCIALIZED DISTURBANCE OF CONDUCT (312.0 Undersocialized conduct disorder aggressive type)* \| \| 312.1 SOCIALIZED DISTURBANCE OF CONDUCT (312.1 Undersocialized conduct disorder unaggressive type)* \| \| 312.2 COMPULSIVE CONDUCT DISORDER (312.2 Socialized conduct disorder)* \| \| 312.3 MIXED DISTURBANCE OF CONDUCT AND EMOTIONS (312.3 Disorders of impulse control not elsewhere classified) \| \| 312.30 Impulse control disorder, unspecified \| \| 312.31 Pathological gambling \| \| 312.32 Kleptomania \| \| 312.33 Pyromania \| \| 312.34 Intermittent explosive disorder \| \| 312.35 Isolated explosive disorder \| \| 312.39 Other disorders of impulse control \| \| 312.4 Mixed disturbance of conduct and emotions \| \| 312.8 OTHER \| \| 312.9 UNSPECIFIED \| \|  \| \| 780.97 Altered mental status \| \|  \| \| V11 PERSONAL HISTORY OF MENTAL DISORDER \| \| V11.0 Personal history of schizophrenia \| \| V11.1 Personal history of affective disorders \| \| V11.2 Personal history of neurosis \| \| V11.3 Personal history of alcoholism \| \| V11.4 Personal history of combat and operational stress reaction \| \| V11.8 Personal history of other mental disorders \| \| V11.9 Personal history of unspecified mental disorder \| \|  \| \| V15.4 PSYCHOLOGICAL TRAUMA (Under OTHER PERSONAL HISTORY PRESENTING HAZARDS TO HEALTH) \| \| V15.41 History of physical abuse \| \| V15.42 History of emotional abuse \| \| V15.49 Other psychological trauma \| \|  \| \| V62.4 SOCIAL MALADJUSTMENT \| \| V62.8 OTHER PSYCHOLOGICAL OR PHYSICAL STRAIN, NOT ELSEWHERE CLASSIFIED \| \| V62.81 Interpersonal problems, not elsewhere classified \| \| V62.82 Bereavement, uncomplicated \| \| V62.83 Counseling for perpetrator of physical/sexual abuse \| \| V62.84 Suicidal ideation \| \| V62.85 Homicidal ideation \| \| V62.89 Other psychological or physical stress, not elsewhere classified \| \|  \| \| V65.40 Counseling NOS \| \| V66.3 Convalescence following psychotherapy and other treatment for mental disorder \| \| V67.3 Follow-up examination, following psychotherapy and other treatment for mental disorder \| \| V70.1 GENERAL PSYCHIATRIC EXAMINATION, REQUESTED BY THE AUTHORITY \| \| V70.2 GENERAL PSYCHIATRIC EXAMINATION, OTHER AND UNSPECIFIED \| \| V71.0 Observation for suspected mental condition \| \| V71.09 Observation for other suspected mental condition \| \| V79 SPECIAL SCREENING FOR MENTAL DISORDERS AND DEVELOPMENTAL HANDICAPS \| \| V79.0 DEPRESSION \| \|  \| \| 50B ANXIETY/DEPRESSION \| \|  \| \| E950 Suicide and self-inflicted poisoning by solid or liquid substances* \| \| E951 Suicide and self-inflicted poisoning by gases in domestic use* \| \| E952 Suicide and self-inflicted poisoning by other gases and vapors* \| \| E953 Suicide and self-inflicted injury by hanging strangulation and suffocation* \| \| E954 Suicide and self-inflicted injury by submersion [drowning]* \| \| E955 Suicide and self-inflicted injury by firearms air guns and explosives* \| \| E956 Suicide and self-inflicted injury by cutting and piercing instrument* \| \| E957 Suicide and self-inflicted injuries by jumping from high place* \| \| E958 Suicide and self-inflicted injury by other and unspecified means* \| \| E959 Late effects of self-inflicted injury* \| \|  \| \| **Lithium** \| \| [Moles/volume] in Serum or Plasma: 0.6 to 1.2 mEq/L; 0.6 to 1.2 mmol/L \| \| Presence \| \|  \| \| Adjustment disorder \| \| Affective psychosis \| \| Agitation \| \| Agoraphobia \| \| Antidepressant \| \| Anxiety \| \| Behavioural concern \| \| Behavioral concern \| \| Behavioural disorder \| \| Behavioural disturbance \| \| Bipolar \| \| Borderline personality \| \| Chronic depression \| \| Chronic psychosis \| \| Delusion \| \| Delusional disorder \| \| Depression \| \| Depressed \| \| Emotional crisis \| \| Emotional distress \| \| Emotional health \| \| Emotional instability \| \| Hallucination \| \| hx of trauma \| \| Hypomania \| \| Lithium \| \| Mania \| \| MDD \| \| Major Depressive Disorder \| \| Mental and behavioural problems \| \| Mental health \| \| Mental health care \| \| mental illness \| \| MH issue \| \| MH Team \| \| Mood disorder \| \| Panic attack \| \| Panic disorder \| \| Paranoia \| \| Paranoid \| \| Paranoid psychosis \| \| paranoid state \| \| Personality disorder \| \| Phantom limb \| \| Phobia \| \| Post traumatic stress disorder \| \| Pseudoseizure \| \| Psychiatric \| \| Psychosis \| \| Psychotic disorder \| \| PTSD \| \| Schizophrenia \| \| Schizo \| \| Schizo affective disorder \| \| Schizeoaffective disorder \| \| Seasonal affective disorder \| \| Social anxiety \| \| Suicidal \| \| Suicide \| \| Trauma history \| \|  \| \| \| --- \| --- \| --- \| --- \| --- \| --- \| --- \| --- \| --- \| --- \| --- \| --- \| --- \| --- \| --- \| --- \| --- \| --- \| --- \| --- \| --- \| --- \| --- \| --- \| --- \| --- \| --- \| --- \| --- \| --- \| --- \| --- \| --- \| --- \| --- \| --- \| --- \| --- \| --- \| --- \| --- \| --- \| --- \| --- \| --- \| --- \| --- \| --- \| --- \| --- \| --- \| --- \| --- \| --- \| --- \| --- \| --- \| --- \| --- \| --- \| --- \| --- \| --- \| --- \| --- \| --- \| --- \| --- \| --- \| --- \| --- \| --- \| --- \| --- \| --- \| --- \| --- \| --- \| --- \| --- \| --- \| --- \| --- \| --- \| --- \| --- \| --- \| --- \| --- \| --- \| --- \| --- \| --- \| --- \| --- \| --- \| --- \| --- \| --- \| --- \| --- \| --- \| --- \| --- \| --- \| --- \| --- \| --- \| --- \| --- \| --- \| --- \| --- \| --- \| --- \| --- \| --- \| --- \| --- \| --- \| --- \| --- \| --- \| --- \| --- \| --- \| --- \| --- \| --- \| --- \| --- \| --- \| --- \| --- \| --- \| --- \| --- \| --- \| --- \| --- \| --- \| --- \| --- \| --- \| --- \| --- \| --- \| --- \| --- \| --- \| --- \| --- \| --- \| --- \| --- \| --- \| --- \| --- \| --- \| --- \| --- \| --- \| --- \| --- \| --- \| --- \| --- \| --- \| --- \| --- \| --- \| --- \| --- \| --- \| --- \| --- \| --- \| --- \| --- \| --- \| --- \| --- \| --- \| --- \| --- \| --- \| --- \| --- \| --- \| --- \| --- \| --- \| --- \| --- \| --- \| --- \| --- \| --- \| --- \| --- \| --- \| --- \| --- \| --- \| --- \| --- \| --- \| --- \| --- \| --- \| --- \| --- \| --- \| --- \| --- \| --- \| --- \| --- \| --- \| --- \| --- \| --- \| --- \| --- \| --- \| --- \| --- \| --- \| --- \| --- \| --- \| --- \| --- \| --- \| --- \| --- \| --- \| --- \| --- \| --- \| --- \| --- \| --- \| --- \| --- \| --- \| --- \| --- \| --- \| --- \| --- \| --- \| --- \| --- \| --- \| --- \| --- \| --- \| --- \| --- \| --- \| --- \| --- \| --- \| --- \| --- \| --- \| --- \| --- \| --- \| --- \| --- \| --- \| --- \| --- \| --- \| --- \| --- \| --- \| --- \| --- \| --- \| --- \| --- \| --- \| --- \| --- \| --- \| --- \| --- \| --- \| --- \| --- \| --- \| --- \| --- \| --- \| --- \| --- \| --- \| --- \| --- \| --- \| --- \| --- \| --- \| --- \| --- \| --- \| --- \| --- \| --- \| --- \| --- \| --- \| | \| **ICD9** \| \| --- \| \| **ICD9** \| \| **ICD9** \| \| **ICD9-CM** \| \| **ICD9-CM** \| \| **ICD9-CM** \| \| **ICD9-CM/TERMWORKS** \| \| **ICD9/TERMWORKS** \| \| **ICD9-CM** \| \| **ICD9-CM/TERMWORKS** \| \| **ICD9** \| \| **ICD9** \| \| **ICD9-CM** \| \| **ICD9-CM** \| \| **ICD9-CM** \| \| **ICD9-CM/TERMWORKS** \| \| **ICD9/TERMWORKS** \| \| **ICD9** \| \|  \| \| **ICD9/TERMWORKS** \| \| **ICD9** \| \| **ICD9** \| \| **ICD9** \| \| **ICD9** \| \| **ICD9** \| \| **ICD9** \| \| **ICD9/TERMWORKS** \| \| **ICD9-CM** \| \| **ICD9-CM** \| \| **ICD9-CM/TERMWORKS** \| \| **ICD9/TERMWORKS** \| \|  \| \| **ICD9/TERMWORKS** \| \| **ICD9** \| \| **ICD9** \| \| **ICD9-CM** \| \| **ICD9-CM** \| \| **ICD9** \| \| **ICD9/TERMWORKS** \| \| **ICD9-CM** \| \| **ICD9-CM** \| \| **ICD9-CM** \| \| **ICD9-CM** \| \| **ICD9-CM/TERMWORKS** \| \| **ICD9-CM/TERMWORKS** \| \| **ICD9/TERMWORKS** \| \|  \| \| **ICD9/TERMWORKS** \| \| **ICD9** \| \| **ICD9** \| \| **ICD9/TERMWORKS** \| \| **ICD9-CM** \| \| **ICD9-CM** \| \| **ICD9-CM/TERMWORKS** \| \| **ICD9-CM/TERMWORKS** \| \| **ICD9-CM/TERMWORKS** \| \| **ICD9/TERMWORKS** \| \|  \| \| **ICD9/TERMWORKS** \| \| **ICD9** \| \| **ICD9** \| \| **ICD9-CM** \| \| **ICD9-CM** \| \| **ICD9-CM** \| \| **ICD9-CM** \| \| **ICD9-CM** \| \| **ICD9/TERMWORKS** \| \| **ICD9/TERMWORKS** \| \|  \| \| **ICD9/TERMWORKS** \| \| **ICD9** \| \| **ICD9/TERMWORKS** \| \| **ICD9/TERMWORKS** \| \| **ICD9/TERMWORKS** \| \| **ICD9** \| \| **ICD9/TERMWORKS** \| \| **ICD9/TERMWORKS** \| \| **ICD9/TERMWORKS** \| \| **ICD9/TERMWORKS** \| \| **ICD9/TERMWORKS** \| \|  \| \| **ICD9/TERMWORKS** \| \| **ICD9** \| \| **ICD9** \| \| **ICD9/TERMWORKS** \| \| **ICD9/TERMWORKS** \| \| **ICD9** \| \| **ICD9/TERMWORKS** \| \| **ICD9** \| \| **ICD9-CM** \| \| **ICD9/TERMWORKS** \| \| **ICD9/TERMWORKS** \| \|  \| \| **ICD9** \| \| **ICD9** \| \| **ICD9** \| \| **ICD9** \| \| **ICD9** \| \| **ICD9** \| \| **ICD9** \| \|  \| \| **ICD9/TERMWORKS** \| \| **ICD9/TERMWORKS** \| \| **ICD9/TERMWORKS** \| \| **ICD9** \| \| **ICD9** \| \| **ICD9/TERMWORKS** \| \| **ICD9/TERMWORKS** \| \| **ICD9/TERMWORKS** \| \|  \| \| **ICD9/TERMWORKS** \| \| **ICD9/TERMWORKS** \| \| **ICD9-CM** \| \| **ICD9-CM** \| \| **ICD9-CM/TERMWORKS** \| \| **ICD9-CM/TERMWORKS** \| \| **ICD9** \| \| **ICD9-CM** \| \| **ICD9** \| \| **ICD9-CM** \| \| **ICD9-CM** \| \| **ICD9-CM** \| \| **ICD9-CM** \| \| **ICD9-CM** \| \| **ICD9-CM** \| \| **ICD9** \| \| **ICD9-CM** \| \| **ICD9-CM** \| \| **ICD9-CM** \| \| **ICD9-CM** \| \| **ICD9-CM** \| \| **ICD9** \| \| **ICD9** \| \| **ICD9** \| \| **ICD9** \| \| **ICD9** \| \| **ICD9** \| \| **ICD9-CM** \| \| **ICD9-CM** \| \| **ICD9-CM** \| \| **ICD9/TERMWORKS** \| \|  \| \| **ICD9** \| \| **ICD9** \| \| **ICD9** \| \| **ICD9-CM** \| \| **ICD9-CM** \| \| **ICD9-CM/TERMWORKS** \| \| **ICD9-CM** \| \| **ICD9** \| \| **ICD9-CM** \| \| **ICD9-CM** \| \| **ICD9-CM** \| \| **ICD9** \| \| **ICD9** \| \| **ICD9** \| \| **ICD9-CM** \| \| **ICD9-CM** \| \| **ICD9-CM** \| \| **ICD9** \| \| **ICD9** \| \| **ICD9** \| \| **ICD9-CM** \| \| **ICD9-CM** \| \| **ICD9-CM** \| \| **ICD9-CM** \| \| **ICD9-CM** \| \| **ICD9** \| \|  \| \| **ICD9/TERMWORKS** \| \| **ICD9/TERMWORKS** \| \| **ICD9** \| \| **ICD9-CM/TERMWORKS** \| \| **ICD9-CM/TERMWORKS** \| \| **ICD9** \| \| **ICD9** \| \| **ICD9** \| \| **ICD9-CM** \| \| **ICD9-CM** \| \| **ICD9-CM** \| \| **ICD9-CM** \| \| **ICD9** \| \|  \| \| **ICD9/TERMWORKS** \| \|  \| \| **ICD9** \| \| **ICD9** \| \| **ICD9** \| \| **ICD9** \| \| **ICD9** \| \| **ICD9-CM** \| \| **ICD9-CM** \| \| **ICD9-CM** \| \| **ICD9-CM** \| \| **ICD9-CM** \| \| **ICD9-CM** \| \| **ICD9-CM** \| \| **ICD9-CM** \| \| **ICD9** \| \| **ICD9** \| \|  \| \| **ICD9-CM/TERMWORKS** \| \|  \| \| **ICD9/TERMWORKS** \| \| **ICD9-CM/TERMWORKS** \| \| **ICD9-CM** \| \| **ICD9-CM** \| \| **ICD9-CM** \| \| **ICD9-CM** \| \| **ICD9-CM/TERMWORKS** \| \| **ICD9-CM/TERMWORKS** \| \|  \| \| **TERMWORKS** \| \| **ICD9-CM** \| \| **ICD9-CM** \| \| **ICD9-CM** \| \|  \| \| **ICD9** \| \| **ICD9** \| \| **ICD9-CM** \| \| **ICD9-CM** \| \| **ICD9-CM** \| \| **ICD9-CM** \| \| **ICD9-CM** \| \| **ICD9-CM** \| \|  \| \| **ICD9-CM** \| \| **ICD9-CM/TERMWORKS** \| \| **ICD9-CM/TERMWORKS** \| \| **ICD9** \| \| **ICD9** \| \| **ICD9-CM/TERMWORKS** \| \| **ICD9-CM/TERMWORKS** \| \| **ICD9/TERMWORKS** \| \| **ICD9/TERMWORKS** \| \|  \| \| **ICD9** \| \|  \| \| **ICD9-CM** \| \| **ICD9-CM** \| \| **ICD9-CM** \| \| **ICD9-CM** \| \| **ICD9-CM** \| \| **ICD9-CM** \| \| **ICD9-CM** \| \| **ICD9-CM** \| \| **ICD9-CM** \| \| **ICD9-CM** \| \|  \| \|  \| \| **LOINC 14334-7** \| \| **LOINC 3720-0** \| \|  \| \| **CPCSSN Free Text** \| \| **CPCSSN Free Text** \| \| **CPCSSN Free Text** \| \| **CPCSSN Free Text** \| \| **CPCSSN Free Text** \| \| **CPCSSN Free Text** \| \| **CPCSSN Free Text** \| \| **CPCSSN Free Text** \| \| **CPCSSN Free Text** \| \| **CPCSSN Free Text** \| \| **CPCSSN Free Text** \| \| **CPCSSN Free Text** \| \| **CPCSSN Free Text** \| \| **CPCSSN Free Text** \| \| **CPCSSN Free Text** \| \| **CPCSSN Free Text** \| \| **CPCSSN Free Text** \| \| **CPCSSN Free Text** \| \| **CPCSSN Free Text** \| \| **CPCSSN Free Text** \| \| **CPCSSN Free Text** \| \| **CPCSSN Free Text** \| \| **CPCSSN Free Text** \| \| **CPCSSN Free Text** \| \| **CPCSSN Free Text** \| \| **CPCSSN Free Text** \| \| **CPCSSN Free Text** \| \| **CPCSSN Free Text** \| \| **CPCSSN Free Text** \| \| **CPCSSN Free Text** \| \| **CPCSSN Free Text** \| \| **CPCSSN Free Text** \| \| **CPCSSN Free Text** \| \| **CPCSSN Free Text** \| \| **CPCSSN Free Text** \| \| **CPCSSN Free Text** \| \| **CPCSSN Free Text** \| \| **CPCSSN Free Text** \| \| **CPCSSN Free Text** \| \| **CPCSSN Free Text** \| \| **CPCSSN Free Text** \| \| **CPCSSN Free Text** \| \| **CPCSSN Free Text** \| \| **CPCSSN Free Text** \| \| **CPCSSN Free Text** \| \| **CPCSSN Free Text** \| \| **CPCSSN Free Text** \| \| **CPCSSN Free Text** \| \| **CPCSSN Free Text** \| \| **CPCSSN Free Text** \| \| **CPCSSN Free Text** \| \| **CPCSSN Free Text** \| \| **CPCSSN Free Text** \| \| **CPCSSN Free Text** \| \| **CPCSSN Free Text** \| \| **CPCSSN Free Text** \| \| **CPCSSN Free Text** \| \| **CPCSSN Free Text** \| \| **CPCSSN Free Text** \| \| **CPCSSN Free Text** \| |

Termworks search terms: mental, trauma, schizophrenia, depression, anxiety, mood, psychosis, depressive

## Medication noncompliance

| **Codes** | **Source(s)** |
| --- | --- |
| \| \| V15.81 Personal history of noncompliance with medical treatment, presenting hazards to health \| \| --- \| \|  \| \| Non-compliant \| \| Noncompliant \| \| Nonadherence \| \| Noncompliance \| \| Non-compliance \| \| \| --- \| --- \| --- \| --- \| --- \| --- \| --- \| --- \| \|  \| | \| **ICD9-CM/TERMWORKS** \| \| --- \| \|  \| \| **CPCSSN Free Text** \| \| **CPCSSN Free Text** \| \| **CPCSSN Free Text** \| \| **CPCSSN Free Text** \| \| **CPCSSN Free Text** \| |

Termworks search terms: medication, medications, noncompliance

## Poverty/financial difficulties

Panelists’ Descriptions:

| -        Poverty (inability to gain access to resources and services required to prevent health decline) |
| --- |
| -        Financial difficulties (leaving people unable to afford basic necessities) |
| -        Loss of income- inability to afford medication, health equipment, housing |
| -        Low income |
| -        Financial insecurity |
| -        Low SES; limited/no access to adequate nutrition, transportation to appointments or the means to pay out of pocket for prescriptions |
|  |

| **Codes** | **Source(s)** |
| --- | --- |
| \| \| V60 HOUSING, HOUSEHOLD AND ECONOMIC CIRCUMSTANCES \| \| --- \| \| V60.2 INADEQUATE MATERIAL RESOURCES \| \|  \| \| Finance \| \| Financial issue \| \| Financial problem \| \| Income \| \| Poverty \| \| Rent payment \| \| Socioeconomic difficulty \| \| \| --- \| --- \| --- \| --- \| --- \| --- \| --- \| --- \| --- \| --- \| --- \| \|  \| | \| **ICD9/TERMWORKS** \| \| --- \| \| **ICD9/TERMWORKS** \| \|  \| \| **CPCSSN Free Text** \| \| **CPCSSN Free Text** \| \| **CPCSSN Free Text** \| \| **CPCSSN Free Text** \| \| **CPCSSN Free Text** \| \| **CPCSSN Free Text** \| \| **CPCSSN Free Text** \| \|  \| |

Termworks search terms: poverty, financial, economic, income, socioeconomic

## Race/ethnic disparity

Panelists’ Descriptions:

| -        Race/ethnicity disparity (i.e. differences in health/health outcomes between racial/ethnic groups) |
| --- |

| **Codes** | **Source(s)** |
| --- | --- |
| \| \| Residential school survivor \| \| --- \| \| First Nations \| \| \| --- \| --- \| --- \| \|  \| | \| **CPCSSN Free Text** \| \| --- \| \| **CPCSSN Free Text** \| |

Termworks search terms: race, ethnic, disparity

## Sedentary/low activity levels

Panelists’ Descriptions:

| -        Sedentary/low activity levels |
| --- |
| -        Views on exercise and activity  -       Exhaustion and fatigue |

| **Codes** | **Source(s)** |
| --- | --- |
| \| \| 278 OBESITY AND OTHER HYPERALIMENTATION \| \| --- \| \| 278.0 OBESITY \| \| 278.00 Obesity, unspecified \| \| 278.01 Morbid obesity \| \| 278.02 Overweight \| \| 278.03 Obesity hypoventilation syndrome \| \|  \| \| 783.1 ABNORMAL WEIGHT GAIN \| \|  \| \| V65.41 Exercise counseling \| \| V69.0 Lack of physical exercise \| \|  \| \| Activity decreased \| \| Exercise intolerance \| \| Exercise tolerance decreased \| \| Inactivity \| \| Inadequate exercise \| \| Lack of physical activity \| \| Morbid obesity \| \| Obese \| \| Obesity \| \| Overweight \| \| Poor fitness \| \| Physical inactivity \| \| Refusing to mobilize \| \| Sedentary \| \| Always tired \| \| Drowsiness \| \| Exhausted \| \| Exhaustion \| \| Fatigue \| \| Lack of energy \| \| Lethargy \| \| Lethargic \| \| Low energy \| \| Malaise \| \| No energy \| \| Poor energy \| \| Tired \| \| Tiredness \| \|  \| \| \| --- \| --- \| --- \| --- \| --- \| --- \| --- \| --- \| --- \| --- \| --- \| --- \| --- \| --- \| --- \| --- \| --- \| --- \| --- \| --- \| --- \| --- \| --- \| --- \| --- \| --- \| --- \| --- \| --- \| --- \| --- \| --- \| --- \| --- \| --- \| --- \| --- \| --- \| --- \| --- \| --- \| --- \| | \| **ICD9** \| \| --- \| \| **ICD9** \| \| **ICD9-CM** \| \| **ICD9-CM** \| \| **ICD9-CM** \| \| **ICD9-CM** \| \|  \| \| **ICD9** \| \|  \| \| **ICD9-CM/TERMWORKS** \| \| **ICD9-CM/TERMWORKS** \| \|  \| \| **CPCSSN Free Text** \| \| **CPCSSN Free Text** \| \| **CPCSSN Free Text** \| \| **CPCSSN Free Text** \| \| **CPCSSN Free Text** \| \| **CPCSSN Free Text** \| \| **CPCSSN Free Text** \| \| **CPCSSN Free Text** \| \| **CPCSSN Free Text** \| \| **CPCSSN Free Text** \| \| **CPCSSN Free Text** \| \| **CPCSSN Free Text** \| \| **CPCSSN Free Text** \| \| **CPCSSN Free Text** \| \| **CPCSSN Free Text** \| \| **CPCSSN Free Text** \| \| **CPCSSN Free Text** \| \| **CPCSSN Free Text** \| \| **CPCSSN Free Text** \| \| **CPCSSN Free Text** \| \| **CPCSSN Free Text** \| \| **CPCSSN Free Text** \| \| **CPCSSN Free Text** \| \| **CPCSSN Free Text** \| \| **CPCSSN Free Text** \| \| **CPCSSN Free Text** \| \| **CPCSSN Free Text** \| \| **CPCSSN Free Text** \| \|  \| |

Termworks search terms: sedentary, low $+ activity, exercise

## Substance use/misuse

Panelists’ Descriptions:

| -        Substance misuse/abuse - alcohol in particular in the elderly but other recreational drug abuse as well. |
| --- |
| -        Alcohol/Smoking |
| -        Addiction |
| -        Drug and substance use disorders |

| **Codes** | **Source(s)** |
| --- | --- |
| \| \| V11.3 Personal history of alcoholism \| \| --- \| \| V15.82 Personal history of tobacco use \| \| V65.42 Counseling on substance use and abuse \| \| V79.1 ALCOHOLISM (Screening for) \| \|  \| \| 303 ALCOHOL DEPENDENCE SYNDROME \| \| 303.0 Acute alcoholic intoxication* \| \| 303.9 Other and unspecified alcohol dependence* \| \|  \| \| 304 DRUG DEPENDENCE* \| \| 304.0 MORPHINE TYPE (304.0 Opioid type dependence)* \| \| 304.1 BARBITURATE TYPE (304.1 Sedative, hypnotic or anxiolytic dependence)* \| \| 304.2 COCAINE* \| \| 304.3 CANNABIS* \| \| 304.4 AMPHETAMINE TYPE AND OTHER PSYCHOSTIMULANTS* \| \| 304.5 HALLUCINOGENS* \| \| 304.6 OTHER SPECIFIED DRUG DEPENDENCE* \| \| 304.7 COMBINATIONS OF MORPHINE TYPE DRUG WITH ANY OTHER (304.7 Combinations of opioid type drug with any other drug dependence)* \| \| 304.8 COMBINATIONS EXCLUDING MORPHINE TYPE DRUG (304.8 Combinations of drug dependence excluding opioid type drug)* \| \| 304.9 UNSPECIFIED* \| \|  \| \| 305 NONDEPENDENT ABUSE OF DRUGS* \| \| 305.0 ALCOHOL* \| \| 305.1 TOBACCO* \| \| 305.2 CANNABIS * \| \| 305.3 HALLUCINOGENS* \| \| 305.4 BARBITURATES AND TRANQUILLIZERS (305.4 Nondependent sedative, hypnotic or anxiolytic abuse)* \| \| 305.5 MORPHINE TYPE (305.5 Nondependent opioid abuse)* \| \| 305.6 COCAINE TYPE* \| \| 305.7 AMPHETAMINE TYPE (305.7 Nondependent amphetamine or related acting sympathomimetic abuse)* \| \| 305.8 ANTIDEPRESSANTS* \| \| 305.9 OTHER, MIXED OR UNSPECIFIED* \| \|  \| \| NON-SPECIFIC ABNORMAL FINDINGS \| \| 790.3 EXCESSIVE BLOOD LEVEL OF ALCOHOL \| \|  \| \| **Urine drug screen** \| \|  \| \| **Cocaine** \| \| Cocaine presence in urine \| \| Cocaine [Presence] in Urine by Confirmatory method \| \| Cocaine [Presence] in Urine by Screen method \| \|  \| \| **Benzodiazepines** \| \| Benzodiazepines [Presence] in Urine \| \| Benzodiazepines [Presence] in Urine by Confirmatory method \| \| Benzodiazepines [Presence] in Urine by Screen method \| \|  \| \| **Opiates** \| \| Opiates [Presence] in Urine \| \| Opiates [Presence] in Urine by Confirmatory method \| \| Opiates [Presence] in Urine by Screen method \| \|  \| \| **Amphetamines** \| \| Amphetamines [Presence] in Urine by Confirmatory method \| \| Amphetamines [Presence] in Urine by Screen method \| \| Amphetamines [Presence] in Urine \| \|  \| \| **Barbiturates** \| \| Barbiturates [Presence] in Urine by Screen method \| \|  \| \| **Cannabis** \| \| Cannabinoids [Presence] in Urine by Screen method \| \|  \| \| **Hallucinogens** \| \|  \| \| **Ethanol** \| \| Ethanol [mass/volume] in Serum or Plasma \| \| Ethanol [Presence] in Urine by Screen method \| \| Ethanol [Presence] in Urine \| \| Ethanol [Moles/volume] in Serum or Plasma \| \| Active drug use \| \| Addiction clinic \| \| Alcohol \| \| Alcohol abuse \| \| Alcohol dependence \| \| Alcohol excessive \| \| Alcohol misuse \| \| Alcoholism \| \| Benzodiazepine misuse \| \| Chemical dependency \| \| Cocaine abuse \| \| Continuous drinking \| \| Drug and alcohol testing \| \| Drug abuse \| \| drug addiction \| \| Drug overdose \| \| Drug screen \| \| Drug seeking \| \| Drug withdrawal \| \| Etoh abuse \| \| Injection drug use \| \| IVDU \| \| Marijuana abuse \| \| Methadone \| \| Methadone maintenance \| \| MMT \| \| Narcotic tolerance \| \| nicotine addiction \| \| Opiate abuse \| \| Opiate addiction \| \| Opioid dependence \| \| Opiate overdose \| \| Opiate use disorder \| \| Opioid type dependence \| \| Polydrug \| \| Polysubstance abuse \| \| Polysubstance dependence \| \| PPRM (prescription pain reliever misuse) \| \| Safe supply \| \| Severe alcohol use disorder \| \| Smoker \| \| Stimulant dependence \| \| Substance abuse \| \| substance dependence \| \| Substance misuse \| \| Substance use \| \| substance use disorder \| \| Tobacco abuse \| \| Tobacco dependence \| \| Urine drug screen \| \| Withdrawal \| \| Withdrawal management \| \| Withdrawal symptoms \| \|  \| \| \| --- \| --- \| --- \| --- \| --- \| --- \| --- \| --- \| --- \| --- \| --- \| --- \| --- \| --- \| --- \| --- \| --- \| --- \| --- \| --- \| --- \| --- \| --- \| --- \| --- \| --- \| --- \| --- \| --- \| --- \| --- \| --- \| --- \| --- \| --- \| --- \| --- \| --- \| --- \| --- \| --- \| --- \| --- \| --- \| --- \| --- \| --- \| --- \| --- \| --- \| --- \| --- \| --- \| --- \| --- \| --- \| --- \| --- \| --- \| --- \| --- \| --- \| --- \| --- \| --- \| --- \| --- \| --- \| --- \| --- \| --- \| --- \| --- \| --- \| --- \| --- \| --- \| --- \| --- \| --- \| --- \| --- \| --- \| --- \| --- \| --- \| --- \| --- \| --- \| --- \| --- \| --- \| --- \| --- \| --- \| --- \| --- \| --- \| --- \| --- \| --- \| --- \| --- \| --- \| --- \| --- \| --- \| --- \| --- \| --- \| --- \| --- \| --- \| --- \| --- \| --- \| --- \| --- \| --- \| --- \| --- \| --- \| --- \| --- \| --- \| --- \| | \| **ICD9-CM** \| \| --- \| \| **ICD9-CM** \| \| **ICD9-CM/TERMWORKS** \| \| **ICD9/TERMWORKS** \| \|  \| \| **ICD9/TERMWORKS** \| \| **ICD9-CM** \| \| **ICD9-CM/TERMWORKS** \| \|  \| \| **ICD9/TERMWORKS** \| \| **ICD9** \| \| **ICD9** \| \| **ICD9** \| \| **ICD9** \| \| **ICD9** \| \| **ICD9** \| \| **ICD9/TERMWORKS** \| \| **ICD9/TERMWORKS** \| \| **ICD9/TERMWORKS** \| \| **ICD9/TERMWORKS** \| \|  \| \| **ICD9/TERMWORKS** \| \| **ICD9/TERMWORKS** \| \| **ICD9** \| \| **ICD9** \| \| **ICD9** \| \| **ICD9** \| \| **ICD9** \| \| **ICD9** \| \| **ICD9** \| \| **ICD9** \| \| **ICD9/TERMWORKS** \| \|  \| \|  \| \| **TERMWORKS** \| \|  \| \| **LOINC 12286-1** \| \|  \| \|  \| \| **LOINC 3397-7** \| \| **LOINC 19360-7** \| \| **LOINC 19359-9** \| \|  \| \|  \| \| **LOINC 3390-2** \| \| **LOINC 16195-0** \| \| **LOINC 14316-4** \| \|  \| \|  \| \| **LOINC 3879-4** \| \| **LOINC 18390-5** \| \| **LOINC 19295-5** \| \|  \| \|  \| \| **LOINC 16369-1** \| \| **LOINC 19261-7** \| \| **LOINC 3349-8** \| \|  \| \|  \| \| **LOINC 19270-8** \| \|  \| \|  \| \| **LOINC 18282-4** \| \|  \| \| **LOINC 12295-2** \| \|  \| \|  \| \| **LOINC 5643-2** \| \| **LOINC 42242-8** \| \| **LOINC 5644-0** \| \| **LOINC 14719-9** \| \|  \| \| **CPCSSN Free Text** \| \| **CPCSSN Free Text** \| \| **CPCSSN Free Text** \| \| **CPCSSN Free Text** \| \| **CPCSSN Free Text** \| \| **CPCSSN Free Text** \| \| **CPCSSN Free Text** \| \| **CPCSSN Free Text** \| \| **CPCSSN Free Text** \| \| **CPCSSN Free Text** \| \| **CPCSSN Free Text** \| \| **CPCSSN Free Text** \| \| **CPCSSN Free Text** \| \| **CPCSSN Free Text** \| \| **CPCSSN Free Text** \| \| **CPCSSN Free Text** \| \| **CPCSSN Free Text** \| \| **CPCSSN Free Text** \| \| **CPCSSN Free Text** \| \| **CPCSSN Free Text** \| \| **CPCSSN Free Text** \| \| **CPCSSN Free Text** \| \| **CPCSSN Free Text** \| \| **CPCSSN Free Text** \| \| **CPCSSN Free Text** \| \| **CPCSSN Free Text** \| \| **CPCSSN Free Text** \| \| **CPCSSN Free Text** \| \| **CPCSSN Free Text** \| \| **CPCSSN Free Text** \| \| **CPCSSN Free Text** \| \| **CPCSSN Free Text** \| \| **CPCSSN Free Text** \| \| **CPCSSN Free Text** \| \| **CPCSSN Free Text** \| \| **CPCSSN Free Text** \| \| **CPCSSN Free Text** \| \| **CPCSSN Free Text** \| \| **CPCSSN Free Text** \| \| **CPCSSN Free Text** \| \| **CPCSSN Free Text** \| \| **CPCSSN Free Text** \| \| **CPCSSN Free Text** \| \| **CPCSSN Free Text** \| \| **CPCSSN Free Text** \| \| **CPCSSN Free Text** \| \| **CPCSSN Free Text** \| \| **CPCSSN Free Text** \| \| **CPCSSN Free Text** \| \| **CPCSSN Free Text** \| \| **CPCSSN Free Text** \| \| **CPCSSN Free Text** \| \| **CPCSSN Free Text** \| |

Termworks search terms: substance, alcohol, smoking, addiction, drugs
