# Supplementary material for: Capturing factors associated with frailty using routinely collected electronic medical record data in British Columbia, Canada, primary care settings
Source: Prim Health Care Res Dev. 2025 May 8;26:e41. doi: 10.1017/S1463423625000337 (PMC12099269; doi:10.1017/S1463423625000337)
Supplement: Thandi et al. supplementary material 2 — Thandi et al. supplementary material [file S1463423625000337sup002.docx]

# Summary of Frequencies of Free Text Terms

This document provides a summary of how often each free text term appeared in the two data fields searched:

**Data Field 1 = “Reason_orig” Data Field in Encounter Data Table**

**Data Field 2 = “DiagnosisText_orig” Data Field in EncounterDiagnosis Data Table**

**eFI Frailty Factors (n=36)**

**Activity Limitation**

**Anaemia and Haematinic Deficiency**

**Arthritis**

**Atrial Fibrillation**

**Cerebrovascular Disease**

**Chronic Kidney Disease**

**Diabetes**

**Dizziness**

**Dyspnea**

**Falls**

**Foot Problems**

**Fragility Fracture**

**Hearing Impairment**

**Heart Failure**

**Heart Valve Disease**

**Housebound**


**Hypertension**

**Hypotension/Syncope**

**Ischemic Heart Disease**

**Memory and/or Cognitive Problems**

**Mobility and Transfer Problems**


**Osteoporosis**

**Parkinsonism and Tremor**

**Peptic Ulcer**

**Peripheral Vascular Disease**

**Polypharmacy**

**Requirement for Care**

**Respiratory Disease**

**Skin Ulcer**

**Sleep Disturbance**

**Social Vulnerability**

##

**Thyroid Disorder**

**Urinary Incontinence**

**Urinary System Disease**

##

**Visual Impairment**

**Weight Loss and/or Anorexia**

**Additional Frailty Factors Suggested by Study 1 Participants**

**Cancer**

**Challenges to Healthcare Access**

| **Free Text Term** | **Data Field 1** | **Data Field 2** | **Total** | **Notes for Uniquness of Terms** |
| --- | --- | --- | --- | --- |
| n/a | n/a | n/a | n/a |  |

**Chronic Pain/Back Pain**

**Communication Challenges**

**Fecal Incontinence**

**Inadequate Diet and Nutrition**

**Liver Failure/Cirrhosis**

**Mental Health Challenges**

##

**Medication Noncompliance**

**Poverty/Financial Difficulties**

**Race/Ethnic Disparity**

**Sedentary/Low Activity Levels**

**Substance Use/Misuse**
